# Supplementary material for: The Casual Association Inference for the Chain of Falls Risk Factors-Falls-Falls Outcomes: A Mendelian Randomization Study
Source: Healthcare (Basel). 2023 Jun 29;11(13):1889. doi: 10.3390/healthcare11131889 (PMC10340577; doi:10.3390/healthcare11131889)
Supplement: Supplementary file 1 [file healthcare-11-01889-s001.zip › healthcare-2448416-supplementary.pdf]

Table S1. Summary genetic instruments between exposures and outcomes.

Exposure	Outcome	SNP	Chromosome	Effect_allele	Other_effect	BETA.exposure	BETA.outcome	SE.exposure	SE.outcome	*P*.exposure	*P*.outcome	R^2	F-statistic
Weight	Falls	rs7550173	1	T	A	0.041	0.00305053	0.007	0.00121498	5.20E-09	0.012	0.000589	35.70830895
Weight	Falls	rs1516728	3	A	T	0.038	0.0032153	0.0075	0.00138889	3.95E-07	0.021	0.00044	26.67178741
Weight	Falls	rs1991431	3	A	G	0.047	-0.000729883	0.0064	0.001192	3.21E-13	0.54	0.000924	56.02522937
Weight	Falls	rs585026	3	C	T	0.037	-0.00288306	0.0071	0.00128771	1.26E-07	0.025	0.000466	28.24643717
Weight	Falls	rs6767671	3	T	G	0.034	0.00170169	0.0065	0.00119852	1.52E-07	0.16	0.000469	28.42408172
Weight	Falls	rs11938781	4	C	T	-0.051	0.000229555	0.0093	0.00159758	3.80E-08	0.89	0.000515	31.24081323
Weight	Falls	rs13130484	4	T	C	0.037	0.00117472	0.0068	0.00119656	5.43E-08	0.33	0.000507	30.76047781
Weight	Falls	rs1712379	4	A	G	0.037	0.00215013	0.007	0.00128414	1.28E-07	0.0940005	0.000479	29.02884247
Weight	Falls	rs7666450	4	T	C	0.039	-0.00074657	0.0066	0.00119319	6.18E-09	0.53	0.000598	36.27596772
Weight	Falls	rs3798560	6	C	T	0.04	0.000640259	0.0078	0.00151531	3.58E-07	0.67	0.000451	27.32783759
Weight	Falls	rs389883	6	T	G	-0.038	0.000166127	0.0071	0.00126123	5.76E-08	0.9	0.000492	29.79237009
Weight	Falls	rs6569648	6	T	C	-0.039	0.00194478	0.0075	0.00138487	1.68E-07	0.16	0.000464	28.09885525
Weight	Falls	rs16905212	8	C	T	-0.038	0.00124559	0.007	0.00128107	7.38E-08	0.33	0.000505	30.61339828
Weight	Falls	rs12577643	11	T	A	-0.035	0.00121282	0.007	0.00128831	4.72E-07	0.35	0.000428	25.95893463
Weight	Falls	rs10875976	12	A	G	0.032	-0.00142966	0.0064	0.00118564	4.95E-07	0.23	0.000429	25.9713982
Weight	Falls	rs7970350	12	T	C	-0.047	0.00181249	0.0064	0.00118296	1.80E-13	0.13	0.000924	56.02426872
Weight	Falls	rs201762	13	G	T	-0.052	-0.000738402	0.0076	0.0014155	1.28E-11	0.6	0.000802	48.63175374
Weight	Falls	rs2241423	15	A	G	-0.04	0.00320968	0.0078	0.00141204	2.93E-07	0.0230001	0.000451	27.3259628
Weight	Falls	rs7498665	16	G	A	0.035	-0.000700615	0.0065	0.00120873	1.18E-07	0.56	0.000498	30.17396744
Weight	Falls	rs6567160	18	C	T	0.063	0.00225129	0.0076	0.00139951	1.24E-16	0.11	0.001177	71.38292552
Weight	Falls	rs1406948	20	A	G	0.036	-7.18E-05	0.0066	0.00122748	6.47E-08	0.95	0.00051	30.90811025
Height	Falls	rs1074078	1	T	C	0.018	-0.000854314	0.0032	0.00125664	2.80E-08	0.5	0.000126	31.89321611
Height	Falls	rs11205303	1	C	T	0.053	0.000313916	0.0035	0.00120535	2.20E-50	0.79	0.000996	252.6218222
Height	Falls	rs11799609	1	T	G	0.026	0.00148554	0.0042	0.0016204	1.10E-09	0.36	0.000152	38.62976958

Height	Falls	rs12065210	1	T	C	0.036	0.00296325	0.005	0.00201947	6.40E-13	0.14	0.000206	52.06178353
Height	Falls	rs12078328	1	C	T	0.022	0.00477754	0.004	0.00167573	7.30E-08	0.00439997	0.000119	30.25740649
Height	Falls	rs12097239	1	A	G	-0.033	0.00247929	0.0034	0.00139246	1.20E-22	0.0749998	0.000372	94.31027113
Height	Falls	rs1244981	1	A	G	0.025	-0.000390632	0.0041	0.00161561	1.20E-09	0.81	0.000148	37.38185441
Height	Falls	rs12754832	1	G	A	-0.019	-0.00225471	0.0035	0.00144801	5.50E-08	0.12	0.000116	29.50503339
Height	Falls	rs1325596	1	A	G	0.025	4.16E-05	0.0029	0.00119038	9.70E-18	0.97	0.000295	74.64932765
Height	Falls	rs1409055	1	T	C	0.016	0.0025802	0.003	0.00119956	2.00E-07	0.0309999	0.000113	28.65658048
Height	Falls	rs1572414	1	C	T	-0.021	-0.000816147	0.0038	0.00162955	4.90E-08	0.62	0.000121	30.54897078
Height	Falls	rs17257113	1	A	G	0.027	-0.000642452	0.0038	0.00155614	3.80E-12	0.68	0.000199	50.4937355
Height	Falls	rs17277008	1	C	T	0.039	0.000767286	0.0032	0.00127455	4.10E-34	0.55	0.000594	150.636331
Height	Falls	rs17369123	1	T	C	0.031	-0.000449027	0.0038	0.00151619	1.40E-16	0.77	0.000263	66.58305465
Height	Falls	rs17380127	1	C	A	-0.018	-0.00215653	0.0033	0.00129642	6.30E-08	0.0959997	0.000118	29.84468047
Height	Falls	rs17391694	1	T	C	0.043	0.00332981	0.0053	0.00171572	3.90E-16	0.0519996	0.000289	73.12554733
Height	Falls	rs1752388	1	C	A	-0.03	-0.00422302	0.0046	0.00187719	7.40E-11	0.0239999	0.000168	42.54198318
Height	Falls	rs1877454	1	T	C	0.017	-0.00131522	0.0033	0.00128541	1.90E-07	0.31	0.000106	26.77393923
Height	Falls	rs2268169	1	A	G	-0.017	-0.00101613	0.0031	0.00120099	5.30E-08	0.4	0.000128	32.37197904
Height	Falls	rs2273368	1	T	C	-0.023	-0.00176024	0.0035	0.00148852	1.60E-10	0.24	0.000171	43.19987645
Height	Falls	rs2284746	1	G	C	0.04	4.08E-05	0.003	0.00118275	1.20E-40	0.97	0.000705	178.7502669
Height	Falls	rs2806561	1	G	A	-0.027	0.0010701	0.0029	0.00118908	1.90E-20	0.37	0.000342	86.76439146
Height	Falls	rs2811594	1	G	A	0.024	-0.00121325	0.0032	0.0012259	4.10E-14	0.32	0.000226	57.25064193
Height	Falls	rs2815379	1	G	A	0.018	0.00148071	0.0033	0.00131834	1.60E-08	0.26	0.00012	30.51850488
Height	Falls	rs2970578	1	G	T	0.021	0.000397368	0.0031	0.00120913	1.00E-11	0.74	0.000184	46.62323098
Height	Falls	rs3814333	1	T	C	0.049	-0.00173543	0.0032	0.00128077	4.80E-51	0.18	0.000934	236.7222494
Height	Falls	rs425277	1	T	C	0.028	-0.000994832	0.0033	0.00132918	1.20E-17	0.450001	0.000285	72.21132395
Height	Falls	rs4655345	1	G	A	-0.024	-0.00120267	0.003	0.001209	5.20E-16	0.32	0.000253	64.02300203
Height	Falls	rs564914	1	T	A	0.024	-0.00208531	0.003	0.00120507	1.90E-15	0.0840001	0.000254	64.3120564

Height	Falls	rs593133	1	C	T	0.025	6.04E-05	0.0037	0.00143121	1.50E-11	0.97	0.000182	46.10647861
Height	Falls	rs599839	1	A	G	-0.019	-0.000377714	0.0035	0.00140819	1.60E-07	0.79	0.000117	29.53632335
Height	Falls	rs6600365	1	T	C	-0.027	-0.00165984	0.0029	0.0011911	1.70E-20	0.16	0.000342	86.69997814
Height	Falls	rs6658835	1	G	A	0.022	0.00209679	0.0034	0.00133763	3.60E-10	0.12	0.000169	42.79577704
Height	Falls	rs6684205	1	G	A	0.034	0.0020762	0.0032	0.00130716	1.50E-26	0.11	0.000447	113.3888832
Height	Falls	rs6691924	1	T	C	0.032	-0.00129279	0.005	0.0018643	2.40E-10	0.49	0.000164	41.49688435
Height	Falls	rs7517682	1	A	G	-0.023	0.00325449	0.003	0.00119751	3.60E-14	0.00659994	0.000234	59.26965182
Height	Falls	rs7536458	1	G	T	-0.043	0.00065807	0.0034	0.00134367	1.80E-36	0.62	0.000636	161.1384113
Height	Falls	rs7542242	1	T	C	-0.018	0.00251837	0.0033	0.00127529	3.80E-08	0.0479999	0.000117	29.76311186
Height	Falls	rs7551732	1	A	T	0.027	0.000434956	0.003	0.00120203	6.30E-20	0.719999	0.00032	81.06785367
Height	Falls	rs9429088	1	A	T	-0.019	0.000915429	0.0029	0.00119252	1.10E-10	0.44	0.000171	43.20140241
Height	Falls	rs9782976	1	C	T	0.028	-0.000270332	0.005	0.0020688	1.90E-08	0.9	0.000124	31.3815582
Height	Falls	rs10445823	2	C	T	-0.047	0.00218965	0.0049	0.00199496	5.40E-22	0.27	0.000363	92.02149748
Height	Falls	rs10469992	2	T	C	0.019	0.00037747	0.0032	0.00130646	4.60E-09	0.77	0.000139	35.2743785
Height	Falls	rs10932619	2	C	T	-0.02	8.98E-05	0.0031	0.00120351	3.10E-10	0.94	0.000169	42.85976224
Height	Falls	rs11684404	2	C	T	0.032	0.000449039	0.0031	0.00124857	9.00E-25	0.719999	0.000421	106.7960071
Height	Falls	rs12693589	2	C	T	0.022	0.00310487	0.0034	0.00134899	9.10E-11	0.021	0.000165	41.89050874
Height	Falls	rs12694443	2	T	G	-0.026	0.00100696	0.003	0.00118538	3.20E-18	0.4	0.000299	75.66676831
Height	Falls	rs12986437	2	C	A	0.022	0.00528014	0.0035	0.00142538	2.30E-10	0.00021	0.000156	39.59179927
Height	Falls	rs12987566	2	T	C	0.024	-0.000848608	0.0033	0.0013421	1.20E-12	0.53	0.00021	53.19942601
Height	Falls	rs13014679	2	C	A	-0.04	0.00307245	0.0071	0.00273333	2.50E-08	0.26	0.000126	31.83740109
Height	Falls	rs13030174	2	C	A	-0.023	-0.000128655	0.0034	0.00139156	3.10E-11	0.93	0.000182	46.11518517
Height	Falls	rs13388725	2	G	A	0.018	-0.00114209	0.003	0.00120734	2.00E-09	0.34	0.000142	36.02175931
Height	Falls	rs1344632	2	C	T	-0.015	0.000433665	0.0029	0.0011816	2.30E-07	0.709999	0.000106	26.78357863
Height	Falls	rs1345128	2	G	C	-0.022	0.00121153	0.0033	0.00129087	5.20E-11	0.35	0.000177	44.8335068
Height	Falls	rs1370394	2	G	T	0.016	-0.0012203	0.0031	0.00126084	4.90E-07	0.33	0.000105	26.65070241

Height	Falls	rs1405649	2	A	G	0.039	0.000685378	0.0056	0.00218503	6.30E-12	0.75	0.000201	50.79405287
Height	Falls	rs1545552	2	G	A	0.029	-0.000931315	0.0034	0.00130738	5.40E-18	0.48	0.000291	73.61993642
Height	Falls	rs17416284	2	T	A	-0.022	-0.00140559	0.0031	0.0012641	2.90E-12	0.27	0.000199	50.53679275
Height	Falls	rs17427088	2	T	C	0.018	0.00100113	0.0034	0.00138674	7.20E-08	0.47	0.000111	28.03974839
Height	Falls	rs17511102	2	T	A	0.053	2.40E-05	0.0057	0.00207937	9.10E-21	0.99	0.000366	92.64907111
Height	Falls	rs2120335	2	A	G	-0.019	0.000446363	0.003	0.00121417	8.40E-10	0.709999	0.000159	40.18758757
Height	Falls	rs2166898	2	A	G	-0.027	-0.000691529	0.0041	0.00157949	8.60E-11	0.66	0.00018	45.57691733
Height	Falls	rs2194736	2	C	T	0.027	-0.000434658	0.0031	0.00127553	8.60E-18	0.73	0.000299	75.87495668
Height	Falls	rs2222413	2	G	A	-0.016	0.00359317	0.0031	0.00124453	4.20E-07	0.00389996	0.000105	26.64712384
Height	Falls	rs2289195	2	A	G	0.038	0.00351759	0.003	0.00120291	2.40E-37	0.00350002	0.00064	162.08781
Height	Falls	rs2853406	2	G	A	-0.05	-0.00159825	0.0053	0.00219341	5.80E-21	0.47	0.000352	89.17039027
Height	Falls	rs3103267	2	C	A	0.038	0.00017094	0.0033	0.00131158	1.40E-31	0.9	0.000523	132.6353705
Height	Falls	rs3755196	2	G	A	0.018	-0.00256681	0.0032	0.00126916	3.40E-08	0.0430002	0.000126	31.90794598
Height	Falls	rs3791679	2	G	A	-0.06	-0.00444716	0.0035	0.00141046	2.40E-67	0.0016	0.001164	295.1161051
Height	Falls	rs3885668	2	T	C	-0.022	0.00260245	0.003	0.00119578	8.40E-13	0.0299999	0.000214	54.28248492
Height	Falls	rs445151	2	T	C	0.022	-0.00167782	0.0038	0.00141356	5.10E-09	0.24	0.000134	33.84444274
Height	Falls	rs4675945	2	T	G	-0.02	-0.00222659	0.003	0.0012235	3.30E-11	0.0690001	0.000176	44.46814571
Height	Falls	rs5013667	2	G	A	-0.021	0.00104137	0.0042	0.00165322	3.30E-07	0.53	0.0001	25.33417352
Height	Falls	rs6435143	2	C	A	-0.019	0.00085815	0.003	0.00119145	1.50E-10	0.47	0.000159	40.16343905
Height	Falls	rs6544650	2	G	A	0.018	-0.0023665	0.003	0.00119425	4.20E-09	0.0479999	0.000143	36.1917569
Height	Falls	rs6713543	2	T	C	0.038	0.00430461	0.0055	0.00218686	3.90E-12	0.0490004	0.000189	47.7832665
Height	Falls	rs6713865	2	G	A	-0.031	0.00165476	0.004	0.00152969	5.30E-15	0.28	0.000238	60.33931865
Height	Falls	rs6739772	2	G	A	0.02	0.00038568	0.0032	0.00130854	1.40E-09	0.77	0.000156	39.40350775
Height	Falls	rs6750552	2	T	C	-0.016	0.00226333	0.0031	0.00126477	2.00E-07	0.0739997	0.000106	26.79825615
Height	Falls	rs7567288	2	C	T	0.029	0.000449818	0.0038	0.00150921	3.00E-14	0.77	0.00023	58.29853943
Height	Falls	rs7586126	2	G	A	-0.036	-0.00344932	0.0053	0.00223051	1.00E-11	0.12	0.000182	46.1488941

Height	Falls	rs7590738	2	A	G	-0.022	-0.0015012	0.003	0.00119653	1.10E-13	0.21	0.000214	54.26713023
Height	Falls	rs897080	2	T	C	-0.028	0.000619904	0.0034	0.00140811	1.60E-16	0.66	0.000271	68.69418526
Height	Falls	rs997400	2	T	C	-0.026	0.00185815	0.0029	0.00118987	1.00E-18	0.12	0.000318	80.49681786
Height	Falls	rs1133415	3	A	G	-0.019	0.000636376	0.0029	0.00118507	2.90E-10	0.59	0.000171	43.20329077
Height	Falls	rs12330322	3	T	C	-0.034	-0.000685754	0.0035	0.00143108	3.30E-22	0.630001	0.000373	94.40387316
Height	Falls	rs13078528	3	A	G	0.045	0.00296584	0.0064	0.00274562	1.40E-12	0.28	0.000195	49.50943162
Height	Falls	rs13084192	3	T	A	-0.023	0.00203656	0.0031	0.00122684	1.40E-13	0.0969996	0.000217	55.06987292
Height	Falls	rs13093019	3	G	A	-0.026	-0.000532827	0.0047	0.00186928	6.60E-08	0.780001	0.000121	30.63389115
Height	Falls	rs1393786	3	A	C	-0.027	0.0020649	0.0033	0.00133429	1.00E-15	0.12	0.000264	66.97361461
Height	Falls	rs1511886	3	T	C	0.017	0.00208659	0.0031	0.0012738	5.40E-08	0.1	0.000119	30.08246106
Height	Falls	rs17469356	3	G	A	-0.02	0.00259687	0.0039	0.00158127	2.40E-07	0.1	0.000104	26.31427936
Height	Falls	rs17806888	3	C	T	-0.034	-0.00434885	0.0048	0.0018359	2.90E-12	0.0179999	0.000204	51.69621303
Height	Falls	rs1797625	3	T	A	0.019	4.37E-05	0.003	0.00125486	4.70E-10	0.97	0.000158	40.11982296
Height	Falls	rs2360960	3	G	C	0.016	0.00204965	0.003	0.0011885	1.20E-07	0.0850002	0.000114	28.92185848
Height	Falls	rs2581830	3	C	T	-0.031	0.00121459	0.003	0.00120354	4.40E-25	0.31	0.000422	106.8267023
Height	Falls	rs2597513	3	T	C	-0.039	-0.00232927	0.0048	0.0019409	3.10E-16	0.23	0.000261	66.06701039
Height	Falls	rs2633761	3	A	G	0.016	0.00301875	0.0029	0.00120214	2.60E-08	0.012	0.000121	30.58788862
Height	Falls	rs3752904	3	T	C	-0.015	0.00225629	0.003	0.00118395	4.70E-07	0.0569994	9.87E-05	25.01204753
Height	Falls	rs4325879	3	T	C	-0.021	-0.000251009	0.0035	0.00135958	3.20E-09	0.85	0.000144	36.44971039
Height	Falls	rs4378999	3	A	T	-0.034	0.00251633	0.0048	0.00191237	6.00E-13	0.19	0.000201	50.81439336
Height	Falls	rs4686904	3	T	C	-0.021	-0.00415726	0.0031	0.0012374	2.50E-11	0.000779992	0.000181	45.93304053
Height	Falls	rs519384	3	A	T	0.031	-0.00148158	0.0033	0.00131405	8.60E-22	0.26	0.000352	89.06435318
Height	Falls	rs6439168	3	G	A	0.037	-0.000715909	0.0036	0.00142539	7.70E-25	0.62	0.000417	105.7019916
Height	Falls	rs6441170	3	C	T	0.022	-0.000949464	0.003	0.0012284	9.70E-13	0.44	0.000212	53.79349408
Height	Falls	rs720390	3	A	G	0.035	-0.0013948	0.0031	0.00122252	1.20E-29	0.25	0.000506	128.2241667
Height	Falls	rs724016	3	G	A	0.078	-0.000674729	0.0029	0.00118973	3.20E-158	0.57	0.002852	724.3281676

Height	Falls	rs7617596	3	T	C	0.019	-0.00158989	0.0037	0.00148009	2.10E-07	0.28	0.000104	26.38169515
Height	Falls	rs7652177	3	G	C	0.038	-0.000705596	0.0029	0.0011836	2.70E-39	0.55	0.000682	172.752754
Height	Falls	rs9816693	3	C	G	0.031	-0.00290029	0.004	0.00153462	3.20E-15	0.0589997	0.000237	60.09215628
Height	Falls	rs9825936	3	T	C	0.019	-0.00146497	0.0034	0.00139669	1.20E-08	0.29	0.000123	31.238487
Height	Falls	rs9825951	3	A	T	-0.022	0.00145634	0.0031	0.00123083	2.60E-12	0.24	0.000201	50.82391188
Height	Falls	rs9834893	3	C	G	-0.038	-0.000606427	0.0058	0.00238983	6.60E-11	0.8	0.000169	42.93661643
Height	Falls	rs9835332	3	C	G	-0.028	-0.00118909	0.0029	0.00118626	4.40E-22	0.32	0.00037	93.63454656
Height	Falls	rs11722554	4	A	G	-0.063	-0.002629	0.0098	0.00309909	1.50E-10	0.4	0.000246	62.24574748
Height	Falls	rs11726922	4	C	G	-0.018	2.71E-05	0.0033	0.00135405	1.20E-07	0.98	0.000117	29.76111362
Height	Falls	rs11735005	4	T	G	0.017	-0.00388153	0.003	0.00119528	4.30E-09	0.0012	0.000127	32.14525067
Height	Falls	rs12513181	4	A	C	-0.02	0.000647941	0.0033	0.00135911	3.20E-09	0.630001	0.000145	36.74037439
Height	Falls	rs12639764	4	C	T	-0.027	-0.00330024	0.003	0.00121617	1.60E-19	0.00669993	0.00032	81.11336644
Height	Falls	rs13150868	4	T	G	0.017	-0.00273869	0.003	0.00119333	2.20E-08	0.0219999	0.000133	33.78119191
Height	Falls	rs13152352	4	A	C	-0.074	0.000636507	0.0041	0.00161245	6.70E-72	0.69	0.001285	325.8163632
Height	Falls	rs1562975	4	A	G	0.025	0.00286632	0.0032	0.00129604	5.50E-15	0.0269998	0.000241	61.12565521
Height	Falls	rs16994718	4	T	C	-0.025	0.00212667	0.0041	0.00175721	1.50E-09	0.23	0.000147	37.3330985
Height	Falls	rs17556750	4	A	C	0.046	0.00262426	0.0032	0.00130711	8.30E-48	0.0449997	0.000819	207.6185271
Height	Falls	rs1812175	4	G	A	0.079	0.00230126	0.004	0.00157776	2.10E-86	0.14	0.001539	390.3476097
Height	Falls	rs1996422	4	G	A	0.022	-0.00116047	0.0033	0.00130527	2.80E-11	0.37	0.000177	44.80174618
Height	Falls	rs2035901	4	G	A	0.031	-0.00336088	0.003	0.00118785	2.40E-25	0.00470002	0.000427	108.0818296
Height	Falls	rs2247341	4	A	G	0.027	0.00240161	0.0031	0.00125105	1.80E-18	0.0549997	0.0003	75.89473725
Height	Falls	rs2302580	4	T	C	-0.029	-0.000160649	0.0036	0.00120513	4.20E-15	0.89	0.000339	85.84949313
Height	Falls	rs2310357	4	C	T	0.018	0.000121195	0.0034	0.00132781	2.00E-07	0.93	0.000119	30.02423154
Height	Falls	rs3796529	4	T	C	0.031	0.000995135	0.0037	0.00152213	6.30E-17	0.51	0.000278	70.37309726
Height	Falls	rs6813055	4	T	A	-0.017	-0.00106514	0.0029	0.00118227	1.50E-08	0.37	0.000136	34.40990705
Height	Falls	rs6838153	4	G	A	0.022	0.00021939	0.0031	0.0012685	2.60E-12	0.86	0.000199	50.38509115

Height	Falls	rs713140	4	G	A	-0.018	-0.000283703	0.003	0.00123927	4.70E-09	0.82	0.000142	36.0288759
Height	Falls	rs955748	4	G	A	0.028	0.00246332	0.0034	0.00138346	3.10E-16	0.0749998	0.000268	67.84873172
Height	Falls	rs9993613	4	G	T	-0.03	-0.0012914	0.0029	0.00118545	4.50E-24	0.28	0.000424	107.3349803
Height	Falls	rs10037280	5	T	A	-0.024	0.000917336	0.0044	0.00174599	6.90E-08	0.6	0.000119	30.2215825
Height	Falls	rs10037512	5	C	T	-0.03	-0.00134582	0.003	0.00118432	5.80E-24	0.26	0.000395	100.1866985
Height	Falls	rs10463065	5	G	C	-0.043	0.00706354	0.0076	0.00293438	1.80E-08	0.016	0.000134	34.06950728
Height	Falls	rs10516138	5	A	G	-0.045	0.000658356	0.0062	0.00185006	2.70E-13	0.719999	0.000245	62.00010208
Height	Falls	rs11740780	5	C	G	0.028	-2.63E-05	0.0034	0.00134054	1.40E-16	0.98	0.000268	67.89378347
Height	Falls	rs11950193	5	A	C	0.062	-0.00517842	0.0098	0.00279636	2.30E-10	0.064	0.000195	49.47497895
Height	Falls	rs11958779	5	A	G	-0.031	-7.60E-05	0.0032	0.00127858	1.70E-22	0.95	0.000378	95.89089254
Height	Falls	rs13185815	5	G	T	-0.041	-0.00674115	0.0056	0.00223595	2.70E-13	0.00259998	0.000212	53.65500422
Height	Falls	rs13361606	5	C	T	-0.016	0.000679706	0.0033	0.00128154	4.50E-07	0.6	9.36E-05	23.69801746
Height	Falls	rs153750	5	G	T	0.031	-0.000790032	0.0031	0.00123923	1.20E-23	0.52	0.000396	100.3192332
Height	Falls	rs165189	5	G	A	0.029	-0.000445724	0.0046	0.00170701	1.60E-10	0.79	0.000161	40.85287398
Height	Falls	rs17409588	5	C	T	0.019	0.00124057	0.0033	0.00126962	5.40E-09	0.33	0.000132	33.38415827
Height	Falls	rs249012	5	G	A	-0.019	-0.000726487	0.0032	0.00129678	1.70E-09	0.58	0.00014	35.44590971
Height	Falls	rs2662027	5	T	G	-0.033	0.000474217	0.0048	0.00194158	6.30E-12	0.81	0.000187	47.28746843
Height	Falls	rs2938772	5	A	G	-0.02	-0.00135349	0.0031	0.00124207	8.30E-11	0.28	0.000167	42.29899477
Height	Falls	rs31203	5	C	T	-0.032	-3.97E-06	0.0032	0.00128856	1.10E-23	1	0.000397	100.71094
Height	Falls	rs34651	5	T	C	-0.041	-0.00204438	0.0058	0.00218069	2.20E-12	0.35	0.000199	50.3888443
Height	Falls	rs3812039	5	C	T	-0.024	0.00274915	0.0033	0.00132436	2.20E-13	0.0379997	0.000209	52.92683155
Height	Falls	rs421239	5	G	A	0.017	0.000498174	0.0033	0.00132984	2.70E-07	0.709999	0.000106	26.760189
Height	Falls	rs4624820	5	A	G	0.018	-0.000228333	0.0029	0.0011861	1.00E-09	0.85	0.000153	38.82369453
Height	Falls	rs4868126	5	G	T	0.036	0.00131738	0.0032	0.00124288	2.80E-29	0.29	0.000513	130.0714918
Height	Falls	rs6869670	5	C	T	0.024	0.000936535	0.0041	0.0016478	4.50E-09	0.57	0.000135	34.29741039
Height	Falls	rs745749	5	G	A	-0.023	-0.0012328	0.0033	0.00126143	4.30E-12	0.33	0.000206	52.06587867

Height	Falls	rs7701414	5	G	A	0.037	0.000472077	0.003	0.00118666	1.30E-34	0.69	0.000603	152.7788392
Height	Falls	rs806100	5	C	T	-0.018	0.0008376	0.003	0.00118554	5.40E-10	0.48	0.000142	36.00739237
Height	Falls	rs9291926	5	G	T	-0.019	-0.00386756	0.0031	0.00118639	3.40E-10	0.00109999	0.000149	37.87019767
Height	Falls	rs9292468	5	C	T	-0.036	-0.00137758	0.003	0.00120815	1.50E-33	0.25	0.000569	144.1542359
Height	Falls	rs1047014	6	C	T	0.032	0.000945627	0.0036	0.001378	1.30E-18	0.49	0.000334	84.50425837
Height	Falls	rs10948222	6	C	T	0.031	-0.00107996	0.0033	0.00120592	9.80E-21	0.37	0.000374	94.88182386
Height	Falls	rs11154483	6	T	C	0.017	-0.00203659	0.0032	0.00128802	3.10E-07	0.11	0.000112	28.3663437
Height	Falls	rs1155939	6	A	C	0.042	-0.00150289	0.0029	0.00118311	9.60E-46	0.2	0.000829	210.0961913
Height	Falls	rs12211255	6	A	C	0.049	0.000683861	0.0048	0.00194855	1.80E-24	0.73	0.000416	105.4739108
Height	Falls	rs12214804	6	T	C	-0.084	-0.00162591	0.0057	0.0020698	1.50E-49	0.43	0.000865	219.3700694
Height	Falls	rs13196428	6	A	G	0.031	-0.000664402	0.006	0.00259166	3.70E-07	0.8	0.000112	28.31140213
Height	Falls	rs13210323	6	C	A	-0.037	-0.000476025	0.0034	0.00131891	1.10E-27	0.719999	0.00047	119.0412893
Height	Falls	rs1415701	6	A	G	-0.044	0.00162785	0.0036	0.00135299	1.50E-34	0.23	0.000606	153.6172803
Height	Falls	rs162965	6	G	A	0.016	0.00143207	0.0031	0.00127812	2.00E-07	0.26	0.000105	26.6448088
Height	Falls	rs16876334	6	T	C	-0.038	0.00287068	0.0068	0.00277298	2.10E-08	0.3	0.000126	31.87008881
Height	Falls	rs1832871	6	G	A	-0.025	-0.00197963	0.0031	0.00125351	1.80E-15	0.11	0.000258	65.28230576
Height	Falls	rs1855078	6	C	T	0.023	-0.000280533	0.0041	0.00168069	3.50E-08	0.87	0.000124	31.48092245
Height	Falls	rs2256183	6	G	A	-0.037	-0.000543865	0.003	0.00118175	2.50E-36	0.649999	0.000608	154.0870383
Height	Falls	rs2487663	6	G	A	0.021	0.000326473	0.0039	0.00155126	1.70E-07	0.83	0.000115	29.15708606
Height	Falls	rs2609334	6	C	T	-0.022	-0.000854162	0.0034	0.00141319	2.40E-10	0.55	0.000165	41.88422163
Height	Falls	rs2748483	6	T	A	-0.019	0.000523909	0.003	0.00119606	4.10E-10	0.66	0.00016	40.5195283
Height	Falls	rs310421	6	T	G	0.032	6.17E-05	0.0029	0.00118888	3.30E-27	0.96	0.000484	122.654223
Height	Falls	rs3812163	6	T	A	0.039	0.00150607	0.003	0.00118735	4.50E-39	0.2	0.000668	169.3423137
Height	Falls	rs389663	6	C	T	-0.022	-0.00128981	0.0031	0.00126657	2.50E-12	0.31	0.000199	50.38330014
Height	Falls	rs4246079	6	G	A	0.038	-0.00321365	0.005	0.00181595	4.50E-14	0.0769999	0.000244	61.80891514
Height	Falls	rs4279453	6	C	T	-0.019	0.000288534	0.003	0.00119597	7.00E-11	0.81	0.000159	40.16883817

Height	Falls	rs4371882	6	G	A	0.027	0.0032564	0.0038	0.00154968	1.10E-12	0.0359998	0.00021	53.11684662
Height	Falls	rs648831	6	T	C	0.031	-0.000379732	0.003	0.00119269	2.60E-26	0.75	0.000425	107.6820509
Height	Falls	rs6903732	6	A	C	0.017	0.00015461	0.003	0.00118662	2.10E-08	0.9	0.000127	32.14728356
Height	Falls	rs6903903	6	G	A	-0.017	-0.000291262	0.0032	0.00125908	1.10E-07	0.82	0.000112	28.48502991
Height	Falls	rs6919321	6	A	G	0.021	-0.00327197	0.003	0.00121602	4.70E-12	0.00710003	0.000195	49.36190336
Height	Falls	rs6920372	6	A	G	-0.025	0.000971521	0.0029	0.00120337	1.70E-17	0.42	0.000293	74.3485791
Height	Falls	rs7751325	6	T	C	0.017	5.46E-05	0.003	0.00118776	3.10E-08	0.96	0.000128	32.44402338
Height	Falls	rs7759938	6	T	C	-0.044	0.00262161	0.0032	0.00126698	2.60E-43	0.0389996	0.000753	190.8482694
Height	Falls	rs7774834	6	A	C	0.018	0.00147159	0.0029	0.00118496	4.90E-10	0.21	0.000153	38.69498886
Height	Falls	rs806794	6	G	A	-0.06	-0.00289885	0.0033	0.00132556	4.60E-74	0.0290001	0.001314	333.2294976
Height	Falls	rs852953	6	T	C	0.017	0.00124414	0.0032	0.00126324	1.90E-07	0.32	0.000111	28.23938006
Height	Falls	rs9384681	6	G	A	-0.041	0.0023576	0.0077	0.00292616	8.30E-08	0.42	0.000124	31.31058124
Height	Falls	rs9389986	6	A	T	-0.052	2.35E-05	0.0033	0.00130915	1.90E-56	0.99	0.00098	248.3717968
Height	Falls	rs9479130	6	C	A	0.031	0.000307759	0.003	0.00119094	5.10E-25	0.8	0.000424	107.4211528
Height	Falls	rs991946	6	T	C	-0.021	0.000156544	0.0029	0.00118524	8.40E-13	0.89	0.000209	52.83537387
Height	Falls	rs10236884	7	G	A	0.023	0.00041009	0.0031	0.00123681	8.50E-13	0.74	0.000226	57.34835916
Height	Falls	rs10262697	7	T	A	-0.02	-0.00247248	0.0038	0.00152427	2.70E-07	0.1	0.00011	27.75924593
Height	Falls	rs1043550	7	G	A	-0.016	8.95E-05	0.003	0.00119866	2.00E-07	0.94	0.000118	29.83806389
Height	Falls	rs10950949	7	C	A	-0.032	0.00158476	0.003	0.00120103	5.10E-26	0.19	0.00045	114.0357569
Height	Falls	rs1113765	7	A	G	-0.024	-0.00056198	0.0038	0.00155517	1.70E-10	0.719999	0.000158	39.90700063
Height	Falls	rs12706014	7	T	C	0.062	0.00182151	0.012	0.00486901	3.00E-07	0.709999	0.00011	27.78426658
Height	Falls	rs17141931	7	C	T	0.044	-0.00196733	0.0083	0.00299609	1.40E-07	0.51	0.000117	29.67865194
Height	Falls	rs17277546	7	A	G	0.039	-0.00520308	0.0072	0.00279103	5.10E-08	0.0619998	0.000124	31.4737253
Height	Falls	rs17807185	7	G	A	0.022	0.00100487	0.003	0.00121057	3.90E-13	0.41	0.000213	53.85815483
Height	Falls	rs2110001	7	G	C	0.032	-0.00122294	0.0035	0.0012998	4.00E-20	0.35	0.000341	86.39355765
Height	Falls	rs2347709	7	C	T	-0.016	-0.00237751	0.003	0.00121422	1.50E-07	0.05	0.000112	28.48008869

Height	Falls	rs273945	7	C	A	0.019	0.000787278	0.0031	0.00119846	1.40E-09	0.51	0.000149	37.72874024
Height	Falls	rs3807931	7	A	G	0.027	-0.00197672	0.0029	0.00119	1.20E-19	0.0969996	0.000342	86.76507722
Height	Falls	rs42039	7	T	C	0.068	-0.000619466	0.0034	0.00137825	3.80E-88	0.649999	0.001581	401.1943041
Height	Falls	rs4721810	7	C	A	-0.031	0.00218135	0.0044	0.00171062	5.50E-12	0.2	0.000204	51.68343577
Height	Falls	rs4722837	7	G	A	-0.017	-0.00037823	0.0031	0.00119199	2.20E-08	0.75	0.00012	30.50411306
Height	Falls	rs4725061	7	G	A	0.02	-0.00133298	0.0031	0.00119073	1.10E-10	0.26	0.000166	42.12962435
Height	Falls	rs552707	7	C	T	-0.046	-0.00194847	0.0032	0.00129956	9.30E-46	0.13	0.000821	208.02204
Height	Falls	rs6952113	7	A	G	-0.018	-0.000937918	0.003	0.00121448	1.20E-09	0.44	0.000142	36.01450329
Height	Falls	rs6962887	7	G	T	-0.023	0.00224751	0.0034	0.00128893	6.10E-11	0.0810009	0.000185	46.80532262
Height	Falls	rs6974574	7	T	A	0.03	-0.00219425	0.0034	0.00124254	9.90E-19	0.0769999	0.00031	78.50087488
Height	Falls	rs723149	7	G	A	-0.021	0.000771398	0.0032	0.00119325	5.10E-11	0.52	0.00018	45.538722
Height	Falls	rs798497	7	G	A	-0.057	0.000303276	0.0032	0.00129186	2.20E-71	0.81	0.001252	317.5785534
Height	Falls	rs822553	7	A	G	0.03	0.00111535	0.0039	0.00134453	2.20E-14	0.41	0.000257	65.15950297
Height	Falls	rs10283100	8	G	A	0.057	-0.00121302	0.0085	0.00258446	3.20E-11	0.64	0.000192	48.6503966
Height	Falls	rs1036821	8	A	G	-0.037	0.00103048	0.0032	0.00128804	1.10E-30	0.42	0.00053	134.3533972
Height	Falls	rs11167042	8	G	A	0.016	-0.0027779	0.0031	0.00121641	1.10E-07	0.0219999	0.000107	27.07994001
Height	Falls	rs13276054	8	C	G	-0.024	-0.000554166	0.0033	0.00128137	5.10E-13	0.67	0.00021	53.12980292
Height	Falls	rs1599473	8	T	G	-0.027	-0.00188627	0.0034	0.00138209	1.00E-14	0.17	0.00025	63.24481241
Height	Falls	rs17088190	8	T	C	-0.028	0.00151929	0.0034	0.00139043	9.80E-17	0.27	0.00027	68.34380651
Height	Falls	rs2313167	8	C	G	0.02	0.000299733	0.003	0.00118467	4.60E-11	0.8	0.000184	46.61772731
Height	Falls	rs2576578	8	A	T	0.017	-0.00111063	0.0029	0.00118648	7.00E-09	0.35	0.000136	34.39182377
Height	Falls	rs2925155	8	T	C	-0.023	0.00122898	0.0034	0.00135861	9.80E-12	0.37	0.000182	46.11885498
Height	Falls	rs429433	8	G	A	-0.046	0.00428326	0.0071	0.00275946	1.30E-10	0.12	0.000167	42.35134675
Height	Falls	rs4733724	8	G	A	-0.05	-0.00226436	0.0037	0.00146765	1.40E-41	0.12	0.000722	183.0856173
Height	Falls	rs4735677	8	T	A	0.037	0.000367673	0.0032	0.00131122	6.00E-30	0.780001	0.000528	133.7447381
Height	Falls	rs4875421	8	A	T	-0.019	0.00263178	0.0029	0.00119165	1.30E-10	0.0269998	0.000171	43.2782717

Height	Falls	rs4876357	8	C	T	-0.017	0.000943281	0.0029	0.00119151	4.30E-09	0.43	0.000136	34.40473846
Height	Falls	rs568610	8	T	C	0.022	-0.0002399	0.0034	0.0013888	1.40E-10	0.86	0.000166	42.08300942
Height	Falls	rs7000226	8	C	G	0.023	0.000133402	0.0034	0.00138267	3.80E-11	0.92	0.000181	45.77552308
Height	Falls	rs7007200	8	C	G	-0.017	0.000245486	0.0032	0.00129481	8.80E-08	0.85	0.000112	28.26830321
Height	Falls	rs9297806	8	T	C	-0.02	-0.000204376	0.0036	0.00145765	5.00E-08	0.89	0.000122	30.87590006
Height	Falls	rs9650315	8	T	G	-0.061	0.000136279	0.0045	0.00174913	1.50E-41	0.94	0.000726	184.0677558
Height	Falls	rs10119624	9	A	G	0.024	9.57E-05	0.0031	0.00125937	3.70E-14	0.94	0.000237	59.96550156
Height	Falls	rs10817960	9	A	G	0.023	-0.00230159	0.0045	0.00163387	4.20E-07	0.16	0.000116	29.34523934
Height	Falls	rs10868439	9	G	A	0.027	0.00107912	0.003	0.0011849	3.00E-19	0.36	0.000323	81.73901576
Height	Falls	rs11144688	9	A	G	-0.063	0.00160266	0.0063	0.00182085	5.70E-24	0.38	0.00044	111.4815141
Height	Falls	rs13302191	9	A	T	-0.025	-0.00162982	0.0033	0.00134179	1.40E-14	0.22	0.000227	57.43927233
Height	Falls	rs1576900	9	A	G	-0.019	-0.00156561	0.0033	0.00132924	6.60E-09	0.24	0.000132	33.46372388
Height	Falls	rs1742829	9	A	T	-0.037	-0.00295238	0.0055	0.00206982	2.30E-11	0.15	0.000181	45.86462175
Height	Falls	rs17743593	9	C	T	-0.03	-0.00291921	0.0058	0.00239529	1.90E-07	0.22	0.000107	27.14993453
Height	Falls	rs2149163	9	C	G	0.02	0.000573744	0.003	0.00121578	1.80E-11	0.64	0.000176	44.48325749
Height	Falls	rs2281645	9	G	A	-0.019	0.00100788	0.0032	0.00127771	1.10E-09	0.43	0.000139	35.27033787
Height	Falls	rs2778027	9	T	C	-0.027	0.00360748	0.0039	0.00151204	3.10E-12	0.017	0.00019	48.1075317
Height	Falls	rs3739707	9	A	C	-0.024	-0.00113894	0.0035	0.00136834	4.00E-12	0.41	0.000186	47.03693607
Height	Falls	rs7033487	9	C	T	-0.037	0.00181413	0.0036	0.00148622	1.10E-24	0.22	0.000417	105.7533809
Height	Falls	rs7033940	9	C	G	-0.024	-0.0016664	0.0044	0.00188924	4.70E-08	0.38	0.000117	29.76217148
Height	Falls	rs7043114	9	T	C	-0.029	0.000329381	0.0029	0.00119225	1.80E-22	0.780001	0.000395	100.0394967
Height	Falls	rs7466269	9	G	A	-0.033	-0.000936097	0.0031	0.00123965	1.00E-27	0.450001	0.000448	113.4224538
Height	Falls	rs7849585	9	T	G	0.036	0.00237501	0.0032	0.00125478	1.10E-29	0.0580003	0.000502	127.1170682
Height	Falls	rs7870253	9	A	T	0.042	-0.00268291	0.0035	0.0014308	3.30E-33	0.061	0.000568	144.0381014
Height	Falls	rs817300	9	A	G	-0.085	-0.000666305	0.0069	0.00225368	4.30E-34	0.77	0.000666	168.7126202
Height	Falls	rs8413	9	C	T	0.023	0.000132148	0.003	0.00119253	2.60E-14	0.91	0.000232	58.86795555

Height	Falls	rs958225	9	A	T	0.046	0.00182327	0.0079	0.00257322	6.80E-09	0.48	0.000145	36.69937472
Height	Falls	rs989393	9	C	T	-0.022	0.00328282	0.0032	0.00130548	3.30E-11	0.012	0.000187	47.31849462
Height	Falls	rs10749552	10	C	T	-0.016	0.00126102	0.0032	0.0012131	4.80E-07	0.3	0.0001	25.33782297
Height	Falls	rs10787959	10	G	A	-0.027	0.00117176	0.0033	0.00131612	1.90E-16	0.37	0.000266	67.29044187
Height	Falls	rs10794175	10	T	G	0.02	-0.00170894	0.003	0.00120118	6.40E-12	0.15	0.000176	44.53922522
Height	Falls	rs10995319	10	C	T	-0.019	0.000901685	0.0034	0.00139843	3.20E-08	0.52	0.000123	31.24157162
Height	Falls	rs11244755	10	T	C	0.018	-0.0030846	0.0032	0.00126822	1.90E-08	0.015	0.000125	31.78544312
Height	Falls	rs1171615	10	T	C	-0.022	0.00112764	0.0038	0.00140552	5.80E-09	0.42	0.000137	34.70840662
Height	Falls	rs12770214	10	C	G	0.017	0.00259879	0.0032	0.00125957	5.70E-08	0.0389996	0.000112	28.26763251
Height	Falls	rs12779328	10	T	C	-0.028	-0.00275484	0.0033	0.00131322	1.70E-17	0.0359998	0.000286	72.505055
Height	Falls	rs1614303	10	T	G	0.022	7.41E-05	0.0038	0.00152692	5.70E-09	0.96	0.000132	33.53733725
Height	Falls	rs1749824	10	A	C	-0.023	-0.00151029	0.003	0.00119347	1.50E-14	0.21	0.000232	58.88239104
Height	Falls	rs1923367	10	C	G	-0.03	-0.00270988	0.003	0.00118958	4.90E-24	0.0230001	0.000395	100.0738841
Height	Falls	rs2181834	10	T	G	0.022	0.000954266	0.0029	0.00119013	2.10E-14	0.42	0.000227	57.59942794
Height	Falls	rs2631676	10	G	A	0.028	0.00064476	0.0039	0.0015109	4.60E-13	0.67	0.000205	51.93875078
Height	Falls	rs4745948	10	A	G	0.021	-0.000139932	0.0029	0.00118317	2.70E-13	0.91	0.000207	52.45331325
Height	Falls	rs4748978	10	T	C	-0.018	-0.000924168	0.0031	0.00123953	4.30E-09	0.46	0.000134	33.87456635
Height	Falls	rs6584575	10	A	G	0.034	-0.00115785	0.0052	0.00200355	9.50E-11	0.56	0.000173	43.72812225
Height	Falls	rs6601882	10	C	T	-0.023	0.000445524	0.0038	0.00130951	6.40E-10	0.73	0.000163	41.23912343
Height	Falls	rs7069985	10	G	A	0.023	-0.00134993	0.0034	0.0014357	1.60E-11	0.35	0.000181	45.79487504
Height	Falls	rs7899004	10	C	T	-0.025	-0.00152922	0.0029	0.0011958	7.00E-17	0.2	0.000294	74.38942076
Height	Falls	rs10767838	11	G	A	-0.025	-0.00264397	0.0033	0.00134014	2.60E-14	0.0490004	0.000238	60.18754466
Height	Falls	rs11022753	11	C	T	0.018	0.000287781	0.0033	0.00133644	1.20E-07	0.83	0.000118	29.82908665
Height	Falls	rs11221442	11	C	G	-0.027	-0.000940676	0.0035	0.00136446	2.70E-14	0.49	0.000236	59.81128423
Height	Falls	rs1681630	11	C	T	-0.029	-0.000971961	0.0031	0.00125036	2.40E-20	0.44	0.000346	87.55137578
Height	Falls	rs17473243	11	A	G	0.023	-0.00296465	0.0031	0.00127939	9.40E-14	0.02	0.000217	55.05421646

Height	Falls	rs1870761	11	A	C	0.018	0.00261191	0.003	0.00120859	1.30E-09	0.0309999	0.000142	36.04511225
Height	Falls	rs2237886	11	T	C	0.043	-0.0031642	0.0049	0.00194018	5.30E-18	0.1	0.000307	77.78811775
Height	Falls	rs239256	11	T	C	0.028	0.00107746	0.0037	0.00142016	2.00E-14	0.450001	0.000228	57.72135214
Height	Falls	rs290195	11	G	A	0.016	-4.58E-05	0.0031	0.00123019	8.30E-08	0.97	0.000105	26.65744116
Height	Falls	rs3802758	11	A	G	0.039	-4.82E-05	0.0066	0.00217711	2.20E-09	0.98	0.000145	36.69513214
Height	Falls	rs4320932	11	C	T	-0.029	-0.0012142	0.0041	0.00153043	2.30E-12	0.43	0.000207	52.37831674
Height	Falls	rs4930585	11	C	T	0.028	-0.00241616	0.0041	0.00161045	4.40E-12	0.13	0.000184	46.65806336
Height	Falls	rs606452	11	C	A	-0.043	0.000566121	0.0043	0.00170284	1.90E-23	0.74	0.000399	101.1432656
Height	Falls	rs625735	11	A	G	0.023	-0.000625479	0.003	0.00120658	7.20E-15	0.6	0.000232	58.79263343
Height	Falls	rs717052	11	A	G	-0.025	-0.00173588	0.0044	0.00167424	6.40E-09	0.3	0.000133	33.64362105
Height	Falls	rs7925983	11	G	A	-0.017	-0.00109692	0.0033	0.00131243	3.40E-07	0.4	0.000105	26.55205079
Height	Falls	rs7935997	11	G	A	0.024	-0.000384863	0.0031	0.00121035	6.00E-15	0.75	0.000242	61.42837067
Height	Falls	rs7941132	11	G	T	0.042	0.000853999	0.0059	0.00243737	1.30E-12	0.73	0.000201	50.97921437
Height	Falls	rs1053051	12	C	T	-0.019	-0.00330229	0.0034	0.00118346	8.70E-09	0.00530005	0.000158	40.02484496
Height	Falls	rs10748128	12	T	G	0.038	0.000164885	0.0034	0.00124565	4.40E-29	0.89	0.000527	133.6370525
Height	Falls	rs10770705	12	C	A	-0.03	0.000861592	0.0031	0.00125104	2.30E-21	0.49	0.000372	94.14830316
Height	Falls	rs10843390	12	T	C	0.021	-0.000920441	0.0032	0.00132429	5.00E-11	0.49	0.00017	43.11969172
Height	Falls	rs10846654	12	A	G	-0.028	-0.00096912	0.0032	0.00123716	2.50E-19	0.43	0.000304	77.10436083
Height	Falls	rs10880969	12	C	T	0.024	0.000424557	0.0033	0.0012933	6.20E-13	0.74	0.000211	53.48722583
Height	Falls	rs11049545	12	A	C	-0.038	0.00139521	0.0032	0.00128345	2.40E-33	0.28	0.000557	141.0590645
Height	Falls	rs11104829	12	G	A	-0.018	0.000980218	0.0033	0.00131347	8.80E-08	0.46	0.000119	30.04553936
Height	Falls	rs11172372	12	C	T	-0.019	-0.00313229	0.003	0.00120227	7.60E-11	0.00920005	0.000158	40.13884378
Height	Falls	rs11172920	12	A	G	0.018	0.000686431	0.003	0.00120078	2.00E-09	0.57	0.000143	36.27699799
Height	Falls	rs11183167	12	G	A	-0.025	-0.00223495	0.0048	0.00176242	2.20E-07	0.2	0.000115	29.04927843
Height	Falls	rs11611927	12	G	A	0.051	-0.00100772	0.0035	0.00142586	2.60E-49	0.48	0.000838	212.3902596
Height	Falls	rs11612228	12	T	C	0.02	-0.000365223	0.0032	0.00125464	6.50E-10	0.77	0.000168	42.48186714

Height	Falls	rs12228415	12	G	A	0.015	-0.00345641	0.003	0.00119238	3.90E-07	0.00369999	9.88E-05	25.01708723
Height	Falls	rs12426318	12	A	C	-0.025	0.000634157	0.0044	0.00173778	3.20E-08	0.719999	0.000128	32.32606806
Height	Falls	rs12427384	12	G	A	-0.039	0.000330647	0.0038	0.00131647	3.40E-25	0.8	0.000424	107.4830383
Height	Falls	rs1353792	12	C	A	-0.018	0.00103889	0.0034	0.001385	3.00E-07	0.450001	0.000111	28.03454403
Height	Falls	rs17118431	12	G	A	0.047	-0.00343912	0.0058	0.00237773	1.10E-15	0.15	0.000265	67.03766803
Height	Falls	rs17192551	12	A	G	-0.023	-0.00300431	0.0041	0.00164895	2.80E-08	0.0680002	0.000126	31.81148457
Height	Falls	rs2358947	12	G	C	-0.028	-0.00168473	0.0044	0.00171441	2.10E-10	0.33	0.00017	43.05355699
Height	Falls	rs2454729	12	T	C	-0.018	-0.000220766	0.003	0.0012357	7.30E-09	0.86	0.000142	36.01763298
Height	Falls	rs2856321	12	A	G	-0.031	0.000892007	0.003	0.00123211	7.60E-24	0.47	0.000422	106.8621584
Height	Falls	rs4767473	12	A	G	0.025	0.00287413	0.0044	0.00177217	3.00E-08	0.1	0.000128	32.36150658
Height	Falls	rs7488294	12	G	A	-0.027	-0.000563325	0.0052	0.00187301	3.30E-07	0.760001	0.000112	28.47399725
Height	Falls	rs7971536	12	A	T	-0.029	0.000437582	0.0032	0.00118462	1.50E-19	0.709999	0.000346	87.55572548
Height	Falls	rs7980687	12	A	G	0.039	0.000621693	0.0037	0.00146699	1.00E-26	0.67	0.000439	111.285772
Height	Falls	rs8756	12	A	C	-0.059	0.00191485	0.0029	0.00118486	4.50E-90	0.11	0.001633	414.3700831
Height	Falls	rs11618507	13	T	G	0.023	-0.0013258	0.0036	0.00141651	3.40E-10	0.35	0.000163	41.31507977
Height	Falls	rs1199734	13	G	T	0.022	0.00157679	0.0039	0.0015676	1.60E-08	0.31	0.000132	33.43797865
Height	Falls	rs2010997	13	A	G	0.02	0.000234808	0.003	0.00119563	8.60E-12	0.84	0.000176	44.56902642
Height	Falls	rs2405414	13	A	G	0.016	-0.000991167	0.0029	0.00119045	1.10E-07	0.41	0.00012	30.46039679
Height	Falls	rs2706242	13	G	C	-0.026	0.000879756	0.0052	0.00203023	3.60E-07	0.66	0.000104	26.22692462
Height	Falls	rs3118905	13	A	G	-0.058	-0.000933756	0.0033	0.0013198	1.10E-69	0.48	0.001219	309.1428164
Height	Falls	rs3818416	13	C	A	0.021	0.00170812	0.0035	0.00139626	1.70E-09	0.22	0.000142	36.02318241
Height	Falls	rs675144	13	A	G	-0.023	-0.00116315	0.0043	0.00169318	1.40E-07	0.49	0.000116	29.27381315
Height	Falls	rs7319045	13	G	A	-0.024	-0.0022539	0.003	0.00122459	8.40E-15	0.0659994	0.000254	64.37232796
Height	Falls	rs7319446	13	A	G	-0.023	-0.00262759	0.0035	0.00140195	3.40E-11	0.061	0.000171	43.20909199
Height	Falls	rs9568328	13	T	A	0.051	0.00266105	0.0063	0.00195857	3.70E-16	0.17	0.000276	69.88720658
Height	Falls	rs9600340	13	A	G	-0.019	-0.000326609	0.003	0.0011999	1.80E-10	0.79	0.000158	40.14788499

Height	Falls	rs10131337	14	T	C	0.027	-0.000236137	0.0038	0.00138325	2.90E-12	0.86	0.000204	51.56955642
Height	Falls	rs10140922	14	T	G	-0.02	-0.00265911	0.003	0.0012042	5.10E-11	0.0269998	0.000177	44.73978219
Height	Falls	rs1190545	14	C	G	0.025	0.00127515	0.0033	0.00134321	4.10E-14	0.34	0.000227	57.42543094
Height	Falls	rs12882130	14	G	C	-0.025	0.00190781	0.0032	0.00123585	4.70E-15	0.12	0.000244	61.741826
Height	Falls	rs1370878	14	G	A	0.019	-0.000924782	0.0029	0.00119585	3.60E-10	0.44	0.00017	42.96189631
Height	Falls	rs17197086	14	T	C	0.035	-0.00281937	0.0048	0.00178641	3.40E-13	0.11	0.000222	56.20562718
Height	Falls	rs1950500	14	C	T	-0.031	0.000279829	0.0032	0.00130175	3.20E-22	0.83	0.000371	93.90808998
Height	Falls	rs2093210	14	T	C	-0.039	-0.000588919	0.0031	0.00121169	3.00E-35	0.630001	0.000633	160.4338268
Height	Falls	rs2215061	14	T	C	-0.017	0.00274514	0.003	0.0011876	5.90E-09	0.021	0.000127	32.1246821
Height	Falls	rs6571772	14	T	A	-0.018	0.000843928	0.0033	0.00129862	6.90E-08	0.52	0.00012	30.39908759
Height	Falls	rs7155279	14	T	G	-0.028	0.000653714	0.0031	0.00123088	4.20E-20	0.6	0.000322	81.62422421
Height	Falls	rs8003738	14	G	A	0.023	-5.51E-05	0.0036	0.00149473	1.80E-10	0.97	0.000161	40.82805642
Height	Falls	rs8017130	14	G	A	0.023	0.00166547	0.0034	0.00128383	1.00E-11	0.19	0.000197	49.97319487
Height	Falls	rs811153	14	T	C	0.02	-0.000157464	0.0037	0.0015087	8.90E-08	0.92	0.000116	29.26335064
Height	Falls	rs862034	14	G	A	0.028	0.000730696	0.003	0.00123481	6.40E-20	0.55	0.000347	87.83239608
Height	Falls	rs927381	14	T	C	0.022	-0.00103662	0.003	0.00119535	4.10E-13	0.39	0.000213	54.01084972
Height	Falls	rs10152739	15	T	A	0.022	-0.00285359	0.0034	0.00138176	1.30E-10	0.0389996	0.000165	41.91667013
Height	Falls	rs11636119	15	C	A	0.022	0.00218172	0.0041	0.00160728	5.40E-08	0.17	0.000115	29.10702999
Height	Falls	rs11855014	15	A	G	-0.022	0.000781094	0.0034	0.00128031	9.20E-11	0.54	0.000167	42.34751861
Height	Falls	rs12900132	15	T	C	0.031	8.45E-05	0.0033	0.00122681	2.90E-20	0.95	0.000356	90.12920075
Height	Falls	rs12908753	15	G	A	-0.038	0.00275162	0.0036	0.00132711	5.70E-26	0.0379997	0.000447	113.3321429
Height	Falls	rs16942383	15	A	C	-0.12	0.0023985	0.0089	0.00290267	7.60E-41	0.41	0.000772	195.599127
Height	Falls	rs16964211	15	A	G	-0.057	-0.00307855	0.0071	0.00281396	1.20E-15	0.27	0.000267	67.73831122
Height	Falls	rs2306578	15	C	T	0.069	0.00601596	0.0097	0.00423299	8.00E-13	0.16	0.000217	54.93572782
Height	Falls	rs316618	15	A	T	-0.026	0.00396095	0.0037	0.00144438	3.30E-12	0.0061	0.000197	49.81057525
Height	Falls	rs4246302	15	G	A	0.027	0.000207644	0.0033	0.00128936	1.70E-16	0.87	0.000273	69.18074622

Height	Falls	rs4461027	15	T	C	0.028	0.000142748	0.0031	0.00120357	7.50E-20	0.91	0.000327	82.86148057
Height	Falls	rs4548838	15	C	T	-0.033	-0.001207	0.003	0.00119045	8.60E-28	0.31	0.000492	124.7150699
Height	Falls	rs5742915	15	C	T	0.035	0.00336127	0.0031	0.00118664	1.50E-29	0.00460002	0.000508	128.8309743
Height	Falls	rs7162542	15	G	C	0.046	0.00252218	0.0029	0.00119011	8.20E-55	0.0340001	0.000996	252.4324348
Height	Falls	rs7162825	15	T	C	0.016	0.00020193	0.0029	0.00118269	3.60E-08	0.86	0.00012	30.45377943
Height	Falls	rs7178424	15	T	C	-0.021	-0.000529074	0.003	0.00118808	5.00E-13	0.66	0.000195	49.29504707
Height	Falls	rs11642612	16	C	A	0.016	0.000469531	0.003	0.00120591	4.20E-08	0.7	0.000112	28.45331905
Height	Falls	rs11648796	16	G	A	0.033	0.00212014	0.0038	0.00146973	1.40E-18	0.15	0.000327	82.77121849
Height	Falls	rs12924101	16	C	A	-0.022	-0.000408307	0.0043	0.00151853	2.70E-07	0.79	0.000104	26.44062802
Height	Falls	rs1659127	16	A	G	0.03	0.000170535	0.0033	0.00125881	2.80E-19	0.89	0.00033	83.50144711
Height	Falls	rs2023693	16	G	A	0.017	-0.00129749	0.003	0.00120037	2.40E-08	0.28	0.000127	32.11986118
Height	Falls	rs2120692	16	A	G	-0.027	0.00390576	0.0051	0.00197991	1.70E-07	0.0490004	0.000112	28.25165505
Height	Falls	rs213653	16	G	A	-0.023	-0.00180281	0.0042	0.00131552	3.40E-08	0.17	0.000139	35.27637134
Height	Falls	rs217181	16	T	C	0.024	-0.00323744	0.0038	0.00149899	3.70E-10	0.0309999	0.000158	39.9828055
Height	Falls	rs2303262	16	T	C	-0.025	-0.000715465	0.0041	0.00140799	1.50E-09	0.61	0.000189	47.80590167
Height	Falls	rs2326458	16	A	C	-0.022	0.000518129	0.0035	0.00135676	5.00E-10	0.7	0.000157	39.77465025
Height	Falls	rs26868	16	A	T	0.029	0.00330766	0.0033	0.00118945	3.20E-18	0.00539995	0.000334	84.50140922
Height	Falls	rs3790086	16	G	C	-0.023	0.00380729	0.0029	0.00118951	3.00E-15	0.00140001	0.000248	62.91745428
Height	Falls	rs3868143	16	C	T	-0.033	-0.00503395	0.0055	0.00225494	2.60E-09	0.0259998	0.000143	36.15315626
Height	Falls	rs8052560	16	A	C	0.036	0.000926777	0.0044	0.00139814	3.80E-16	0.51	0.000281	71.22517524
Height	Falls	rs8058684	16	A	G	0.021	-0.00210865	0.0032	0.00128747	1.20E-10	0.1	0.00017	43.15073627
Height	Falls	rs960006	16	C	T	0.02	-0.00155332	0.0033	0.00118784	1.00E-09	0.19	0.000186	47.04168652
Height	Falls	rs9929889	16	T	C	-0.017	-4.15E-05	0.0032	0.00120736	7.60E-08	0.97	0.000114	28.90673257
Height	Falls	rs11652094	17	C	G	0.018	-0.00183834	0.0032	0.00128452	9.30E-09	0.15	0.000126	31.82179184
Height	Falls	rs1401796	17	A	C	-0.031	-0.000421483	0.003	0.00119128	2.30E-24	0.719999	0.000435	110.3467087
Height	Falls	rs1552173	17	T	C	-0.018	-0.00204251	0.0029	0.00119045	7.90E-10	0.0860003	0.000153	38.75707962

Height	Falls	rs16967866	17	G	T	-0.038	0.0020164	0.0066	0.00277791	6.50E-09	0.47	0.000131	33.16408149
Height	Falls	rs180165	17	C	T	0.019	0.00140131	0.003	0.00119682	6.40E-10	0.24	0.00016	40.47191106
Height	Falls	rs2028067	17	C	T	0.031	-0.00376484	0.0039	0.00161706	8.20E-16	0.02	0.000251	63.67637153
Height	Falls	rs2070776	17	G	A	0.042	-0.000670361	0.0031	0.00123955	6.00E-41	0.59	0.00073	185.016803
Height	Falls	rs2072268	17	A	G	-0.02	-0.000223917	0.0031	0.00118458	1.10E-10	0.85	0.000174	43.99441424
Height	Falls	rs2079795	17	C	T	-0.045	-0.000876676	0.0031	0.00124819	1.70E-46	0.48	0.000832	210.832872
Height	Falls	rs227723	17	T	C	0.03	0.000679025	0.0032	0.00128429	8.00E-21	0.6	0.000349	88.35141724
Height	Falls	rs3764419	17	A	C	-0.041	-0.000275491	0.003	0.00121828	2.70E-41	0.82	0.000738	186.9815273
Height	Falls	rs423887	17	C	T	0.015	-0.000739166	0.0029	0.00118403	2.20E-07	0.53	0.000106	26.84235479
Height	Falls	rs4239020	17	T	C	-0.021	-0.000509614	0.0031	0.00125698	8.80E-12	0.69	0.000181	45.90927374
Height	Falls	rs4969440	17	C	T	0.023	-0.000236059	0.0043	0.00130657	1.20E-07	0.86	0.000131	33.23729281
Height	Falls	rs4986172	17	T	C	-0.034	0.000221219	0.0032	0.0012474	7.60E-27	0.86	0.000471	119.337964
Height	Falls	rs584828	17	T	C	-0.025	0.000614999	0.003	0.00120968	3.50E-17	0.61	0.000275	69.78387788
Height	Falls	rs8067165	17	G	C	0.023	0.000670252	0.0033	0.00121906	7.30E-12	0.58	0.000208	52.61605928
Height	Falls	rs9217	17	C	T	0.028	-0.00172601	0.003	0.00123313	4.60E-20	0.16	0.000344	87.24165361
Height	Falls	rs9893629	17	C	T	-0.02	-0.000292762	0.0036	0.00125382	1.00E-08	0.82	0.000127	32.27838232
Height	Falls	rs999474	17	A	G	0.022	-0.00162868	0.003	0.00119217	1.10E-13	0.17	0.000212	53.80879443
Height	Falls	rs11152213	18	C	A	0.025	0.00207838	0.0035	0.00139219	6.90E-13	0.14	0.000202	51.06577111
Height	Falls	rs3017036	18	T	G	-0.021	-0.00158463	0.0036	0.00130616	4.20E-09	0.23	0.000172	43.48362935
Height	Falls	rs4369779	18	C	T	0.056	0.00368374	0.0036	0.00144802	1.50E-53	0.0109999	0.000955	242.0670565
Height	Falls	rs4939837	18	G	A	-0.019	-0.00097614	0.0033	0.00124905	1.10E-08	0.43	0.000134	33.97749831
Height	Falls	rs498685	18	C	T	-0.016	0.000986621	0.0029	0.00120284	7.80E-08	0.41	0.00012	30.44836737
Height	Falls	rs8090312	18	G	A	-0.024	0.00052991	0.0034	0.00127683	3.30E-13	0.68	0.000199	50.4821175
Height	Falls	rs8097893	18	G	A	-0.042	-0.00115666	0.0068	0.00294271	4.60E-10	0.69	0.000151	38.26602571
Height	Falls	rs888403	18	G	A	0.019	0.000181825	0.0033	0.00124063	9.30E-09	0.88	0.000133	33.72501956
Height	Falls	rs9967417	18	C	G	-0.04	0.00126926	0.003	0.00119298	2.20E-40	0.29	0.000707	179.2248169

Height	Falls	rs11667331	19	G	A	0.024	-0.00441127	0.004	0.00160093	1.30E-09	0.00589997	0.000142	36.0102364
Height	Falls	rs11880124	19	G	A	-0.041	0.000778245	0.0054	0.00212473	2.20E-14	0.709999	0.000229	58.10003912
Height	Falls	rs11880992	19	A	G	0.033	0.00129333	0.003	0.00120355	6.90E-28	0.28	0.000481	121.995088
Height	Falls	rs13306455	19	T	C	0.031	0.00315514	0.0039	0.00157199	2.10E-15	0.0449997	0.000252	63.86900525
Height	Falls	rs2279007	19	A	G	-0.025	0.00139459	0.0036	0.00136398	7.20E-12	0.31	0.000195	49.36707052
Height	Falls	rs4542783	19	C	T	-0.032	-0.0016622	0.0039	0.00119362	1.20E-15	0.16	0.000396	100.3428228
Height	Falls	rs4803468	19	G	A	-0.03	0.000987901	0.0031	0.00121249	1.70E-21	0.42	0.000383	96.99201962
Height	Falls	rs4806934	19	G	A	0.029	0.00137268	0.0031	0.00123743	1.30E-20	0.27	0.000355	89.82707787
Height	Falls	rs7256192	19	A	G	-0.017	0.00104672	0.0031	0.00123641	5.70E-08	0.4	0.000121	30.72919344
Height	Falls	rs8103992	19	C	A	-0.029	-0.00055745	0.0037	0.00148158	1.20E-14	0.709999	0.000243	61.48194858
Height	Falls	rs8108722	19	C	T	0.02	-0.00038659	0.0038	0.00150212	1.20E-07	0.8	0.000111	28.22831624
Height	Falls	rs143384	20	G	A	0.075	0.000522578	0.0032	0.00120406	1.10E-121	0.66	0.002212	561.5138801
Height	Falls	rs1884897	20	G	A	-0.044	0.00104623	0.003	0.00122791	1.30E-48	0.39	0.000849	215.2862055
Height	Falls	rs2057291	20	G	A	-0.02	-0.00119511	0.0032	0.00124441	2.20E-10	0.34	0.000157	39.73024979
Height	Falls	rs237711	20	G	C	0.037	0.00190262	0.0036	0.00143044	3.00E-25	0.18	0.000417	105.6915527
Height	Falls	rs4239726	20	G	C	-0.025	-0.00136962	0.005	0.00204989	4.40E-07	0.5	0.0001	25.35588293
Height	Falls	rs4812888	20	A	G	0.021	0.003295	0.0037	0.00150445	1.40E-08	0.0290001	0.000127	32.24448401
Height	Falls	rs6012799	20	T	C	-0.021	0.00245484	0.0035	0.00141192	2.80E-09	0.0819993	0.000142	36.0499575
Height	Falls	rs6047319	20	A	G	-0.019	-0.00151161	0.0032	0.00126608	1.20E-09	0.23	0.00014	35.37990086
Height	Falls	rs6085662	20	C	G	0.02	0.00264347	0.0032	0.00123373	3.00E-10	0.032	0.000156	39.55758278
Height	Falls	rs7261425	20	G	C	-0.021	-0.000813716	0.0034	0.00132163	1.80E-10	0.54	0.000151	38.28012437
Height	Falls	rs7273787	20	G	A	0.022	0.000225963	0.0031	0.00124588	2.80E-12	0.86	0.0002	50.5528145
Height	Falls	rs7274811	20	T	G	-0.045	0.00115315	0.0035	0.00135036	5.50E-38	0.39	0.000655	165.9620133
Height	Falls	rs913000	20	C	T	-0.024	-0.000405177	0.0033	0.0013129	5.50E-13	0.760001	0.000219	55.60559775
Height	Falls	rs2829964	21	G	A	0.016	0.000900949	0.0029	0.00118462	7.90E-08	0.450001	0.00012	30.46340563
Height	Falls	rs2834442	21	A	T	0.024	-0.00163176	0.0031	0.00123356	4.40E-15	0.19	0.000237	59.99204167

Height	Falls	rs2836288	21	A	C	-0.019	0.00327906	0.0029	0.00119091	3.80E-11	0.00589997	0.00017	42.99723618
Height	Falls	rs9977276	21	G	T	0.022	-0.00151976	0.0035	0.00141595	2.90E-10	0.28	0.000156	39.57488987
Height	Falls	rs4821890	22	A	G	0.017	-0.000522178	0.0031	0.00126954	4.20E-08	0.68	0.000119	30.08281748
Height	Falls	rs5754185	22	G	C	-0.035	-0.000722749	0.0042	0.00166776	4.50E-17	0.66	0.000274	69.45952729
Height	Falls	rs5754217	22	T	G	-0.019	-0.00101202	0.0036	0.00149939	8.50E-08	0.5	0.00011	27.86296871
Height	Falls	rs5757318	22	T	A	0.029	-0.00124029	0.0048	0.00163949	1.90E-09	0.450001	0.000147	37.26652935
Height	Falls	rs9614670	22	T	C	0.02	-0.000886176	0.0038	0.00147932	8.00E-08	0.55	0.000112	28.41201189
Sitting height	Falls	rs1044299	1	T	C	0.0197906	-0.000109766	0.00189041	0.00119152	1.21E-25	0.93	0.000326	109.598828
Sitting height	Falls	rs10911335	1	C	T	0.0342755	0.00157962	0.00230015	0.00145005	3.35E-50	0.28	0.00066	222.0523023
Sitting height	Falls	rs11205303	1	C	T	0.0209368	0.000313916	0.00191074	0.00120535	6.19E-28	0.79	0.000357	120.0652745
Sitting height	Falls	rs11576222	1	A	G	0.0171192	-0.000239148	0.00252508	0.00159142	1.21E-11	0.88	0.000137	45.96387632
Sitting height	Falls	rs11578046	1	A	G	-0.0103119	0.000360543	0.00200465	0.00126192	2.69E-07	0.780001	7.87E-05	26.46063541
Sitting height	Falls	rs11588850	1	G	A	-0.0281147	0.00184859	0.00249611	0.00158043	2.01E-29	0.24	0.000377	126.8643116
Sitting height	Falls	rs12082939	1	T	C	0.0126969	0.000283933	0.00192506	0.00121453	4.24E-11	0.82	0.000129	43.50176301
Sitting height	Falls	rs12089041	1	T	C	0.0255335	0.00315719	0.00328851	0.00206999	8.22E-15	0.13	0.000179	60.28681853
Sitting height	Falls	rs12096239	1	C	G	-0.0216335	0.00102778	0.00215912	0.00135816	1.26E-23	0.450001	0.000299	100.3922101
Sitting height	Falls	rs12127605	1	T	C	-0.0154685	-0.000916546	0.00270584	0.00170644	1.09E-08	0.59	9.72E-05	32.6807623
Sitting height	Falls	rs12130046	1	A	G	-0.0140808	-0.00233943	0.00264246	0.00167065	9.90E-08	0.16	8.45E-05	28.39473474
Sitting height	Falls	rs12134270	1	G	T	0.0165673	-0.00163414	0.00225596	0.00142643	2.08E-13	0.25	0.00016	53.93127443
Sitting height	Falls	rs12730973	1	G	A	0.016667	0.000408101	0.00188864	0.00118965	1.10E-18	0.73	0.000232	77.87830737
Sitting height	Falls	rs1327124	1	G	A	0.0244777	-0.000812772	0.00197334	0.00124647	2.52E-35	0.51	0.000457	153.864127
Sitting height	Falls	rs1360504	1	A	G	0.0170258	0.000102705	0.00238308	0.0014998	9.05E-13	0.95	0.000152	51.04319035
Sitting height	Falls	rs1679937	1	T	G	0.0117891	0.002726	0.00228242	0.00143624	2.40E-07	0.0580003	7.94E-05	26.67903856
Sitting height	Falls	rs17369123	1	T	C	0.0215085	-0.000449027	0.00239668	0.00151619	2.87E-19	0.77	0.00024	80.53787084
Sitting height	Falls	rs17465651	1	T	C	-0.0169104	-0.00298216	0.00188397	0.00118727	2.82E-19	0.012	0.00024	80.56748286
Sitting height	Falls	rs1768809	1	C	A	-0.0140011	0.00140191	0.0018946	0.00119449	1.47E-13	0.24	0.000162	54.61214681

Sitting height	Falls	rs1926869	1	T	C	-0.0114718	-0.00021273	0.00203998	0.00128551	1.87E-08	0.87	9.41E-05	31.62360047
Sitting height	Falls	rs1985278	1	T	G	-0.010841	-0.000161452	0.00194217	0.00122422	2.38E-08	0.9	9.27E-05	31.15761495
Sitting height	Falls	rs2001744	1	G	A	0.0106331	0.000849294	0.00195055	0.00123	5.00E-08	0.49	8.84E-05	29.71704499
Sitting height	Falls	rs2501256	1	T	C	-0.0271934	0.00213033	0.002003	0.00126378	5.67E-42	0.0920005	0.000548	184.3168855
Sitting height	Falls	rs2570972	1	G	A	-0.00958156	-0.00121444	0.00189221	0.00119137	4.11E-07	0.31	7.63E-05	25.64093043
Sitting height	Falls	rs2782639	1	G	A	-0.0135671	0.00134441	0.00207207	0.00130384	5.85E-11	0.3	0.000128	42.8711572
Sitting height	Falls	rs2798631	1	G	A	0.0160652	-0.000160131	0.00189096	0.00119109	1.97E-17	0.89	0.000215	72.17845619
Sitting height	Falls	rs3157	1	G	A	0.0245415	0.00211876	0.00188278	0.00118685	7.94E-39	0.0739997	0.000505	169.9037853
Sitting height	Falls	rs34517439	1	A	C	0.0285343	0.0029921	0.00287775	0.00183027	3.59E-23	0.1	0.000292	98.31693265
Sitting height	Falls	rs35485741	1	C	G	-0.0182627	-0.00305431	0.00298032	0.00186395	8.92E-10	0.1	0.000112	37.54950149
Sitting height	Falls	rs3738001	1	G	A	0.0241863	0.00049919	0.00362695	0.00228085	2.59E-11	0.83	0.000132	44.46883314
Sitting height	Falls	rs4233334	1	T	A	0.0159522	0.000127702	0.0018805	0.00118546	2.20E-17	0.91	0.000214	71.9605537
Sitting height	Falls	rs6675441	1	A	G	-0.0146827	-0.000826173	0.00225661	0.00141926	7.70E-11	0.56	0.000126	42.33492882
Sitting height	Falls	rs7515675	1	G	A	-0.014403	-0.00123767	0.001964	0.0012374	2.25E-13	0.32	0.00016	53.78026706
Sitting height	Falls	rs7520808	1	G	C	0.0228958	-0.000176267	0.00218796	0.00137579	1.27E-25	0.9	0.000326	109.5047327
Sitting height	Falls	rs7540825	1	C	G	0.0137404	-0.0012005	0.00194731	0.00122787	1.72E-12	0.33	0.000148	49.78844509
Sitting height	Falls	rs79429162	1	A	G	-0.017813	-0.00331358	0.00333494	0.00209789	9.23E-08	0.11	8.49E-05	28.5297579
Sitting height	Falls	rs823121	1	A	G	-0.0186202	-0.004161	0.00189632	0.00119597	9.38E-23	5.00E-04	0.000287	96.41518345
Sitting height	Falls	rs9428100	1	C	T	0.0148784	0.000766283	0.00227358	0.00143099	6.00E-11	0.59	0.000127	42.82446837
Sitting height	Falls	rs9435731	1	A	C	0.021257	2.36E-05	0.00187819	0.00118363	1.08E-29	0.98	0.000381	128.0928526
Sitting height	Falls	rs946602	1	C	T	0.0142126	0.00189537	0.00192822	0.00121365	1.70E-13	0.12	0.000162	54.32927386
Sitting height	Falls	rs10165255	2	G	A	-0.0143973	0.00260309	0.00189784	0.00119551	3.30E-14	0.0290001	0.000171	57.54968049
Sitting height	Falls	rs10200995	2	A	T	-0.0153445	0.000546356	0.00273145	0.0017127	1.94E-08	0.75	9.39E-05	31.55869041
Sitting height	Falls	rs1153122	2	C	T	0.0173506	-0.00462029	0.00205234	0.00129197	2.82E-17	0.000350002	0.000213	71.47108517
Sitting height	Falls	rs11676298	2	G	C	-0.0154922	0.00173444	0.00238113	0.00150391	7.72E-11	0.25	0.000126	42.33114117
Sitting height	Falls	rs11694153	2	C	G	-0.0196604	0.00190258	0.00306346	0.00192353	1.39E-10	0.32	0.000123	41.18701054

Sitting height	Falls	rs12470285	2	T	C	-0.0256497	0.00213954	0.0024987	0.00157745	1.02E-24	0.17	0.000313	105.3746988
Sitting height	Falls	rs12471018	2	G	A	0.0125314	0.00170629	0.00217998	0.00137465	9.02E-09	0.21	9.83E-05	33.04411821
Sitting height	Falls	rs12477783	2	A	G	0.025598	0.000891292	0.00361642	0.00229582	1.46E-12	0.7	0.000149	50.10191718
Sitting height	Falls	rs12693968	2	A	G	-0.0130032	0.000832536	0.00215364	0.00135567	1.56E-09	0.54	0.000108	36.45476199
Sitting height	Falls	rs12694416	2	C	A	0.0250929	0.000576983	0.00190819	0.00120327	1.74E-39	0.630001	0.000514	172.9252772
Sitting height	Falls	rs12987656	2	T	C	0.0128243	-0.00133791	0.00226178	0.00142238	1.43E-08	0.35	9.56E-05	32.1489371
Sitting height	Falls	rs13007688	2	T	C	0.011974	-0.001586	0.00194622	0.0012258	7.64E-10	0.2	0.000113	37.85250672
Sitting height	Falls	rs1569135	2	G	A	0.0123712	0.000705826	0.00188692	0.00118778	5.52E-11	0.55	0.000128	42.98497521
Sitting height	Falls	rs17400325	2	C	T	0.0484646	-0.00265132	0.00472672	0.00296186	1.15E-24	0.37	0.000313	105.1306894
Sitting height	Falls	rs17743415	2	C	T	-0.022046	-0.00133566	0.00189648	0.00119516	3.13E-31	0.26	0.000402	135.1335146
Sitting height	Falls	rs1805165	2	A	C	-0.0192546	0.000160575	0.00208339	0.00131574	2.43E-20	0.9	0.000254	85.41376214
Sitting height	Falls	rs183743021	2	C	T	-0.0466598	0.00430525	0.00628458	0.00397014	1.13E-13	0.28	0.000164	55.12304854
Sitting height	Falls	rs2028106	2	G	C	-0.038661	-0.00105878	0.00523235	0.00330553	1.48E-13	0.75	0.000162	54.59496509
Sitting height	Falls	rs2140046	2	C	T	-0.017029	-0.00100228	0.00195141	0.00123023	2.64E-18	0.42	0.000226	76.15198681
Sitting height	Falls	rs2309725	2	A	C	0.0122994	-0.000703541	0.00189073	0.0011915	7.77E-11	0.55	0.000126	42.31641049
Sitting height	Falls	rs3815305	2	A	G	0.01939	0.0028485	0.00333442	0.00209287	6.06E-09	0.17	0.000101	33.81543774
Sitting height	Falls	rs4665972	2	C	T	0.019001	-0.00192116	0.00193211	0.00121548	8.06E-23	0.11	0.000288	96.71397187
Sitting height	Falls	rs4671965	2	C	A	-0.0114696	-0.00116249	0.00199414	0.00125828	8.84E-09	0.36	9.84E-05	33.08150466
Sitting height	Falls	rs500422	2	A	C	0.0149428	-0.000703649	0.00189513	0.00119362	3.16E-15	0.56	0.000185	62.17072821
Sitting height	Falls	rs56200610	2	T	C	0.0219922	0.000808644	0.00210407	0.00132823	1.44E-25	0.54	0.000325	109.2488777
Sitting height	Falls	rs56372408	2	G	A	-0.0205913	-0.000234783	0.0021788	0.00137891	3.38E-21	0.86	0.000266	89.31672675
Sitting height	Falls	rs59985551	2	T	C	-0.0312003	-0.00444007	0.00224856	0.00141279	9.15E-44	0.0017	0.000572	192.5345075
Sitting height	Falls	rs60695939	2	C	T	-0.0473687	-0.00299358	0.00607283	0.00374624	6.20E-15	0.42	0.000181	60.84160809
Sitting height	Falls	rs6546564	2	T	C	-0.0100776	0.000233392	0.0019439	0.00122566	2.17E-07	0.85	7.99E-05	26.87610896
Sitting height	Falls	rs66989638	2	A	G	0.0186779	0.000440557	0.00291909	0.00184065	1.57E-10	0.81	0.000122	40.94125219
Sitting height	Falls	rs6714546	2	G	A	0.0236083	-0.000742988	0.00207088	0.00130607	4.23E-30	0.57	0.000386	129.9629506

Sitting height	Falls	rs6728237	2	T	C	0.0120612	0.00158778	0.00190785	0.00120244	2.59E-10	0.19	0.000119	39.96617497
Sitting height	Falls	rs6754426	2	A	G	-0.0156361	0.00110954	0.00192725	0.00121357	4.95E-16	0.36	0.000196	65.8234695
Sitting height	Falls	rs702938	2	G	A	0.0143693	-5.67E-05	0.0019764	0.00124385	3.59E-13	0.96	0.000157	52.85931533
Sitting height	Falls	rs734586	2	C	T	0.0125474	-0.000155788	0.00188492	0.00118791	2.80E-11	0.9	0.000132	44.31202949
Sitting height	Falls	rs7565457	2	T	C	0.0111493	-0.00138016	0.00188305	0.0011869	3.21E-09	0.24	0.000104	35.05673769
Sitting height	Falls	rs7593987	2	G	A	-0.0154984	4.54E-05	0.00238748	0.00150583	8.51E-11	0.98	0.000125	42.13997238
Sitting height	Falls	rs7598861	2	A	G	-0.0159253	-0.00117213	0.00249774	0.00157314	1.82E-10	0.46	0.000121	40.65189422
Sitting height	Falls	rs77735628	2	A	C	-0.0263891	0.000750952	0.00344302	0.00216168	1.80E-14	0.73	0.000175	58.7448862
Sitting height	Falls	rs78198962	2	T	C	0.0556434	-0.0041261	0.00534263	0.0033419	2.14E-25	0.22	0.000323	108.4718685
Sitting height	Falls	rs78905646	2	A	G	0.0446727	-0.0022785	0.00604446	0.00384077	1.46E-13	0.55	0.000162	54.62222512
Sitting height	Falls	rs848538	2	C	G	-0.0132153	0.00109878	0.00208109	0.00131116	2.15E-10	0.4	0.00012	40.32480986
Sitting height	Falls	rs9636434	2	G	A	0.0313047	0.000445925	0.00190535	0.00120193	1.23E-60	0.709999	0.000802	269.9414422
Sitting height	Falls	rs9989781	2	A	G	0.0264151	-0.00105959	0.005007	0.00314177	1.32E-07	0.74	8.28E-05	27.83231529
Sitting height	Falls	rs11130440	3	T	C	0.0110567	0.000933582	0.00192394	0.00121196	9.10E-09	0.44	9.82E-05	33.02691466
Sitting height	Falls	rs11707955	3	C	T	0.0148095	0.000640678	0.00189293	0.00119285	5.15E-15	0.59	0.000182	61.20849255
Sitting height	Falls	rs11708067	3	G	A	-0.0169391	0.00157593	0.00217989	0.00137951	7.83E-15	0.25	0.00018	60.38255777
Sitting height	Falls	rs11719737	3	C	T	0.0170889	-0.000538445	0.00277382	0.00174654	7.25E-10	0.760001	0.000113	37.9552334
Sitting height	Falls	rs12636560	3	G	C	-0.0130772	0.00334298	0.0018822	0.00118673	3.72E-12	0.00479999	0.000144	48.27230017
Sitting height	Falls	rs13085781	3	T	A	0.00995696	-0.00163577	0.00197417	0.00124312	4.57E-07	0.19	7.57E-05	25.43808587
Sitting height	Falls	rs146370649	3	G	C	0.0616504	-0.00443061	0.00624605	0.00399127	5.64E-23	0.27	0.00029	97.42286218
Sitting height	Falls	rs1823217	3	G	A	-0.0109321	0.00018141	0.00196951	0.00124043	2.85E-08	0.88	9.16E-05	30.80993704
Sitting height	Falls	rs1895030	3	C	T	-0.011578	-0.00189828	0.00188697	0.00118926	8.49E-10	0.11	0.000112	37.64758299
Sitting height	Falls	rs1910466	3	C	T	-0.0195689	-0.000214098	0.00188904	0.00119017	3.84E-25	0.86	0.000319	107.3125512
Sitting height	Falls	rs2194411	3	A	G	0.0414841	-0.00135168	0.00284252	0.00178958	3.17E-48	0.450001	0.000633	212.9885647
Sitting height	Falls	rs2270894	3	G	C	-0.0271965	0.00191666	0.00241471	0.00152642	2.03E-29	0.21	0.000377	126.851636
Sitting height	Falls	rs2346020	3	A	G	0.0111963	-0.00065784	0.00200596	0.00126271	2.39E-08	0.6	9.27E-05	31.1533329

Sitting height	Falls	rs2871960	3	C	A	0.0469322	-0.000664292	0.00188566	0.00118964	1.30E-136	0.58	0.001839	619.462539
Sitting height	Falls	rs300982	3	A	G	-0.0227654	0.00366775	0.00433626	0.00272171	1.52E-07	0.18	8.20E-05	27.56258475
Sitting height	Falls	rs343422	3	C	G	0.010342	-0.0032361	0.0020102	0.00126669	2.68E-07	0.0109999	7.87E-05	26.46857311
Sitting height	Falls	rs34390533	3	A	C	-0.0111319	0.00244793	0.00217847	0.00137568	3.22E-07	0.0749998	7.77E-05	26.11171672
Sitting height	Falls	rs34693680	3	T	C	0.0172914	-0.000193468	0.00275723	0.00174606	3.58E-10	0.91	0.000117	39.32912864
Sitting height	Falls	rs4073154	3	G	A	0.0248511	-0.00114092	0.00226795	0.00143026	6.18E-28	0.43	0.000357	120.0671722
Sitting height	Falls	rs4076108	3	T	A	0.0138313	-0.00382674	0.00218816	0.00138068	2.60E-10	0.00560003	0.000119	39.95469805
Sitting height	Falls	rs4331690	3	C	T	0.011919	0.00294584	0.00201676	0.00126997	3.42E-09	0.02	0.000104	34.92779758
Sitting height	Falls	rs4681299	3	A	G	0.0152995	0.000483758	0.0018895	0.00119075	5.65E-16	0.68	0.000195	65.56328167
Sitting height	Falls	rs4974539	3	A	G	-0.0104212	0.00116425	0.0019266	0.0012153	6.34E-08	0.34	8.70E-05	29.2585197
Sitting height	Falls	rs519384	3	A	T	0.0239181	-0.00148158	0.00207784	0.00131405	1.18E-30	0.26	0.000394	132.504049
Sitting height	Falls	rs61732778	3	A	G	0.03253	0.00386762	0.00364624	0.00229945	4.62E-19	0.0929994	0.000237	79.59350401
Sitting height	Falls	rs6770544	3	T	G	-0.0209386	-0.000986451	0.00199056	0.00125651	7.13E-26	0.43	0.000329	110.6482973
Sitting height	Falls	rs6810039	3	A	C	-0.0139435	0.00093051	0.00191575	0.0012077	3.39E-13	0.44	0.000158	52.97438623
Sitting height	Falls	rs73081811	3	G	T	0.0126088	0.000990611	0.00188651	0.00118765	2.33E-11	0.4	0.000133	44.67137174
Sitting height	Falls	rs75495843	3	A	G	0.0597306	0.00488374	0.00548545	0.00345352	1.32E-27	0.16	0.000353	118.5684747
Sitting height	Falls	rs7612882	3	A	G	0.00984311	-0.00151515	0.00188773	0.00119074	1.85E-07	0.2	8.09E-05	27.18847956
Sitting height	Falls	rs7633840	3	C	T	0.0130841	0.000342928	0.00203563	0.00128281	1.30E-10	0.79	0.000123	41.31331308
Sitting height	Falls	rs7645611	3	C	G	0.0263812	-0.00143853	0.00309456	0.00195196	1.53E-17	0.46	0.000216	72.67604374
Sitting height	Falls	rs7652177	3	G	C	0.0191747	-0.000705596	0.00187636	0.0011836	1.64E-24	0.55	0.000311	104.4298907
Sitting height	Falls	rs900400	3	C	T	0.0143602	-0.00223051	0.00191816	0.00120647	7.10E-14	0.064	0.000167	56.04686388
Sitting height	Falls	rs9809116	3	G	A	-0.0181743	-0.000237479	0.00192499	0.00121244	3.71E-21	0.84	0.000265	89.13708545
Sitting height	Falls	rs985344	3	A	G	-0.02479	-0.00196518	0.00312732	0.00195061	2.25E-15	0.31	0.000187	62.83598234
Sitting height	Falls	rs9860524	3	T	C	0.00964658	-0.000179282	0.00188105	0.00118496	2.93E-07	0.88	7.82E-05	26.29941337
Sitting height	Falls	rs113518534	4	C	T	0.017399	0.00106564	0.00207466	0.0013072	5.03E-17	0.41	0.000209	70.33228201
Sitting height	Falls	rs11731421	4	A	G	0.0151129	0.00197489	0.00199353	0.00125733	3.44E-14	0.12	0.000171	57.47117365

Sitting height	Falls	rs12506356	4	A	G	0.0109009	-5.76E-05	0.00208983	0.00131564	1.83E-07	0.97	8.09E-05	27.20838678
Sitting height	Falls	rs13103161	4	A	T	-0.0264665	-0.00330526	0.00192664	0.00121659	6.24E-43	0.00659994	0.000561	188.7086816
Sitting height	Falls	rs13115570	4	G	T	0.0150343	0.0029582	0.0020389	0.0012802	1.66E-13	0.021	0.000162	54.37190788
Sitting height	Falls	rs13125694	4	T	C	0.0415072	-0.00111999	0.00189557	0.00119489	3.30E-106	0.35	0.001424	479.4763844
Sitting height	Falls	rs13146142	4	C	T	-0.0491772	0.000506523	0.00258189	0.0016172	7.68E-81	0.75	0.001078	362.787341
Sitting height	Falls	rs145216811	4	A	G	0.0425368	-0.000531017	0.00694827	0.00439047	9.26E-10	0.9	0.000111	37.47798717
Sitting height	Falls	rs1662835	4	C	T	0.0264333	0.00252022	0.00203991	0.0012865	2.16E-38	0.05	0.000499	167.9116217
Sitting height	Falls	rs17600719	4	A	G	0.0212093	0.00190782	0.00256826	0.00161758	1.48E-16	0.24	0.000203	68.19847991
Sitting height	Falls	rs2162202	4	C	T	0.0108419	-0.000447572	0.00188442	0.0011883	8.75E-09	0.709999	9.85E-05	33.10208768
Sitting height	Falls	rs2306596	4	A	C	0.0105638	0.00187581	0.00187988	0.00118368	1.92E-08	0.11	9.39E-05	31.57767028
Sitting height	Falls	rs2610986	4	T	C	-0.0185388	0.000372059	0.00202753	0.00127973	6.08E-20	0.77	0.000249	83.60430879
Sitting height	Falls	rs2627692	4	T	C	-0.0222825	-0.00129672	0.00187833	0.0011821	1.87E-32	0.27	0.000418	140.7291422
Sitting height	Falls	rs2699427	4	C	T	-0.0158138	-0.00346004	0.00271342	0.00170728	5.61E-09	0.0430002	0.000101	33.96553348
Sitting height	Falls	rs312330	4	T	C	-0.0104733	0.00187416	0.00197264	0.00124515	1.10E-07	0.13	8.38E-05	28.18846435
Sitting height	Falls	rs3828559	4	G	A	0.0130154	0.0023656	0.00219234	0.00138214	2.91E-09	0.0870001	0.000105	35.2451387
Sitting height	Falls	rs4621412	4	A	G	-0.0149134	0.000753963	0.00192124	0.00121006	8.36E-15	0.53	0.000179	60.25458406
Sitting height	Falls	rs55915313	4	G	A	0.0197499	-0.00212091	0.00389994	0.00245768	4.10E-07	0.39	7.63E-05	25.64566417
Sitting height	Falls	rs55997920	4	G	A	-0.0184166	0.00116636	0.00200186	0.00126361	3.61E-20	0.36	0.000252	84.63529404
Sitting height	Falls	rs62289300	4	A	G	0.0135497	-0.0023154	0.00187956	0.00118364	5.65E-13	0.05	0.000155	51.96931249
Sitting height	Falls	rs62316290	4	C	G	0.0107034	0.00274381	0.00209054	0.00131759	3.06E-07	0.0369999	7.80E-05	26.21359277
Sitting height	Falls	rs62323058	4	C	T	-0.0146462	-0.000330115	0.0028578	0.00181777	2.98E-07	0.86	7.81E-05	26.26553533
Sitting height	Falls	rs6554107	4	A	G	-0.014687	0.00037423	0.00242582	0.00152378	1.41E-09	0.81	0.000109	36.65633536
Sitting height	Falls	rs66746153	4	G	T	0.0126283	0.000279743	0.00207584	0.00130758	1.18E-09	0.83	0.00011	37.00854649
Sitting height	Falls	rs6817908	4	A	G	0.0148388	-0.000745219	0.00285943	0.00179616	2.11E-07	0.68	8.01E-05	26.9301408
Sitting height	Falls	rs6818581	4	G	A	0.0439352	0.00788079	0.00852342	0.005413	2.54E-07	0.15	7.90E-05	26.57035989
Sitting height	Falls	rs7666178	4	T	A	0.0138243	-0.00116008	0.00204151	0.001288	1.28E-11	0.37	0.000136	45.85463876

Sitting height	Falls	rs7681267	4	C	T	0.0457174	0.00317019	0.00732406	0.00465678	4.32E-10	0.5	0.000116	38.96361651
Sitting height	Falls	rs7688346	4	G	A	-0.0163355	0.00207556	0.00272414	0.00170204	2.02E-09	0.22	0.000107	35.9588685
Sitting height	Falls	rs7697556	4	C	T	-0.00967821	-0.00143497	0.00187909	0.00118448	2.60E-07	0.23	7.89E-05	26.52741428
Sitting height	Falls	rs10055993	5	C	A	0.0202467	0.00153223	0.00279765	0.0017668	4.60E-13	0.39	0.000156	52.37472232
Sitting height	Falls	rs10066581	5	G	A	-0.0146656	0.00359413	0.00257483	0.00163063	1.23E-08	0.0280001	9.65E-05	32.44162127
Sitting height	Falls	rs10514136	5	G	A	-0.0280792	-0.000795469	0.00220437	0.00139214	3.71E-37	0.57	0.000482	162.2558921
Sitting height	Falls	rs10515235	5	A	G	-0.0109775	-0.00070577	0.00210797	0.00133133	1.91E-07	0.6	8.07E-05	27.11927281
Sitting height	Falls	rs11738691	5	T	G	-0.0183312	-0.000728774	0.00198833	0.00125233	3.00E-20	0.56	0.000253	84.99724732
Sitting height	Falls	rs11738827	5	T	C	0.0102501	0.00248477	0.00195698	0.00123297	1.63E-07	0.0439997	8.16E-05	27.43363968
Sitting height	Falls	rs11954686	5	G	A	-0.0126013	-0.00187664	0.00244936	0.00154754	2.68E-07	0.23	7.87E-05	26.46826411
Sitting height	Falls	rs153560	5	A	G	0.013182	0.000522354	0.00193549	0.00122048	9.73E-12	0.67	0.000138	46.38534072
Sitting height	Falls	rs1582931	5	A	G	-0.0215543	-0.00450649	0.00189542	0.00119488	5.85E-30	0.00016	0.000385	129.3173894
Sitting height	Falls	rs158409	5	T	C	-0.0159945	-0.000182153	0.00208341	0.00130772	1.63E-14	0.89	0.000175	58.93751869
Sitting height	Falls	rs1593071	5	C	A	-0.0172188	-0.000752716	0.00188592	0.00118907	6.88E-20	0.53	0.000248	83.3602927
Sitting height	Falls	rs16896999	5	A	G	0.034497	0.00234123	0.00591893	0.00372474	5.61E-09	0.53	0.000101	33.96849063
Sitting height	Falls	rs1818782	5	C	A	0.0193494	-0.00247277	0.00198838	0.00125265	2.23E-22	0.0479999	0.000282	94.69700266
Sitting height	Falls	rs28421137	5	G	A	0.0150466	0.000875605	0.0019576	0.00123422	1.52E-14	0.48	0.000176	59.0784155
Sitting height	Falls	rs2974438	5	A	G	-0.022894	-0.000811776	0.00231234	0.00145426	4.16E-23	0.58	0.000292	98.02570472
Sitting height	Falls	rs31211	5	A	G	-0.0187304	0.000606686	0.00216796	0.00136482	5.66E-18	0.66	0.000222	74.64342767
Sitting height	Falls	rs32796	5	A	C	-0.0309004	-1.85E-05	0.00335308	0.00212201	3.11E-20	0.99	0.000253	84.92594188
Sitting height	Falls	rs33852	5	G	A	0.0226578	0.000520301	0.00200629	0.00126502	1.43E-29	0.68	0.000379	127.5404841
Sitting height	Falls	rs3756668	5	A	G	-0.0124057	-0.00384208	0.0018831	0.00118632	4.47E-11	0.0012	0.000129	43.4006049
Sitting height	Falls	rs4073717	5	T	G	-0.0318034	-0.0012648	0.00234391	0.00147631	6.31E-42	0.39	0.000547	184.1048541
Sitting height	Falls	rs4867721	5	T	G	-0.0233845	-0.00189286	0.00191947	0.0012096	3.90E-34	0.12	0.000441	148.4203732
Sitting height	Falls	rs4916664	5	T	C	-0.013409	-0.00167506	0.00187874	0.00118387	9.54E-13	0.16	0.000152	50.94005595
Sitting height	Falls	rs55758152	5	A	G	0.0147149	0.000562232	0.00202427	0.00127702	3.62E-13	0.66	0.000157	52.84181818

Sitting height	Falls	rs6453143	5	C	G	-0.0100608	-0.00109606	0.00195727	0.00123215	2.75E-07	0.37	7.86E-05	26.42187006
Sitting height	Falls	rs6555970	5	A	G	-0.0158909	0.000982252	0.00226544	0.00142491	2.31E-12	0.49	0.000146	49.20303141
Sitting height	Falls	rs6874142	5	G	T	0.0259348	0.00385558	0.00311373	0.00197162	8.17E-17	0.051	0.000206	69.37514509
Sitting height	Falls	rs7717604	5	T	C	0.0118906	-0.000121125	0.00197409	0.00124531	1.71E-09	0.92	0.000108	36.28053163
Sitting height	Falls	rs7733331	5	C	T	-0.026781	-0.00132948	0.00191757	0.00120837	2.58E-44	0.27	0.00058	195.0523224
Sitting height	Falls	rs7736027	5	G	A	0.0125802	-0.000358304	0.00207985	0.001309	1.46E-09	0.780001	0.000109	36.58567416
Sitting height	Falls	rs832552	5	T	G	0.0129399	-0.000622178	0.00197435	0.00124108	5.61E-11	0.62	0.000128	42.95498306
Sitting height	Falls	rs860664	5	G	T	0.0136498	-0.00181818	0.0026164	0.00164778	1.82E-07	0.27	8.10E-05	27.2172533
Sitting height	Falls	rs1040525	6	T	C	-0.0563999	5.22E-05	0.00208208	0.00130909	2.02E-161	0.97	0.002178	733.7731954
Sitting height	Falls	rs10498672	6	G	C	0.0276616	0.00335121	0.00245758	0.00155345	2.20E-29	0.0309999	0.000377	126.6891043
Sitting height	Falls	rs10807137	6	T	C	-0.0351127	-0.000968711	0.00247171	0.0015571	8.69E-46	0.53	0.0006	201.8056972
Sitting height	Falls	rs1102077	6	C	A	-0.0163016	-0.00181232	0.00220602	0.00138571	1.48E-13	0.19	0.000162	54.60615204
Sitting height	Falls	rs11243202	6	C	T	0.0265197	0.0015321	0.00188607	0.00118874	6.81E-45	0.2	0.000588	197.7067966
Sitting height	Falls	rs117542858	6	A	G	0.0417976	-0.00349119	0.00735103	0.00470714	1.30E-08	0.46	9.62E-05	32.33004438
Sitting height	Falls	rs12213409	6	G	T	-0.0168522	-0.000189363	0.00189241	0.00119287	5.36E-19	0.87	0.000236	79.30174441
Sitting height	Falls	rs12523788	6	A	T	-0.0197572	-0.00207689	0.00195323	0.00123413	4.77E-24	0.0920005	0.000304	102.31611
Sitting height	Falls	rs12662365	6	A	T	-0.0398604	0.00151725	0.00201634	0.001271	6.21E-87	0.23	0.001161	390.8010966
Sitting height	Falls	rs1319012	6	A	T	-0.0449378	0.00223726	0.00367068	0.00231404	1.88E-34	0.33	0.000446	149.8754717
Sitting height	Falls	rs1490384	6	T	C	0.035317	-0.00163067	0.00187642	0.00118296	5.53E-79	0.17	0.001053	354.2480793
Sitting height	Falls	rs17632964	6	G	A	-0.0205572	0.000638592	0.00317071	0.00200002	8.98E-11	0.75	0.000125	42.0353703
Sitting height	Falls	rs1963689	6	C	T	0.0151277	-0.00148342	0.00218252	0.00137573	4.18E-12	0.28	0.000143	48.04291413
Sitting height	Falls	rs228139	6	T	G	-0.0241423	-0.00382783	0.00310497	0.00195936	7.54E-15	0.051	0.00018	60.45642577
Sitting height	Falls	rs2523578	6	A	G	-0.0379524	-0.000338854	0.00205851	0.00129815	7.26E-76	0.79	0.00101	339.9167197
Sitting height	Falls	rs270261	6	T	C	0.0101925	-0.00182042	0.00202251	0.00127534	4.67E-07	0.15	7.55E-05	25.39686352
Sitting height	Falls	rs35146243	6	C	G	-0.02361	0.000575239	0.00190815	0.00120265	3.71E-35	0.630001	0.000455	153.0970953
Sitting height	Falls	rs3853252	6	A	G	0.0217843	0.000201022	0.00189187	0.00119386	1.13E-30	0.87	0.000394	132.5881275

Sitting height	Falls	rs41271299	6	T	C	0.0603444	0.000508806	0.00424138	0.00268132	6.38E-46	0.85	0.000602	202.4228703
Sitting height	Falls	rs4380799	6	G	T	-0.0238388	0.00160018	0.00212369	0.00134155	3.10E-29	0.23	0.000375	126.0046395
Sitting height	Falls	rs483981	6	C	T	0.0215076	-0.00109744	0.00229622	0.00144857	7.54E-21	0.450001	0.000261	87.73177397
Sitting height	Falls	rs62390617	6	C	G	0.0160552	0.00154146	0.00278743	0.00175325	8.43E-09	0.38	9.87E-05	33.17596011
Sitting height	Falls	rs6938628	6	C	T	-0.0148625	0.00051098	0.00284091	0.00177646	1.68E-07	0.77	8.14E-05	27.36962104
Sitting height	Falls	rs76453248	6	G	A	0.0212797	-0.000359981	0.00287232	0.00181755	1.28E-13	0.84	0.000163	54.88647725
Sitting height	Falls	rs766406	6	T	G	-0.0178284	-0.00398948	0.00194581	0.00122764	5.10E-20	0.0012	0.00025	83.95061399
Sitting height	Falls	rs76659622	6	G	C	-0.0146679	-0.00116474	0.00209559	0.00132008	2.57E-12	0.38	0.000146	48.99178309
Sitting height	Falls	rs768023	6	A	G	0.0183117	0.00128198	0.00194199	0.00122271	4.15E-21	0.29	0.000264	88.91260834
Sitting height	Falls	rs7753558	6	A	C	0.0258951	-0.00189596	0.00196242	0.00123557	9.53E-40	0.12	0.000518	174.1210439
Sitting height	Falls	rs852927	6	T	C	0.0148164	0.00135218	0.00194753	0.00122613	2.80E-14	0.27	0.000172	57.87847448
Sitting height	Falls	rs9321266	6	G	A	0.0203124	-0.000533205	0.00192955	0.00121628	6.54E-26	0.66	0.00033	110.8180252
Sitting height	Falls	rs9344126	6	C	T	-0.0252245	0.000204513	0.00189667	0.00119577	2.39E-40	0.86	0.000526	176.8730166
Sitting height	Falls	rs9353214	6	G	C	-0.0141461	-0.00257368	0.0018837	0.00118674	5.94E-14	0.0299999	0.000168	56.39621689
Sitting height	Falls	rs9357471	6	T	C	0.0228125	-0.00141889	0.0019334	0.00121949	4.00E-32	0.24	0.000414	139.2202262
Sitting height	Falls	rs9379084	6	A	G	-0.0380032	-0.00281084	0.00302979	0.00190665	4.41E-36	0.14	0.000468	157.3313535
Sitting height	Falls	rs9391254	6	T	C	0.014134	-0.00250588	0.00200853	0.00126716	1.97E-12	0.0479999	0.000147	49.51918955
Sitting height	Falls	rs9459596	6	A	G	-0.0181089	5.03E-05	0.00187818	0.00118362	5.36E-22	0.97	0.000276	92.96291169
Sitting height	Falls	rs10228519	7	C	G	0.0116695	0.000592081	0.00187677	0.00118351	5.04E-10	0.62	0.000115	38.66182862
Sitting height	Falls	rs111872419	7	A	G	0.0140289	-0.00150018	0.00238355	0.00150348	3.97E-09	0.32	0.000103	34.64166087
Sitting height	Falls	rs113755488	7	T	C	-0.0136655	0.00377695	0.00264507	0.00168043	2.39E-07	0.025	7.94E-05	26.6917294
Sitting height	Falls	rs11555134	7	T	C	0.0171645	-0.00214862	0.00223792	0.00141118	1.73E-14	0.13	0.000175	58.8265256
Sitting height	Falls	rs1178124	7	G	A	0.0157448	-0.0023111	0.00233781	0.00147516	1.64E-11	0.12	0.000135	45.35820637
Sitting height	Falls	rs12534093	7	A	T	-0.0266955	0.00254901	0.00218192	0.00137595	2.06E-34	0.064	0.000445	149.6919592
Sitting height	Falls	rs12700901	7	A	C	-0.0212848	-0.000252398	0.00191965	0.00121045	1.45E-28	0.83	0.000366	122.9405187
Sitting height	Falls	rs143986132	7	G	A	-0.0384874	0.00626299	0.00721554	0.00460554	9.62E-08	0.17	8.46E-05	28.45112671

Sitting height	Falls	rs1450851	7	C	T	-0.0111303	0.00152066	0.00196422	0.00123847	1.46E-08	0.22	9.55E-05	32.10949556
Sitting height	Falls	rs16140	7	G	C	-0.0110051	0.00160398	0.00215254	0.00135423	3.18E-07	0.24	7.77E-05	26.13879275
Sitting height	Falls	rs17170170	7	G	A	-0.014676	0.000668051	0.00226967	0.00143335	1.01E-10	0.64	0.000124	41.81094386
Sitting height	Falls	rs2471548	7	C	T	0.0225808	0.00166316	0.00189669	0.00119488	1.13E-32	0.16	0.000421	141.7378822
Sitting height	Falls	rs2965026	7	T	C	0.0116642	0.00116108	0.00219309	0.00138326	1.05E-07	0.4	8.41E-05	28.28765887
Sitting height	Falls	rs3110795	7	T	G	0.0204882	-0.000739159	0.00243913	0.00154249	4.49E-17	0.630001	0.00021	70.55661129
Sitting height	Falls	rs34060476	7	G	A	0.0231686	0.00062792	0.00275534	0.0017404	4.16E-17	0.719999	0.00021	70.70484739
Sitting height	Falls	rs42044	7	G	T	0.0354857	-0.000499842	0.00211506	0.00133043	3.77E-63	0.709999	0.000837	281.4889619
Sitting height	Falls	rs45446698	7	G	T	0.0391421	-0.00259347	0.00463597	0.00295345	3.10E-17	0.38	0.000212	71.28645372
Sitting height	Falls	rs4720831	7	A	G	-0.0107144	-0.000336079	0.00190166	0.00119796	1.76E-08	0.780001	9.44E-05	31.744608
Sitting height	Falls	rs4727453	7	G	A	0.0110252	-6.49E-05	0.00190697	0.00120318	7.41E-09	0.96	9.94E-05	33.42606329
Sitting height	Falls	rs506154	7	C	T	-0.0224348	-0.00195174	0.00205718	0.00130039	1.10E-27	0.13	0.000354	118.9323
Sitting height	Falls	rs57920537	7	A	T	-0.0119992	0.00029989	0.00201301	0.00126598	2.51E-09	0.81	0.000106	35.53143271
Sitting height	Falls	rs58574564	7	G	A	0.0403178	-0.000729639	0.00483039	0.00298208	7.05E-17	0.81	0.000207	69.66734486
Sitting height	Falls	rs6956736	7	C	G	-0.0116437	0.000297729	0.00215972	0.00136346	7.00E-08	0.83	8.65E-05	29.0661235
Sitting height	Falls	rs6964492	7	C	T	-0.0136151	-0.00295361	0.00188624	0.0011891	5.28E-13	0.0129999	0.000155	52.1012053
Sitting height	Falls	rs6977416	7	A	G	0.0226952	-0.00209693	0.0020111	0.00126459	1.58E-29	0.0969996	0.000379	127.3505124
Sitting height	Falls	rs723149	7	G	A	-0.0168863	0.000771398	0.00189249	0.00119325	4.57E-19	0.52	0.000237	79.61626769
Sitting height	Falls	rs757717	7	C	T	0.0148059	0.00540072	0.00279396	0.00177175	1.16E-07	0.00230001	8.35E-05	28.0820789
Sitting height	Falls	rs798488	7	C	T	-0.0403206	0.000131911	0.00204781	0.00129168	2.96E-86	0.92	0.001152	387.6811217
Sitting height	Falls	rs822530	7	T	A	0.0253332	0.000726873	0.00233926	0.00147608	2.52E-27	0.62	0.000349	117.2797723
Sitting height	Falls	rs10097502	8	A	G	0.0104114	0.00102361	0.00202472	0.00127771	2.72E-07	0.42	7.86E-05	26.44163578
Sitting height	Falls	rs10103539	8	G	A	-0.0143835	-0.000514954	0.00192789	0.00121618	8.62E-14	0.67	0.000166	55.66274941
Sitting height	Falls	rs10109521	8	A	G	-0.0163744	6.86E-05	0.00189146	0.00119154	4.86E-18	0.95	0.000223	74.94393075
Sitting height	Falls	rs1036821	8	A	G	-0.0215027	0.00103048	0.00204358	0.00128804	6.90E-26	0.42	0.000329	110.7140414
Sitting height	Falls	rs10808583	8	A	G	-0.0397498	-0.00246349	0.00233985	0.00147407	1.07E-64	0.0949992	0.000858	288.5983617

Sitting height	Falls	rs11778175	8	T	C	-0.0118069	-0.00152364	0.0019468	0.00122456	1.32E-09	0.21	0.000109	36.78147107
Sitting height	Falls	rs11783086	8	C	T	0.0157735	0.00113043	0.00197277	0.0012421	1.29E-15	0.36	0.00019	63.929783
Sitting height	Falls	rs11786903	8	A	G	-0.0173211	0.00269054	0.00189876	0.00119751	7.39E-20	0.025	0.000247	83.21675751
Sitting height	Falls	rs13271337	8	C	G	-0.0148367	0.000653277	0.00194851	0.00122811	2.66E-14	0.59	0.000172	57.97881738
Sitting height	Falls	rs13271505	8	A	G	-0.0165926	0.00058392	0.00213111	0.00134458	6.94E-15	0.66	0.00018	60.6201705
Sitting height	Falls	rs147918758	8	G	C	-0.011589	0.00275957	0.00192912	0.00121698	1.89E-09	0.0230001	0.000107	36.08888286
Sitting height	Falls	rs1599473	8	T	G	-0.0186829	-0.00188627	0.00219449	0.00138209	1.69E-17	0.17	0.000216	72.48053097
Sitting height	Falls	rs2407277	8	G	C	0.0114667	0.00228142	0.0022612	0.00141969	3.96E-07	0.11	7.65E-05	25.71573532
Sitting height	Falls	rs2737218	8	C	T	-0.0235255	-1.64E-05	0.00230981	0.00146035	2.33E-24	0.99	0.000308	103.7349759
Sitting height	Falls	rs2954869	8	G	A	-0.0148206	0.000972356	0.00215161	0.00135339	5.66E-12	0.47	0.000141	47.44653089
Sitting height	Falls	rs310317	8	C	T	-0.0154116	-0.00228351	0.00189066	0.00119199	3.61E-16	0.0549997	0.000198	66.4459607
Sitting height	Falls	rs4737446	8	T	G	0.0147196	0.00097773	0.00204474	0.00129029	6.09E-13	0.450001	0.000154	51.82219819
Sitting height	Falls	rs62515432	8	C	T	0.0379726	0.00187187	0.00231628	0.00146267	2.24E-60	0.2	0.000799	268.7562387
Sitting height	Falls	rs6473015	8	C	A	0.0322543	0.000244601	0.00207674	0.00130989	2.23E-54	0.85	0.000717	241.2187076
Sitting height	Falls	rs6997082	8	C	T	-0.0156339	0.000762629	0.00205498	0.00129951	2.79E-14	0.56	0.000172	57.87879229
Sitting height	Falls	rs72722756	8	C	T	0.0218768	-0.00172973	0.00246203	0.00154353	6.38E-19	0.26	0.000235	78.95523161
Sitting height	Falls	rs76020419	8	T	G	-0.0294604	1.91E-05	0.00506582	0.00319367	6.05E-09	1	0.000101	33.82032447
Sitting height	Falls	rs7822301	8	G	T	0.0110903	0.00133821	0.00193712	0.00122253	1.03E-08	0.27	9.75E-05	32.77732734
Sitting height	Falls	rs10122419	9	T	C	-0.0142599	0.000719686	0.0022187	0.00139984	1.30E-10	0.61	0.000123	41.30815455
Sitting height	Falls	rs10746837	9	A	G	-0.023893	0.0030147	0.00191274	0.00120585	8.46E-36	0.012	0.000464	156.0376805
Sitting height	Falls	rs10817161	9	C	G	-0.0125266	-0.00116294	0.0019028	0.0011981	4.61E-11	0.33	0.000129	43.33912451
Sitting height	Falls	rs12347137	9	C	A	-0.0362287	0.00165336	0.00233054	0.00147503	1.79E-54	0.26	0.000718	241.6531042
Sitting height	Falls	rs13283037	9	A	G	0.0155338	-0.00221419	0.00198743	0.00125111	5.47E-15	0.0769999	0.000182	61.09022662
Sitting height	Falls	rs1553728	9	T	G	0.013273	-0.00237274	0.00190115	0.00119809	2.92E-12	0.0479999	0.000145	48.74223296
Sitting height	Falls	rs1983753	9	A	G	0.0166606	-0.00213888	0.00265391	0.00167137	3.44E-10	0.2	0.000117	39.41022311
Sitting height	Falls	rs2181071	9	T	C	0.0299381	-0.00484	0.00240243	0.00151639	1.23E-35	0.00140001	0.000462	155.2912496

Sitting height	Falls	rs2274116	9	T	C	-0.0214607	-0.000673563	0.0019863	0.00125407	3.32E-27	0.59	0.000347	116.7341921
Sitting height	Falls	rs2381770	9	A	G	-0.0170959	-0.000875143	0.0032404	0.00203602	1.32E-07	0.67	8.28E-05	27.83470934
Sitting height	Falls	rs2493638	9	T	C	0.0247797	0.00143826	0.00258611	0.00162563	9.59E-22	0.38	0.000273	91.81171527
Sitting height	Falls	rs28457693	9	G	A	0.0481407	-0.00344365	0.00304406	0.00191959	2.58E-56	0.0729995	0.000743	250.1027029
Sitting height	Falls	rs28615587	9	G	T	0.0118132	0.000574864	0.00203795	0.00128639	6.77E-09	0.649999	9.99E-05	33.60067982
Sitting height	Falls	rs35307904	9	A	G	-0.025626	0.000655537	0.00288149	0.00181402	5.96E-19	0.719999	0.000235	79.09106256
Sitting height	Falls	rs35990522	9	A	T	0.0296196	0.00247451	0.00355998	0.00224931	8.82E-17	0.27	0.000206	69.22504748
Sitting height	Falls	rs3789280	9	A	T	0.0124838	0.00189083	0.00196227	0.00123864	2.00E-10	0.13	0.00012	40.47399523
Sitting height	Falls	rs4258054	9	C	T	-0.0117456	0.00193897	0.00211313	0.00133191	2.72E-08	0.15	9.19E-05	30.89569592
Sitting height	Falls	rs57205988	9	A	C	-0.0120687	0.0021352	0.0023844	0.00149965	4.16E-07	0.15	7.62E-05	25.61903443
Sitting height	Falls	rs6475052	9	T	C	0.0154308	0.000218214	0.00191475	0.00120635	7.72E-16	0.86	0.000193	64.94604788
Sitting height	Falls	rs7869176	9	G	A	0.0159977	0.000843758	0.00224098	0.00141307	9.44E-13	0.55	0.000152	50.9611401
Sitting height	Falls	rs79634589	9	A	G	-0.0268219	0.00118897	0.00390164	0.0024617	6.23E-12	0.630001	0.000141	47.25901743
Sitting height	Falls	rs80322448	9	A	G	-0.0198563	-0.000879888	0.00339344	0.00214214	4.88E-09	0.68	0.000102	34.23862395
Sitting height	Falls	rs9409609	9	A	G	0.0167228	0.000583899	0.00238632	0.00150409	2.43E-12	0.7	0.000146	49.10894782
Sitting height	Falls	rs944593	9	C	G	-0.0154181	-6.54E-05	0.00188512	0.00118736	2.88E-16	0.96	0.000199	66.89346834
Sitting height	Falls	rs9657746	9	G	C	-0.0195512	-0.00131034	0.00197679	0.00124773	4.62E-23	0.29	0.000291	97.81957371
Sitting height	Falls	rs10082476	10	G	A	-0.0141097	0.000827256	0.00217644	0.00137394	9.01E-11	0.55	0.000125	42.02833272
Sitting height	Falls	rs10786678	10	T	C	-0.0217335	-0.00141092	0.0019072	0.00120237	4.46E-30	0.24	0.000386	129.8574489
Sitting height	Falls	rs10822055	10	A	T	-0.01807	0.000983804	0.00221567	0.00139756	3.49E-16	0.48	0.000198	66.51294082
Sitting height	Falls	rs10823137	10	C	A	0.0140846	-0.000395725	0.00212541	0.0013367	3.44E-11	0.77	0.000131	43.91406052
Sitting height	Falls	rs11014285	10	A	G	0.0239078	-0.000165906	0.00255832	0.00161252	9.23E-21	0.92	0.00026	87.33121376
Sitting height	Falls	rs11198886	10	T	C	0.0202668	-0.00174652	0.00316798	0.00200682	1.58E-10	0.38	0.000122	40.92658438
Sitting height	Falls	rs11204260	10	A	T	0.0105057	-0.000272691	0.00203154	0.00128077	2.33E-07	0.83	7.95E-05	26.74232942
Sitting height	Falls	rs11253170	10	G	C	0.0140221	-0.000267713	0.00201911	0.00127046	3.80E-12	0.83	0.000143	48.22876717
Sitting height	Falls	rs11599090	10	A	G	-0.0106024	0.00234582	0.00202118	0.00127419	1.56E-07	0.0659994	8.18E-05	27.51682904

Sitting height	Falls	rs1171614	10	C	T	-0.0191292	0.00115818	0.00222955	0.00140631	9.54E-18	0.41	0.000219	73.61379087
Sitting height	Falls	rs117543413	10	T	C	-0.0593217	0.000927084	0.00716977	0.00455148	1.30E-16	0.84	0.000204	68.45682113
Sitting height	Falls	rs1900016	10	T	C	0.0236668	-0.000136232	0.00187832	0.00118333	2.15E-36	0.91	0.000472	158.7595707
Sitting height	Falls	rs2181834	10	T	G	0.0166888	0.000954266	0.00188714	0.00119013	9.33E-19	0.42	0.000233	78.20634329
Sitting height	Falls	rs2631676	10	G	A	0.0187601	0.00064476	0.00239032	0.0015109	4.23E-15	0.67	0.000183	61.59680804
Sitting height	Falls	rs3740535	10	C	T	-0.0114123	0.00302209	0.00218282	0.00137753	1.71E-07	0.0280001	8.13E-05	27.33446075
Sitting height	Falls	rs4980067	10	A	C	-0.0183978	-0.00262634	0.00187834	0.00118274	1.19E-22	0.0259998	0.000285	95.93639007
Sitting height	Falls	rs555438	10	G	A	-0.0121472	-0.000320316	0.00223487	0.00140971	5.47E-08	0.82	8.79E-05	29.54254003
Sitting height	Falls	rs57822625	10	T	C	0.0205127	-0.000723892	0.00286945	0.00180526	8.78E-13	0.69	0.000152	51.103228
Sitting height	Falls	rs6584383	10	G	C	-0.0141805	-0.00223313	0.00226709	0.00142922	3.98E-10	0.12	0.000116	39.1242086
Sitting height	Falls	rs76216796	10	C	T	-0.0354196	0.00253138	0.00294596	0.0018482	2.73E-33	0.17	0.00043	144.5551648
Sitting height	Falls	rs7898996	10	G	A	-0.00984297	0.0018029	0.00189919	0.00119698	2.19E-07	0.13	7.99E-05	26.86058662
Sitting height	Falls	rs7910211	10	C	T	0.0242074	-0.000871631	0.00258915	0.00162048	8.85E-21	0.59	0.00026	87.41417633
Sitting height	Falls	rs7914592	10	C	T	-0.0170596	9.80E-05	0.00253758	0.00159564	1.79E-11	0.95	0.000134	45.19581293
Sitting height	Falls	rs7920126	10	A	T	0.0112789	-0.00200752	0.00212571	0.0013373	1.12E-07	0.13	8.37E-05	28.15305062
Sitting height	Falls	rs11038720	11	T	A	0.0121596	0.00165779	0.00197664	0.00124647	7.68E-10	0.18	0.000113	37.84281356
Sitting height	Falls	rs11039389	11	C	T	-0.0213783	-0.00305497	0.00196628	0.00123865	1.58E-27	0.0140001	0.000352	118.2103793
Sitting height	Falls	rs113875379	11	C	A	0.0339835	-0.00313075	0.00520608	0.00329265	6.69E-11	0.34	0.000127	42.61029449
Sitting height	Falls	rs12222197	11	T	G	0.01636	0.000498966	0.00190961	0.00120429	1.06E-17	0.68	0.000218	73.39681901
Sitting height	Falls	rs144998346	11	A	G	-0.0225998	0.00317617	0.00251553	0.00159172	2.62E-19	0.0460002	0.00024	80.71424515
Sitting height	Falls	rs1468291	11	C	T	0.0135738	0.000715741	0.00192147	0.00120998	1.62E-12	0.55	0.000148	49.90403654
Sitting height	Falls	rs1971929	11	C	G	0.0275257	-0.00328139	0.00265975	0.00168112	4.27E-25	0.051	0.000318	107.1013905
Sitting height	Falls	rs2509180	11	A	G	-0.0149975	-0.00353591	0.00290492	0.0018237	2.43E-07	0.0530005	7.93E-05	26.65442928
Sitting height	Falls	rs35506085	11	A	G	-0.0268363	-0.000582253	0.00245325	0.00153518	7.58E-28	0.7	0.000356	119.663489
Sitting height	Falls	rs596864	11	G	A	-0.0101128	0.00308486	0.00190632	0.00120116	1.13E-07	0.01	8.37E-05	28.14175826
Sitting height	Falls	rs615133	11	A	C	0.0105378	-0.00133181	0.00188965	0.00119212	2.46E-08	0.26	9.25E-05	31.09833659

Sitting height	Falls	rs622722	11	A	G	0.0124149	-0.00072057	0.00193755	0.00122163	1.48E-10	0.56	0.000122	41.0563762
Sitting height	Falls	rs704660	11	T	C	0.016044	0.00319186	0.00190846	0.00120362	4.23E-17	0.008	0.00021	70.67392038
Sitting height	Falls	rs7119815	11	A	G	0.0105828	-8.10E-06	0.00189362	0.00119307	2.29E-08	0.99	9.29E-05	31.23313034
Sitting height	Falls	rs72979233	11	G	A	-0.020855	0.00251224	0.00218146	0.00137362	1.18E-21	0.0669993	0.000272	91.39572459
Sitting height	Falls	rs7928209	11	C	T	0.0251527	0.00103183	0.00385078	0.00243763	6.51E-11	0.67	0.000127	42.66500559
Sitting height	Falls	rs8177343	11	A	C	-0.0132271	0.000798719	0.00255501	0.00161376	2.26E-07	0.62	7.97E-05	26.80057213
Sitting height	Falls	rs10744078	12	G	T	0.0101437	-0.00190952	0.00190474	0.00120156	1.01E-07	0.11	8.44E-05	28.36099012
Sitting height	Falls	rs10745666	12	T	G	-0.0165671	-0.000407257	0.00188185	0.00118665	1.33E-18	0.73	0.00023	77.50379718
Sitting height	Falls	rs10748128	12	T	G	0.0196193	0.000164885	0.00197494	0.00124565	2.98E-23	0.89	0.000293	98.68683119
Sitting height	Falls	rs10777534	12	C	T	-0.0217632	0.00105792	0.00196019	0.00123569	1.23E-28	0.39	0.000367	123.2676645
Sitting height	Falls	rs10849032	12	A	G	0.0108608	-0.00221978	0.00191958	0.00120854	1.53E-08	0.0659994	9.52E-05	32.01188144
Sitting height	Falls	rs10861879	12	A	G	0.0183301	-0.00209694	0.00204034	0.00128387	2.63E-19	0.1	0.00024	80.70948589
Sitting height	Falls	rs11049698	12	A	G	0.0185271	-0.00039053	0.00189046	0.00119251	1.13E-22	0.74	0.000286	96.04613786
Sitting height	Falls	rs11060406	12	T	C	-0.0306618	0.00280884	0.00505463	0.00319639	1.31E-09	0.38	0.000109	36.79735069
Sitting height	Falls	rs11067228	12	G	A	0.0112252	-0.000526274	0.0018837	0.00118776	2.54E-09	0.66	0.000106	35.51114696
Sitting height	Falls	rs11107120	12	C	T	0.050292	-0.000901485	0.00225781	0.00142784	7.82E-110	0.53	0.001474	496.1614628
Sitting height	Falls	rs11113186	12	A	G	-0.0108437	-6.57E-05	0.00194251	0.00122487	2.38E-08	0.96	9.27E-05	31.16222518
Sitting height	Falls	rs11615175	12	A	G	0.0151214	-0.00124492	0.002023	0.00127635	7.75E-14	0.33	0.000166	55.87174531
Sitting height	Falls	rs118115924	12	T	G	-0.0483928	0.00343218	0.00872476	0.00548412	2.91E-08	0.53	9.15E-05	30.76483028
Sitting height	Falls	rs1215761	12	T	C	-0.0102968	-0.00148188	0.00194688	0.00122735	1.23E-07	0.23	8.32E-05	27.97217182
Sitting height	Falls	rs12818067	12	A	G	0.0339097	0.000908058	0.00234735	0.0014784	2.75E-47	0.54	0.00062	208.6854624
Sitting height	Falls	rs1444643	12	A	C	0.0142381	0.00130604	0.00203432	0.00127969	2.58E-12	0.31	0.000146	48.98527383
Sitting height	Falls	rs145350287	12	A	T	-0.0290657	-0.00439595	0.00481039	0.00302907	1.52E-09	0.15	0.000109	36.50908909
Sitting height	Falls	rs2732439	12	A	C	0.0165352	-0.000555793	0.00188788	0.00119012	1.99E-18	0.64	0.000228	76.71320602
Sitting height	Falls	rs310797	12	T	G	0.0121442	0.00148928	0.00199211	0.00125544	1.09E-09	0.24	0.000111	37.1630364
Sitting height	Falls	rs35756741	12	T	C	-0.0233925	0.00607859	0.00326163	0.00205477	7.40E-13	0.00309999	0.000153	51.43797822

Sitting height	Falls	rs4456353	12	G	C	-0.011638	0.000876998	0.00190639	0.00120216	1.03E-09	0.47	0.000111	37.26775255
Sitting height	Falls	rs6581626	12	G	A	-0.014445	0.000335101	0.00188459	0.00118819	1.80E-14	0.780001	0.000175	58.74911486
Sitting height	Falls	rs709284	12	C	T	0.0148066	0.00124271	0.00196249	0.00123734	4.54E-14	0.32	0.000169	56.92404893
Sitting height	Falls	rs7132825	12	A	G	-0.023747	0.00372238	0.00339719	0.00213	2.75E-12	0.0810009	0.000145	48.86274156
Sitting height	Falls	rs7135760	12	T	C	0.010415	0.00119721	0.00205908	0.00129662	4.24E-07	0.36	7.61E-05	25.5842157
Sitting height	Falls	rs74431741	12	A	G	0.0415191	-0.000790255	0.00438145	0.00278239	2.66E-21	0.780001	0.000267	89.79665139
Sitting height	Falls	rs74900775	12	C	T	0.0359573	-0.00597501	0.00472465	0.00297291	2.74E-14	0.0439997	0.000172	57.92084161
Sitting height	Falls	rs76895963	12	G	T	0.120269	-0.00242466	0.00727563	0.0045803	2.34E-61	0.6	0.000812	273.2537872
Sitting height	Falls	rs7961994	12	T	A	0.0195784	-0.000370602	0.00192043	0.00121156	2.11E-24	0.760001	0.000309	103.9339495
Sitting height	Falls	rs7968682	12	T	G	-0.0306998	0.00192254	0.00188605	0.00118768	1.51E-59	0.11	0.000788	264.9504666
Sitting height	Falls	rs7971877	12	G	T	-0.0150386	-0.00314247	0.00190671	0.00120166	3.10E-15	0.0089	0.000185	62.20789809
Sitting height	Falls	rs79747671	12	T	C	-0.0328037	-0.000429005	0.00301481	0.00190526	1.44E-27	0.82	0.000352	118.39293
Sitting height	Falls	rs9804944	12	G	C	-0.0200551	0.000553248	0.00228573	0.00144624	1.73E-18	0.7	0.000229	76.98388195
Sitting height	Falls	rs11840862	13	G	A	-0.0135069	0.00231921	0.0020638	0.00129701	5.97E-11	0.0739997	0.000127	42.83276917
Sitting height	Falls	rs1327313	13	A	T	0.0142366	-0.00310276	0.0022704	0.00143485	3.60E-10	0.0309999	0.000117	39.319484
Sitting height	Falls	rs1330062	13	G	C	-0.0181456	-0.00214539	0.00194636	0.00122561	1.14E-20	0.08	0.000258	86.91531824
Sitting height	Falls	rs146851424	13	C	A	0.0804127	0.00395487	0.00642885	0.00409964	6.87E-36	0.33	0.000465	156.4525707
Sitting height	Falls	rs17078904	13	A	G	0.0127807	-0.000567669	0.00224746	0.00141604	1.30E-08	0.69	9.62E-05	32.33890717
Sitting height	Falls	rs2225226	13	T	C	-0.0397622	-0.00155109	0.00227929	0.0014403	4.03E-68	0.28	0.000904	304.3278099
Sitting height	Falls	rs2772172	13	A	G	-0.0166833	0.00230809	0.00252182	0.00158529	3.71E-11	0.15	0.00013	43.76588848
Sitting height	Falls	rs3764129	13	C	G	-0.0141069	0.00173873	0.00234474	0.00147387	1.79E-09	0.24	0.000108	36.19710111
Sitting height	Falls	rs7994573	13	C	T	-0.0163609	-0.00245137	0.00221444	0.00139433	1.49E-13	0.0790005	0.000162	54.58666292
Sitting height	Falls	rs9543069	13	G	C	0.0143267	0.00076338	0.002739	0.00173191	1.69E-07	0.66	8.14E-05	27.35950712
Sitting height	Falls	rs9557276	13	T	G	0.0109099	-0.00304482	0.00188048	0.0011854	6.57E-09	0.01	0.0001	33.65922422
Sitting height	Falls	rs1003740	14	C	T	-0.0101738	0.000774865	0.00191036	0.00120283	1.01E-07	0.52	8.44E-05	28.36194167
Sitting height	Falls	rs10131337	14	T	C	0.0148863	-0.000236137	0.00219197	0.00138325	1.11E-11	0.86	0.000137	46.12159544

Sitting height	Falls	rs10137553	14	A	C	-0.0105204	0.000749091	0.00201239	0.00126594	1.72E-07	0.55	8.13E-05	27.33003602
Sitting height	Falls	rs10400773	14	T	C	-0.0168318	-0.00142446	0.00233968	0.00147171	6.30E-13	0.33	0.000154	51.75450973
Sitting height	Falls	rs10483727	14	C	T	-0.0278843	-0.00049256	0.00192768	0.00121269	2.08E-47	0.68	0.000622	209.2423602
Sitting height	Falls	rs112635299	14	T	G	0.0805155	-0.0055112	0.0065086	0.0041733	3.84E-35	0.19	0.000455	153.0325534
Sitting height	Falls	rs11846330	14	T	C	0.0103655	0.000680296	0.00190306	0.00119989	5.13E-08	0.57	8.82E-05	29.66713104
Sitting height	Falls	rs12586289	14	T	G	0.0140789	-0.00107779	0.0018976	0.00119525	1.18E-13	0.37	0.000164	55.0462965
Sitting height	Falls	rs12884366	14	G	A	0.0129295	-0.00179794	0.00192314	0.00121351	1.78E-11	0.14	0.000134	45.20033556
Sitting height	Falls	rs2296316	14	C	T	-0.0175078	0.00390836	0.00189612	0.00119486	2.63E-20	0.00109999	0.000254	85.25728831
Sitting height	Falls	rs2319817	14	A	G	0.0226424	-0.00203227	0.00299481	0.00189076	4.02E-14	0.28	0.00017	57.16186205
Sitting height	Falls	rs35511437	14	A	G	0.0111175	0.000674099	0.00220849	0.00138888	4.81E-07	0.630001	7.54E-05	25.34097976
Sitting height	Falls	rs3819779	14	C	G	-0.015599	-0.000247977	0.00237724	0.00149401	5.32E-11	0.87	0.000128	43.05736591
Sitting height	Falls	rs4903905	14	A	G	-0.010525	0.000524277	0.0019678	0.00124021	8.87E-08	0.67	8.51E-05	28.60765743
Sitting height	Falls	rs60908064	14	G	A	0.0179307	0.00144875	0.00268381	0.00169348	2.38E-11	0.39	0.000133	44.63658444
Sitting height	Falls	rs708545	14	G	T	0.0131015	-0.000185878	0.00240382	0.00150863	5.03E-08	0.9	8.84E-05	29.70558804
Sitting height	Falls	rs11070250	15	A	G	0.0114541	0.00138356	0.00207521	0.00131006	3.40E-08	0.29	9.06E-05	30.46476538
Sitting height	Falls	rs11635385	15	A	G	-0.0135581	-0.000753859	0.00217906	0.00137354	4.92E-10	0.58	0.000115	38.71321935
Sitting height	Falls	rs12900487	15	C	T	0.0159088	-0.00102878	0.00187541	0.00118314	2.21E-17	0.38	0.000214	71.95854765
Sitting height	Falls	rs12904319	15	C	A	0.0256749	0.00113903	0.00290728	0.00182329	1.04E-18	0.53	0.000232	77.99087632
Sitting height	Falls	rs12906244	15	A	G	0.0469719	0.00563227	0.00694166	0.00442044	1.32E-11	0.2	0.000136	45.78777908
Sitting height	Falls	rs17200030	15	T	C	-0.042317	-0.00402547	0.0055165	0.00349582	1.71E-14	0.25	0.000175	58.84404215
Sitting height	Falls	rs2119260	15	C	T	-0.0229295	-0.000107566	0.00207422	0.00130749	2.11E-28	0.93	0.000363	122.2023434
Sitting height	Falls	rs2124135	15	G	A	0.0322845	-4.70E-05	0.00397192	0.00247905	4.37E-16	0.98	0.000196	66.06738908
Sitting height	Falls	rs2663126	15	A	G	-0.0154513	-0.00203144	0.00202956	0.00127937	2.68E-14	0.11	0.000172	57.95971747
Sitting height	Falls	rs28542042	15	T	C	0.0121378	0.00208509	0.00204776	0.00129254	3.08E-09	0.11	0.000105	35.13353455
Sitting height	Falls	rs2871865	15	G	C	-0.0455629	0.00250036	0.0029359	0.00184541	2.69E-54	0.18	0.000716	240.846422
Sitting height	Falls	rs4988781	15	C	A	-0.0145176	-0.00224662	0.00200681	0.00126335	4.69E-13	0.0749998	0.000156	52.33318172

Sitting height	Falls	rs62011286	15	A	G	0.013561	-0.000964826	0.00197878	0.00124795	7.23E-12	0.44	0.00014	46.96652274
Sitting height	Falls	rs7162063	15	G	A	0.0106148	0.000391559	0.00198731	0.00125258	9.24E-08	0.75	8.49E-05	28.52938408
Sitting height	Falls	rs7176941	15	G	A	0.0212707	0.000250648	0.00188235	0.00118731	1.33E-29	0.83	0.00038	127.6917409
Sitting height	Falls	rs7179506	15	G	T	-0.0158497	-0.00104969	0.00190084	0.00119848	7.57E-17	0.38	0.000207	69.52659616
Sitting height	Falls	rs7182563	15	G	A	0.0207478	0.00246415	0.00187813	0.0011837	2.29E-28	0.0369999	0.000363	122.0373606
Sitting height	Falls	rs72770234	15	A	T	-0.0277433	0.00178186	0.00385035	0.00244062	5.80E-13	0.47	0.000154	51.91774466
Sitting height	Falls	rs8029053	15	T	C	-0.0129123	7.48E-05	0.00208512	0.00131716	5.93E-10	0.95	0.000114	38.34821094
Sitting height	Falls	rs8042740	15	T	C	-0.0246216	0.00382291	0.0033357	0.00210046	1.57E-13	0.0690001	0.000162	54.4826939
Sitting height	Falls	rs10445034	16	T	C	-0.0200797	0.00100218	0.00222238	0.0013977	1.65E-19	0.47	0.000243	81.63526368
Sitting height	Falls	rs1046991	16	T	A	-0.0106013	0.000320012	0.00192556	0.00121387	3.68E-08	0.79	9.02E-05	30.31127032
Sitting height	Falls	rs10794639	16	G	A	-0.0150225	0.0023155	0.00188204	0.00118627	1.44E-15	0.051	0.000189	63.71280512
Sitting height	Falls	rs112898929	16	A	T	-0.023204	0.00373065	0.00351444	0.00222432	4.05E-11	0.0940005	0.00013	43.59266771
Sitting height	Falls	rs11540358	16	G	C	0.0208059	-0.00206607	0.00203307	0.0012816	1.41E-24	0.11	0.000311	104.7293359
Sitting height	Falls	rs116008080	16	A	G	-0.0397402	-0.00196056	0.00616378	0.0039206	1.14E-10	0.62	0.000124	41.56864214
Sitting height	Falls	rs11864330	16	A	G	-0.01643	-0.000217539	0.00229701	0.0014454	8.52E-13	0.88	0.000152	51.16221696
Sitting height	Falls	rs12925127	16	A	G	0.0137318	0.000433145	0.00198835	0.00125399	4.99E-12	0.73	0.000142	47.69460667
Sitting height	Falls	rs13336428	16	A	G	-0.0129058	-0.00121003	0.00190569	0.00120083	1.27E-11	0.31	0.000136	45.86330537
Sitting height	Falls	rs1741	16	C	G	-0.0147239	-0.000314711	0.00205913	0.00129686	8.66E-13	0.81	0.000152	51.13028181
Sitting height	Falls	rs26832	16	G	A	0.0165871	0.00265124	0.00190747	0.00120304	3.46E-18	0.0280001	0.000225	75.6180534
Sitting height	Falls	rs30215	16	C	A	0.0107543	0.00019641	0.00204478	0.00128947	1.45E-07	0.88	8.23E-05	27.6612064
Sitting height	Falls	rs3810817	16	C	A	-0.0169507	5.50E-07	0.00303805	0.0019018	2.41E-08	1	9.26E-05	31.13045303
Sitting height	Falls	rs3848370	16	C	G	0.0257766	0.00204701	0.00221863	0.00139737	3.38E-31	0.14	0.000401	134.983754
Sitting height	Falls	rs4985445	16	G	A	-0.0239689	0.00379486	0.00188534	0.00118885	5.09E-37	0.00140001	0.000481	161.628091
Sitting height	Falls	rs76291069	16	T	C	0.014232	-0.00151123	0.00268303	0.00169465	1.13E-07	0.37	8.37E-05	28.1371958
Sitting height	Falls	rs763665	16	T	C	-0.0163453	0.00266405	0.00257134	0.00162033	2.06E-10	0.1	0.00012	40.40794649
Sitting height	Falls	rs933717	16	C	T	-0.0108669	-0.00144185	0.00190848	0.00119903	1.24E-08	0.23	9.64E-05	32.42172479

Sitting height	Falls	rs9745989	16	C	T	0.0167698	3.94E-07	0.00198673	0.0012536	3.16E-17	1	0.000212	71.2488841
Sitting height	Falls	rs9925261	16	C	A	0.0115548	-0.00130931	0.0020042	0.00126373	8.16E-09	0.3	9.89E-05	33.23860205
Sitting height	Falls	rs1043515	17	G	A	0.0179432	-4.68E-05	0.00189179	0.00119219	2.44E-21	0.97	0.000268	89.96093176
Sitting height	Falls	rs10468467	17	G	A	0.0152186	0.000175441	0.00235815	0.00148086	1.09E-10	0.91	0.000124	41.64918919
Sitting height	Falls	rs113886615	17	T	C	0.027186	-0.00139424	0.00384069	0.00242378	1.46E-12	0.57	0.000149	50.10396737
Sitting height	Falls	rs11654212	17	A	G	0.0137305	-0.00114588	0.00245167	0.00154678	2.14E-08	0.46	9.33E-05	31.36524464
Sitting height	Falls	rs11655860	17	A	G	0.0101239	0.000220147	0.00197949	0.00124866	3.15E-07	0.86	7.78E-05	26.15706847
Sitting height	Falls	rs11658342	17	A	G	-0.0120588	-0.00129095	0.0019348	0.00122115	4.59E-10	0.29	0.000116	38.84508062
Sitting height	Falls	rs12452505	17	G	C	-0.0261887	0.00224084	0.00270954	0.00169865	4.26E-22	0.19	0.000278	93.41932994
Sitting height	Falls	rs12951873	17	C	T	-0.0115248	0.000139996	0.00188523	0.00118891	9.78E-10	0.91	0.000111	37.37129183
Sitting height	Falls	rs1317867	17	G	A	0.0135436	-0.00106068	0.00189476	0.00119373	8.83E-13	0.37	0.000152	51.0928139
Sitting height	Falls	rs16961974	17	C	T	-0.0107984	0.00181008	0.00214478	0.00134916	4.79E-07	0.18	7.54E-05	25.34856153
Sitting height	Falls	rs17690703	17	T	C	-0.0239988	0.00437686	0.00212064	0.00133993	1.10E-29	0.00109999	0.000381	128.0693352
Sitting height	Falls	rs178793	17	G	C	0.00965302	0.0019964	0.00188176	0.00118667	2.90E-07	0.0929994	7.83E-05	26.31467113
Sitting height	Falls	rs187187134	17	A	G	0.0446475	0.00628248	0.00746254	0.00476345	2.19E-09	0.19	0.000106	35.79488305
Sitting height	Falls	rs2532111	17	G	A	0.0314412	-0.00068188	0.00196956	0.00124123	2.41E-57	0.58	0.000757	254.8354224
Sitting height	Falls	rs35344256	17	A	C	-0.0202699	-0.000749068	0.00204009	0.00128508	2.93E-23	0.56	0.000294	98.71986622
Sitting height	Falls	rs35643058	17	G	A	0.0105189	0.000721715	0.00209226	0.00132027	4.97E-07	0.58	7.52E-05	25.2760583
Sitting height	Falls	rs4625755	17	G	C	-0.0147977	-0.00100497	0.001915	0.00120567	1.10E-14	0.4	0.000178	59.7105237
Sitting height	Falls	rs4794006	17	A	G	-0.0197584	0.000844819	0.00188949	0.00119015	1.37E-25	0.48	0.000325	109.348883
Sitting height	Falls	rs4925137	17	G	A	-0.013233	0.00180954	0.00262117	0.00164295	4.46E-07	0.27	7.58E-05	25.48744183
Sitting height	Falls	rs55749333	17	T	C	-0.0242553	0.00187232	0.00195544	0.00123213	2.53E-35	0.13	0.000457	153.8594982
Sitting height	Falls	rs62060660	17	A	G	-0.0140877	0.00221445	0.00274718	0.00173319	2.93E-07	0.2	7.82E-05	26.29698491
Sitting height	Falls	rs7220854	17	C	T	0.0192095	-0.000190861	0.00198077	0.00124863	3.10E-22	0.88	0.00028	94.05113095
Sitting height	Falls	rs7223535	17	A	G	-0.0173744	0.000143007	0.00211319	0.00133189	2.01E-16	0.91	0.000201	67.59935278
Sitting height	Falls	rs9907628	17	T	C	0.0140753	-0.000440423	0.00228241	0.00143972	6.98E-10	0.760001	0.000113	38.03014641

Sitting height	Falls	rs113476837	18	C	A	-0.0306787	0.000393335	0.00485572	0.00306625	2.65E-10	0.9	0.000119	39.91780331
Sitting height	Falls	rs11660869	18	A	G	-0.0153739	-0.00235356	0.00247159	0.00155005	4.97E-10	0.13	0.000115	38.69147132
Sitting height	Falls	rs17647041	18	A	G	0.0121762	0.000597137	0.0023854	0.00150622	3.32E-07	0.69	7.75E-05	26.05560226
Sitting height	Falls	rs17700144	18	A	G	0.0144533	0.00225329	0.00228978	0.00144732	2.76E-10	0.12	0.000119	39.84249428
Sitting height	Falls	rs2042717	18	T	C	0.0102291	-0.0014477	0.00190725	0.00120386	8.18E-08	0.23	8.56E-05	28.76468361
Sitting height	Falls	rs2347808	18	A	G	-0.0112424	-0.000626434	0.00187708	0.00118366	2.11E-09	0.6	0.000107	35.87174484
Sitting height	Falls	rs35054365	18	A	T	0.0329436	-0.00101674	0.00189065	0.00119246	5.76E-68	0.39	0.000902	303.6126302
Sitting height	Falls	rs4350646	18	C	T	-0.0110491	0.00316577	0.0019314	0.00121618	1.06E-08	0.00920005	9.73E-05	32.72723796
Sitting height	Falls	rs4377239	18	T	C	-0.0147649	-0.00384491	0.00287771	0.00180812	2.89E-07	0.0329997	7.83E-05	26.32491441
Sitting height	Falls	rs4800451	18	T	C	0.0224683	0.00329316	0.00215324	0.00135919	1.74E-25	0.015	0.000324	108.8818647
Sitting height	Falls	rs4939837	18	G	A	-0.014624	-0.00097614	0.00197873	0.00124905	1.46E-13	0.43	0.000162	54.62095388
Sitting height	Falls	rs6508220	18	G	A	-0.0178291	-0.00211961	0.00188123	0.001186	2.62E-21	0.0739997	0.000267	89.8204135
Sitting height	Falls	rs7239515	18	C	G	0.0135735	-0.00120319	0.00194882	0.00122832	3.29E-12	0.33	0.000144	48.51100127
Sitting height	Falls	rs10401891	19	T	C	-0.0180709	9.49E-05	0.00200355	0.0012655	1.90E-19	0.94	0.000242	81.35030681
Sitting height	Falls	rs10948	19	T	G	-0.0178191	0.000559328	0.0019885	0.0012525	3.23E-19	0.66	0.000239	80.30088645
Sitting height	Falls	rs113540008	19	T	C	-0.0189347	0.0037152	0.0036387	0.00231088	1.95E-07	0.11	8.05E-05	27.07848457
Sitting height	Falls	rs116843977	19	G	A	0.038102	-0.00693799	0.00570416	0.0036015	2.40E-11	0.0539995	0.000133	44.61821046
Sitting height	Falls	rs12459155	19	G	C	-0.0145278	-0.00219219	0.00279353	0.00175861	1.99E-07	0.21	8.04E-05	27.04537612
Sitting height	Falls	rs12981072	19	G	C	0.0112398	0.000992464	0.0019026	0.00119839	3.47E-09	0.41	0.000104	34.89973925
Sitting height	Falls	rs12982509	19	C	T	0.0147801	0.001429	0.0022593	0.00142865	6.08E-11	0.32	0.000127	42.79637065
Sitting height	Falls	rs12985850	19	A	G	0.0223717	0.000972265	0.00192313	0.00121271	2.84E-31	0.42	0.000402	135.3258265
Sitting height	Falls	rs147110934	19	T	G	-0.0577736	0.00592451	0.00611595	0.00383698	3.53E-21	0.12	0.000265	89.23413291
Sitting height	Falls	rs16975663	19	G	A	-0.0181199	0.000397235	0.00292187	0.00183433	5.60E-10	0.83	0.000114	38.45827622
Sitting height	Falls	rs17318596	19	A	G	0.017309	-0.000962033	0.00195195	0.00123038	7.51E-19	0.43	0.000234	78.63331378
Sitting height	Falls	rs34831515	19	T	C	-0.0165673	0.00116276	0.00222318	0.00140409	9.21E-14	0.41	0.000165	55.53339433
Sitting height	Falls	rs35296670	19	A	G	0.0118583	0.00137229	0.00222773	0.00140416	1.02E-07	0.33	8.43E-05	28.33477444

Sitting height	Falls	rs4252548	19	T	C	-0.0526816	-0.00124939	0.00648822	0.00403478	4.69E-16	0.760001	0.000196	65.92752595
Sitting height	Falls	rs6508712	19	G	T	-0.00979216	0.00247492	0.00190902	0.00120317	2.91E-07	0.04	7.83E-05	26.31092069
Sitting height	Falls	rs7252363	19	A	G	-0.0156324	0.00114332	0.00196417	0.00123762	1.74E-15	0.36	0.000188	63.34220411
Sitting height	Falls	rs73030677	19	C	T	-0.0167707	0.00375767	0.00306805	0.00193723	4.60E-08	0.0519996	8.89E-05	29.87978818
Sitting height	Falls	rs8103992	19	C	A	-0.0201294	-0.00055745	0.00235657	0.00148158	1.33E-17	0.709999	0.000217	72.96271242
Sitting height	Falls	rs13037942	20	C	T	0.0232566	0.00206917	0.00218591	0.00138238	1.98E-26	0.13	0.000337	113.1951692
Sitting height	Falls	rs143384	20	G	A	0.0646491	0.000522578	0.00190903	0.00120406	1.00E-200	0.66	0.0034	1146.831096
Sitting height	Falls	rs151123887	20	A	G	-0.0391601	-0.0031643	0.00665179	0.00416929	3.93E-09	0.450001	0.000103	34.65856079
Sitting height	Falls	rs201911727	20	G	A	0.0339749	0.000928164	0.0057652	0.00356457	3.79E-09	0.79	0.000103	34.72862736
Sitting height	Falls	rs2650965	20	G	A	-0.0234282	-0.00218318	0.00200448	0.00126286	1.49E-31	0.0840001	0.000406	136.607452
Sitting height	Falls	rs34919557	20	T	C	-0.0179808	0.00446056	0.00325236	0.00204317	3.23E-08	0.0290001	9.09E-05	30.56474653
Sitting height	Falls	rs4812056	20	C	A	0.012989	0.00123756	0.00219077	0.00138438	3.05E-09	0.37	0.000105	35.15263326
Sitting height	Falls	rs55990870	20	T	C	-0.0274548	0.00106223	0.00213857	0.00134503	1.03E-37	0.43	0.00049	164.8123031
Sitting height	Falls	rs6021385	20	C	G	-0.011258	0.00134087	0.00211595	0.00133602	1.04E-07	0.32	8.42E-05	28.30816292
Sitting height	Falls	rs6026578	20	G	C	-0.0126855	-0.00120223	0.00194775	0.00122823	7.38E-11	0.33	0.000126	42.41785989
Sitting height	Falls	rs6052358	20	C	T	0.0138324	-0.00150892	0.00188418	0.00118809	2.12E-13	0.2	0.00016	53.89522623
Sitting height	Falls	rs6060983	20	C	T	0.0186576	-0.00394821	0.0020517	0.00129312	9.62E-20	0.00230001	0.000246	82.69587353
Sitting height	Falls	rs6066104	20	T	C	0.0185704	-0.000885714	0.00200255	0.00126514	1.81E-20	0.48	0.000256	85.99551069
Sitting height	Falls	rs6082358	20	T	C	-0.0150089	-0.00216065	0.00201582	0.00127105	9.68E-14	0.089	0.000165	55.43629901
Sitting height	Falls	rs6106241	20	C	T	0.0124388	-0.000355097	0.00200914	0.00126646	5.98E-10	0.780001	0.000114	38.32980146
Sitting height	Falls	rs6140015	20	G	A	-0.0197431	-0.000195117	0.00191395	0.00120804	6.06E-25	0.87	0.000316	106.4068322
Sitting height	Falls	rs742698	20	C	T	-0.0105046	-0.00154347	0.00205093	0.0012934	3.03E-07	0.23	7.80E-05	26.23356808
Sitting height	Falls	rs747680	20	C	T	0.0112107	0.00153317	0.00187639	0.00118163	2.31E-09	0.19	0.000106	35.69597447
Sitting height	Falls	rs1043801	21	A	G	0.0248284	-0.00505052	0.00400424	0.00253887	5.63E-10	0.0470002	0.000114	38.44654055
Sitting height	Falls	rs162355	21	G	A	0.0120289	-0.00108897	0.0023389	0.00147193	2.71E-07	0.46	7.87E-05	26.45017326
Sitting height	Falls	rs56165045	21	A	G	-0.0162716	0.00200453	0.00272443	0.00170385	2.34E-09	0.24	0.000106	35.67050119

Sitting height	Falls	rs6586286	21	G	T	-0.0126441	-0.0015888	0.00188779	0.00119026	2.12E-11	0.18	0.000133	44.86095158
Sitting height	Falls	rs73189390	21	A	G	-0.012518	-0.00174196	0.00243447	0.0015334	2.72E-07	0.26	7.86E-05	26.439975
Sitting height	Falls	rs8127309	21	G	A	-0.0175605	0.00173202	0.00201445	0.00126968	2.86E-18	0.17	0.000226	75.99075686
Sitting height	Falls	rs2256609	22	G	A	-0.0192116	-0.00122668	0.00239012	0.00150936	9.17E-16	0.42	0.000192	64.60820265
Sitting height	Falls	rs2285179	22	A	G	0.0119919	6.87E-05	0.00189017	0.00119174	2.24E-10	0.95	0.00012	40.25077666
Sitting height	Falls	rs28575622	22	T	G	0.0204977	-0.00114389	0.00390437	0.00244695	1.52E-07	0.64	8.20E-05	27.56184785
Sitting height	Falls	rs429940	22	G	A	0.0102362	-0.00118871	0.00195469	0.00123218	1.64E-07	0.33	8.16E-05	27.42342809
Sitting height	Falls	rs55906059	22	T	C	-0.0296511	0.00184839	0.00323364	0.00205033	4.77E-20	0.37	0.00025	84.08108014
Sitting height	Falls	rs5759002	22	G	A	-0.0114653	-0.000788512	0.00188252	0.00118595	1.13E-09	0.51	0.00011	37.0929734
Sitting height	Falls	rs74385072	22	C	T	-0.0807723	0.010368	0.00763258	0.00482676	3.62E-26	0.032	0.000333	111.9907542
Hip circumference	Falls	rs10493979	1	G	T	-0.0139097	6.82293E-05	0.00262114	0.00128865	1.11681E-07	0.96	8.37E-05	38.62991727
Hip circumference	Falls	rs10493988	1	G	A	0.0132618	-0.00242925	0.002503	0.00123113	1.16926E-07	0.0479999	8.34E-05	38.50808629
Hip circumference	Falls	rs112646560	1	T	C	0.0208306	0.00178907	0.00294004	0.00144486	1.39187E-12	0.22	0.000149	68.85980175
Hip circumference	Falls	rs1127100	1	C	T	0.0149329	0.00102927	0.00252469	0.00123935	3.32683E-09	0.41	0.000104	47.98890951
Hip circumference	Falls	rs11584359	1	T	C	-0.0211394	-0.00101457	0.0031669	0.00155774	2.474E-11	0.51	0.000132	61.12027512
Hip circumference	Falls	rs11803990	1	G	C	0.0252396	0.000226788	0.00438591	0.00217669	8.6868E-09	0.92	9.84E-05	45.42701412
Hip circumference	Falls	rs12096864	1	C	T	0.0219612	0.00245922	0.00376456	0.00185134	5.42713E-09	0.18	0.000101	46.68231392
Hip circumference	Falls	rs12097230	1	A	G	-0.0128953	-0.00364165	0.00242728	0.00119398	1.08126E-07	0.00230001	8.38E-05	38.71612049
Hip circumference	Falls	rs12140153	1	T	G	-0.0248568	-0.000863235	0.00417296	0.00207595	2.57751E-09	0.68	0.000105	48.67104598
Hip circumference	Falls	rs1229128	1	A	G	0.0158537	-0.000626894	0.00304025	0.00149518	1.84327E-07	0.68	8.08E-05	37.30021754
Hip circumference	Falls	rs12561919	1	T	C	0.0200114	0.00328149	0.00338817	0.00167364	3.50268E-09	0.05	0.000104	47.85124567
Hip circumference	Falls	rs16825336	1	A	G	0.0213158	-0.000744156	0.00414967	0.00204929	2.79679E-07	0.719999	7.84E-05	36.19468811
Hip circumference	Falls	rs17024393	1	C	T	0.0568069	0.00354916	0.0076056	0.00373283	8.09469E-14	0.34	0.000166	76.5251
Hip circumference	Falls	rs2678204	1	G	T	0.0216541	0.000439715	0.00253869	0.00124922	1.47367E-17	0.719999	0.000216	99.79978803
Hip circumference	Falls	rs2761185	1	A	G	-0.0318384	-0.00287873	0.00611646	0.0029527	1.93731E-07	0.33	8.05E-05	37.16814416
Hip circumference	Falls	rs2802774	1	A	C	0.016994	0.00183197	0.00245316	0.00120687	4.29438E-12	0.13	0.000143	65.82762188

Hip circumference	Falls	rs2815753	1	A	G	0.0218333	0.00323345	0.00245234	0.00120802	5.46009E-19	0.00739997	0.000235	108.7291738
Hip circumference	Falls	rs33955687	1	A	C	-0.0160724	0.00177732	0.00270852	0.00133322	2.9591E-09	0.18	0.000105	48.30210508
Hip circumference	Falls	rs34517439	1	A	C	0.0429378	0.0029921	0.00368662	0.00183027	2.41268E-31	0.1	0.000403	186.0764943
Hip circumference	Falls	rs3845344	1	T	C	0.0146464	0.00222827	0.00246158	0.00121191	2.68448E-09	0.0659994	0.000105	48.56267034
Hip circumference	Falls	rs4471313	1	T	G	-0.0170096	0.000109112	0.00272413	0.00133685	4.26933E-10	0.93	0.000116	53.4812108
Hip circumference	Falls	rs4660586	1	T	C	-0.019614	-0.00039832	0.00274699	0.00135154	9.34114E-13	0.77	0.000151	69.93375898
Hip circumference	Falls	rs4908677	1	T	C	0.0132201	0.00118633	0.00242907	0.00119468	5.25872E-08	0.32	8.8E-05	40.63105577
Hip circumference	Falls	rs543874	1	G	A	0.0442781	-0.00160565	0.00296152	0.00146521	1.58928E-50	0.27	0.000664	306.6318767
Hip circumference	Falls	rs588660	1	A	G	0.0188173	-0.00135347	0.00244358	0.0012009	1.35644E-14	0.26	0.000176	81.34487221
Hip circumference	Falls	rs60226453	1	T	C	0.0177113	0.00158374	0.00316301	0.00154783	2.15105E-08	0.31	9.31E-05	43.00990033
Hip circumference	Falls	rs6669341	1	G	A	-0.0146303	0.000620792	0.00244057	0.00119953	2.04183E-09	0.6	0.000107	49.29383573
Hip circumference	Falls	rs7516554	1	T	C	0.0155427	0.00182512	0.00245713	0.00120961	2.52604E-10	0.13	0.000119	54.88647598
Hip circumference	Falls	rs7548408	1	C	T	0.0150333	0.00385425	0.00243163	0.0011955	6.32033E-10	0.00129999	0.000114	52.4303009
Hip circumference	Falls	rs76798800	1	T	G	0.0254141	-0.000385192	0.0027214	0.00134096	9.81522E-21	0.77	0.000259	119.6281524
Hip circumference	Falls	rs815335	1	T	C	0.0184289	0.0025805	0.00250163	0.00123201	1.75227E-13	0.0359998	0.000161	74.4425706
Hip circumference	Falls	rs9424466	1	C	A	0.0138768	0.000795216	0.00269806	0.00132839	2.70197E-07	0.55	7.86E-05	36.28642131
Hip circumference	Falls	rs1014291	2	T	G	-0.0160298	0.00375212	0.00243785	0.0011992	4.85959E-11	0.00179999	0.000128	59.30767988
Hip circumference	Falls	rs10210468	2	C	T	-0.0150017	0.000260515	0.00244268	0.00120176	8.18502E-10	0.83	0.000112	51.73881611
Hip circumference	Falls	rs1118151	2	G	T	0.016606	-0.00134107	0.00269097	0.00132342	6.79297E-10	0.31	0.000113	52.23733861
Hip circumference	Falls	rs11688707	2	A	G	0.0130416	-0.000705711	0.00252167	0.0012421	2.31953E-07	0.57	7.95E-05	36.6905258
Hip circumference	Falls	rs12467963	2	T	A	-0.013934	0.000665948	0.00247214	0.00121619	1.73796E-08	0.58	9.44E-05	43.57869055
Hip circumference	Falls	rs12475388	2	A	G	-0.0134418	-0.00126777	0.00241781	0.00119023	2.70763E-08	0.29	9.18E-05	42.39739829
Hip circumference	Falls	rs12619178	2	T	C	-0.0158059	-0.00251479	0.00245114	0.00120658	1.13191E-10	0.0369999	0.000124	57.03886996
Hip circumference	Falls	rs13389219	2	T	C	0.0263086	0.0010595	0.0024607	0.0012107	1.12642E-26	0.38	0.000339	156.8002767
Hip circumference	Falls	rs13410783	2	G	A	0.0146689	-0.0016733	0.00249694	0.00122607	4.23838E-09	0.17	0.000103	47.34210562
Hip circumference	Falls	rs1528450	2	C	T	0.0172654	0.00116123	0.0024864	0.00122357	3.82032E-12	0.34	0.000143	66.14240496

Hip circumference	Falls	rs2193618	2	C	T	0.0133907	0.000997745	0.00247204	0.00121604	6.07002E-08	0.41	8.72E-05	40.24984948
Hip circumference	Falls	rs2244786	2	A	G	0.0139551	-0.000577712	0.00251229	0.001237	2.78221E-08	0.64	9.17E-05	42.32481358
Hip circumference	Falls	rs2693823	2	A	T	-0.0150177	-0.00172948	0.00276473	0.00135431	5.57995E-08	0.2	8.76E-05	40.47343699
Hip circumference	Falls	rs2861690	2	G	C	-0.0171381	-0.00319532	0.00246592	0.00121247	3.66016E-12	0.00840001	0.000143	66.25765582
Hip circumference	Falls	rs34168749	2	T	C	-0.0125537	-0.00103627	0.0024143	0.00118809	1.99678E-07	0.38	8.03E-05	37.08768371
Hip circumference	Falls	rs35882248	2	T	C	0.0170391	4.52964E-05	0.00259225	0.00127238	4.93515E-11	0.97	0.000128	59.26635588
Hip circumference	Falls	rs4430895	2	T	C	0.0241932	-1.41813E-05	0.00240643	0.00118432	8.92894E-24	0.99	0.0003	138.6464814
Hip circumference	Falls	rs4482463	2	A	C	-0.029946	0.00181299	0.00455065	0.00222627	4.69245E-11	0.42	0.000129	59.40178004
Hip circumference	Falls	rs4670612	2	G	A	0.0163808	-0.00223043	0.00253152	0.0012477	9.76562E-11	0.0739997	0.000124	57.43494076
Hip circumference	Falls	rs4832298	2	T	C	-0.013755	-0.00197573	0.00259071	0.00127453	1.10073E-07	0.12	8.37E-05	38.66805021
Hip circumference	Falls	rs58584712	2	A	G	0.0198104	-0.000030239	0.00295089	0.00144957	1.90458E-11	0.98	0.000134	61.82286615
Hip circumference	Falls	rs62166769	2	A	T	0.0127581	-0.00099719	0.00249432	0.00122777	3.14152E-07	0.42	7.77E-05	35.88693377
Hip circumference	Falls	rs62183012	2	C	T	-0.0134016	-0.0019205	0.00265696	0.00130821	4.56215E-07	0.14	7.56E-05	34.89891761
Hip circumference	Falls	rs6437277	2	G	A	-0.0150561	0.000508271	0.00286809	0.00141182	1.52584E-07	0.719999	8.19E-05	37.80142033
Hip circumference	Falls	rs6707036	2	G	A	-0.0146292	-0.00140835	0.00255395	0.00125817	1.01653E-08	0.26	9.75E-05	45.00751769
Hip circumference	Falls	rs6739755	2	G	A	-0.0152363	-0.00421612	0.00246322	0.00121231	6.19669E-10	0.00051	0.000114	52.48332165
Hip circumference	Falls	rs6747657	2	A	G	0.0140974	-0.00123874	0.00266546	0.00131376	1.23132E-07	0.35	8.31E-05	38.37093602
Hip circumference	Falls	rs72917533	2	C	T	-0.0164346	-0.00355141	0.00309747	0.00152651	1.12241E-07	0.02	8.36E-05	38.61648554
Hip circumference	Falls	rs75543804	2	T	G	-0.040796	0.00436358	0.00679915	0.00336616	1.9731E-09	0.19	0.000107	49.38494889
Hip circumference	Falls	rs968379	2	T	C	-0.0206395	0.0011668	0.00286037	0.00141217	5.37898E-13	0.41	0.000155	71.42045337
Hip circumference	Falls	rs11915747	3	G	C	-0.0187962	-0.00024281	0.00251523	0.00123818	7.85959E-14	0.84	0.000166	76.60434325
Hip circumference	Falls	rs13085031	3	T	C	0.0131865	-0.00335806	0.00246501	0.00121213	8.82633E-08	0.00560003	8.5E-05	39.25458539
Hip circumference	Falls	rs1406779	3	T	C	0.0201178	0.00319796	0.00258241	0.00126837	6.70348E-15	0.012	0.00018	83.24904697
Hip circumference	Falls	rs1727901	3	T	C	0.0188821	0.00298037	0.00272522	0.00134357	4.25598E-12	0.0269998	0.000143	65.85157082
Hip circumference	Falls	rs2034768	3	G	A	-0.016569	-0.00177927	0.002406	0.001183	5.72532E-12	0.13	0.000141	65.05340561
Hip circumference	Falls	rs2270894	3	G	C	-0.0199916	0.00191666	0.00309373	0.00152642	1.03474E-10	0.21	0.000124	57.27946821

Hip circumference	Falls	rs2371767	3	C	G	0.0198003	0.00015889	0.00270203	0.00131908	2.34261E-13	0.9	0.00016	73.66003063
Hip circumference	Falls	rs34373881	3	A	G	-0.0149443	-0.00107311	0.00268972	0.00132754	2.76096E-08	0.42	9.17E-05	42.34534712
Hip circumference	Falls	rs35779991	3	C	T	0.0143334	0.00150266	0.00242195	0.00119226	3.25949E-09	0.21	0.000104	48.04373722
Hip circumference	Falls	rs4017425	3	T	C	-0.0138473	-0.00153602	0.00241466	0.00118716	9.77822E-09	0.2	9.77E-05	45.11146324
Hip circumference	Falls	rs55932154	3	G	A	-0.0236291	-0.00528765	0.0037729	0.00185795	3.7846E-10	0.00439997	0.000117	53.80373921
Hip circumference	Falls	rs62243489	3	G	T	-0.0153338	-0.00317627	0.00276351	0.00135677	2.88058E-08	0.0189998	9.15E-05	42.2324421
Hip circumference	Falls	rs62246314	3	A	G	0.0247126	0.00223832	0.00398491	0.0019499	5.59783E-10	0.25	0.000114	52.75559583
Hip circumference	Falls	rs6810023	3	A	G	0.0170797	0.00611795	0.00335675	0.00165503	3.61701E-07	0.000219999	7.69E-05	35.51331132
Hip circumference	Falls	rs724016	3	G	A	0.0264891	-0.000674729	0.0024177	0.00118973	6.26325E-28	0.57	0.000357	164.6638525
Hip circumference	Falls	rs73175572	3	G	A	0.0264617	0.000685891	0.00385671	0.00189774	6.84069E-12	0.719999	0.00014	64.57586057
Hip circumference	Falls	rs7426945	3	G	A	0.0156387	-0.00149055	0.0024133	0.00118868	9.17065E-11	0.21	0.000125	57.60329928
Hip circumference	Falls	rs7610647	3	G	A	-0.0139905	-0.000409971	0.00265614	0.00130484	1.38596E-07	0.75	8.24E-05	38.05688473
Hip circumference	Falls	rs79375047	3	T	C	0.0158983	0.00241976	0.00309756	0.0015308	2.86036E-07	0.11	7.83E-05	36.1352121
Hip circumference	Falls	rs79597869	3	G	A	0.0122375	0.00107665	0.00240601	0.00118364	3.6546E-07	0.36	7.69E-05	35.48617916
Hip circumference	Falls	rs8192675	3	C	T	0.0172691	0.00311706	0.00265297	0.0013038	7.55962E-11	0.017	0.000126	58.12238384
Hip circumference	Falls	rs838204	3	G	A	0.013251	0.000790573	0.00254985	0.00125564	2.02922E-07	0.53	8.02E-05	37.04560822
Hip circumference	Falls	rs869400	3	G	T	0.0196495	0.00351102	0.00311102	0.00152583	2.6859E-10	0.021	0.000119	54.72252002
Hip circumference	Falls	rs9808900	3	T	G	0.0269504	0.00013044	0.00301663	0.00148239	4.13048E-19	0.93	0.000237	109.4851019
Hip circumference	Falls	rs9814633	3	A	G	0.0147487	0.00140985	0.00253421	0.00124532	5.89522E-09	0.26	0.000101	46.46125861
Hip circumference	Falls	rs9843653	3	C	T	0.0205097	0.00235581	0.00240714	0.00118412	1.59698E-17	0.0470002	0.000216	99.58286201
Hip circumference	Falls	rs10938397	4	G	A	0.0231136	0.00114437	0.002432	0.00119576	2.03283E-21	0.34	0.000268	123.901548
Hip circumference	Falls	rs1296328	4	C	A	-0.0155122	0.00136134	0.00243358	0.00119712	1.84128E-10	0.26	0.000121	55.73451462
Hip circumference	Falls	rs13107325	4	T	C	0.042561	0.0066067	0.00457779	0.00224919	1.44877E-20	0.00329997	0.000257	118.5715787
Hip circumference	Falls	rs13151185	4	T	G	-0.0136099	-0.00153509	0.00241049	0.00118551	1.64263E-08	0.2	9.47E-05	43.72883301
Hip circumference	Falls	rs2102278	4	G	A	0.0158475	9.05228E-05	0.00257945	0.00126723	8.06845E-10	0.94	0.000112	51.77689234
Hip circumference	Falls	rs2318543	4	G	A	-0.0179454	-0.00211337	0.00292494	0.0014398	8.50805E-10	0.14	0.000112	51.63462773

Hip circumference	Falls	rs28418580	4	T	C	-0.0174415	-0.0036365	0.00242792	0.00119302	6.79829E-13	0.00230001	0.000153	70.78929819
Hip circumference	Falls	rs34049648	4	A	G	0.0176063	0.00244449	0.00254462	0.00125237	4.55512E-12	0.051	0.000142	65.6687894
Hip circumference	Falls	rs34811474	4	A	G	-0.0205406	-0.00458518	0.00284677	0.0014043	5.3889E-13	0.00109999	0.000155	71.41512114
Hip circumference	Falls	rs4240326	4	G	A	-0.0278879	0.00158589	0.00241489	0.00118844	7.62781E-31	0.18	0.000396	182.9387074
Hip circumference	Falls	rs6535240	4	G	A	0.0144793	0.0027261	0.00259902	0.00127903	2.5339E-08	0.0329997	9.22E-05	42.57401191
Hip circumference	Falls	rs66679256	4	T	C	0.015685	0.00337649	0.00242434	0.00119152	9.82653E-11	0.00460002	0.000124	57.41834616
Hip circumference	Falls	rs6821305	4	C	A	0.0150058	0.000195601	0.00245839	0.00120733	1.03605E-09	0.87	0.000111	51.10759371
Hip circumference	Falls	rs6837528	4	A	G	-0.0134485	-0.00175828	0.00266145	0.00130872	4.34961E-07	0.18	7.59E-05	35.02513023
Hip circumference	Falls	rs6840236	4	C	T	0.0166995	0.0028093	0.00241801	0.00118815	4.98425E-12	0.0179999	0.000142	65.42736626
Hip circumference	Falls	rs73213484	4	T	A	-0.0195843	-0.00550756	0.00347756	0.00170084	1.78665E-08	0.0012	9.42E-05	43.50463509
Hip circumference	Falls	rs750090	4	C	T	-0.0169135	-0.00244347	0.00253756	0.00124803	2.64606E-11	0.05	0.000132	60.94008045
Hip circumference	Falls	rs11745618	5	G	A	0.0125425	-0.00091257	0.00243148	0.00119683	2.49218E-07	0.450001	7.9E-05	36.5002218
Hip circumference	Falls	rs12519997	5	A	G	-0.014272	-0.0041332	0.00242205	0.00119061	3.8075E-09	0.000519996	0.000103	47.62907561
Hip circumference	Falls	rs1428120	5	T	G	-0.0136416	-0.000530624	0.0024288	0.0011945	1.94877E-08	0.66	9.37E-05	43.27288005
Hip circumference	Falls	rs1477290	5	C	T	0.0281297	0.00404939	0.00354094	0.00173547	1.96155E-15	0.02	0.000187	86.5688708
Hip circumference	Falls	rs1582931	5	A	G	-0.0196312	-0.00450649	0.00242829	0.00119488	6.27047E-16	0.00016	0.000194	89.65226782
Hip circumference	Falls	rs185299	5	A	G	0.0144078	1.92559E-05	0.00271402	0.0013346	1.10502E-07	0.99	8.37E-05	38.65787189
Hip circumference	Falls	rs2307111	5	C	T	-0.0282307	-0.00153717	0.00246445	0.00121084	2.2444E-30	0.2	0.00039	179.9997734
Hip circumference	Falls	rs252749	5	A	G	-0.0230008	-0.00108435	0.00278435	0.0013715	1.45278E-16	0.43	0.000203	93.60669636
Hip circumference	Falls	rs33967909	5	A	G	0.0154853	0.00115869	0.00293324	0.0014381	1.29799E-07	0.42	8.28E-05	38.23074285
Hip circumference	Falls	rs34629844	5	G	A	0.0219304	0.000771263	0.00358667	0.00176781	9.70175E-10	0.66	0.000111	51.28365301
Hip circumference	Falls	rs3811951	5	G	A	0.0146228	-0.000252313	0.00266992	0.00131364	4.33251E-08	0.85	8.91E-05	41.14653516
Hip circumference	Falls	rs3943933	5	A	T	0.0132294	0.00208187	0.00240628	0.00118338	3.84698E-08	0.0790005	8.98E-05	41.46261225
Hip circumference	Falls	rs40071	5	C	T	-0.0174246	0.00109749	0.00314153	0.00154604	2.91622E-08	0.48	9.14E-05	42.19994637
Hip circumference	Falls	rs4073717	5	T	G	-0.0175965	-0.0012648	0.00300343	0.00147631	4.66702E-09	0.39	0.000102	47.08539805
Hip circumference	Falls	rs4267859	5	A	C	-0.0130751	0.0011551	0.00248944	0.00122589	1.50359E-07	0.35	8.19E-05	37.84037213

Hip circumference	Falls	rs4866585	5	G	T	0.0134649	-0.000506282	0.0024895	0.00122613	6.3536E-08	0.68	8.69E-05	40.12829347
Hip circumference	Falls	rs4921301	5	T	C	-0.0156418	-0.00235187	0.00298724	0.00146964	1.64002E-07	0.11	8.14E-05	37.60987696
Hip circumference	Falls	rs59738707	5	A	G	-0.0232805	-0.000104668	0.0036192	0.00178024	1.25684E-10	0.95	0.000123	56.75813647
Hip circumference	Falls	rs6867299	5	C	T	0.0205436	0.000774973	0.00250483	0.0012329	2.38067E-16	0.53	0.0002	92.27101033
Hip circumference	Falls	rs7442885	5	G	C	-0.020481	-0.0025164	0.00296022	0.00144483	4.56457E-12	0.0819993	0.000142	65.66334846
Hip circumference	Falls	rs74473266	5	T	C	-0.0256909	-0.005145	0.00511014	0.00251806	4.97348E-07	0.0409996	7.51E-05	34.67062956
Hip circumference	Falls	rs75949361	5	T	C	0.0436087	-0.000409116	0.00678949	0.0033783	1.33814E-10	0.9	0.000123	56.59012614
Hip circumference	Falls	rs7703744	5	G	C	0.0143594	0.00089215	0.00271623	0.00133622	1.24733E-07	0.5	8.3E-05	38.33612313
Hip circumference	Falls	rs7714611	5	G	A	-0.0129498	-0.00286002	0.00255816	0.00125965	4.14811E-07	0.0230001	7.61E-05	35.15113898
Hip circumference	Falls	rs9314057	5	C	T	-0.0136589	-0.000127062	0.00241745	0.00118805	1.60469E-08	0.91	9.48E-05	43.79102772
Hip circumference	Falls	rs10947137	6	C	A	-0.0244192	0.0024044	0.00458477	0.00225618	1.00378E-07	0.29	8.43E-05	38.91315498
Hip circumference	Falls	rs12209223	6	A	C	0.0224965	0.00070343	0.00398646	0.00196961	1.67036E-08	0.719999	9.46E-05	43.68413874
Hip circumference	Falls	rs12528644	6	A	C	0.0227005	-0.000101902	0.00267839	0.00131544	2.3518E-17	0.94	0.000213	98.53531375
Hip circumference	Falls	rs1294437	6	T	C	0.0186801	0.000483837	0.00254412	0.00125217	2.10184E-13	0.7	0.00016	73.95233597
Hip circumference	Falls	rs1847912	6	G	A	-0.0150781	-0.000707528	0.00279356	0.00137555	6.76426E-08	0.61	8.65E-05	39.96188069
Hip circumference	Falls	rs2253310	6	G	C	0.0205891	0.00144197	0.00248737	0.00122258	1.2627E-16	0.24	0.000204	93.9858837
Hip circumference	Falls	rs2499468	6	A	C	0.0142064	0.00275746	0.00253046	0.00124275	1.97679E-08	0.0259998	9.36E-05	43.23523376
Hip circumference	Falls	rs2814943	6	A	G	0.0550792	0.00391875	0.00345869	0.00170053	4.46992E-57	0.021	0.000753	347.8729486
Hip circumference	Falls	rs28366156	6	C	T	-0.0295856	-0.0029647	0.00355966	0.00175527	9.4973E-17	0.0909997	0.000205	94.7572423
Hip circumference	Falls	rs390192	6	G	A	-0.0140741	0.000281794	0.00242328	0.00119143	6.33242E-09	0.81	0.0001	46.27034699
Hip circumference	Falls	rs41271299	6	T	C	0.0407137	0.000508806	0.00543493	0.00268132	6.84542E-14	0.85	0.000167	76.97716671
Hip circumference	Falls	rs4467770	6	A	G	0.0166846	0.0010205	0.00271997	0.00133983	8.57314E-10	0.450001	0.000112	51.61453961
Hip circumference	Falls	rs4870057	6	G	A	0.0138845	0.000261918	0.00256765	0.00126472	6.39646E-08	0.84	8.69E-05	40.11044948
Hip circumference	Falls	rs577721086	6	C	T	-0.0699953	-0.00578102	0.00554393	0.00276125	1.55561E-36	0.0359998	0.000473	218.6608187
Hip circumference	Falls	rs62396185	6	C	G	-0.0365089	-0.00324245	0.00275766	0.00134979	5.34072E-40	0.016	0.00052	240.4278341
Hip circumference	Falls	rs62405860	6	C	T	0.0203123	0.00253116	0.00280058	0.00137616	4.0879E-13	0.0659994	0.000156	72.15906973

Hip circumference	Falls	rs62425398	6	A	C	0.023635	0.00148559	0.00390514	0.00193021	1.42955E-09	0.44	0.000109	50.24660053
Hip circumference	Falls	rs675162	6	G	A	0.0179101	0.00100574	0.00241142	0.00118622	1.11173E-13	0.4	0.000164	75.66917372
Hip circumference	Falls	rs6907872	6	T	C	0.0147591	-0.000192916	0.0026284	0.00129384	1.96458E-08	0.88	9.37E-05	43.25193294
Hip circumference	Falls	rs72892910	6	T	G	0.0355509	0.00197831	0.00320796	0.00156957	1.55024E-28	0.21	0.000365	168.4658967
Hip circumference	Falls	rs7740107	6	A	T	-0.0229511	0.00177445	0.002723	0.00134179	3.51156E-17	0.19	0.000211	97.44967951
Hip circumference	Falls	rs854917	6	T	C	-0.0147852	0.00048833	0.0027489	0.00134841	7.51225E-08	0.719999	8.59E-05	39.68306534
Hip circumference	Falls	rs9378684	6	T	C	0.0198865	-0.0011095	0.00302803	0.00149074	5.12507E-11	0.46	0.000128	59.16502458
Hip circumference	Falls	rs9489620	6	C	G	0.01267	0.00174596	0.00241932	0.00119017	1.63283E-07	0.14	8.15E-05	37.62142884
Hip circumference	Falls	rs9496567	6	A	G	0.021977	0.00313794	0.00281121	0.00138133	5.39759E-15	0.0230001	0.000182	83.83375628
Hip circumference	Falls	rs962554	6	C	T	-0.0218562	0.000142633	0.0026826	0.00131477	3.73078E-16	0.91	0.000197	91.05549489
Hip circumference	Falls	rs998584	6	A	C	-0.0211236	-0.00360507	0.00241248	0.00118602	2.03236E-18	0.00239999	0.000228	105.1664258
Hip circumference	Falls	rs10236214	7	T	C	0.0181559	-0.000358321	0.00252278	0.00124041	6.17874E-13	0.77	0.000154	71.04695885
Hip circumference	Falls	rs10237317	7	G	A	0.014219	0.00250055	0.00244995	0.00120537	6.48799E-09	0.0379997	0.0001	46.2053595
Hip circumference	Falls	rs10269774	7	A	G	0.0234509	0.000384677	0.0025724	0.00126172	7.81088E-20	0.760001	0.000247	114.0015392
Hip circumference	Falls	rs11766945	7	A	G	-0.0197938	-0.00338987	0.00302094	0.001478	5.67806E-11	0.0219999	0.000128	58.89017482
Hip circumference	Falls	rs1182199	7	A	C	-0.0279324	-1.77908E-05	0.00261283	0.00128616	1.1392E-26	0.99	0.000339	156.7699735
Hip circumference	Falls	rs12701265	7	A	G	0.0146579	-0.00155376	0.00246003	0.00121005	2.54941E-09	0.2	0.000105	48.70027232
Hip circumference	Falls	rs17149254	7	C	T	-0.0194615	-0.00191999	0.00311973	0.00153071	4.43119E-10	0.21	0.000116	53.38107188
Hip circumference	Falls	rs227940	7	G	T	0.0129923	-0.000411609	0.0024136	0.00118696	7.33263E-08	0.73	8.61E-05	39.74753252
Hip circumference	Falls	rs2289379	7	T	C	-0.0125447	-0.00180169	0.00246821	0.00121436	3.72726E-07	0.14	7.67E-05	35.43439581
Hip circumference	Falls	rs34748838	7	T	C	0.0204746	0.00235282	0.00240761	0.00118328	1.83696E-17	0.0470002	0.000215	99.20356111
Hip circumference	Falls	rs36078773	7	G	T	0.0165822	0.0020892	0.00243452	0.00119855	9.68947E-12	0.0810009	0.000138	63.63943157
Hip circumference	Falls	rs3729793	7	G	C	0.0215897	0.00636085	0.00413262	0.00203107	1.74989E-07	0.0017	8.11E-05	37.43785487
Hip circumference	Falls	rs3807566	7	T	G	-0.0159279	0.00198616	0.00242654	0.00119424	5.24445E-11	0.0959997	0.000128	59.10317595
Hip circumference	Falls	rs4722398	7	T	C	0.0222577	0.00208865	0.00350131	0.00172079	2.0603E-10	0.22	0.00012	55.43296746
Hip circumference	Falls	rs57116196	7	T	C	0.016147	-0.0013364	0.00298851	0.00146182	6.55722E-08	0.36	8.67E-05	40.04450699

Hip circumference	Falls	rs58862095	7	T	C	-0.0188631	0.000192359	0.00244247	0.00120149	1.13972E-14	0.87	0.000177	81.81564251
Hip circumference	Falls	rs6973656	7	G	A	0.0188205	0.0011033	0.00245506	0.00120806	1.7791E-14	0.36	0.000175	80.61331498
Hip circumference	Falls	rs982692	7	C	T	0.0144682	0.00155407	0.00250216	0.00123192	7.37514E-09	0.21	9.93E-05	45.86353656
Hip circumference	Falls	rs10100245	8	A	G	0.0204889	-0.000298038	0.00242571	0.0011939	3.01231E-17	0.8	0.000212	97.86518374
Hip circumference	Falls	rs10103997	8	G	C	0.0162422	-0.000259576	0.00288595	0.00142081	1.82415E-08	0.86	9.41E-05	43.44910052
Hip circumference	Falls	rs113364497	8	T	C	-0.0157612	-3.76398E-06	0.00282326	0.00138674	2.37077E-08	1	9.26E-05	42.75092315
Hip circumference	Falls	rs11997077	8	G	A	-0.0128247	-0.00332511	0.00249347	0.00122603	2.70078E-07	0.00669993	7.86E-05	36.28731394
Hip circumference	Falls	rs12543555	8	G	A	-0.0157172	-0.000908671	0.00297541	0.00146524	1.27603E-07	0.54	8.29E-05	38.27589988
Hip circumference	Falls	rs12680342	8	G	T	-0.0186937	-0.000840056	0.00285757	0.00140574	6.08555E-11	0.55	0.000127	58.70369923
Hip circumference	Falls	rs13264909	8	T	A	-0.0157479	0.000450935	0.00243238	0.00119713	9.54113E-11	0.709999	0.000125	57.49778789
Hip circumference	Falls	rs2737250	8	G	A	-0.0213899	-0.00159222	0.00252036	0.00124277	2.13157E-17	0.2	0.000214	98.80093145
Hip circumference	Falls	rs2954021	8	G	A	0.0177343	0.000403077	0.00240615	0.00118298	1.7053E-13	0.73	0.000161	74.51631903
Hip circumference	Falls	rs310307	8	G	A	-0.0125543	-0.0023368	0.00242099	0.00119141	2.15452E-07	0.05	7.99E-05	36.88652142
Hip circumference	Falls	rs4872142	8	G	C	-0.0206355	0.000671692	0.00309464	0.00152141	2.59478E-11	0.66	0.000132	60.99277747
Hip circumference	Falls	rs6470771	8	C	A	-0.0176853	-0.00280046	0.00320267	0.00157477	3.35305E-08	0.0749998	9.06E-05	41.82819909
Hip circumference	Falls	rs6601527	8	A	C	-0.0155086	-0.00118532	0.00244837	0.00120646	2.38825E-10	0.33	0.000119	55.03763681
Hip circumference	Falls	rs6998644	8	T	C	-0.0128742	0.000232718	0.00242764	0.00119352	1.13865E-07	0.85	8.35E-05	38.57808105
Hip circumference	Falls	rs6999725	8	T	C	-0.0404537	8.21421E-05	0.00670288	0.00329261	1.58866E-09	0.98	0.000108	49.96458649
Hip circumference	Falls	rs72656010	8	C	T	-0.0239918	0.000204483	0.00357594	0.00175298	1.95975E-11	0.91	0.000134	61.74672185
Hip circumference	Falls	rs7460093	8	A	G	0.0150887	0.000229883	0.00243191	0.00119546	5.49402E-10	0.85	0.000114	52.80527855
Hip circumference	Falls	rs77978038	8	G	T	0.0145795	0.00147837	0.00286142	0.00140996	3.48546E-07	0.29	7.71E-05	35.61153805
Hip circumference	Falls	rs7833077	8	C	G	-0.0132735	-0.000596866	0.00253124	0.00124693	1.57329E-07	0.630001	8.17E-05	37.72010961
Hip circumference	Falls	rs7845090	8	A	G	-0.0226011	-4.21028E-05	0.00266783	0.00130879	2.42773E-17	0.97	0.000213	98.44905171
Hip circumference	Falls	rs78565420	8	T	C	0.0276211	0.00417915	0.00546097	0.00271874	4.24121E-07	0.12	7.6E-05	35.0922364
Hip circumference	Falls	rs894347	8	G	A	-0.0182603	0.000111015	0.0024579	0.00120984	1.09471E-13	0.93	0.000164	75.71049486
Hip circumference	Falls	rs10118701	9	G	A	0.0183313	0.00282001	0.00257954	0.00126428	1.19316E-12	0.0259998	0.00015	69.27406675

Hip circumference	Falls	rs10756798	9	T	C	-0.0154182	0.000794226	0.00251451	0.00123386	8.70362E-10	0.52	0.000112	51.57382033
Hip circumference	Falls	rs10820852	9	A	C	-0.0162227	-0.00231264	0.00270056	0.00132442	1.88995E-09	0.0810009	0.000107	49.50023948
Hip circumference	Falls	rs12346647	9	C	T	0.0156951	-0.000857012	0.00302959	0.00148982	2.21294E-07	0.57	7.97E-05	36.81536787
Hip circumference	Falls	rs17770336	9	T	C	0.0205165	-0.0010714	0.00256585	0.00126312	1.28944E-15	0.4	0.00019	87.70264852
Hip circumference	Falls	rs28377268	9	T	G	0.0253898	-0.00305605	0.00388572	0.00191105	6.40767E-11	0.11	0.000127	58.5658218
Hip circumference	Falls	rs4297095	9	A	G	-0.0222453	0.00130876	0.0039494	0.00195734	1.77672E-08	0.5	9.42E-05	43.51940788
Hip circumference	Falls	rs4741546	9	T	C	-0.0177033	-0.00212428	0.00246644	0.00121515	7.10559E-13	0.08	0.000153	70.6701536
Hip circumference	Falls	rs937482	9	G	A	-0.0125853	0.00159168	0.0024067	0.00118421	1.70255E-07	0.18	8.12E-05	37.51041908
Hip circumference	Falls	rs10883553	10	A	C	0.0160261	8.99719E-05	0.00242185	0.00119084	3.66269E-11	0.94	0.00013	60.06616459
Hip circumference	Falls	rs10887571	10	T	C	0.0145648	0.000996061	0.00243391	0.00119843	2.17791E-09	0.41	0.000106	49.12117062
Hip circumference	Falls	rs11146442	10	A	C	-0.0209335	-0.0020266	0.00384937	0.0018972	5.38729E-08	0.29	8.79E-05	40.56701627
Hip circumference	Falls	rs12254441	10	T	C	-0.0149948	-0.00124839	0.00257352	0.00126691	5.66291E-09	0.32	0.000101	46.56878928
Hip circumference	Falls	rs12414412	10	G	C	0.0231133	0.0042181	0.00434374	0.00212182	1.03221E-07	0.0470002	8.41E-05	38.83872422
Hip circumference	Falls	rs12765337	10	C	G	0.0155046	1.03482E-05	0.00253466	0.00124464	9.54333E-10	0.99	0.000111	51.32753383
Hip circumference	Falls	rs12779865	10	C	T	0.0226272	-0.000816762	0.00255912	0.00125805	9.46673E-19	0.52	0.000232	107.2380768
Hip circumference	Falls	rs2439823	10	G	A	0.0188359	-0.000139325	0.00242211	0.00119119	7.46793E-15	0.91	0.00018	82.95712913
Hip circumference	Falls	rs6585201	10	A	G	-0.0208428	-0.00354895	0.00241636	0.00118927	6.39735E-18	0.00280001	0.000221	102.060463
Hip circumference	Falls	rs7087701	10	G	C	-0.0129751	-0.00194815	0.00247335	0.00121681	1.55568E-07	0.11	8.18E-05	37.75017425
Hip circumference	Falls	rs7893571	10	T	G	0.0156277	0.000900371	0.00255052	0.00125721	8.94994E-10	0.47	0.000112	51.49930306
Hip circumference	Falls	rs7915723	10	A	C	-0.0137936	-0.0036004	0.0024247	0.00119298	1.28056E-08	0.0025	9.61E-05	44.39232772
Hip circumference	Falls	rs79969674	10	T	C	0.0265356	0.00404128	0.00462325	0.00227829	9.49839E-09	0.0759994	9.79E-05	45.18889843
Hip circumference	Falls	rs845084	10	A	G	0.0180027	0.000235707	0.00275957	0.00135662	6.8691E-11	0.86	0.000126	58.3796042
Hip circumference	Falls	rs9415106	10	A	G	-0.0151806	-0.00324099	0.00266693	0.00131041	1.25551E-08	0.0129999	9.62E-05	44.44504272
Hip circumference	Falls	rs11030119	11	A	G	0.0314539	0.00388165	0.002604	0.00128331	1.38484E-33	0.0025	0.000433	200.1408459
Hip circumference	Falls	rs12805742	11	T	C	-0.0185191	-0.00134334	0.00285874	0.00140897	9.30251E-11	0.34	0.000125	57.56508153
Hip circumference	Falls	rs140201358	11	G	C	-0.0637486	-0.00158472	0.010272	0.00508251	5.44014E-10	0.760001	0.000114	52.83235491

Hip circumference	Falls	rs143840904	11	T	C	-0.0483109	0.00226169	0.00902762	0.00439211	8.73052E-08	0.61	8.51E-05	39.28370162
Hip circumference	Falls	rs1662185	11	G	A	0.0170127	0.00204043	0.00265128	0.0012997	1.39367E-10	0.12	0.000122	56.48120889
Hip circumference	Falls	rs17245511	11	A	G	-0.0199228	-0.00171946	0.00338136	0.00166402	3.82023E-09	0.3	0.000103	47.61969573
Hip circumference	Falls	rs2187449	11	A	G	0.0163764	7.07777E-05	0.00286336	0.00140925	1.07051E-08	0.96	9.72E-05	44.86975055
Hip circumference	Falls	rs35099456	11	C	G	-0.0406467	0.00132675	0.00498534	0.00246626	3.55468E-16	0.59	0.000197	91.18632363
Hip circumference	Falls	rs4148172	11	G	A	-0.0235908	0.00340607	0.00469185	0.0022872	4.95804E-07	0.14	7.51E-05	34.67893778
Hip circumference	Falls	rs4936671	11	G	C	-0.0127849	0.00118799	0.00252321	0.00124152	4.04511E-07	0.34	7.63E-05	35.21734111
Hip circumference	Falls	rs667515	11	C	G	-0.0139118	-0.00283637	0.00247623	0.00121899	1.93183E-08	0.02	9.38E-05	43.29655846
Hip circumference	Falls	rs7116641	11	G	T	0.0218983	-0.000902552	0.00258946	0.00127391	2.76121E-17	0.48	0.000212	98.10046732
Hip circumference	Falls	rs7124681	11	A	C	0.019971	-0.00174699	0.00244272	0.00120059	2.95121E-16	0.15	0.000199	91.68978075
Hip circumference	Falls	rs74749286	11	A	G	0.0306898	-0.000512783	0.00389923	0.00192078	3.5359E-15	0.79	0.000184	84.97655046
Hip circumference	Falls	rs7480253	11	T	C	0.0125489	-0.000942055	0.0024362	0.00119798	2.59263E-07	0.43	7.88E-05	36.39603941
Hip circumference	Falls	rs7930275	11	T	C	0.0186124	-0.00181571	0.0029418	0.00145026	2.50478E-10	0.21	0.000119	54.90945775
Hip circumference	Falls	rs7940866	11	A	T	0.0129611	0.00229446	0.00241693	0.00118916	8.20843E-08	0.0539995	8.54E-05	39.44793422
Hip circumference	Falls	rs10777859	12	G	A	-0.0154525	0.000228961	0.00240837	0.00118519	1.3992E-10	0.85	0.000122	56.47025832
Hip circumference	Falls	rs11056870	12	T	G	0.0173557	0.0025604	0.00341687	0.00168117	3.78844E-07	0.13	7.66E-05	35.39126166
Hip circumference	Falls	rs11109097	12	T	C	-0.0156389	0.00262109	0.0026263	0.00129206	2.60783E-09	0.0420001	0.000105	48.63987222
Hip circumference	Falls	rs12300276	12	A	G	0.0159752	0.00390407	0.00283175	0.00139985	1.68729E-08	0.00530005	9.45E-05	43.65675756
Hip circumference	Falls	rs1271309	12	G	A	-0.0219913	-0.000231868	0.00325813	0.00160864	1.48423E-11	0.89	0.000135	62.49333132
Hip circumference	Falls	rs1458156	12	T	C	0.0133122	0.00143211	0.0024078	0.00118416	3.22664E-08	0.23	9.08E-05	41.930258
Hip circumference	Falls	rs147730268	12	T	G	-0.0522646	-0.00181298	0.00433365	0.00214397	1.74181E-33	0.4	0.000432	199.5153354
Hip circumference	Falls	rs1964599	12	T	C	-0.022999	-0.00186408	0.0025863	0.00126971	5.99653E-19	0.14	0.000235	108.4747841
Hip circumference	Falls	rs1979440	12	C	T	-0.0149999	0.00211764	0.00245372	0.0012045	9.77958E-10	0.0790005	0.000111	51.26198383
Hip circumference	Falls	rs2013002	12	C	T	0.018078	0.000393988	0.00245987	0.00120725	1.99894E-13	0.74	0.00016	74.087534
Hip circumference	Falls	rs2129869	12	T	A	-0.0214884	-0.00207	0.00291524	0.00142942	1.69668E-13	0.15	0.000161	74.5295018
Hip circumference	Falls	rs2897968	12	A	G	0.0137469	-0.000345248	0.00246483	0.00121361	2.4454E-08	0.780001	9.24E-05	42.66819703

Hip circumference	Falls	rs34911908	12	A	G	0.0130448	0.00375062	0.00248022	0.00121979	1.44494E-07	0.0021	8.22E-05	37.94574876
Hip circumference	Falls	rs36120387	12	T	C	-0.0203173	0.00293188	0.00388325	0.00191239	1.67764E-07	0.13	8.13E-05	37.55000267
Hip circumference	Falls	rs3730071	12	A	C	-0.0378377	-0.00769069	0.00701291	0.00345479	6.84006E-08	0.0259998	8.65E-05	39.93205086
Hip circumference	Falls	rs4842681	12	G	C	0.0152955	0.00287821	0.00268676	0.00132246	1.2498E-08	0.0299999	9.63E-05	44.45680995
Hip circumference	Falls	rs55726687	12	A	G	0.0213623	-0.000269637	0.00294561	0.0014515	4.10677E-13	0.85	0.000156	72.14632533
Hip circumference	Falls	rs6539064	12	G	C	-0.0187741	-0.00070166	0.00276939	0.00136023	1.21088E-11	0.61	0.000137	63.04036247
Hip circumference	Falls	rs66630777	12	T	G	-0.0150299	-0.000609758	0.0027754	0.00136893	6.11913E-08	0.66	8.71E-05	40.22811672
Hip circumference	Falls	rs697883	12	A	G	-0.0223877	-0.0018236	0.00429796	0.00211497	1.90068E-07	0.39	8.06E-05	37.21884623
Hip circumference	Falls	rs7132908	12	A	G	0.0248529	-0.000719674	0.00247489	0.00121664	1.00392E-23	0.55	0.0003	138.328297
Hip circumference	Falls	rs7305790	12	C	A	0.013906	-0.000672335	0.00268774	0.00131842	2.29462E-07	0.61	7.95E-05	36.71965804
Hip circumference	Falls	rs76895963	12	G	T	0.0962201	-0.00242466	0.00932168	0.0045803	5.64157E-25	0.6	0.000316	146.1544069
Hip circumference	Falls	rs78470967	12	A	T	0.0359219	0.00518883	0.00606978	0.00298796	3.25889E-09	0.0819993	0.000104	48.04424643
Hip circumference	Falls	rs882378	12	C	A	0.0154797	0.000728332	0.00261008	0.00128658	3.0187E-09	0.57	0.000104	48.24875697
Hip circumference	Falls	rs11839227	13	C	T	-0.0181298	0.00212673	0.00311049	0.00153311	5.59461E-09	0.17	0.000101	46.60119943
Hip circumference	Falls	rs12877270	13	A	G	0.0151117	0.00204673	0.00244173	0.00119951	6.06373E-10	0.0879995	0.000114	52.54120825
Hip circumference	Falls	rs1441264	13	A	G	0.0168087	0.000175051	0.00249936	0.00123026	1.7559E-11	0.89	0.000134	62.04107428
Hip circumference	Falls	rs1886558	13	G	A	-0.0159051	-0.0023562	0.00248456	0.00122287	1.53954E-10	0.0539995	0.000122	56.21374474
Hip circumference	Falls	rs41284816	13	T	G	0.0791573	0.00606636	0.00891062	0.00442881	6.51028E-19	0.17	0.000234	108.251715
Hip circumference	Falls	rs7982447	13	C	T	0.0162648	0.00130983	0.0029811	0.00146723	4.87405E-08	0.37	8.84E-05	40.83316588
Hip circumference	Falls	rs9512696	13	G	A	0.0176296	0.00078365	0.00254397	0.0012516	4.21794E-12	0.53	0.000143	65.87636597
Hip circumference	Falls	rs9513030	13	C	G	-0.0132237	-0.00194904	0.00248635	0.00122271	1.04691E-07	0.11	8.4E-05	38.80164484
Hip circumference	Falls	rs9565536	13	A	T	-0.0143477	-7.25079E-05	0.00269818	0.00132535	1.05228E-07	0.96	8.4E-05	38.78746748
Hip circumference	Falls	rs9603697	13	T	C	0.0139508	0.00167521	0.00256358	0.00126229	5.27473E-08	0.18	8.8E-05	40.62310948
Hip circumference	Falls	rs1285997	14	G	C	0.0234272	-0.000353874	0.0026491	0.00130555	9.3261E-19	0.79	0.000232	107.2785148
Hip circumference	Falls	rs12883788	14	T	C	0.0154765	0.00288691	0.00242117	0.00119093	1.63761E-10	0.015	0.000121	56.04845213
Hip circumference	Falls	rs1955695	14	G	A	-0.0191353	-0.00049404	0.00248689	0.00122069	1.42495E-14	0.69	0.000176	81.2130996

Hip circumference	Falls	rs28479795	14	T	C	0.0244818	-0.00327723	0.00289895	0.00142709	3.0507E-17	0.0219999	0.000212	97.83042249
Hip circumference	Falls	rs3803286	14	G	A	-0.0175828	0.00148349	0.00254731	0.0012538	5.11918E-12	0.24	0.000142	65.35535208
Hip circumference	Falls	rs6575340	14	A	G	0.0204335	0.00151177	0.0025066	0.00123225	3.59584E-16	0.22	0.000197	91.15576519
Hip circumference	Falls	rs71413981	14	A	G	0.0191825	0.000648371	0.00325375	0.00160679	3.73869E-09	0.69	0.000103	47.67719965
Hip circumference	Falls	rs7145337	14	T	C	-0.0159056	-0.00139167	0.00265409	0.00130359	2.06381E-09	0.29	0.000107	49.26488904
Hip circumference	Falls	rs8011368	14	T	C	-0.016411	-0.00146584	0.00270853	0.00133361	1.3712E-09	0.27	0.000109	50.35834837
Hip circumference	Falls	rs9323375	14	A	T	0.0164338	0.00158973	0.00284639	0.00140141	7.76837E-09	0.26	9.9E-05	45.72522719
Hip circumference	Falls	rs9788550	14	C	G	-0.0207023	0.00137678	0.00279459	0.00137568	1.28529E-13	0.32	0.000163	75.27827895
Hip circumference	Falls	rs1559676	15	G	C	-0.0142302	-0.0016814	0.00265959	0.00130712	8.77566E-08	0.2	8.5E-05	39.2700372
Hip circumference	Falls	rs2715439	15	T	C	0.0157972	-0.0016451	0.00241641	0.00118978	6.26614E-11	0.17	0.000127	58.62565012
Hip circumference	Falls	rs34769775	15	T	C	-0.0165941	-0.00221871	0.00262638	0.00129442	2.64893E-10	0.0870001	0.000119	54.75968839
Hip circumference	Falls	rs35874463	15	G	A	0.0325725	0.00675279	0.00514563	0.00253371	2.45273E-10	0.00769999	0.000119	54.96595315
Hip circumference	Falls	rs4776970	15	T	A	-0.0201543	0.000726307	0.002508	0.00123166	9.31322E-16	0.56	0.000192	88.58273004
Hip circumference	Falls	rs4777541	15	T	C	0.0191721	0.00249722	0.00284958	0.00139794	1.72227E-11	0.0739997	0.000134	62.09353006
Hip circumference	Falls	rs4843158	15	C	G	0.0202544	0.00272556	0.00258511	0.00127394	4.70111E-15	0.032	0.000182	84.20723304
Hip circumference	Falls	rs4966012	15	G	C	0.0149488	-0.00270192	0.00257511	0.00126686	6.43888E-09	0.0329997	0.0001	46.22636878
Hip circumference	Falls	rs55707100	15	T	C	-0.0400543	-0.00190268	0.00774721	0.00371079	2.34018E-07	0.61	7.94E-05	36.66707976
Hip circumference	Falls	rs8023263	15	T	G	0.0148049	-0.00103054	0.00240753	0.00118581	7.78718E-10	0.38	0.000112	51.87238647
Hip circumference	Falls	rs8042404	15	A	G	0.0170942	0.00108594	0.00270872	0.00133014	2.77939E-10	0.41	0.000118	54.63085787
Hip circumference	Falls	rs11150461	16	G	C	-0.0150251	0.000965049	0.00271243	0.00133382	3.03816E-08	0.47	9.12E-05	42.09071967
Hip circumference	Falls	rs1150188	16	C	G	-0.0144553	-0.00219875	0.0024709	0.00121699	4.91394E-09	0.0710003	0.000102	46.94751044
Hip circumference	Falls	rs117342986	16	T	C	0.0410233	0.00258089	0.00782839	0.00386976	1.60395E-07	0.5	8.16E-05	37.66907592
Hip circumference	Falls	rs12920259	16	A	G	-0.0164136	-0.0041862	0.00247121	0.00121495	3.10027E-11	0.000569994	0.000131	60.51416887
Hip circumference	Falls	rs13333747	16	C	T	-0.0239707	-0.0021432	0.00313212	0.00153836	1.96562E-14	0.16	0.000174	80.34397883
Hip circumference	Falls	rs25849	16	G	C	0.020294	0.00256462	0.00266649	0.00131073	2.73212E-14	0.05	0.000172	79.4555212
Hip circumference	Falls	rs35057083	16	T	C	0.0144062	-0.000727078	0.00260593	0.00127827	3.23691E-08	0.57	9.08E-05	41.92200792

Hip circumference	Falls	rs4402589	16	G	T	0.0295957	0.000065121	0.00241926	0.00118909	2.09652E-34	0.96	0.000444	205.286708
Hip circumference	Falls	rs4985407	16	G	A	0.0153191	0.00285223	0.00240871	0.00118565	2.02134E-10	0.016	0.00012	55.48379499
Hip circumference	Falls	rs56094641	16	G	A	0.0610681	0.00460695	0.00245004	0.00120512	5.2602E-137	0.000129999	0.001842	852.2188851
Hip circumference	Falls	rs62037365	16	G	C	0.0322128	-0.00076343	0.00245225	0.00120784	2.09363E-39	0.53	0.000512	236.698853
Hip circumference	Falls	rs72801854	16	A	G	0.0183289	-0.00201245	0.00261408	0.0012863	2.36102E-12	0.12	0.000146	67.43785406
Hip circumference	Falls	rs756717	16	A	G	-0.015681	0.000760757	0.00248428	0.00122281	2.75645E-10	0.53	0.000118	54.65313903
Hip circumference	Falls	rs879620	16	T	C	0.027011	0.000811503	0.00248095	0.00121845	1.33814E-27	0.51	0.000352	162.5975471
Hip circumference	Falls	rs10153248	17	G	A	-0.0170889	-0.00109446	0.0024225	0.00119102	1.7386E-12	0.36	0.000148	68.2604792
Hip circumference	Falls	rs113866544	17	C	T	0.0327216	-0.000418834	0.00479193	0.00234635	8.59607E-12	0.86	0.000139	63.96123187
Hip circumference	Falls	rs1396513	17	T	C	0.0183151	0.000529143	0.00240892	0.001185	2.90068E-14	0.66	0.000172	79.29440071
Hip circumference	Falls	rs1918249	17	A	T	0.0159582	0.00274166	0.00281683	0.00137829	1.46903E-08	0.0470002	9.53E-05	44.02660729
Hip circumference	Falls	rs2355374	17	T	G	0.0128665	0.00220374	0.00243068	0.00119532	1.20138E-07	0.0649995	8.32E-05	38.43562619
Hip circumference	Falls	rs3826408	17	T	C	0.0151967	2.57616E-05	0.00241335	0.00118628	3.03928E-10	0.98	0.000118	54.39095022
Hip circumference	Falls	rs4790292	17	A	C	-0.027819	0.00100584	0.00334073	0.00164628	8.30615E-17	0.54	0.000206	95.11936935
Hip circumference	Falls	rs4794222	17	G	A	-0.0174748	0.000168795	0.00276456	0.00136413	2.60202E-10	0.9	0.000119	54.80763664
Hip circumference	Falls	rs4889867	17	T	C	-0.0165547	-0.000609308	0.00242054	0.00119129	7.97444E-12	0.61	0.000139	64.1633144
Hip circumference	Falls	rs56288810	17	G	A	0.0183373	0.00143357	0.00294765	0.00145152	4.94686E-10	0.32	0.000115	53.08693334
Hip circumference	Falls	rs58551145	17	G	A	0.0238057	0.00325688	0.00303824	0.00149491	4.68813E-15	0.0290001	0.000182	84.21441685
Hip circumference	Falls	rs6501601	17	A	G	-0.01902	-0.000146989	0.00246888	0.00121538	1.32343E-14	0.9	0.000176	81.41224849
Hip circumference	Falls	rs7226064	17	G	A	-0.01458	-0.00276898	0.00243911	0.00119947	2.2671E-09	0.021	0.000106	49.01409203
Hip circumference	Falls	rs731758	17	G	C	-0.0176334	0.000245525	0.00248153	0.00122179	1.19812E-12	0.84	0.00015	69.26307142
Hip circumference	Falls	rs11661691	18	G	T	0.0130311	-0.00185318	0.00240751	0.00118467	6.21327E-08	0.12	8.7E-05	40.18783801
Hip circumference	Falls	rs11664106	18	T	A	0.015224	0.0024034	0.002547	0.00125248	2.27175E-09	0.0549997	0.000106	49.00815443
Hip circumference	Falls	rs12607512	18	G	A	0.0131918	-0.000959825	0.00241734	0.00119038	4.84161E-08	0.42	8.85E-05	40.85087161
Hip circumference	Falls	rs12965488	18	T	C	0.0168958	0.00392204	0.00312596	0.00153512	6.4856E-08	0.0109999	8.68E-05	40.07369371
Hip circumference	Falls	rs1618725	18	T	C	-0.021172	0.000436059	0.00240567	0.00118342	1.36333E-18	0.709999	0.00023	106.2478996

Hip circumference	Falls	rs273759	18	G	A	-0.0129908	-0.00105191	0.00241253	0.00118718	7.26089E-08	0.38	8.61E-05	39.77361226
Hip circumference	Falls	rs57636386	18	C	T	-0.036841	-0.00218238	0.004358	0.00214141	2.834E-17	0.31	0.000212	98.02956052
Hip circumference	Falls	rs6567160	18	C	T	0.0496794	0.00225129	0.00283765	0.00139951	1.35301E-68	0.11	0.00091	420.4396982
Hip circumference	Falls	rs7238896	18	G	A	0.0212081	0.00124156	0.00347746	0.0017131	1.07026E-09	0.47	0.00011	51.02087778
Hip circumference	Falls	rs7241211	18	T	C	0.0130235	0.000492279	0.00243868	0.00119836	9.27962E-08	0.68	8.47E-05	39.12140855
Hip circumference	Falls	rs8087074	18	T	G	0.0144204	0.000747965	0.00275049	0.00135127	1.58201E-07	0.58	8.17E-05	37.70536604
Hip circumference	Falls	rs8097809	18	A	G	-0.017963	0.00256688	0.00355561	0.00173587	4.37401E-07	0.14	7.58E-05	35.01049001
Hip circumference	Falls	rs9967367	18	T	C	-0.0164671	-0.00149361	0.00265273	0.00130677	5.38667E-10	0.25	0.000114	52.85874349
Hip circumference	Falls	rs10404726	19	T	C	-0.0166254	-0.000161352	0.00240995	0.00118719	5.25896E-12	0.89	0.000141	65.28250776
Hip circumference	Falls	rs11882409	19	A	C	0.0181856	0.00365092	0.00270443	0.00133047	1.76604E-11	0.0061	0.000134	62.02581313
Hip circumference	Falls	rs1231281	19	A	G	-0.013948	-0.000631962	0.00240776	0.00118242	6.92309E-09	0.59	9.97E-05	46.03267091
Hip circumference	Falls	rs12972720	19	C	G	0.0177427	0.00106823	0.00243123	0.00119739	2.93089E-13	0.37	0.000158	73.0560209
Hip circumference	Falls	rs141622900	19	A	G	0.0316845	0.000229593	0.00551034	0.00271844	8.93079E-09	0.93	9.82E-05	45.35295723
Hip circumference	Falls	rs2238689	19	C	T	-0.0126136	0.00150321	0.00245659	0.00120741	2.82898E-07	0.21	7.83E-05	36.16441442
Hip circumference	Falls	rs273505	19	C	T	0.0147072	-0.00137993	0.00243691	0.00119779	1.58957E-09	0.25	0.000108	49.96313764
Hip circumference	Falls	rs3218036	19	A	G	0.0194082	0.000921725	0.0025688	0.00126455	4.18794E-14	0.47	0.00017	78.30303944
Hip circumference	Falls	rs34013042	19	T	C	0.0154229	0.00381298	0.00275189	0.00135273	2.0904E-08	0.00479999	9.33E-05	43.08624361
Hip circumference	Falls	rs350832	19	A	G	0.0151016	0.00169877	0.00287737	0.00141478	1.53494E-07	0.23	8.18E-05	37.78532748
Hip circumference	Falls	rs3810291	19	A	G	0.023107	0.00231761	0.0025705	0.00126163	2.50035E-19	0.0659994	0.00024	110.8461465
Hip circumference	Falls	rs1293395	20	T	G	-0.0274278	-0.00773788	0.00453926	0.00223264	1.52037E-09	0.000530005	0.000108	50.08186778
Hip circumference	Falls	rs143384	20	G	A	0.0281276	0.000522578	0.00244924	0.00120406	1.60435E-30	0.66	0.000392	180.913658
Hip circumference	Falls	rs2236519	20	A	G	-0.0208582	0.000275934	0.00248544	0.00122328	4.79181E-17	0.82	0.000209	96.60859703
Hip circumference	Falls	rs3746759	20	G	T	-0.017084	-0.000130647	0.00299536	0.00146271	1.17482E-08	0.93	9.66E-05	44.62207492
Hip circumference	Falls	rs55886426	20	G	C	-0.0337871	0.000220337	0.00552551	0.0027497	9.68077E-10	0.94	0.000111	51.28918326
Hip circumference	Falls	rs6029180	20	G	A	0.0136196	0.0011446	0.00259026	0.00127388	1.4571E-07	0.37	8.21E-05	37.9236976
Hip circumference	Falls	rs6080646	20	A	G	-0.0133714	-2.28511E-05	0.00241122	0.00118647	2.93346E-08	0.98	9.14E-05	42.18409869

Hip circumference	Falls	rs6111562	20	T	G	0.0334476	0.0093183	0.00616057	0.00303655	5.66161E-08	0.0021	8.76E-05	40.43494119
Hip circumference	Falls	rs6142059	20	C	T	0.0159821	-0.00158888	0.00240831	0.00118559	3.22255E-11	0.18	0.000131	60.41038407
Hip circumference	Falls	rs7274811	20	T	G	-0.0211475	0.00115315	0.00275032	0.00135036	1.48559E-14	0.39	0.000176	81.09989664
Hip circumference	Falls	rs10427502	21	A	G	-0.0129865	0.00190652	0.00248172	0.00122099	1.67001E-07	0.12	8.13E-05	37.56188388
Hip circumference	Falls	rs60984707	21	T	C	-0.018209	-0.00235272	0.00313296	0.00153748	6.17604E-09	0.13	0.0001	46.33734849
Hip circumference	Falls	rs76040172	21	A	G	-0.034453	0.00255371	0.0053139	0.0026329	8.97016E-11	0.33	0.000125	57.66283902
Hip circumference	Falls	rs8133137	21	G	A	0.0150007	-0.00139399	0.00255038	0.00125212	4.06359E-09	0.27	0.000103	47.45499704
Hip circumference	Falls	rs11704728	22	T	C	0.0152795	0.00310716	0.00303796	0.00149387	4.91983E-07	0.0379997	7.51E-05	34.69947231
Hip circumference	Falls	rs133015	22	G	C	0.0129392	0.00336079	0.00242369	0.00119326	9.37022E-08	0.00490004	8.47E-05	39.09573632
Hip circumference	Falls	rs138767	22	C	T	0.0146128	-0.00131307	0.0025195	0.00124092	6.64186E-09	0.29	9.99E-05	46.14310792
Hip circumference	Falls	rs140947018	22	A	G	0.0369121	-0.00479512	0.00727511	0.00358277	3.90256E-07	0.18	7.65E-05	35.31238626
Hip circumference	Falls	rs1569497	22	G	A	0.0148474	0.000741778	0.00241868	0.00119016	8.33221E-10	0.53	0.000112	51.69073152
Waist circumference	Falls	rs11165493	1	A	G	0.002282	0.00125488	0.002282	0.00125488	1.98857E-07	0.62	8.03E-05	27.04546916
Waist circumference	Falls	rs11208779	1	C	G	0.00216129	0.00118796	0.00216129	0.00118796	1.95637E-09	0.000560003	0.000107	36.01865794
Waist circumference	Falls	rs112566467	1	T	C	0.00264517	0.00145509	0.00264517	0.00145509	7.25437E-12	0.24	0.000139	46.96075916
Waist circumference	Falls	rs12037698	1	A	G	0.00308427	0.00170438	0.00308427	0.00170438	7.45641E-08	0.0032	8.6E-05	28.94379786
Waist circumference	Falls	rs12060713	1	A	G	0.00220788	0.00121457	0.00220788	0.00121457	3.14768E-08	0.79	9.09E-05	30.61559243
Waist circumference	Falls	rs12096864	1	C	T	0.00336684	0.00185134	0.00336684	0.00185134	1.5428E-10	0.18	0.000122	40.97644057
Waist circumference	Falls	rs12128526	1	A	G	0.00216039	0.00118793	0.00216039	0.00118793	2.93934E-08	0.96	9.13E-05	30.74875727
Waist circumference	Falls	rs12140153	1	T	G	0.00373213	0.00207595	0.00373213	0.00207595	1.28855E-11	0.68	0.000136	45.83536668
Waist circumference	Falls	rs12725767	1	G	A	0.00225266	0.00123933	0.00225266	0.00123933	1.28615E-07	0.29	8.28E-05	27.88803518
Waist circumference	Falls	rs1490382	1	G	A	0.00221817	0.00121915	0.00221817	0.00121915	3.19411E-07	0.57	7.76E-05	26.1298475
Waist circumference	Falls	rs241461	1	A	T	0.00230559	0.00126932	0.00230559	0.00126932	3.51641E-15	0.0759994	0.000184	61.95947652
Waist circumference	Falls	rs2678204	1	G	T	0.00227049	0.00124922	0.00227049	0.00124922	1.14921E-17	0.719999	0.000218	73.24574605
Waist circumference	Falls	rs3028171	1	C	A	0.00267155	0.00147046	0.00267155	0.00147046	9.92019E-08	0.95	8.43E-05	28.39070165
Waist circumference	Falls	rs309535	1	G	A	0.0029032	0.00159157	0.0029032	0.00159157	1.58935E-07	0.61	8.16E-05	27.47865645

Waist circumference	Falls	rs3766823	1	A	G	0.00285308	0.00156884	0.00285308	0.00156884	4.75226E-08	0.002	8.86E-05	29.81651158
Waist circumference	Falls	rs3935032	1	T	C	0.00225489	0.00124115	0.00225489	0.00124115	8.72971E-11	0.23	0.000125	42.08996442
Waist circumference	Falls	rs41279738	1	G	T	0.00679322	0.00373339	0.00679322	0.00373339	9.56093E-14	0.37	0.000165	55.45976177
Waist circumference	Falls	rs4322261	1	A	G	0.00290548	0.00160197	0.00290548	0.00160197	2.93083E-10	0.00539995	0.000118	39.72286113
Waist circumference	Falls	rs4562625	1	G	C	0.00220784	0.00121542	0.00220784	0.00121542	1.45415E-07	0.86	8.21E-05	27.65074267
Waist circumference	Falls	rs4652839	1	G	C	0.00229333	0.00126058	0.00229333	0.00126058	1.61142E-07	0.00599998	8.15E-05	27.45189173
Waist circumference	Falls	rs4926726	1	A	G	0.00215892	0.00118653	0.00215892	0.00118653	1.51768E-07	0.3	8.19E-05	27.56770424
Waist circumference	Falls	rs539515	1	C	A	0.00265034	0.00146606	0.00265034	0.00146606	1.34679E-40	0.26	0.000529	178.0146397
Waist circumference	Falls	rs588660	1	A	G	0.00218549	0.0012009	0.00218549	0.0012009	3.5229E-11	0.26	0.00013	43.86533955
Waist circumference	Falls	rs61813324	1	T	C	0.00319021	0.00175263	0.00319021	0.00175263	2.77294E-10	0.00269998	0.000118	39.83063869
Waist circumference	Falls	rs61826867	1	G	A	0.00343971	0.00188133	0.00343971	0.00188133	3.38454E-08	0.95	9.05E-05	30.47489614
Waist circumference	Falls	rs6687953	1	G	A	0.00220383	0.00121154	0.00220383	0.00121154	1.74936E-09	0.000409996	0.000108	36.23681294
Waist circumference	Falls	rs71658797	1	A	T	0.00327841	0.00181878	0.00327841	0.00181878	2.36157E-19	0.0439997	0.00024	80.92046268
Waist circumference	Falls	rs7531118	1	C	T	0.00216988	0.0011944	0.00216988	0.0011944	4.37421E-13	0.00016	0.000156	52.47145838
Waist circumference	Falls	rs815163	1	C	T	0.00216641	0.00119179	0.00216641	0.00119179	4.55491E-10	0.017	0.000115	38.86160533
Waist circumference	Falls	rs10172196	2	A	G	0.00234352	0.00128744	0.00234352	0.00128744	2.19563E-09	0.1	0.000106	35.79383735
Waist circumference	Falls	rs10803762	2	A	G	0.00231166	0.00126932	0.00231166	0.00126932	3.81804E-10	0.0749998	0.000116	39.20606214
Waist circumference	Falls	rs114964326	2	A	G	0.00640531	0.00355926	0.00640531	0.00355926	8.89324E-08	0.66	8.5E-05	28.60227646
Waist circumference	Falls	rs12619178	2	T	C	0.00219213	0.00120658	0.00219213	0.00120658	6.1546E-12	0.0369999	0.00014	47.28322867
Waist circumference	Falls	rs13420048	2	A	C	0.00224737	0.00123274	0.00224737	0.00123274	5.37279E-10	0.37	0.000114	38.53886092
Waist circumference	Falls	rs13423444	2	A	G	0.00311058	0.00170969	0.00311058	0.00170969	1.03093E-08	0.025	9.74E-05	32.78383227
Waist circumference	Falls	rs13427822	2	G	A	0.002443	0.00134583	0.002443	0.00134583	6.21298E-11	0.1	0.000127	42.75489692
Waist circumference	Falls	rs1405261	2	A	T	0.00217773	0.00119647	0.00217773	0.00119647	1.98834E-07	0.94	8.03E-05	27.04575015
Waist circumference	Falls	rs1609303	2	A	T	0.002238	0.00123141	0.002238	0.00123141	2.0893E-14	0.12	0.000174	58.4514224
Waist circumference	Falls	rs1881934	2	T	A	0.00227206	0.00124854	0.00227206	0.00124854	9.25358E-08	0.0490004	8.47E-05	28.52547019
Waist circumference	Falls	rs2015769	2	G	A	0.00216238	0.00118928	0.00216238	0.00118928	1.30608E-07	0.61	8.27E-05	27.85854524

Waist circumference	Falls	rs2196150	2	T	G	0.00220711	0.00121528	0.00220711	0.00121528	4.18938E-07	0.0870001	7.61E-05	25.60599994
Waist circumference	Falls	rs2433733	2	A	G	0.00230959	0.00126649	0.00230959	0.00126649	2.67793E-13	0.87	0.000159	53.43608164
Waist circumference	Falls	rs2861692	2	C	T	0.00240824	0.0013243	0.00240824	0.0013243	1.79391E-13	0.0350002	0.000161	54.22250672
Waist circumference	Falls	rs4482463	2	A	C	0.00406987	0.00222627	0.00406987	0.00222627	1.48081E-12	0.42	0.000149	50.07742585
Waist circumference	Falls	rs4549080	2	T	C	0.00226903	0.0012463	0.00226903	0.0012463	3.14196E-08	0.0580003	9.09E-05	30.61924423
Waist circumference	Falls	rs4670172	2	A	T	0.0022548	0.00124144	0.0022548	0.00124144	2.68022E-07	0.0449997	7.86E-05	26.46853333
Waist circumference	Falls	rs4671328	2	G	T	0.00218011	0.00119903	0.00218011	0.00119903	5.39262E-14	0.0025	0.000168	56.58540131
Waist circumference	Falls	rs62106258	2	C	T	0.00500793	0.00275153	0.00500793	0.00275153	5.4325E-42	0.0002	0.000547	184.4039839
Waist circumference	Falls	rs6433243	2	C	T	0.00225296	0.00123976	0.00225296	0.00123976	1.98198E-10	0.0389996	0.00012	40.48672696
Waist circumference	Falls	rs6711584	2	A	G	0.00216817	0.00119416	0.00216817	0.00119416	6.5429E-08	0.0710003	8.67E-05	29.19678251
Waist circumference	Falls	rs6739755	2	G	A	0.0022029	0.00121231	0.0022029	0.00121231	2.49115E-14	0.00051	0.000173	58.10523812
Waist circumference	Falls	rs72844755	2	C	A	0.00397385	0.00217931	0.00397385	0.00217931	4.78079E-07	0.75	7.53E-05	25.35139035
Waist circumference	Falls	rs72917544	2	A	G	0.00278077	0.00153276	0.00278077	0.00153276	1.27057E-09	0.02	0.000109	36.85985883
Waist circumference	Falls	rs73985439	2	C	A	0.00233516	0.00128526	0.00233516	0.00128526	9.72994E-10	0.44	0.000111	37.38011502
Waist circumference	Falls	rs76286777	2	C	T	0.00260034	0.0014332	0.00260034	0.0014332	3.87347E-24	0.32	0.000305	102.7287807
Waist circumference	Falls	rs80330591	2	A	G	0.00303827	0.00167332	0.00303827	0.00167332	4.47899E-08	0.92	8.89E-05	29.93157028
Waist circumference	Falls	rs1154988	3	A	T	0.00257031	0.00141145	0.00257031	0.00141145	9.6984E-12	0.000290001	0.000138	46.39193945
Waist circumference	Falls	rs11921483	3	A	C	0.00216076	0.00118945	0.00216076	0.00118945	3.26806E-07	0.12	7.75E-05	26.08569727
Waist circumference	Falls	rs13322435	3	G	A	0.00220729	0.0012117	0.00220729	0.0012117	1.10332E-13	0.0420001	0.000164	55.17888499
Waist circumference	Falls	rs1454687	3	G	C	0.00215185	0.00118345	0.00215185	0.00118345	2.02162E-16	0.25	0.000201	67.58821181
Waist circumference	Falls	rs1515733	3	C	G	0.00214986	0.00118293	0.00214986	0.00118293	6.5681E-09	0.34	1E-04	33.66057155
Waist circumference	Falls	rs17639996	3	A	G	0.00301196	0.00166357	0.00301196	0.00166357	3.92844E-08	0.760001	8.97E-05	30.18583003
Waist circumference	Falls	rs2016469	3	A	G	0.00224103	0.00123342	0.00224103	0.00123342	3.26385E-08	0.17	9.07E-05	30.54539688
Waist circumference	Falls	rs2035831	3	C	G	0.00229195	0.00126011	0.00229195	0.00126011	2.48788E-07	0.44	7.9E-05	26.61226463
Waist circumference	Falls	rs2455821	3	A	C	0.00242133	0.00133308	0.00242133	0.00133308	2.97687E-10	0.001	0.000118	39.69219081
Waist circumference	Falls	rs4856407	3	T	C	0.00223286	0.00122944	0.00223286	0.00122944	4.20049E-12	0.26	0.000143	48.03222612

Waist circumference	Falls	rs59815219	3	T	C	0.00215144	0.00118342	0.00215144	0.00118342	1.47401E-07	0.35	8.21E-05	27.62422343
Waist circumference	Falls	rs62246314	3	A	G	0.00356414	0.0019499	0.00356414	0.0019499	1.16598E-09	0.25	0.00011	37.02759412
Waist circumference	Falls	rs62261725	3	G	A	0.00229544	0.00126405	0.00229544	0.00126405	2.41268E-15	0.53	0.000186	62.70166503
Waist circumference	Falls	rs73140125	3	G	A	0.0032274	0.00178045	0.0032274	0.00178045	2.58964E-09	0.00619998	0.000105	35.47211124
Waist circumference	Falls	rs7610647	3	G	A	0.00237559	0.00130484	0.00237559	0.00130484	3.30027E-07	0.75	7.74E-05	26.06666864
Waist circumference	Falls	rs7635592	3	T	C	0.00267244	0.0014663	0.00267244	0.0014663	1.28292E-15	0.2	0.00019	63.94600831
Waist circumference	Falls	rs8192675	3	C	T	0.00237273	0.0013038	0.00237273	0.0013038	1.12834E-10	0.017	0.000124	41.5877758
Waist circumference	Falls	rs869400	3	G	T	0.00278235	0.00152583	0.00278235	0.00152583	4.97279E-13	0.021	0.000155	52.21943348
Waist circumference	Falls	rs9289630	3	C	G	0.00221351	0.00121781	0.00221351	0.00121781	2.30038E-12	0.25	0.000146	49.21293373
Waist circumference	Falls	rs9814633	3	A	G	0.0022665	0.00124532	0.0022665	0.00124532	2.14333E-07	0.26	7.99E-05	26.90022962
Waist circumference	Falls	rs9843653	3	C	T	0.00215278	0.00118412	0.00215278	0.00118412	2.67609E-22	0.0470002	0.00028	94.33919143
Waist circumference	Falls	rs9849919	3	T	C	0.00226642	0.00124635	0.00226642	0.00124635	4.2418E-07	0.32	7.6E-05	25.58223762
Waist circumference	Falls	rs9867068	3	G	C	0.00249685	0.00137425	0.00249685	0.00137425	3.42768E-13	0.95	0.000157	52.95078026
Waist circumference	Falls	rs9968060	3	T	C	0.00228856	0.00125768	0.00228856	0.00125768	4.38127E-11	0.19	0.000129	43.43840706
Waist circumference	Falls	rs10938398	4	A	G	0.00217734	0.00119701	0.00217734	0.00119701	1.67958E-21	0.33	0.000269	90.70319779
Waist circumference	Falls	rs11099020	4	T	C	0.00224865	0.00123483	0.00224865	0.00123483	2.06943E-08	0.00929994	9.34E-05	31.43006867
Waist circumference	Falls	rs12506689	4	A	G	0.00223518	0.001229	0.00223518	0.001229	1.33144E-07	0.2	8.26E-05	27.82102557
Waist circumference	Falls	rs1383723	4	T	A	0.00261578	0.00143972	0.00261578	0.00143972	2.61758E-11	0.14	0.000132	44.4468232
Waist circumference	Falls	rs1472872	4	G	A	0.003991	0.00219533	0.003991	0.00219533	3.86803E-08	0.13	8.97E-05	30.21583276
Waist circumference	Falls	rs1724557	4	A	C	0.00219535	0.00120756	0.00219535	0.00120756	1.52332E-10	0.75	0.000122	41.00117371
Waist circumference	Falls	rs2102278	4	G	A	0.00230699	0.00126723	0.00230699	0.00126723	8.20994E-08	0.94	8.54E-05	28.75717856
Waist circumference	Falls	rs2192527	4	G	A	0.00216127	0.00118761	0.00216127	0.00118761	4.31122E-12	0.00599998	0.000143	47.98129515
Waist circumference	Falls	rs2798304	4	C	T	0.00216567	0.00119193	0.00216567	0.00119193	2.99116E-07	0.0129999	7.8E-05	26.25635516
Waist circumference	Falls	rs4419475	4	T	A	0.00218769	0.00120395	0.00218769	0.00120395	8.79832E-08	0.42	8.5E-05	28.62327523
Waist circumference	Falls	rs4527444	4	G	A	0.00215952	0.00118826	0.00215952	0.00118826	2.27096E-07	0.0710003	7.96E-05	26.78869657
Waist circumference	Falls	rs6536575	4	C	T	0.00215288	0.00118432	0.00215288	0.00118432	7.72752E-08	0.2	8.58E-05	28.87449886

Waist circumference	Falls	rs73213484	4	T	A	0.00311019	0.00170084	0.00311019	0.00170084	8.20673E-10	0.0012	0.000112	37.71236755
Waist circumference	Falls	rs7377083	4	A	C	0.00218612	0.00120368	0.00218612	0.00120368	4.14572E-11	0.35	0.000129	43.5467655
Waist circumference	Falls	rs750090	4	C	T	0.00226957	0.00124803	0.00226957	0.00124803	3.18845E-08	0.05	9.09E-05	30.5905394
Waist circumference	Falls	rs75152244	4	G	A	0.00315871	0.00172956	0.00315871	0.00172956	1.64361E-07	0.55	8.14E-05	27.41368844
Waist circumference	Falls	rs809955	4	A	G	0.00223476	0.00122964	0.00223476	0.00122964	3.3302E-08	0.0269998	9.06E-05	30.50659619
Waist circumference	Falls	rs12186509	5	G	T	0.00245425	0.00135131	0.00245425	0.00135131	8.53867E-08	0.38	8.52E-05	28.68122368
Waist circumference	Falls	rs146311547	5	G	A	0.0032254	0.00177384	0.0032254	0.00177384	3.78216E-08	0.28	8.99E-05	30.25954996
Waist circumference	Falls	rs1503527	5	T	C	0.00215212	0.00118337	0.00215212	0.00118337	2.36385E-07	0.0779992	7.93E-05	26.71131626
Waist circumference	Falls	rs1582931	5	A	G	0.00217176	0.00119488	0.00217176	0.00119488	1.35457E-11	0.00016	0.000136	45.73684317
Waist circumference	Falls	rs1985524	5	C	G	0.00216006	0.00118809	0.00216006	0.00118809	1.61102E-07	0.24	8.15E-05	27.45267864
Waist circumference	Falls	rs2126165	5	G	A	0.00215364	0.00118376	0.00215364	0.00118376	3.08177E-11	0.000700003	0.000131	44.12741002
Waist circumference	Falls	rs2307111	5	C	T	0.00220411	0.00121084	0.00220411	0.00121084	7.65949E-29	0.2	0.000369	124.2119761
Waist circumference	Falls	rs245775	5	G	A	0.0024218	0.00133254	0.0024218	0.00133254	2.69774E-11	0.00359998	0.000132	44.3881222
Waist circumference	Falls	rs248142	5	C	T	0.00366822	0.00201088	0.00366822	0.00201088	6.19028E-08	0.48	8.7E-05	29.30418486
Waist circumference	Falls	rs286818	5	A	T	0.00286927	0.0015789	0.00286927	0.0015789	2.17691E-10	0.5	0.00012	40.30318411
Waist circumference	Falls	rs34483452	5	A	C	0.0031711	0.00173816	0.0031711	0.00173816	2.36919E-15	0.021	0.000186	62.73732978
Waist circumference	Falls	rs66637616	5	A	T	0.00225343	0.00124071	0.00225343	0.00124071	1.47221E-07	0.0140001	8.21E-05	27.62680449
Waist circumference	Falls	rs67632512	5	A	C	0.00338782	0.00186309	0.00338782	0.00186309	1.46329E-08	0.83	9.54E-05	32.10292676
Waist circumference	Falls	rs67913249	5	G	C	0.00227292	0.0012518	0.00227292	0.0012518	8.5312E-08	0.25	8.52E-05	28.68268533
Waist circumference	Falls	rs6873229	5	T	C	0.00482223	0.00265433	0.00482223	0.00265433	1.04328E-07	0.13	8.4E-05	28.29321398
Waist circumference	Falls	rs7442885	5	G	C	0.00264733	0.00144483	0.00264733	0.00144483	5.55392E-17	0.0819993	0.000208	70.13689995
Waist circumference	Falls	rs7728095	5	G	A	0.00222461	0.001224	0.00222461	0.001224	1.29393E-10	0.44	0.000123	41.3203798
Waist circumference	Falls	rs1159974	6	C	T	0.0021527	0.001183	0.0021527	0.001183	4.19208E-09	0.74	0.000103	34.53411734
Waist circumference	Falls	rs11757278	6	C	T	0.00233576	0.00128686	0.00233576	0.00128686	2.03746E-09	0.86	0.000107	35.93960818
Waist circumference	Falls	rs1184570	6	T	C	0.00215343	0.00118384	0.00215343	0.00118384	3.83866E-10	0.86	0.000116	39.19532251
Waist circumference	Falls	rs13210406	6	G	C	0.00237523	0.00130482	0.00237523	0.00130482	8.39808E-10	0.0690001	0.000112	37.66748961

Waist circumference	Falls	rs1321519	6	G	A	0.00225862	0.00124326	0.00225862	0.00124326	5.56929E-11	0.19	0.000128	42.96887187
Waist circumference	Falls	rs17708311	6	C	G	0.00429829	0.00237205	0.00429829	0.00237205	3.40997E-09	0.021	0.000104	34.93636706
Waist circumference	Falls	rs2183947	6	A	G	0.00257399	0.00141385	0.00257399	0.00141385	9.09076E-21	0.0230001	0.000259	87.36216381
Waist circumference	Falls	rs2253310	6	G	C	0.00222456	0.00122258	0.00222456	0.00122258	8.86135E-18	0.24	0.000219	73.75898454
Waist circumference	Falls	rs2814943	6	A	G	0.00309386	0.00170053	0.00309386	0.00170053	1.11148E-28	0.021	0.000367	123.4731512
Waist circumference	Falls	rs28366156	6	C	T	0.0031838	0.00175527	0.0031838	0.00175527	2.52988E-11	0.0909997	0.000132	44.51385053
Waist circumference	Falls	rs36007635	6	A	G	0.00311724	0.00171795	0.00311724	0.00171795	4.50495E-07	0.016	7.56E-05	25.46602366
Waist circumference	Falls	rs3757050	6	G	C	0.00222199	0.00122328	0.00222199	0.00122328	4.90253E-07	0.2	7.52E-05	25.3026671
Waist circumference	Falls	rs4467770	6	A	G	0.00243256	0.00133983	0.00243256	0.00133983	8.21372E-09	0.450001	9.87E-05	33.22570614
Waist circumference	Falls	rs584170	6	A	G	0.00220623	0.00121199	0.00220623	0.00121199	2.24347E-07	0.39	7.96E-05	26.81234614
Waist circumference	Falls	rs62421910	6	G	A	0.0041117	0.00226297	0.0041117	0.00226297	4.19228E-08	0.0449997	8.93E-05	30.05968569
Waist circumference	Falls	rs72892910	6	T	G	0.00286885	0.00156957	0.00286885	0.00156957	1.30798E-28	0.21	0.000366	123.1501516
Waist circumference	Falls	rs7752202	6	T	C	0.00306988	0.00167584	0.00306988	0.00167584	2.13796E-11	0.0479999	0.000133	44.84332419
Waist circumference	Falls	rs9370243	6	T	G	0.00394402	0.00215516	0.00394402	0.00215516	2.15705E-08	0.7	9.31E-05	31.34917584
Waist circumference	Falls	rs9376507	6	T	A	0.00240493	0.00132078	0.00240493	0.00132078	1.82696E-07	0.0179999	8.08E-05	27.20919417
Waist circumference	Falls	rs9378684	6	T	C	0.00270816	0.00149074	0.00270816	0.00149074	8.06752E-10	0.46	0.000112	37.74585463
Waist circumference	Falls	rs9402104	6	A	G	0.00219525	0.00120594	0.00219525	0.00120594	3.94766E-08	0.00819993	8.96E-05	30.17651092
Waist circumference	Falls	rs9448745	6	T	A	0.00217383	0.00119418	0.00217383	0.00119418	5.36451E-08	0.36	8.79E-05	29.5819135
Waist circumference	Falls	rs9688977	6	C	T	0.00304984	0.0016852	0.00304984	0.0016852	8.804E-10	0.0959997	0.000112	37.57521488
Waist circumference	Falls	rs10236214	7	T	C	0.00225631	0.00124041	0.00225631	0.00124041	1.13682E-10	0.77	0.000123	41.57334974
Waist circumference	Falls	rs10237306	7	T	G	0.00221471	0.0012155	0.00221471	0.0012155	1.63648E-09	0.47	0.000108	36.36688312
Waist circumference	Falls	rs10269774	7	A	G	0.00230082	0.00126172	0.00230082	0.00126172	9.6323E-09	0.760001	9.78E-05	32.91564086
Waist circumference	Falls	rs11764337	7	T	C	0.00278852	0.0015245	0.00278852	0.0015245	4.25344E-08	0.000430002	8.92E-05	30.03162001
Waist circumference	Falls	rs1182199	7	A	C	0.002337	0.00128616	0.002337	0.00128616	6.59933E-12	0.99	0.00014	47.14582243
Waist circumference	Falls	rs12375196	7	A	C	0.00218693	0.00120389	0.00218693	0.00120389	9.70443E-10	0.08	0.000111	37.38552506
Waist circumference	Falls	rs1470749	7	T	G	0.0021534	0.00118466	0.0021534	0.00118466	3.25762E-10	0.1	0.000117	39.51570862

Waist circumference	Falls	rs17149254	7	C	T	0.00279014	0.00153071	0.00279014	0.00153071	1.04095E-09	0.21	0.000111	37.24868954
Waist circumference	Falls	rs17167306	7	C	A	0.00312606	0.00171958	0.00312606	0.00171958	5.10047E-08	0.0409996	8.82E-05	29.6797459
Waist circumference	Falls	rs1922879	7	A	G	0.00228302	0.00125497	0.00228302	0.00125497	3.99871E-07	0.49	7.63E-05	25.69596717
Waist circumference	Falls	rs2188306	7	A	G	0.00391319	0.00214002	0.00391319	0.00214002	3.54691E-07	0.032	7.7E-05	25.92751806
Waist circumference	Falls	rs2289379	7	T	C	0.00220747	0.00121436	0.00220747	0.00121436	1.05495E-07	0.14	8.4E-05	28.27184754
Waist circumference	Falls	rs2404324	7	G	A	0.00297384	0.00163545	0.00297384	0.00163545	8.48399E-11	0.23	0.000125	42.14576152
Waist circumference	Falls	rs4718964	7	T	G	0.00219068	0.00120501	0.00219068	0.00120501	2.49058E-11	0.0479999	0.000132	44.54411418
Waist circumference	Falls	rs4722398	7	T	C	0.00313148	0.00172079	0.00313148	0.00172079	3.70493E-09	0.22	0.000103	34.77484417
Waist circumference	Falls	rs541577	7	G	A	0.00222647	0.00122256	0.00222647	0.00122256	4.66101E-08	0.00719996	8.87E-05	29.85415691
Waist circumference	Falls	rs58862095	7	T	C	0.0021844	0.00120149	0.0021844	0.00120149	1.34865E-16	0.87	0.000203	68.38693349
Waist circumference	Falls	rs73068448	7	T	C	0.00289425	0.00159569	0.00289425	0.00159569	3.46553E-07	0.02	7.71E-05	25.97204029
Waist circumference	Falls	rs10100245	8	A	G	0.00216949	0.0011939	0.00216949	0.0011939	4.07662E-12	0.8	0.000143	48.09094438
Waist circumference	Falls	rs10957088	8	C	T	0.00293621	0.00161578	0.00293621	0.00161578	2.68436E-08	0.37	9.19E-05	30.9244793
Waist circumference	Falls	rs12679106	8	T	G	0.00238599	0.00130851	0.00238599	0.00130851	4.70869E-20	0.97	0.00025	84.10866863
Waist circumference	Falls	rs12680342	8	G	T	0.00255577	0.00140574	0.00255577	0.00140574	1.99982E-08	0.55	9.36E-05	31.49594389
Waist circumference	Falls	rs13264909	8	T	A	0.00217545	0.00119713	0.00217545	0.00119713	5.49921E-09	0.709999	0.000101	34.00619563
Waist circumference	Falls	rs1559900	8	T	C	0.00238216	0.00130851	0.00238216	0.00130851	4.62424E-08	0.13	8.87E-05	29.86941669
Waist circumference	Falls	rs1566085	8	T	G	0.00217593	0.00119767	0.00217593	0.00119767	4.35311E-09	0.81	0.000102	34.46062778
Waist circumference	Falls	rs17716502	8	T	C	0.00267351	0.0014784	0.00267351	0.0014784	2.23306E-11	0.0580003	0.000133	44.75819247
Waist circumference	Falls	rs2725371	8	G	A	0.00234606	0.00129105	0.00234606	0.00129105	2.16272E-14	0.0239999	0.000173	58.38328862
Waist circumference	Falls	rs2919389	8	T	C	0.00220486	0.00121326	0.00220486	0.00121326	2.23991E-07	0.42	7.96E-05	26.81513595
Waist circumference	Falls	rs4072917	8	A	G	0.00216259	0.00118981	0.00216259	0.00118981	1.5037E-08	0.35	9.52E-05	32.04994718
Waist circumference	Falls	rs4623474	8	A	G	0.0033222	0.00182456	0.0033222	0.00182456	6.45535E-08	0.54	8.68E-05	29.22284039
Waist circumference	Falls	rs59104534	8	T	C	0.00235384	0.00129728	0.00235384	0.00129728	1.73856E-07	0.32	8.11E-05	27.30500258
Waist circumference	Falls	rs7827410	8	C	A	0.00293537	0.00161454	0.00293537	0.00161454	4.62115E-07	0.87	7.55E-05	25.41683267
Waist circumference	Falls	rs1019240	9	T	A	0.00225438	0.00123924	0.00225438	0.00123924	2.32906E-08	0.29	9.27E-05	31.20058777

Waist circumference	Falls	rs10992841	9	T	C	0.00232451	0.00127509	0.00232451	0.00127509	4.31688E-09	0.0309999	0.000102	34.47723794
Waist circumference	Falls	rs12001437	9	C	T	0.00223129	0.00122665	0.00223129	0.00122665	4.18659E-07	0.38	7.61E-05	25.60710424
Waist circumference	Falls	rs12335914	9	C	G	0.0021576	0.00118665	0.0021576	0.00118665	1.09408E-10	0.00669993	0.000124	41.64843036
Waist circumference	Falls	rs1411432	9	C	A	0.00278338	0.00152495	0.00278338	0.00152495	3.51641E-09	0.54	0.000104	34.87641295
Waist circumference	Falls	rs1412239	9	G	C	0.00229433	0.001263	0.00229433	0.001263	8.13392E-18	0.46	0.00022	73.92823222
Waist circumference	Falls	rs16916303	9	G	A	0.00334806	0.00184197	0.00334806	0.00184197	8.00221E-08	0.48	8.56E-05	28.80681137
Waist circumference	Falls	rs1752169	9	A	C	0.0024928	0.00136653	0.0024928	0.00136653	1.89557E-09	0.94	0.000107	36.08043641
Waist circumference	Falls	rs2417998	9	G	C	0.00236952	0.00130702	0.00236952	0.00130702	2.01002E-11	0.36	0.000134	44.96420364
Waist circumference	Falls	rs2482704	9	T	G	0.00217695	0.00119426	0.00217695	0.00119426	2.68992E-09	0.4	0.000105	35.39838668
Waist circumference	Falls	rs4741546	9	T	C	0.0022059	0.00121515	0.0022059	0.00121515	2.38451E-12	0.08	0.000146	49.1429029
Waist circumference	Falls	rs10787738	10	T	C	0.00250799	0.00137909	0.00250799	0.00137909	3.17103E-12	0.19	0.000144	48.58373307
Waist circumference	Falls	rs10887578	10	C	G	0.00216271	0.0011902	0.00216271	0.0011902	1.65699E-07	0.00309999	8.14E-05	27.39802741
Waist circumference	Falls	rs10995427	10	A	G	0.00225224	0.00123792	0.00225224	0.00123792	7.90751E-08	0.0109999	8.56E-05	28.83008705
Waist circumference	Falls	rs11012732	10	G	A	0.00228772	0.00125718	0.00228772	0.00125718	3.26738E-21	0.5	0.000265	89.38551432
Waist circumference	Falls	rs11594905	10	A	G	0.00312358	0.00172411	0.00312358	0.00172411	5.95539E-08	0.00969996	8.73E-05	29.37916387
Waist circumference	Falls	rs17399739	10	G	A	0.00422808	0.00233901	0.00422808	0.00233901	9.4367E-08	0.0259998	8.46E-05	28.48744947
Waist circumference	Falls	rs2172131	10	C	T	0.00218023	0.00119897	0.00218023	0.00119897	1.35379E-10	0.00389996	0.000122	41.2318514
Waist circumference	Falls	rs2439823	10	G	A	0.00216629	0.00119119	0.00216629	0.00119119	2.6687E-13	0.91	0.000159	53.44230981
Waist circumference	Falls	rs2492462	10	G	A	0.00285442	0.0015726	0.00285442	0.0015726	2.13781E-08	0.82	9.32E-05	31.36685907
Waist circumference	Falls	rs35972789	10	A	C	0.00563553	0.00311334	0.00563553	0.00311334	3.05971E-08	0.2	9.11E-05	30.67069176
Waist circumference	Falls	rs61871615	10	T	C	0.00391527	0.00214921	0.00391527	0.00214921	1.45502E-07	0.95	8.21E-05	27.64954634
Waist circumference	Falls	rs7094644	10	A	G	0.00233716	0.00128917	0.00233716	0.00128917	6.04617E-10	0.31	0.000114	38.30857647
Waist circumference	Falls	rs71495049	10	A	G	0.00387652	0.00213721	0.00387652	0.00213721	3.87391E-09	0.15	0.000103	34.68814115
Waist circumference	Falls	rs10128597	11	A	G	0.00242631	0.00133183	0.00242631	0.00133183	1.79639E-11	0.37	0.000134	45.18386653
Waist circumference	Falls	rs10898330	11	T	C	0.00216356	0.0011899	0.00216356	0.0011899	1.1471E-07	0.91	8.35E-05	28.10926748
Waist circumference	Falls	rs11039266	11	G	T	0.00239802	0.00131738	0.00239802	0.00131738	1.5574E-19	0.51	0.000243	81.74346014

Waist circumference	Falls	rs11601136	11	C	T	0.00218558	0.0012029	0.00218558	0.0012029	1.02989E-07	0.3	8.41E-05	28.31805074
Waist circumference	Falls	rs11824092	11	C	T	0.0022508	0.00123738	0.0022508	0.00123738	2.51739E-09	0.51	0.000106	35.52759521
Waist circumference	Falls	rs12806052	11	T	C	0.00289848	0.00160133	0.00289848	0.00160133	7.01455E-11	0.08	0.000126	42.51764535
Waist circumference	Falls	rs35023999	11	C	A	0.00215371	0.00118373	0.00215371	0.00118373	9.44996E-08	0.9	8.46E-05	28.4848067
Waist circumference	Falls	rs3802858	11	C	T	0.00217553	0.00119556	0.00217553	0.00119556	3.0162E-08	0.0389996	9.12E-05	30.69854624
Waist circumference	Falls	rs58568715	11	G	A	0.00291834	0.00160811	0.00291834	0.00160811	3.30157E-09	0.34	0.000104	34.99905827
Waist circumference	Falls	rs59227842	11	G	A	0.00234316	0.00128836	0.00234316	0.00128836	1.90678E-16	0.35	0.000201	67.70405403
Waist circumference	Falls	rs61888762	11	G	C	0.00230029	0.00126754	0.00230029	0.00126754	1.2782E-31	0.0005	0.000407	136.9114346
Waist circumference	Falls	rs61903695	11	G	A	0.00246411	0.00135918	0.00246411	0.00135918	4.0155E-07	0.82	7.63E-05	25.68787587
Waist circumference	Falls	rs7117842	11	C	T	0.0022369	0.00123163	0.0022369	0.00123163	3.34503E-07	0.27	7.73E-05	26.04060581
Waist circumference	Falls	rs72915955	11	A	G	0.00293417	0.00161934	0.00293417	0.00161934	3.63195E-07	0.17	7.69E-05	25.88165104
Waist circumference	Falls	rs7925100	11	A	G	0.00219878	0.00121005	0.00219878	0.00121005	4.2715E-10	0.92	0.000116	38.98706846
Waist circumference	Falls	rs7930006	11	T	C	0.002166	0.00119117	0.002166	0.00119117	6.62171E-10	0.56	0.000113	38.13091009
Waist circumference	Falls	rs7948120	11	T	C	0.00246739	0.00135497	0.00246739	0.00135497	1.01555E-08	0.0280001	9.75E-05	32.81272378
Waist circumference	Falls	rs7952436	11	T	C	0.00389664	0.00215593	0.00389664	0.00215593	2.3206E-11	0.68	0.000133	44.68291738
Waist circumference	Falls	rs868784	11	A	G	0.00222281	0.00122207	0.00222281	0.00122207	3.17695E-08	0.43	9.09E-05	30.59812177
Waist circumference	Falls	rs12367809	12	T	C	0.00223898	0.00123032	0.00223898	0.00123032	2.07683E-22	0.44	0.000282	94.84093344
Waist circumference	Falls	rs12817084	12	C	T	0.00349746	0.00192524	0.00349746	0.00192524	8.56052E-08	0.18	8.52E-05	28.67633643
Waist circumference	Falls	rs147786161	12	G	A	0.00218105	0.00119897	0.00218105	0.00119897	4.8908E-08	0.99	8.84E-05	29.76088806
Waist circumference	Falls	rs1901241	12	G	A	0.00294595	0.00161748	0.00294595	0.00161748	4.67994E-07	0.0739997	7.54E-05	25.39240345
Waist circumference	Falls	rs1904387	12	T	A	0.00246018	0.00134978	0.00246018	0.00134978	2.62011E-08	0.32	9.2E-05	30.97147938
Waist circumference	Falls	rs2012464	12	G	A	0.00227764	0.00125304	0.00227764	0.00125304	3.31444E-07	0.55	7.74E-05	26.05854971
Waist circumference	Falls	rs2242259	12	C	T	0.00216726	0.00119281	0.00216726	0.00119281	5.08159E-11	0.0309999	0.000128	43.14854248
Waist circumference	Falls	rs2373980	12	A	T	0.00218603	0.00120335	0.00218603	0.00120335	7.07799E-08	0.0129999	8.63E-05	29.04469512
Waist circumference	Falls	rs2608703	12	A	C	0.00215852	0.00118757	0.00215852	0.00118757	4.81615E-11	0.28	0.000128	43.25335775
Waist circumference	Falls	rs3764002	12	T	C	0.00244769	0.00134553	0.00244769	0.00134553	7.753E-10	0.08	0.000112	37.8235377

Waist circumference	Falls	rs55726687	12	A	G	0.00263446	0.0014515	0.00263446	0.0014515	1.0067E-11	0.85	0.000138	46.31887299
Waist circumference	Falls	rs56362718	12	C	T	0.00232596	0.00128197	0.00232596	0.00128197	8.46447E-11	0.53	0.000125	42.15034946
Waist circumference	Falls	rs704061	12	C	T	0.00216115	0.0011879	0.00216115	0.0011879	4.79292E-14	0.0350002	0.000169	56.81761638
Waist circumference	Falls	rs76895963	12	G	T	0.00833756	0.0045803	0.00833756	0.0045803	4.89215E-11	0.6	0.000128	43.22280717
Waist circumference	Falls	rs76929617	12	G	A	0.00556915	0.00306517	0.00556915	0.00306517	1.04256E-14	0.14	0.000178	59.8194608
Waist circumference	Falls	rs894736	12	G	A	0.00224469	0.001235	0.00224469	0.001235	5.22516E-15	0.18	0.000182	61.17904822
Waist circumference	Falls	rs1218824	13	A	G	0.00227409	0.00125094	0.00227409	0.00125094	1.33248E-08	0.53	9.59E-05	32.28522704
Waist circumference	Falls	rs12877270	13	A	G	0.00218377	0.00119951	0.00218377	0.00119951	9.98114E-10	0.0879995	0.000111	37.33078667
Waist circumference	Falls	rs1379828	13	T	C	0.00268642	0.00147811	0.00268642	0.00147811	5.41814E-10	0.36	0.000114	38.52264888
Waist circumference	Falls	rs1441264	13	A	G	0.00223532	0.00123026	0.00223532	0.00123026	5.82237E-13	0.89	0.000154	51.91021613
Waist circumference	Falls	rs17060974	13	G	T	0.00256015	0.00140601	0.00256015	0.00140601	4.70533E-08	0.2	8.86E-05	29.83583652
Waist circumference	Falls	rs1928496	13	T	C	0.00245655	0.00135321	0.00245655	0.00135321	3.94994E-09	0.51	0.000103	34.65001206
Waist circumference	Falls	rs2121058	13	C	T	0.00256163	0.0014121	0.00256163	0.0014121	3.60579E-12	0.18	0.000144	48.3316514
Waist circumference	Falls	rs525101	13	C	T	0.00222956	0.00122717	0.00222956	0.00122717	6.82009E-09	0.00129999	9.98E-05	33.5872225
Waist circumference	Falls	rs9522279	13	T	C	0.00218009	0.00119973	0.00218009	0.00119973	1.96811E-09	0.32	0.000107	36.00693583
Waist circumference	Falls	rs9528841	13	A	T	0.00257213	0.0014145	0.00257213	0.0014145	1.13297E-07	0.16	8.36E-05	28.13374599
Waist circumference	Falls	rs10146816	14	G	A	0.0042027	0.00233036	0.0042027	0.00233036	1.52265E-07	0.56	8.19E-05	27.56156311
Waist circumference	Falls	rs12881629	14	G	A	0.00390935	0.00214542	0.00390935	0.00214542	1.62708E-08	0.94	9.47E-05	31.89670022
Waist circumference	Falls	rs17115183	14	T	C	0.00219929	0.00121019	0.00219929	0.00121019	2.38046E-07	0.11	7.93E-05	26.69774132
Waist circumference	Falls	rs217671	14	G	A	0.00241684	0.00132958	0.00241684	0.00132958	1.034E-08	0.0589997	9.74E-05	32.7779512
Waist circumference	Falls	rs2370982	14	T	C	0.0026361	0.00145183	0.0026361	0.00145183	6.461E-15	0.00259998	0.00018	60.76143655
Waist circumference	Falls	rs3803286	14	G	A	0.00227816	0.0012538	0.00227816	0.0012538	2.74979E-13	0.24	0.000159	53.38381307
Waist circumference	Falls	rs4981693	14	A	G	0.00256987	0.00141396	0.00256987	0.00141396	7.20444E-12	0.33	0.00014	46.97417436
Waist circumference	Falls	rs61992671	14	G	A	0.00224606	0.00123655	0.00224606	0.00123655	8.5426E-09	0.00109999	9.85E-05	33.14936789
Waist circumference	Falls	rs6575340	14	A	G	0.00224179	0.00123225	0.00224179	0.00123225	5.77032E-15	0.22	0.000181	60.98369892
Waist circumference	Falls	rs7154982	14	A	G	0.00242686	0.00133326	0.00242686	0.00133326	4.79148E-10	1	0.000115	38.76240714

Waist circumference	Falls	rs72681698	14	C	T	0.0103613	0.00573944	0.0103613	0.00573944	3.89736E-07	0.14	7.65E-05	25.74544055
Waist circumference	Falls	rs12102086	15	A	G	0.0026132	0.00144213	0.0026132	0.00144213	2.09604E-11	0.22	0.000133	44.88159078
Waist circumference	Falls	rs138847555	15	C	T	0.00858346	0.0047378	0.00858346	0.0047378	1.10268E-07	0.0629999	8.37E-05	28.18598925
Waist circumference	Falls	rs17296856	15	C	A	0.00239446	0.00131845	0.00239446	0.00131845	7.61605E-08	0.1	8.58E-05	28.90264697
Waist circumference	Falls	rs2470167	15	A	G	0.00267827	0.00147317	0.00267827	0.00147317	2.84132E-08	0.0369999	9.15E-05	30.81452634
Waist circumference	Falls	rs2682909	15	C	G	0.00224186	0.00123057	0.00224186	0.00123057	2.06962E-07	0.83	8.01E-05	26.96827294
Waist circumference	Falls	rs34994596	15	C	T	0.00234759	0.00129367	0.00234759	0.00129367	5.2942E-12	0.0980009	0.000141	47.57843252
Waist circumference	Falls	rs3784692	15	T	C	0.0021979	0.0012065	0.0021979	0.0012065	6.40767E-19	0.93	0.000234	78.94774053
Waist circumference	Falls	rs56803094	15	G	A	0.0025732	0.00141735	0.0025732	0.00141735	9.26403E-10	0.450001	0.000111	37.47588444
Waist circumference	Falls	rs7171864	15	A	G	0.00228283	0.00125421	0.00228283	0.00125421	2.44028E-09	0.32	0.000106	35.58811865
Waist circumference	Falls	rs7183417	15	T	C	0.00217352	0.0011951	0.00217352	0.0011951	1.56585E-07	0.87	8.17E-05	27.50755207
Waist circumference	Falls	rs11642015	16	T	C	0.00219182	0.00120555	0.00219182	0.00120555	1.4093E-145	0.00017	0.001959	660.7626675
Waist circumference	Falls	rs11646719	16	G	C	0.00228695	0.00125648	0.00228695	0.00125648	2.99813E-07	0.000420001	7.8E-05	26.25209685
Waist circumference	Falls	rs12103006	16	G	A	0.00217666	0.00119555	0.00217666	0.00119555	1.21367E-12	0.6	0.00015	50.46751679
Waist circumference	Falls	rs12926311	16	C	G	0.00225378	0.00124141	0.00225378	0.00124141	3.11293E-10	0.0449997	0.000118	39.60499006
Waist circumference	Falls	rs13333747	16	C	T	0.00280123	0.00153836	0.00280123	0.00153836	2.07062E-16	0.16	0.000201	67.54075702
Waist circumference	Falls	rs2032912	16	T	G	0.00219463	0.00120694	0.00219463	0.00120694	2.31047E-15	0.0369999	0.000186	62.78654005
Waist circumference	Falls	rs2660241	16	C	T	0.00224209	0.00123039	0.00224209	0.00123039	6.38117E-11	0.4	0.000127	42.70297139
Waist circumference	Falls	rs3814883	16	T	C	0.00215875	0.00118728	0.00215875	0.00118728	2.08545E-38	0.99	0.000499	167.9808117
Waist circumference	Falls	rs629471	16	G	A	0.00237824	0.00130567	0.00237824	0.00130567	1.58573E-07	0.5	8.16E-05	27.48282223
Waist circumference	Falls	rs71396924	16	T	G	0.00271593	0.00149418	0.00271593	0.00149418	2.25372E-07	0.760001	7.96E-05	26.80335106
Waist circumference	Falls	rs7206608	16	G	C	0.00230276	0.00126736	0.00230276	0.00126736	1.39463E-08	0.016	9.56E-05	32.19644317
Waist circumference	Falls	rs72793809	16	T	C	0.00219042	0.00120619	0.00219042	0.00120619	9.09913E-34	0.57	0.000436	146.7385118
Waist circumference	Falls	rs7500458	16	G	A	0.00237307	0.00130438	0.00237307	0.00130438	2.7165E-07	0.29	7.85E-05	26.44265182
Waist circumference	Falls	rs756717	16	A	G	0.0022219	0.00122281	0.0022219	0.00122281	2.71844E-07	0.53	7.85E-05	26.44104434
Waist circumference	Falls	rs879620	16	T	C	0.00221895	0.00121845	0.00221895	0.00121845	3.12536E-21	0.51	0.000266	89.47482905

Waist circumference	Falls	rs11078883	17	G	C	0.00226725	0.00124796	0.00226725	0.00124796	4.3417E-07	0.59	7.59E-05	25.53720305
Waist circumference	Falls	rs11150745	17	G	A	0.00230943	0.00127228	0.00230943	0.00127228	1.33168E-12	0.69	0.000149	50.28554722
Waist circumference	Falls	rs11653367	17	G	A	0.00230317	0.00126622	0.00230317	0.00126622	5.10975E-15	0.27	0.000182	61.22311508
Waist circumference	Falls	rs1914888	17	G	A	0.00215881	0.00118631	0.00215881	0.00118631	1.81443E-08	0.48	9.41E-05	31.68542463
Waist circumference	Falls	rs2020942	17	T	C	0.00220343	0.00121266	0.00220343	0.00121266	3.92808E-07	0.4	7.64E-05	25.73026235
Waist circumference	Falls	rs208015	17	C	T	0.004285	0.00234653	0.004285	0.00234653	1.91161E-13	0.87	0.000161	54.09817059
Waist circumference	Falls	rs2306593	17	T	C	0.00215553	0.00118581	0.00215553	0.00118581	4.02717E-15	0.61	0.000183	61.69163702
Waist circumference	Falls	rs35850753	17	T	C	0.00778073	0.00433805	0.00778073	0.00433805	9.57084E-08	0.7	8.45E-05	28.46017551
Waist circumference	Falls	rs3826408	17	T	C	0.0021584	0.00118628	0.0021584	0.00118628	8.63635E-09	0.98	9.84E-05	33.12806713
Waist circumference	Falls	rs4790841	17	T	C	0.00298062	0.00164297	0.00298062	0.00164297	3.10527E-17	0.43	0.000212	71.28382603
Waist circumference	Falls	rs62071997	17	C	T	0.00261513	0.00143881	0.00261513	0.00143881	3.15203E-10	0.32	0.000118	39.58013155
Waist circumference	Falls	rs80135947	17	C	A	0.00271445	0.00149298	0.00271445	0.00149298	2.19634E-20	0.0290001	0.000254	85.61660561
Waist circumference	Falls	rs9916444	17	G	C	0.0022766	0.00124865	0.0022766	0.00124865	5.35649E-08	0.450001	8.79E-05	29.58450138
Waist circumference	Falls	rs1652376	18	T	G	0.00215595	0.00118637	0.00215595	0.00118637	1.58234E-19	0.93	0.000243	81.71163762
Waist circumference	Falls	rs1834144	18	A	C	0.00222893	0.00122617	0.00222893	0.00122617	4.77309E-11	0.0109999	0.000129	43.27125539
Waist circumference	Falls	rs1942826	18	A	G	0.00326444	0.00178371	0.00326444	0.00178371	2.71951E-10	0.53	0.000118	39.86901701
Waist circumference	Falls	rs57636386	18	C	T	0.00389755	0.00214141	0.00389755	0.00214141	4.3481E-17	0.31	0.00021	70.61984104
Waist circumference	Falls	rs66922415	18	G	A	0.00253565	0.00139822	0.00253565	0.00139822	1.45144E-65	0.11	0.000868	292.5824652
Waist circumference	Falls	rs7239114	18	A	G	0.00217368	0.00119619	0.00217368	0.00119619	6.24065E-09	0.0920005	0.0001	33.75994479
Waist circumference	Falls	rs8097672	18	T	A	0.00306494	0.00168716	0.00306494	0.00168716	2.99282E-09	0.28	0.000105	35.19046625
Waist circumference	Falls	rs10423928	19	A	T	0.00271482	0.00149218	0.00271482	0.00149218	9.07194E-22	0.99	0.000273	91.92272193
Waist circumference	Falls	rs111640872	19	C	G	0.00229116	0.00126113	0.00229116	0.00126113	1.24022E-17	0.62	0.000217	73.0956487
Waist circumference	Falls	rs11666480	19	G	C	0.00216606	0.00119286	0.00216606	0.00119286	9.28966E-13	0.54	0.000151	50.99233509
Waist circumference	Falls	rs11878477	19	G	A	0.00215797	0.00118805	0.00215797	0.00118805	1.94446E-11	0.82	0.000134	45.02920765
Waist circumference	Falls	rs1964415	19	C	T	0.00234357	0.00128796	0.00234357	0.00128796	9.2841E-08	0.760001	8.47E-05	28.51895507
Waist circumference	Falls	rs2903738	19	T	A	0.00259614	0.00142429	0.00259614	0.00142429	2.57745E-09	0.26	0.000105	35.48123259

Waist circumference	Falls	rs350832	19	A	G	0.00257337	0.00141478	0.00257337	0.00141478	3.95421E-10	0.23	0.000116	39.13752278
Waist circumference	Falls	rs35343344	19	A	C	0.00247495	0.00136184	0.00247495	0.00136184	9.81296E-12	0.0189998	0.000138	46.36889392
Waist circumference	Falls	rs429358	19	C	T	0.00296137	0.00163843	0.00296137	0.00163843	3.19154E-16	0.00025	0.000198	66.68825807
Waist circumference	Falls	rs62120394	19	A	G	0.00237797	0.00130526	0.00237797	0.00130526	2.33668E-18	0.0840001	0.000227	76.39158566
Waist circumference	Falls	rs7259070	19	C	T	0.0022141	0.00121604	0.0022141	0.00121604	4.39845E-15	0.13	0.000183	61.51841387
Waist circumference	Falls	rs1056441	20	C	T	0.00229345	0.0012635	0.00229345	0.0012635	2.34617E-08	0.0460002	9.26E-05	31.18629193
Waist circumference	Falls	rs11474838	20	G	T	0.00221385	0.00121799	0.00221385	0.00121799	1.66633E-09	0.00560003	0.000108	36.33140492
Waist circumference	Falls	rs6030803	20	C	T	0.0032673	0.00178524	0.0032673	0.00178524	6.46249E-08	0.28	8.68E-05	29.2207864
Waist circumference	Falls	rs13047416	21	G	C	0.00222813	0.00122344	0.00222813	0.00122344	2.95413E-09	0.91	0.000105	35.21595985
Waist circumference	Falls	rs76040172	21	A	G	0.00475228	0.0026329	0.00475228	0.0026329	1.26241E-12	0.33	0.00015	50.39091421
Waist circumference	Falls	rs139915	22	T	C	0.00219324	0.00120729	0.00219324	0.00120729	2.33733E-07	0.0025	7.94E-05	26.73290369
Waist circumference	Falls	rs444102	22	C	G	0.00285704	0.00157117	0.00285704	0.00157117	1.81937E-07	0.02	8.08E-05	27.21717051
Waist circumference	Falls	rs9610311	22	C	T	0.00239488	0.00131726	0.00239488	0.00131726	1.07409E-08	0.8	9.71E-05	32.70400605
Waist-hip ratio	Falls	rs1409158	1	T	C	0.002	0.000934104	3.00E-04	0.00139275	2.92E-09	0.5	0.000517	44.44444444
Waist-hip ratio	Falls	rs2810894	1	G	A	0.0017	0.00199878	3.00E-04	0.00127672	1.24E-08	0.12	0.000373	32.11111111
Waist-hip ratio	Falls	rs10933390	2	A	G	-0.0015	0.000766885	3.00E-04	0.00124087	3.93E-07	0.54	0.000298	25.6442684
Waist-hip ratio	Falls	rs13389219	2	T	C	-0.0024	0.0010595	3.00E-04	0.0012107	2.34E-16	0.38	0.000744	64
Waist-hip ratio	Falls	rs4132228	3	T	C	-0.0019	0.000538139	3.00E-04	0.001299	1.46E-09	0.68	0.000466	40.11111111
Waist-hip ratio	Falls	rs78125783	4	A	C	-0.0055	0.00433135	0.001	0.00445488	1.69E-07	0.33	0.000373	32.10911381
Waist-hip ratio	Falls	rs11154375	6	G	A	-0.0015	-0.000373123	3.00E-04	0.00118413	7.01E-08	0.75	0.000291	25
Waist-hip ratio	Falls	rs115675705	6	G	A	0.0056	0.00302765	9.00E-04	0.00320061	5.93E-11	0.34	0.000478	41.09547226
Waist-hip ratio	Falls	rs2249742	6	T	C	0.002	0.00125544	4.00E-04	0.00118677	1.40E-07	0.29	0.000483	41.57527225
Waist-hip ratio	Falls	rs4711750	6	A	T	0.0024	-0.00349747	3.00E-04	0.00118232	3.92E-17	0.00309999	0.000763	65.6493271
Waist-hip ratio	Falls	rs72959041	6	A	G	0.0089	-0.00545675	6.00E-04	0.00277501	1.25E-43	0.0490004	0.002709	233.5503126
Waist-hip ratio	Falls	rs10270542	7	G	A	-0.0015	0.00180584	3.00E-04	0.00119971	3.95E-07	0.13	0.000291	25
Waist-hip ratio	Falls	rs1534696	7	A	C	-0.0017	-1.44E-05	3.00E-04	0.00118571	9.08E-09	0.99	0.000373	32.11111111

Waist-hip ratio	Falls	rs6968925	7	T	C	0.0017	-0.00064826	3.00E-04	0.00126906	9.70E-08	0.61	0.000383	32.93863807
Waist-hip ratio	Falls	rs10820747	9	A	G	0.0021	-0.00183088	3.00E-04	0.00139326	7.06E-10	0.19	0.00057	49
Waist-hip ratio	Falls	rs148806124	9	T	G	-0.0067	-0.00725282	0.0013	0.00541083	3.14E-07	0.18	0.000374	32.20751
Waist-hip ratio	Falls	rs1027087	12	A	T	0.0017	-0.00232615	3.00E-04	0.00137778	3.78E-07	0.0909997	0.000373	32.11111111
Waist-hip ratio	Falls	rs1838173	12	C	T	-0.0021	0.000275712	4.00E-04	0.00171518	2.16E-07	0.87	0.00032	27.5625
Waist-hip ratio	Falls	rs2292137	12	G	C	0.0018	-0.00153088	3.00E-04	0.00121897	3.41E-09	0.21	0.000434	37.30697652
Waist-hip ratio	Falls	rs7133378	12	A	G	-0.0024	0.00256415	3.00E-04	0.00127127	5.79E-15	0.0439997	0.000744	64
Waist-hip ratio	Falls	rs549058	13	T	G	-0.0023	-0.00052947	4.00E-04	0.00181872	4.67E-08	0.77	0.000387	33.2625177
Waist-hip ratio	Falls	rs3803459	15	C	T	0.0023	0.000733706	4.00E-04	0.00181003	8.82E-08	0.69	0.00039	33.54870176
Waist-hip ratio	Falls	rs4923910	15	C	G	0.0016	0.000328673	3.00E-04	0.00122528	7.86E-08	0.79	0.000331	28.44444444
Waist-hip ratio	Falls	rs1469024	19	T	C	-0.0022	0.000889619	3.00E-04	0.00122768	5.93E-14	0.47	0.000625	53.77777778
Waist-hip ratio	Falls	rs17833947	19	C	A	0.002	-0.000781379	4.00E-04	0.00161623	1.34E-07	0.630001	0.000291	25
Waist-hip ratio	Falls	rs6068191	20	A	C	0.0017	0.00139979	3.00E-04	0.00121761	6.13E-09	0.25	0.000373	32.11111111
Rheumatoid Arthritis	Falls	rs4512588	1	C	T	0.000972395	0.000761376	1080927	0.00173306	1	0.66	6.71E-05	30.98712704
Rheumatoid Arthritis	Falls	rs741384	2	G	C	0.000608882	-0.000878097	217551954	0.00120051	2	0.46	5.48E-05	25.32226476
Rheumatoid Arthritis	Falls	rs17612712	6	T	A	0.00145753	0.00297587	32615945	0.00128469	6	0.021	0.000274	126.7629119
Rheumatoid Arthritis	Falls	rs3104415	6	C	A	0.00213886	0.00284109	32582577	0.0012508	6	0.0230001	0.000623	287.9154452
Sleeplessness	Falls	rs12049261	1	C	G	0.0111868	0.00209705	0.00163048	0.00129964	6.79986E-12	0.11	0.000102	47.07396667
Sleeplessness	Falls	rs2644128	1	G	C	0.0106284	0.0024163	0.00149103	0.00118855	1E-12	0.0420001	0.00011	50.81161726
Sleeplessness	Falls	rs2803296	1	C	G	-0.00862206	0.00116524	0.00149047	0.0011881	7.29995E-09	0.33	7.24E-05	33.46382677
Sleeplessness	Falls	rs35267450	1	C	T	0.00892042	0.0021229	0.00164053	0.00130775	5.39995E-08	0.1	6.39E-05	29.56665898
Sleeplessness	Falls	rs6690017	1	G	T	-0.0102669	-0.00309946	0.00151022	0.00120378	1.10002E-11	0.01	1E-04	46.21662517
Sleeplessness	Falls	rs76265753	1	T	C	0.0211056	0.00450731	0.00399311	0.00318254	1.29999E-07	0.16	6.04E-05	27.93655551
Sleeplessness	Falls	rs113851554	2	T	G	0.0467802	-0.000633962	0.00331329	0.00264162	2.90001E-45	0.81	0.000431	199.344955
Sleeplessness	Falls	rs12470989	2	G	A	-0.0102429	-0.00115316	0.00184485	0.00147042	2.80001E-08	0.43	6.67E-05	30.82646636
Sleeplessness	Falls	rs1867814	2	A	G	-0.00767576	0.00115609	0.0014862	0.00118467	2.39999E-07	0.33	5.77E-05	26.67400696

Sleeplessness	Falls	rs4572538	2	T	C	-0.00960596	0.000470514	0.00156161	0.00124482	7.69999E-10	0.709999	8.18E-05	37.83871548
Sleeplessness	Falls	rs4577309	2	G	A	-0.00854833	0.000755894	0.00149214	0.00118936	0.00000001	0.53	7.1E-05	32.8203655
Sleeplessness	Falls	rs56093896	2	A	C	-0.0124111	-0.000747358	0.00181352	0.00144559	7.70016E-12	0.61	0.000101	46.83557439
Sleeplessness	Falls	rs56365214	2	A	C	-0.0147948	-0.00179138	0.00205225	0.00163571	5.60015E-13	0.27	0.000112	51.97059256
Sleeplessness	Falls	rs6543087	2	T	A	0.00766484	0.000896866	0.00151726	0.00120937	4.39997E-07	0.46	5.52E-05	25.5203232
Sleeplessness	Falls	rs78887635	2	C	T	-0.0116184	-0.00145037	0.00224665	0.00179068	2.30001E-07	0.42	5.78E-05	26.74371951
Sleeplessness	Falls	rs2014830	3	T	C	-0.0116018	-0.00330279	0.0016233	0.00129365	8.9002E-13	0.0109999	0.00011	51.08026854
Sleeplessness	Falls	rs75709417	3	T	C	0.0123444	-0.000284602	0.00228118	0.00181801	6.29999E-08	0.88	6.33E-05	29.28335694
Sleeplessness	Falls	rs7625896	3	G	A	-0.00819859	-0.00196286	0.00156815	0.0012496	0.00000017	0.12	5.91E-05	27.33399636
Sleeplessness	Falls	rs9845387	3	A	C	-0.0218562	-0.00458394	0.00377648	0.0030094	7.10003E-09	0.13	7.24E-05	33.49461068
Sleeplessness	Falls	rs11097861	4	G	A	0.0100437	0.00233971	0.00164946	0.0013142	1.09999E-09	0.0749998	8.02E-05	37.07694031
Sleeplessness	Falls	rs1988337	4	G	A	0.00838726	0.000937391	0.00149645	0.00119239	0.000000021	0.43	6.79E-05	31.41346124
Sleeplessness	Falls	rs2604551	4	G	T	-0.00848084	-0.00170107	0.00155238	0.0012371	4.70002E-08	0.17	6.45E-05	29.84569262
Sleeplessness	Falls	rs6833329	4	G	A	-0.00820472	-0.000552347	0.00153119	0.00122023	8.40001E-08	0.649999	6.21E-05	28.7123911
Sleeplessness	Falls	rs1083562	5	C	T	-0.00855653	0.00256161	0.0016541	0.00131792	2.30001E-07	0.0519996	5.79E-05	26.75912027
Sleeplessness	Falls	rs1430205	5	T	C	0.00947493	-8.14949E-05	0.00149107	0.00118807	2.1E-10	0.95	8.73E-05	40.37903684
Sleeplessness	Falls	rs2270914	5	T	C	-0.00922378	-0.00203667	0.00175421	0.00139791	0.00000015	0.15	5.98E-05	27.64742617
Sleeplessness	Falls	rs36116812	5	C	T	0.00777747	0.00371789	0.00148243	0.00118123	0.00000016	0.0016	5.95E-05	27.52506154
Sleeplessness	Falls	rs7711696	5	T	G	0.0111716	0.00018706	0.00161138	0.00128395	4.10015E-12	0.88	0.000104	48.06564967
Sleeplessness	Falls	rs10947690	6	G	A	0.00919149	0.0034784	0.00169355	0.00134939	5.69994E-08	0.00990011	6.37E-05	29.45613704
Sleeplessness	Falls	rs11963889	6	C	T	-0.0075944	0.00159853	0.00148676	0.00118472	3.29997E-07	0.18	5.64E-05	26.09186959
Sleeplessness	Falls	rs17466209	6	T	A	0.00884283	-0.000187231	0.00166381	0.00132572	1.09999E-07	0.89	6.11E-05	28.24717961
Sleeplessness	Falls	rs240112	6	A	C	-0.0076731	0.000148826	0.00149592	0.0011921	2.90001E-07	0.9	5.69E-05	26.31025027
Sleeplessness	Falls	rs314280	6	G	A	0.00971363	0.0021846	0.00149134	0.00118827	7.29962E-11	0.0659994	9.18E-05	42.42382091
Sleeplessness	Falls	rs6938026	6	G	A	0.00984256	0.00434969	0.00182573	0.00145473	7.00003E-08	0.00280001	6.29E-05	29.06317374
Sleeplessness	Falls	rs10235198	7	T	C	0.01254	-0.00137249	0.00233378	0.00185956	7.69999E-08	0.46	6.24E-05	28.87189207

Sleeplessness	Falls	rs10276441	7	A	G	0.0096237	0.00236865	0.00181232	0.00144416	1.09999E-07	0.1	6.1E-05	28.19774548
Sleeplessness	Falls	rs1731951	7	A	T	-0.00787651	0.000329033	0.00150645	0.00120037	0.00000017	0.780001	5.91E-05	27.33746336
Sleeplessness	Falls	rs17151854	8	T	G	0.0129893	0.000610776	0.00207434	0.00165285	3.79997E-10	0.709999	8.48E-05	39.21133337
Sleeplessness	Falls	rs4831364	8	C	T	-0.0135102	-0.00110968	0.00260316	0.00207407	0.00000021	0.59	5.83E-05	26.93530092
Sleeplessness	Falls	rs11790060	9	C	T	-0.0103391	-0.00382724	0.00157854	0.00125785	5.79963E-11	0.00230001	9.28E-05	42.89970335
Sleeplessness	Falls	rs72704320	9	C	G	0.0103811	0.000962043	0.0019475	0.00155191	9.80009E-08	0.54	6.15E-05	28.41396338
Sleeplessness	Falls	rs17709610	10	G	A	-0.0099161	-0.000991664	0.00162078	0.00129136	9.49992E-10	0.44	8.1E-05	37.43119202
Sleeplessness	Falls	rs224032	10	A	G	0.00839066	0.000573643	0.00149108	0.00118811	1.79999E-08	0.630001	6.85E-05	31.66579213
Sleeplessness	Falls	rs2297787	10	A	T	-0.0178001	-0.00337767	0.00274996	0.00219109	9.60064E-11	0.12	9.06E-05	41.89788793
Sleeplessness	Falls	rs4748580	10	T	C	0.00796249	0.00346202	0.00154673	0.00123248	2.59998E-07	0.005	5.73E-05	26.50139783
Sleeplessness	Falls	rs10838708	11	A	G	-0.00947656	-0.00194502	0.00150271	0.00119735	2.90001E-10	0.1	8.6E-05	39.76958694
Sleeplessness	Falls	rs518143	11	G	A	-0.00789994	-0.00207476	0.00155741	0.00124092	3.89996E-07	0.0949992	5.56E-05	25.73011148
Sleeplessness	Falls	rs533757	11	C	T	0.00764807	0.00160639	0.00149524	0.00119136	3.09999E-07	0.18	5.66E-05	26.16265988
Sleeplessness	Falls	rs544566	11	C	T	-0.008001	-0.000335994	0.00149998	0.00119518	9.59997E-08	0.780001	6.15E-05	28.45231472
Sleeplessness	Falls	rs6486359	11	C	T	-0.00777985	-0.0035665	0.00150217	0.00119679	2.19999E-07	0.00290001	5.8E-05	26.82281032
Sleeplessness	Falls	rs72924721	11	T	C	0.016478	0.00237836	0.00288103	0.00229547	1.09999E-08	0.3	7.07E-05	32.71247742
Sleeplessness	Falls	rs324017	12	C	A	-0.00988248	-0.00329149	0.00163145	0.00129994	1.40001E-09	0.0109999	7.94E-05	36.69309554
Sleeplessness	Falls	rs4767643	12	C	G	-0.00762513	8.65159E-05	0.00150129	0.00119619	3.79997E-07	0.94	5.58E-05	25.79676937
Sleeplessness	Falls	rs68094047	12	T	C	0.0103356	0.00459805	0.0017167	0.00136795	1.7E-09	0.000779992	7.84E-05	36.24787673
Sleeplessness	Falls	rs931221	12	A	T	0.0106361	0.00393387	0.00175313	0.00139695	1.29999E-09	0.00490004	7.96E-05	36.80752203
Sleeplessness	Falls	rs1547630	13	A	G	0.00910814	0.00114101	0.00156447	0.0012465	5.80003E-09	0.36	7.33E-05	33.89416316
Sleeplessness	Falls	rs2451437	13	T	A	-0.00790356	-0.00269904	0.00152888	0.00121816	2.30001E-07	0.0269998	5.78E-05	26.72383061
Sleeplessness	Falls	rs6561715	13	A	T	-0.011623	-0.000758405	0.00154234	0.00122879	4.79954E-14	0.54	0.000123	56.79057052
Sleeplessness	Falls	rs9570080	13	C	T	-0.0106379	-0.00195841	0.00157858	0.00125778	1.59993E-11	0.12	9.82E-05	45.41283501
Sleeplessness	Falls	rs9576155	13	A	G	0.0081654	-0.000518673	0.00156892	0.0012501	1.89998E-07	0.68	5.86E-05	27.0865274
Sleeplessness	Falls	rs11628001	14	A	C	-0.00885303	4.51849E-05	0.00169038	0.00134674	0.00000016	0.97	5.93E-05	27.4293288

Sleeplessness	Falls	rs11635495	15	C	T	0.00937342	0.000340833	0.00148509	0.00118322	2.80001E-10	0.77	8.62E-05	39.83736519
Sleeplessness	Falls	rs176644	15	T	G	0.00805211	-0.000507182	0.0015188	0.00121005	1.09999E-07	0.68	6.08E-05	28.10724125
Sleeplessness	Falls	rs4886860	15	C	G	-0.011796	-0.000642989	0.00175566	0.00139863	1.80011E-11	0.649999	9.76E-05	45.1428214
Sleeplessness	Falls	rs872369	15	G	A	-0.00781943	0.000351843	0.00150709	0.00120065	0.00000021	0.77	5.82E-05	26.9197992
Sleeplessness	Falls	rs1125988	16	G	C	-0.00781557	0.0006882	0.00148983	0.00118709	0.00000016	0.56	5.95E-05	27.51996543
Sleeplessness	Falls	rs17139246	16	C	T	0.00786398	0.00113045	0.00153586	0.00122373	3.09999E-07	0.36	5.67E-05	26.21691229
Sleeplessness	Falls	rs2045458	16	T	A	0.00856982	0.000857197	0.00161985	0.00129068	0.00000012	0.51	6.05E-05	27.9894136
Sleeplessness	Falls	rs2062113	16	C	T	-0.00961678	0.000224244	0.00150275	0.00119731	1.6E-10	0.85	8.86E-05	40.95301577
Sleeplessness	Falls	rs35198836	16	T	C	0.00882425	0.00190304	0.00165213	0.00131637	9.20005E-08	0.15	6.17E-05	28.52772718
Sleeplessness	Falls	rs8074498	17	A	T	0.00810568	0.000418386	0.00151731	0.00120888	9.20005E-08	0.73	6.17E-05	28.53844257
Sleeplessness	Falls	rs9894577	17	A	G	0.0132051	0.000506292	0.00159687	0.00127231	1.29987E-16	0.69	0.000148	68.38238831
Sleeplessness	Falls	rs9906181	17	G	A	-0.00915026	0.00140168	0.00163869	0.00130554	2.39999E-08	0.28	6.74E-05	31.17979547
Sleeplessness	Falls	rs11152363	18	A	G	0.0156393	0.00377893	0.00192518	0.00153381	4.49987E-16	0.0140001	0.000143	65.99209845
Sleeplessness	Falls	rs2110119	19	A	G	-0.00816352	-0.00197586	0.0016033	0.00127717	3.50002E-07	0.12	5.61E-05	25.92539231
Sleeplessness	Falls	rs56330606	19	G	A	0.00930932	0.00322163	0.00153044	0.0012192	1.2E-09	0.00819993	8E-05	37.00013444
Sleeplessness	Falls	rs13040828	20	T	A	-0.00772419	-0.00130285	0.00152564	0.00121533	4.09996E-07	0.28	5.54E-05	25.63313723
Sleeplessness	Falls	rs6119267	20	G	C	0.00846101	0.0038395	0.00160721	0.0012804	1.40001E-07	0.00269998	5.99E-05	27.71399695
Sleeplessness	Falls	rs12484368	22	T	C	-0.0316884	-0.00773837	0.00607756	0.00484278	1.79999E-07	0.11	5.88E-05	27.18579971
Osteoporosis	Falls	rs2566755	1	C	T	-0.0020807	-0.00127821	0.00036342	0.00142837	1.03319E-08	0.37	9.72E-05	32.77942161
Osteoporosis	Falls	rs6684375	1	T	C	-0.00211508	-0.00409662	0.000395973	0.00155058	9.22529E-08	0.00819993	8.46E-05	28.53135841
Osteoporosis	Falls	rs10490823	3	T	C	-0.00191327	-0.00207816	0.000302262	0.0011871	2.45731E-10	0.08	0.000119	40.06687032
Osteoporosis	Falls	rs28402693	3	T	C	0.00226354	-0.000487275	0.000449257	0.00177386	4.69645E-07	0.780001	7.53E-05	25.38555376
Osteoporosis	Falls	rs7683315	4	A	T	0.00206293	0.000101011	0.000318431	0.00125104	9.28111E-11	0.94	0.000124	41.9699355
Osteoporosis	Falls	rs10280461	7	A	C	-0.00199254	-0.00159267	0.000302704	0.00118936	4.6334E-11	0.18	0.000128	43.32891135
Osteoporosis	Falls	rs3801387	7	G	A	-0.00249722	-0.00286447	0.000336089	0.00132219	1.08668E-13	0.0299999	0.000164	55.20838106
Osteoporosis	Falls	rs74777717	7	G	C	0.00301364	0.000771525	0.000578604	0.00229537	1.90507E-07	0.74	8.05E-05	27.12813137

Osteoporosis	Falls	rs11228240	11	T	C	0.00229604	-4.06063E-05	0.000336196	0.00132404	8.5369E-12	0.98	0.000138	46.64164032
Osteoporosis	Falls	rs4542364	11	A	G	0.00158514	0.000335359	0.000306966	0.00120554	2.42019E-07	0.780001	7.91E-05	26.66580509
Osteoporosis	Falls	rs7303922	12	G	A	0.00180259	-0.000628325	0.000331372	0.00130491	5.33777E-08	0.630001	8.78E-05	29.5911809
Osteoporosis	Falls	rs9594738	13	T	C	0.00205707	0.000462044	0.000300962	0.00118209	8.21486E-12	0.7	0.000139	46.71698563
BMI	Falls	rs11165643	1	C	T	-0.0221	0.0011462	0.003	0.00120242	1.434E-13	0.34	0.015191	32.88806094
BMI	Falls	rs17381664	1	C	T	0.0201	-0.000137404	0.0031	0.00120279	4.568E-11	0.91	0.013528	29.23953623
BMI	Falls	rs2820292	1	A	C	-0.0181	-0.00307633	0.0029	0.00119291	5.452E-10	0.00990011	0.01275	27.53603636
BMI	Falls	rs543874	1	G	A	0.0497	-0.00160565	0.0037	0.00146521	2.287E-40	0.27	0.027324	59.89536789
BMI	Falls	rs657452	1	A	G	0.0227	0.00224964	0.0031	0.00121677	2.123E-13	0.064	0.015083	32.65093402
BMI	Falls	rs7550711	1	T	C	0.0659	0.00337175	0.0087	0.00373152	5.059E-14	0.37	0.015472	33.50629033
BMI	Falls	rs977747	1	T	G	0.0168	-0.00075103	0.003	0.00120125	2.182E-08	0.53	0.0115	24.80474014
BMI	Falls	rs1016287	2	T	C	0.0228	0.00250527	0.0033	0.00129443	4.355E-12	0.0530005	0.014228	30.77411404
BMI	Falls	rs10182181	2	A	G	-0.0309	0.00028042	0.0029	0.00118379	8.071E-26	0.81	0.021585	47.0376612
BMI	Falls	rs10929925	2	C	A	0.0157	0.000013897	0.0029	0.00119648	9.434E-08	0.99	0.010966	23.64115103
BMI	Falls	rs12986742	2	C	T	0.0207	-0.00310595	0.0036	0.00118991	8.924E-09	0.00899995	0.011814	25.49089314
BMI	Falls	rs1460676	2	T	C	-0.0209	-0.00058129	0.0038	0.00163182	4.978E-08	0.719999	0.011202	24.154673
BMI	Falls	rs1528435	2	T	C	0.0175	0.0010941	0.003	0.00122066	4.774E-09	0.37	0.01203	25.9616526
BMI	Falls	rs1554622	2	T	G	-0.0158	-0.00124139	0.0029	0.0011829	5.938E-08	0.29	0.011137	24.01388797
BMI	Falls	rs17203016	2	G	A	0.0211	-2.26033E-05	0.0038	0.00148087	3.406E-08	0.99	0.011339	24.45464028
BMI	Falls	rs1979755	2	G	C	-0.0187	-0.000465384	0.0037	0.00119612	4.325E-07	0.7	0.010384	22.37217853
BMI	Falls	rs2890652	2	T	C	-0.0279	-0.00171949	0.0049	0.00160995	1.242E-08	0.29	0.011699	25.23994576
BMI	Falls	rs6713510	2	A	G	0.0164	0.000835336	0.0029	0.00118755	1.974E-08	0.48	0.011536	24.88338183
BMI	Falls	rs7599312	2	G	A	0.0214	-0.00248483	0.0033	0.00134824	4.73E-11	0.0649995	0.013518	29.21651144
BMI	Falls	rs972540	2	A	G	-0.0169	-0.000630156	0.0033	0.001323	3.951E-07	0.630001	0.01042	22.4504823
BMI	Falls	rs13078960	3	T	G	-0.029	0.000356794	0.0038	0.00147661	1.423E-14	0.81	0.015809	34.24846004
BMI	Falls	rs1516725	3	T	C	-0.0448	-0.00371655	0.0044	0.00172047	1.394E-24	0.0309999	0.021026	45.79411719

BMI	Falls	rs16851483	3	G	T	-0.0478	-1.53306E-05	0.0075	0.00238131	1.85E-10	0.99	0.013095	28.29014399
BMI	Falls	rs2365389	3	C	T	0.0195	0.00127835	0.003	0.00120655	1.346E-10	0.29	0.013193	28.50661094
BMI	Falls	rs3849570	3	A	C	0.0183	0.000205465	0.0033	0.00124253	1.933E-08	0.87	0.011543	24.8992808
BMI	Falls	rs6804842	3	A	G	-0.0183	-0.00174107	0.003	0.00119937	8.016E-10	0.15	0.012625	27.26176018
BMI	Falls	rs7613875	3	A	C	0.0156	0.00279744	0.0031	0.00118968	4.548E-07	0.0189998	0.010364	22.32982589
BMI	Falls	rs7649970	3	T	C	0.0231	0.00538388	0.0043	0.001814	1.008E-07	0.00299999	0.010941	23.58534006
BMI	Falls	rs876424	3	G	T	0.0188	0.00308419	0.0037	0.00123335	3.753E-07	0.012	0.01044	22.49466885
BMI	Falls	rs11727676	4	C	T	-0.0365	0.00307549	0.0063	0.00200212	6.247E-09	0.12	0.011937	25.75937987
BMI	Falls	rs13107325	4	C	T	-0.0472	-0.0066067	0.0066	0.00224919	1.064E-12	0.00329997	0.014634	31.66468685
BMI	Falls	rs13130484	4	C	T	-0.0398	-0.00117472	0.003	0.00119656	8.011E-41	0.33	0.027485	60.2578342
BMI	Falls	rs17001654	4	C	G	-0.0304	-0.00458344	0.0052	0.00168038	5.031E-09	0.0064	0.012012	25.92204039
BMI	Falls	rs11951673	5	T	C	-0.0154	-0.00126784	0.003	0.00120884	0.000000275	0.29	0.01056	22.7568097
BMI	Falls	rs2112347	5	G	T	-0.0254	-0.00135819	0.003	0.0012347	1.96E-17	0.27	0.017456	37.87922809
BMI	Falls	rs6864049	5	A	G	-0.0154	-0.000237698	0.003	0.00119924	2.123E-07	0.84	0.01066	22.97462686
BMI	Falls	rs7715256	5	G	T	0.0168	0.000531258	0.0029	0.00119455	8.851E-09	0.66	0.011817	25.49686761
BMI	Falls	rs13191362	6	A	G	0.0285	0.00396918	0.0047	0.00179809	1.092E-09	0.0269998	0.012524	27.04211104
BMI	Falls	rs13201877	6	A	G	-0.0236	0.000777992	0.0043	0.00175658	4.285E-08	0.66	0.011256	24.27320584
BMI	Falls	rs2228213	6	G	A	0.0156	6.22074E-05	0.0031	0.00124278	3.268E-07	0.96	0.010493	22.61077033
BMI	Falls	rs3800229	6	T	G	0.0175	0.000493044	0.0032	0.0013129	4.95E-08	0.709999	0.011204	24.15948546
BMI	Falls	rs539958	6	C	T	-0.0149	-0.000646119	0.0029	0.00118515	0.000000352	0.59	0.010465	22.548389
BMI	Falls	rs6457796	6	T	C	-0.0209	-0.0023778	0.0033	0.00133254	2.535E-10	0.0739997	0.012994	28.07082901
BMI	Falls	rs9374842	6	T	C	0.0196	0.000491594	0.0034	0.00140779	7.198E-09	0.73	0.011888	25.65288586
BMI	Falls	rs943005	6	T	C	0.0444	0.00176741	0.0038	0.00157548	4.524E-31	0.26	0.023815	52.01545823
BMI	Falls	rs10499694	7	G	A	-0.0147	0.00213457	0.0029	0.0011853	4.717E-07	0.0719996	0.01035	22.29843182
BMI	Falls	rs1167827	7	A	G	-0.02	-0.000247787	0.0031	0.00119412	1.975E-10	0.84	0.013073	28.24328827
BMI	Falls	rs2245368	7	T	C	-0.0288	-0.00170345	0.0053	0.00157605	7.008E-08	0.28	0.011076	23.88092217

BMI	Falls	rs9641123	7	G	C	-0.0193	-0.00112774	0.0037	0.00120521	1.826E-07	0.35	0.010717	23.09695011
BMI	Falls	rs2033732	8	C	T	0.0176	0.00123992	0.0034	0.00135846	2.256E-07	0.36	0.010636	22.92126504
BMI	Falls	rs2060604	8	T	C	0.0203	0.00201455	0.003	0.00120249	9.46E-12	0.0940005	0.014001	30.27611941
BMI	Falls	rs6990042	8	G	T	0.0155	0.00198559	0.0031	0.00118664	4.477E-07	0.0940005	0.01037	22.34308323
BMI	Falls	rs7844647	8	T	C	0.0171	0.00205512	0.0033	0.00134235	2.694E-07	0.13	0.010569	22.77535042
BMI	Falls	rs10733682	9	A	G	0.0188	-0.00124772	0.003	0.00120337	2.455E-10	0.3	0.013005	28.09304272
BMI	Falls	rs12352785	9	A	C	0.016	-0.0020664	0.0032	0.00127814	4.869E-07	0.11	0.010338	22.27162473
BMI	Falls	rs1928295	9	C	T	-0.0182	0.00125109	0.0029	0.00119304	4.318E-10	0.29	0.012825	27.69980499
BMI	Falls	rs2183825	9	C	T	0.0241	-0.00101705	0.0032	0.00126137	2.223E-14	0.42	0.015691	33.98917698
BMI	Falls	rs2270204	9	T	G	-0.018	-0.00177971	0.0035	0.00138997	0.000000322	0.2	0.010499	22.623798
BMI	Falls	rs4740619	9	T	C	0.017	0.00219673	0.0029	0.00118946	6.356E-09	0.0649995	0.011931	25.74627088
BMI	Falls	rs6477694	9	C	T	0.0169	-0.00129574	0.003	0.00124228	1.705E-08	0.3	0.011586	24.99289459
BMI	Falls	rs11191343	10	G	A	-0.0258	0.00313435	0.0051	0.00218663	4.862E-07	0.15	0.010338	22.27338691
BMI	Falls	rs17094222	10	C	T	0.0249	-0.0010788	0.0037	0.00144842	2.186E-11	0.46	0.013751	29.72780533
BMI	Falls	rs7899106	10	A	G	-0.0379	-0.00450774	0.0067	0.0027218	1.269E-08	0.0980009	0.011691	25.2216452
BMI	Falls	rs7903146	10	T	C	-0.0235	-0.00119135	0.0033	0.00130178	1.103E-12	0.36	0.014623	31.64165524
BMI	Falls	rs10840100	11	G	A	0.0206	0.0010745	0.003	0.00124487	6.666E-12	0.39	0.014104	30.50171046
BMI	Falls	rs12286929	11	G	A	0.0211	0.00264881	0.0029	0.00118637	5.443E-13	0.0259998	0.014821	32.07685339
BMI	Falls	rs2176598	11	T	C	0.0185	-0.00104364	0.0033	0.00137084	3.469E-08	0.450001	0.011333	24.44089852
BMI	Falls	rs3817334	11	C	T	-0.0256	0.00168327	0.003	0.00120086	1.168E-17	0.16	0.017578	38.15017714
BMI	Falls	rs11057405	12	A	G	-0.0304	-0.00235599	0.0053	0.00193304	1.22E-08	0.22	0.011705	25.25238688
BMI	Falls	rs11170468	12	C	A	-0.0186	-0.00166858	0.0035	0.00139966	1.013E-07	0.23	0.010941	23.58534006
BMI	Falls	rs11611246	12	T	G	0.0202	-0.000489547	0.0039	0.00144752	1.696E-07	0.74	0.010745	23.15800609
BMI	Falls	rs2579106	12	C	T	0.0212	-0.000360799	0.0041	0.00135479	2.332E-07	0.79	0.010624	22.89577187
BMI	Falls	rs7138803	12	G	A	-0.032	0.0010207	0.003	0.00122498	5.115E-26	0.4	0.021673	47.23365517
BMI	Falls	rs12429545	13	G	A	-0.0324	3.82993E-06	0.0044	0.00177762	3.152E-13	1	0.014973	32.41084356

BMI	Falls	rs1441264	13	A	G	0.0172	0.000175051	0.0031	0.00123026	2.959E-08	0.89	0.01139	24.56587922
BMI	Falls	rs9540493	13	G	A	-0.0182	-0.000951718	0.0031	0.00119387	3.952E-09	0.43	0.012094	26.10199489
BMI	Falls	rs9579083	13	G	C	-0.0295	0.00156503	0.0046	0.00154236	1.426E-10	0.31	0.013175	28.46717211
BMI	Falls	rs10132280	14	A	C	-0.0221	-0.000328161	0.0033	0.00129591	1.401E-11	0.8	0.013885	30.02121701
BMI	Falls	rs12887636	14	G	T	-0.0169	0.00160766	0.0032	0.00125135	1.346E-07	0.2	0.010832	23.3480778
BMI	Falls	rs3783890	14	C	T	-0.0198	0.000593273	0.0038	0.00152368	0.00000014	0.7	0.010818	23.31818522
BMI	Falls	rs709400	14	A	G	0.016	-9.61261E-05	0.003	0.00122392	1.382E-07	0.94	0.010824	23.33001627
BMI	Falls	rs7144011	14	T	G	0.0274	-0.00349416	0.0035	0.00142694	6.045E-15	0.0140001	0.016032	34.73855451
BMI	Falls	rs13329567	15	T	C	-0.0307	0.00301653	0.0035	0.00141777	1.526E-18	0.0329997	0.018054	39.20165076
BMI	Falls	rs3736485	15	A	G	0.016	-1.85302E-05	0.0029	0.00119154	4.524E-08	0.99	0.011237	24.23175934
BMI	Falls	rs4984406	15	T	C	0.0156	6.00622E-05	0.0029	0.00118412	7.568E-08	0.96	0.011048	23.8189941
BMI	Falls	rs12448257	16	G	A	-0.0246	0.000834261	0.0037	0.00144793	3.898E-11	0.56	0.013576	29.3453974
BMI	Falls	rs2307022	16	G	A	-0.0159	0.00249274	0.0031	0.00125521	4.322E-07	0.0470002	0.010385	22.37415267
BMI	Falls	rs3888190	16	A	C	0.0311	-0.000734478	0.003	0.00120709	3.454E-25	0.54	0.021302	46.40676591
BMI	Falls	rs879620	16	C	T	-0.0244	-0.000811503	0.0039	0.00121845	3.939E-10	0.51	0.012854	27.76445467
BMI	Falls	rs9926784	16	T	C	0.0249	-0.00129904	0.0038	0.00152388	8.548E-11	0.39	0.013336	28.81776102
BMI	Falls	rs1000940	17	G	A	0.0184	0.000517539	0.0033	0.00129156	1.812E-08	0.69	0.011566	24.94897391
BMI	Falls	rs12150665	17	T	C	0.016	0.000199668	0.003	0.00120258	8.058E-08	0.87	0.011025	23.76834697
BMI	Falls	rs12940622	17	A	G	-0.0183	-0.000746144	0.0029	0.00119049	3.636E-10	0.53	0.01288	27.81995219
BMI	Falls	rs4986044	17	C	T	0.0155	0.00074664	0.003	0.00118968	1.787E-07	0.53	0.010725	23.1152773
BMI	Falls	rs6504108	17	C	T	0.0172	0.00179864	0.0032	0.00131627	1.117E-07	0.17	0.010902	23.50105568
BMI	Falls	rs7223966	17	G	A	0.0171	0.000592078	0.0033	0.0013144	1.378E-07	0.649999	0.010824	23.33001627
BMI	Falls	rs17066856	18	C	T	-0.0371	-0.00248599	0.005	0.00205426	2.003E-13	0.23	0.015099	32.68612582
BMI	Falls	rs6567160	18	C	T	0.0562	0.00225129	0.0035	0.00139951	6.684E-59	0.11	0.033241	73.31242373
BMI	Falls	rs7239883	18	G	A	0.0152	0.000487098	0.003	0.00122285	3.142E-07	0.69	0.010509	22.64505447
BMI	Falls	rs891389	18	C	T	-0.0209	0.00150823	0.0037	0.00124494	1.617E-08	0.23	0.011605	25.03461234

BMI	Falls	rs11672550	19	T	C	0.0211	0.000450669	0.004	0.00120941	1.328E-07	0.709999	0.010837	23.36033597
BMI	Falls	rs11672660	19	C	T	0.0339	-9.95071E-05	0.0038	0.00149257	7.911E-19	0.95	0.018206	39.53709516
BMI	Falls	rs11880870	19	G	A	-0.0176	-9.29693E-05	0.0034	0.00118332	0.000000239	0.94	0.010614	22.87450327
BMI	Falls	rs14810	19	C	G	-0.0183	-0.00128644	0.0033	0.00126717	1.923E-08	0.31	0.011545	24.90330556
BMI	Falls	rs17724992	19	A	G	0.0196	0.00256002	0.0034	0.00133897	7.787E-09	0.0560003	0.011861	25.59343522
BMI	Falls	rs405509	19	T	G	-0.0166	0.000823027	0.0032	0.00118286	2.654E-07	0.49	0.010575	22.78793431
BMI	Falls	rs9304665	19	A	T	0.0243	0.00198744	0.0043	0.00139477	1.594E-08	0.15	0.011612	25.04902028
BMI	Falls	rs6091540	20	C	T	0.0185	0.00382413	0.0033	0.00129482	2.138E-08	0.00309999	0.011506	24.81913512
BMI	Falls	rs8123881	20	G	A	0.0216	0.00217962	0.0043	0.0017565	3.908E-07	0.21	0.010424	22.45914403
BMI	Falls	rs2836754	21	C	T	0.0169	-0.00130202	0.003	0.00122913	1.605E-08	0.29	0.011607	25.03938596
BMI	Falls	rs427943	21	C	A	0.0182	0.000197875	0.0035	0.00119501	1.593E-07	0.87	0.01077	23.2133071
Type 2 diabetes	Falls	rs1127655	1	T	C	-0.0438	-0.000400375	0.0079	0.00118578	2.47E-08	0.74	0.000489	30.7393046
Type 2 diabetes	Falls	rs12088739	1	G	A	-0.0884	0.00438465	0.013	0.00205852	9.79E-12	0.0329997	0.000735	46.24
Type 2 diabetes	Falls	rs12568159	1	T	A	0.0782	-0.00161987	0.0153	0.00237877	3.08E-07	0.5	0.000415	26.12345679
Type 2 diabetes	Falls	rs2296173	1	G	A	0.065	0.00107953	0.0087	0.00144911	7.66E-14	0.46	0.000887	55.81979125
Type 2 diabetes	Falls	rs2493394	1	G	A	0.073	0.00276749	0.0113	0.00190172	1.15E-10	0.15	0.000663	41.73388676
Type 2 diabetes	Falls	rs2820426	1	G	A	0.0521	-0.00403843	0.0073	0.00121434	1.30E-12	0.000879995	0.000809	50.93657347
Type 2 diabetes	Falls	rs340874	1	C	T	0.0626	-0.00231975	0.0073	0.00119204	8.41E-18	0.0519996	0.001168	73.5364984
Type 2 diabetes	Falls	rs348330	1	A	G	-0.0487	0.000596819	0.0081	0.00123125	1.86E-09	0.630001	0.000574	36.14830056
Type 2 diabetes	Falls	rs522367	1	C	T	0.0415	-0.000269169	0.0078	0.00118718	1.05E-07	0.82	0.00045	28.30785667
Type 2 diabetes	Falls	rs58786391	1	G	A	-0.0449	-9.46E-05	0.0085	0.00127265	1.25E-07	0.94	0.000443	27.9032526
Type 2 diabetes	Falls	rs12617659	2	T	C	-0.0685	0.00155925	0.0103	0.00166838	2.83E-11	0.35	0.000703	44.22895655
Type 2 diabetes	Falls	rs13389219	2	T	C	-0.0722	0.0010595	0.0074	0.0012107	2.11E-22	0.38	0.001511	95.19430241
Type 2 diabetes	Falls	rs17334919	2	T	C	-0.1398	-0.0037598	0.0128	0.00197136	6.69E-28	0.0560003	0.001893	119.2873535
Type 2 diabetes	Falls	rs243019	2	C	T	0.0566	-0.00130489	0.0071	0.00119123	2.29E-15	0.27	0.001009	63.55008927
Type 2 diabetes	Falls	rs28545614	2	T	C	0.0563	0.00441504	0.0106	0.00163397	1.22E-07	0.00690001	0.000448	28.21012816

Type 2 diabetes	Falls	rs2972144	2	G	A	0.0913	-0.00209098	0.0075	0.00123725	2.55E-34	0.0909997	0.002351	148.1900444
Type 2 diabetes	Falls	rs6545714	2	A	G	-0.0383	-0.00428784	0.0074	0.0012104	1.89E-07	4.00E-04	0.000426	26.7876187
Type 2 diabetes	Falls	rs7572970	2	G	A	0.059	0.0013384	0.0087	0.00131748	1.39E-11	0.31	0.000731	45.99022328
Type 2 diabetes	Falls	rs780094	2	C	T	0.0692	-0.00174736	0.0074	0.00121427	5.16E-21	0.15	0.001389	87.4477721
Type 2 diabetes	Falls	rs840967	2	A	C	-0.0497	0.00240436	0.008	0.00121304	5.44E-10	0.0470002	0.000613	38.59515625
Type 2 diabetes	Falls	rs11242	3	C	T	0.039	0.000920705	0.0072	0.00119049	6.24E-08	0.44	0.000466	29.34027778
Type 2 diabetes	Falls	rs11708067	3	G	A	-0.0965	0.00157593	0.0086	0.00137951	5.93E-29	0.25	0.001998	125.9092753
Type 2 diabetes	Falls	rs11925227	3	A	G	-0.0534	0.00322871	0.0095	0.00152932	2.25E-08	0.0350002	0.000502	31.59623269
Type 2 diabetes	Falls	rs11926707	3	C	T	0.0463	0.00210009	0.0082	0.00122571	1.69E-08	0.0870001	0.000507	31.88117192
Type 2 diabetes	Falls	rs1496653	3	G	A	-0.0769	-0.000326015	0.0088	0.00146792	2.57E-18	0.82	0.001213	76.3637655
Type 2 diabetes	Falls	rs1899951	3	T	C	-0.1118	0.00554726	0.0109	0.0018055	1.64E-24	0.0021	0.00167	105.2036024
Type 2 diabetes	Falls	rs2292662	3	T	C	-0.0629	-0.00012222	0.0111	0.00165611	1.24E-08	0.94	0.00051	32.11111111
Type 2 diabetes	Falls	rs4686471	3	C	T	0.0534	-0.000817337	0.0081	0.00121812	4.28E-11	0.5	0.000691	43.46227709
Type 2 diabetes	Falls	rs6795735	3	T	C	-0.0558	0.0011635	0.0073	0.00120118	1.63E-14	0.33	0.000928	58.42822293
Type 2 diabetes	Falls	rs7619041	3	A	T	-0.0431	-0.000519644	0.0078	0.00118189	2.76E-08	0.66	0.000485	30.53270874
Type 2 diabetes	Falls	rs7651090	3	G	A	0.1204	-0.0020446	0.0076	0.001274	3.85E-57	0.11	0.003975	250.9722992
Type 2 diabetes	Falls	rs9822589	3	A	G	-0.0421	-0.00116931	0.0079	0.00119048	9.52E-08	0.33	0.000451	28.39945522
Type 2 diabetes	Falls	rs9844972	3	C	G	0.0956	-2.82E-06	0.0148	0.00234051	1.03E-10	1	0.000663	41.72461651
Type 2 diabetes	Falls	rs9845672	3	A	G	-0.0396	-0.00378624	0.0074	0.00124271	8.91E-08	0.00230001	0.000455	28.63696129
Type 2 diabetes	Falls	rs11098676	4	C	T	0.054	0.000765125	0.0096	0.00145013	2.03E-08	0.6	0.000503	31.640625
Type 2 diabetes	Falls	rs12505596	4	G	A	-0.0404	0.000266932	0.0078	0.00118531	1.94E-07	0.82	0.000426	26.82708744
Type 2 diabetes	Falls	rs1296328	4	C	A	-0.0412	0.00136134	0.0079	0.00119712	1.98E-07	0.26	0.000432	27.19820542
Type 2 diabetes	Falls	rs17086692	4	T	G	-0.0467	-0.00150871	0.0084	0.00127923	2.48E-08	0.24	0.000491	30.90830499
Type 2 diabetes	Falls	rs1801214	4	T	C	0.0903	0.000805898	0.0074	0.00120888	5.52E-34	0.5	0.002362	148.9059533
Type 2 diabetes	Falls	rs4697140	4	A	C	-0.0604	-0.00458326	0.0111	0.00171119	5.75E-08	0.00739997	0.000471	29.60928496
Type 2 diabetes	Falls	rs735949	4	C	T	-0.0711	0.00167254	0.0106	0.00170438	1.95E-11	0.33	0.000715	44.99118904

Type 2 diabetes	Falls	rs7674212	4	T	G	-0.0465	-0.000136562	0.0075	0.00120883	6.18E-10	0.91	0.000611	38.44
Type 2 diabetes	Falls	rs7685296	4	T	C	-0.0511	0.000383799	0.0081	0.00131964	2.32E-10	0.77	0.000632	39.79896357
Type 2 diabetes	Falls	rs993380	4	G	A	-0.0507	0.00283484	0.0081	0.00125411	4.59E-10	0.0239999	0.000623	39.17832647
Type 2 diabetes	Falls	rs10077431	5	A	C	-0.0487	-0.000354054	0.0089	0.0014399	4.75E-08	0.81	0.000476	29.94180028
Type 2 diabetes	Falls	rs1061813	5	A	G	-0.0429	-0.0017819	0.0073	0.00118732	3.37E-09	0.13	0.000549	34.5357478
Type 2 diabetes	Falls	rs1291041	5	T	G	-0.0411	-0.000624277	0.0081	0.00123788	4.46E-07	0.61	0.000409	25.74622771
Type 2 diabetes	Falls	rs329122	5	A	G	0.0372	-0.00313767	0.0072	0.00120008	2.35E-07	0.0089	0.000424	26.69444444
Type 2 diabetes	Falls	rs459193	5	G	A	0.0711	-0.0012021	0.0083	0.00135726	8.81E-18	0.38	0.001165	73.38089708
Type 2 diabetes	Falls	rs4865796	5	A	G	0.053	-0.000557364	0.0078	0.00128217	1.33E-11	0.66	0.000734	46.17028271
Type 2 diabetes	Falls	rs597350	5	C	T	0.0409	0.00188276	0.0081	0.00119519	3.92E-07	0.12	0.000405	25.49626581
Type 2 diabetes	Falls	rs6878122	5	A	G	-0.0564	6.99E-05	0.0079	0.00126916	1.19E-12	0.96	0.00081	50.96875501
Type 2 diabetes	Falls	rs7729395	5	T	C	0.1373	0.00405233	0.016	0.00265625	1.10E-17	0.13	0.00117	73.63785156
Type 2 diabetes	Falls	rs1050226	6	G	A	-0.0491	-0.00170859	0.0074	0.00120308	3.34E-11	0.16	0.0007	44.02501826
Type 2 diabetes	Falls	rs1063355	6	G	T	0.0709	0.00174687	0.0079	0.00121381	3.72E-19	0.15	0.001279	80.54494472
Type 2 diabetes	Falls	rs197482	6	C	T	0.0424	0.000629906	0.0081	0.00122366	1.74E-07	0.61	0.000436	27.40070111
Type 2 diabetes	Falls	rs2246618	6	T	C	0.0513	-0.0017909	0.0084	0.00128088	1.20E-09	0.16	0.000593	37.29719388
Type 2 diabetes	Falls	rs3756784	6	G	T	0.0505	0.00407041	0.0091	0.00151456	2.59E-08	0.00719996	0.000489	30.7964014
Type 2 diabetes	Falls	rs622217	6	C	T	-0.0485	0.000780799	0.0077	0.00118679	3.13E-10	0.51	0.00063	39.67363805
Type 2 diabetes	Falls	rs72892910	6	T	G	0.0648	0.00197831	0.0099	0.00156957	6.43E-11	0.21	0.000681	42.84297521
Type 2 diabetes	Falls	rs7756992	6	G	A	0.1297	0.00173661	0.0078	0.00133703	6.00E-62	0.19	0.004377	276.4972058
Type 2 diabetes	Falls	rs853974	6	C	T	-0.0601	4.05E-05	0.0088	0.00134773	7.86E-12	0.98	0.000741	46.64269112
Type 2 diabetes	Falls	rs9369425	6	A	G	-0.0546	0.00111797	0.0085	0.00129954	1.13E-10	0.39	0.000656	41.2617301
Type 2 diabetes	Falls	rs13234269	7	A	T	-0.0583	0.00252818	0.0078	0.00118712	6.98E-14	0.0329997	0.000888	55.86604208
Type 2 diabetes	Falls	rs13239186	7	T	C	0.0539	0.00321672	0.0085	0.00129606	2.70E-10	0.0129999	0.000639	40.21051903
Type 2 diabetes	Falls	rs17132130	7	C	G	-0.0485	-0.00462941	0.0095	0.0014274	3.09E-07	0.0012	0.000414	26.06371191
Type 2 diabetes	Falls	rs17168486	7	T	C	0.0742	0.00208921	0.0094	0.00156966	2.18E-15	0.18	0.00099	62.30918968

Type 2 diabetes	Falls	rs2191348	7	T	G	0.0652	0.000515014	0.0073	0.00118933	3.44E-19	0.66	0.001267	79.7718146
Type 2 diabetes	Falls	rs2267716	7	C	T	-0.0477	-0.000812635	0.0093	0.00139836	2.57E-07	0.56	0.000418	26.3069719
Type 2 diabetes	Falls	rs2299383	7	T	C	0.0412	0.00216922	0.0073	0.00120254	1.49E-08	0.0710003	0.000506	31.85288047
Type 2 diabetes	Falls	rs2908282	7	A	G	0.0552	0.00090137	0.0094	0.00154654	4.25E-09	0.56	0.000548	34.48438207
Type 2 diabetes	Falls	rs7786095	7	G	A	-0.0743	-0.000181677	0.0129	0.00192858	9.64E-09	0.92	0.000527	33.174028
Type 2 diabetes	Falls	rs849135	7	A	G	-0.0999	-0.000612444	0.0072	0.00118267	1.04E-43	0.6	0.003052	192.515625
Type 2 diabetes	Falls	rs10087241	8	A	G	-0.0475	-0.00153439	0.008	0.00120682	2.80E-09	0.2	0.00056	35.25390625
Type 2 diabetes	Falls	rs10100265	8	C	A	-0.0491	-0.000708328	0.0079	0.00121673	6.29E-10	0.56	0.000614	38.62858516
Type 2 diabetes	Falls	rs1561927	8	T	C	-0.0412	0.000499897	0.0081	0.00134246	3.95E-07	0.709999	0.000411	25.8716659
Type 2 diabetes	Falls	rs17411031	8	G	C	-0.045	0.000315465	0.0081	0.00134435	3.04E-08	0.81	0.000491	30.86419753
Type 2 diabetes	Falls	rs2294120	8	G	A	-0.0443	0.000861676	0.0079	0.00118858	1.62E-08	0.47	0.0005	31.44512097
Type 2 diabetes	Falls	rs28592805	8	T	A	-0.0432	-0.00044343	0.0082	0.00123622	1.17E-07	0.719999	0.000441	27.75490779
Type 2 diabetes	Falls	rs3802177	8	A	G	-0.1217	-0.000389123	0.008	0.00128126	2.32E-52	0.760001	0.003666	231.4201563
Type 2 diabetes	Falls	rs516946	8	C	T	0.0824	-0.000744716	0.0085	0.0013879	3.16E-22	0.59	0.001492	93.97591696
Type 2 diabetes	Falls	rs7845219	8	C	T	-0.0422	-0.00215232	0.0072	0.00118425	4.54E-09	0.0690001	0.000546	34.35262346
Type 2 diabetes	Falls	rs10114341	9	C	T	-0.0409	-0.00122224	0.0072	0.00119311	1.15E-08	0.31	0.000513	32.26871142
Type 2 diabetes	Falls	rs10811661	9	C	T	-0.1569	3.81E-05	0.0098	0.00156334	4.13E-58	0.98	0.004059	256.3266347
Type 2 diabetes	Falls	rs10974438	9	C	A	0.0591	-0.00143678	0.0075	0.00123998	3.01E-15	0.25	0.000986	62.0944
Type 2 diabetes	Falls	rs1333039	9	C	G	0.0534	2.62E-05	0.0074	0.00120717	5.64E-13	0.98	0.000827	52.07377648
Type 2 diabetes	Falls	rs1758632	9	G	C	0.0491	0.00137451	0.0081	0.00121924	1.36E-09	0.26	0.000584	36.74455114
Type 2 diabetes	Falls	rs17791483	9	G	A	-0.102	0.000328521	0.0147	0.00244791	3.42E-12	0.89	0.000765	48.14660558
Type 2 diabetes	Falls	rs2796441	9	A	G	-0.0715	0.000261008	0.0073	0.00119697	1.96E-22	0.83	0.001523	95.93263276
Type 2 diabetes	Falls	rs7856768	9	A	G	0.041	-0.00187956	0.008	0.00121078	2.85E-07	0.12	0.000417	26.265625
Type 2 diabetes	Falls	rs10740322	10	A	G	0.0477	0.0010781	0.0085	0.00128073	2.11E-08	0.4	0.0005	31.49190311
Type 2 diabetes	Falls	rs11000760	10	A	G	0.041	-0.00112377	0.008	0.00120134	2.72E-07	0.35	0.000417	26.265625
Type 2 diabetes	Falls	rs11257655	10	T	C	0.0737	0.000176616	0.0087	0.00145625	1.97E-17	0.9	0.00114	71.76231999

Type 2 diabetes	Falls	rs3817285	10	C	T	-0.0399	-0.000488529	0.0079	0.00119055	4.57E-07	0.68	0.000405	25.50889281
Type 2 diabetes	Falls	rs753270	10	C	T	0.0528	0.00105689	0.0079	0.00120614	2.70E-11	0.38	0.00071	44.66976446
Type 2 diabetes	Falls	rs7903146	10	T	C	0.3059	-0.00119135	0.0077	0.00130178	1.00E-200	0.36	0.024481	1578.256198
Type 2 diabetes	Falls	rs7923866	10	T	C	-0.0972	-0.000291429	0.0074	0.00121848	9.34E-40	0.81	0.002736	172.531775
Type 2 diabetes	Falls	rs10830963	11	G	C	0.0909	-0.000927787	0.008	0.00132324	5.85E-30	0.48	0.002049	129.1064063
Type 2 diabetes	Falls	rs11229389	11	A	G	-0.0388	-0.00239016	0.0075	0.00121891	1.88E-07	0.05	0.000425	26.76337778
Type 2 diabetes	Falls	rs12802926	11	A	C	0.0666	0.00119469	0.0123	0.00188206	5.66E-08	0.53	0.000466	29.31826294
Type 2 diabetes	Falls	rs1552224	11	C	A	-0.1034	-0.00161471	0.0101	0.00163621	8.64E-25	0.32	0.001664	104.8089403
Type 2 diabetes	Falls	rs2237892	11	T	C	-0.096	0.000545986	0.0157	0.00241378	8.75E-10	0.82	0.000594	37.38894073
Type 2 diabetes	Falls	rs5215	11	T	C	-0.0678	0.00245124	0.0073	0.00123426	2.09E-20	0.0470002	0.00137	86.26083693
Type 2 diabetes	Falls	rs67232546	11	T	C	0.0596	-0.000995536	0.0096	0.001456	4.66E-10	0.49	0.000612	38.54340278
Type 2 diabetes	Falls	rs67924081	11	G	A	0.0451	-0.00120158	0.0089	0.00133828	3.38E-07	0.37	0.000408	25.67870218
Type 2 diabetes	Falls	rs7929543	11	C	A	0.0828	0.00452141	0.0138	0.0021406	2.20E-09	0.0350002	0.000572	36
Type 2 diabetes	Falls	rs10842994	12	T	C	-0.0755	-0.00238546	0.0091	0.00148663	1.02E-16	0.11	0.001093	68.83528559
Type 2 diabetes	Falls	rs10875976	12	A	G	0.0369	-0.00142966	0.0072	0.00118564	3.34E-07	0.23	0.000417	26.265625
Type 2 diabetes	Falls	rs11107116	12	T	G	0.0467	-0.000997406	0.0085	0.00142602	3.75E-08	0.48	0.00048	30.18532872
Type 2 diabetes	Falls	rs12299509	12	G	A	0.0467	0.00113457	0.0073	0.00118439	2.09E-10	0.34	0.00065	40.92493901
Type 2 diabetes	Falls	rs1579238	12	G	A	-0.0483	-0.000553078	0.0091	0.00138211	1.24E-07	0.69	0.000448	28.17159763
Type 2 diabetes	Falls	rs2261181	12	T	C	0.0985	0.00297745	0.0118	0.00199874	9.18E-17	0.14	0.001107	69.68004884
Type 2 diabetes	Falls	rs61953351	12	T	G	-0.07	-0.000759545	0.0091	0.00136598	1.98E-14	0.58	0.00094	59.17159763
Type 2 diabetes	Falls	rs7138300	12	T	C	-0.0443	0.00150677	0.0072	0.00119239	5.65E-10	0.21	0.000602	37.85667438
Type 2 diabetes	Falls	rs825476	12	T	C	0.0524	-0.00152555	0.0073	0.00119801	6.80E-13	0.2	0.000819	51.52486395
Type 2 diabetes	Falls	rs1359790	13	A	G	-0.0796	-0.00134345	0.008	0.00130758	2.80E-23	0.3	0.001572	99.0025
Type 2 diabetes	Falls	rs576674	13	A	G	-0.0654	-0.0010115	0.0097	0.00158049	1.79E-11	0.52	0.000722	45.45817834
Type 2 diabetes	Falls	rs7489413	13	C	T	-0.0585	-0.000935713	0.0115	0.00176645	3.81E-07	0.6	0.000411	25.87712665
Type 2 diabetes	Falls	rs963740	13	T	A	-0.0479	-0.00056009	0.0086	0.00129618	2.23E-08	0.67	0.000493	31.02230936

Type 2 diabetes	Falls	rs17522122	14	T	G	0.0403	0.00287557	0.0074	0.00118907	5.21E-08	0.016	0.000471	29.65832725
Type 2 diabetes	Falls	rs2183237	14	A	G	-0.0487	0.00255394	0.0092	0.00137975	1.22E-07	0.064	0.000445	28.0209121
Type 2 diabetes	Falls	rs7144011	14	T	G	0.0482	-0.00349416	0.0085	0.00142694	1.64E-08	0.0140001	0.000511	32.15557093
Type 2 diabetes	Falls	rs1031664	15	A	C	-0.0364	0.000321487	0.0072	0.00118606	3.89E-07	0.79	0.000406	25.55864198
Type 2 diabetes	Falls	rs12910825	15	G	A	0.0517	0.00306028	0.0074	0.00123189	2.16E-12	0.0129999	0.000776	48.81099343
Type 2 diabetes	Falls	rs2289739	15	T	G	0.0438	0.00241962	0.0082	0.00125165	1.05E-07	0.0530005	0.000453	28.53123141
Type 2 diabetes	Falls	rs2440352	15	G	T	0.0419	0.000211056	0.0082	0.00134871	2.75E-07	0.88	0.000415	26.10960738
Type 2 diabetes	Falls	rs6494307	15	G	C	-0.0443	0.000327056	0.0078	0.00119559	1.67E-08	0.780001	0.000513	32.25657462
Type 2 diabetes	Falls	rs7177055	15	A	G	0.0647	-0.000792246	0.0079	0.00131241	2.75E-16	0.55	0.001065	67.0740266
Type 2 diabetes	Falls	rs982077	15	G	A	-0.0453	-0.00066072	0.0072	0.00119476	2.58E-10	0.58	0.000629	39.58506944
Type 2 diabetes	Falls	rs11646985	16	G	C	0.038	0.000143914	0.0074	0.00118945	3.23E-07	0.9	0.000419	26.36961286
Type 2 diabetes	Falls	rs13330951	16	G	A	-0.0456	-0.00113126	0.0081	0.00118606	1.54E-08	0.34	0.000504	31.69272977
Type 2 diabetes	Falls	rs2925979	16	C	T	-0.0534	0.00326538	0.0078	0.00129124	9.06E-12	0.0109999	0.000745	46.86982249
Type 2 diabetes	Falls	rs7185735	16	G	A	0.1056	0.00417648	0.0073	0.00121009	1.59E-47	0.000560003	0.003316	209.2580221
Type 2 diabetes	Falls	rs77258096	16	A	C	-0.1171	-0.000121528	0.0134	0.00196217	1.78E-18	0.95	0.001213	76.36672978
Type 2 diabetes	Falls	rs9940149	16	A	G	-0.058	0.000857374	0.0095	0.00154029	9.29E-10	0.58	0.000592	37.27423823
Type 2 diabetes	Falls	rs1035061	17	G	A	0.0586	0.00183997	0.0115	0.0019201	3.83E-07	0.34	0.000413	25.96567108
Type 2 diabetes	Falls	rs12945601	17	C	T	-0.048	0.00432774	0.008	0.00122151	1.72E-09	4.00E-04	0.000572	36
Type 2 diabetes	Falls	rs17405722	17	A	G	0.087	0.00228777	0.0146	0.00225377	2.28E-09	0.31	0.000564	35.50853819
Type 2 diabetes	Falls	rs17631783	17	T	C	-0.0487	-0.000435922	0.0089	0.00134925	3.95E-08	0.75	0.000476	29.94180028
Type 2 diabetes	Falls	rs8068804	17	A	G	0.0587	-0.00128824	0.0078	0.00126216	4.41E-14	0.31	0.0009	56.63527285
Type 2 diabetes	Falls	rs9894220	17	G	A	-0.0585	-0.00163777	0.0079	0.00119219	1.52E-13	0.17	0.000871	54.83496235
Type 2 diabetes	Falls	rs1062557	18	A	C	0.046	-0.000580509	0.009	0.0013508	3.24E-07	0.67	0.000415	26.12345679
Type 2 diabetes	Falls	rs12970134	18	A	G	0.0555	0.00144855	0.008	0.00133848	5.31E-12	0.28	0.000765	48.12890625
Type 2 diabetes	Falls	rs7240767	18	C	T	0.0451	-0.00125008	0.0081	0.00121599	2.16E-08	0.3	0.000493	31.00152416
Type 2 diabetes	Falls	rs10401969	19	C	T	0.0921	0.00181863	0.0133	0.00223432	4.13E-12	0.42	0.000762	47.95302165

Type 2 diabetes	Falls	rs8108269	19	G	T	0.0644	0.00238124	0.0079	0.00131443	3.11E-16	0.0700003	0.001056	66.45345297
Type 2 diabetes	Falls	rs4810426	20	T	C	0.0726	-0.000204208	0.013	0.00200507	2.15E-08	0.92	0.000496	31.18792899
Type 2 diabetes	Falls	rs4812034	20	T	G	0.0401	0.00135203	0.0078	0.00118774	2.59E-07	0.25	0.00042	26.43014464
Type 2 diabetes	Falls	rs6059662	20	G	A	0.0446	0.000485508	0.0079	0.00125363	1.51E-08	0.7	0.000507	31.87245634
Type 2 diabetes	Falls	rs6066138	20	A	G	-0.049	6.18E-05	0.0082	0.00131661	1.93E-09	0.96	0.000567	35.70791196
Type 2 diabetes	Falls	rs6515236	20	C	A	-0.0504	-0.00106616	0.0091	0.00136871	3.34E-08	0.44	0.000488	30.67455621
Type 2 diabetes	Falls	rs16988333	22	G	A	-0.0745	-0.00318014	0.013	0.00204557	9.17E-09	0.12	0.000522	32.84171598
Type 2 diabetes	Falls	rs4823182	22	G	A	0.0482	0.000666876	0.0077	0.00125543	3.36E-10	0.6	0.000623	39.18434812
Cataract	Falls	rs3738725	1	C	T	0.00160377	0.000307457	0.000285721	0.00118435	2.00E-08	0.8	6.80E-05	31.50647729
Cataract	Falls	rs9842371	3	T	C	0.00201022	0.00113311	0.00029732	0.00123254	1.40E-11	0.36	9.87E-05	45.71291655
Cataract	Falls	rs116049102	4	G	C	-0.00373197	0.000460145	0.000680778	0.00282163	4.20E-08	0.87	6.49E-05	30.05143829
Cataract	Falls	rs6535231	4	A	G	-0.00414025	0.000783268	0.000715431	0.00296565	7.20E-09	0.79	7.23E-05	33.49019088
Cataract	Falls	rs17172647	7	G	A	0.00235069	-0.000360176	0.000331001	0.00137223	1.20E-12	0.79	0.000109	50.43500961
Cataract	Falls	rs1679013	9	T	C	0.00175087	-0.00242415	0.000300302	0.00124499	5.50E-09	0.0519996	7.34E-05	33.99314554
Cataract	Falls	rs150159363	11	C	T	0.00376928	-0.00364584	0.000487203	0.00202005	1.00E-14	0.0710003	0.000129	59.85451175
Cataract	Falls	rs8049373	16	T	G	0.00147075	0.00251479	0.000289884	0.00120181	3.90E-07	0.0359998	5.56E-05	25.74122575
Cataract	Falls	rs9911460	17	T	A	0.00159111	0.00060839	0.000286376	0.00118737	2.80E-08	0.61	6.67E-05	30.86932784
Cataract	Falls	rs6046142	20	A	G	0.00378953	-6.98E-05	0.000391167	0.00162192	3.40E-22	0.97	0.000203	93.85259396
Alzheimer's disease	Falls	rs6733839	2	T	C	0.00313752	-0.000402344	0.000499313	0.00123281	3.30E-10	0.74	9.88E-05	39.48455602
Alzheimer's disease	Falls	rs77043094	4	T	C	0.00707863	-0.00285004	0.00139886	0.00345852	4.20E-07	0.41	6.40E-05	25.60648229
Alzheimer's disease	Falls	rs4803748	19	T	C	-0.00286042	-0.000245027	0.00049505	0.00122326	7.60E-09	0.84	8.35E-05	33.38577654
Alzheimer's disease	Falls	rs56394238	19	G	A	0.00365176	-0.00200955	0.00048412	0.00119585	4.60E-14	0.0929994	0.000142	56.89818397
Alzheimer's disease	Falls	rs6076601	20	A	G	0.0026353	-0.00100392	0.000517113	0.00127666	3.50E-07	0.43	6.50E-05	25.97103229
Depression	Falls	rs10942927	5	T	A	0.00260137	-0.000296215	0.000501953	0.00122904	2.20E-07	0.81	5.80E-05	26.85827688
Depression	Falls	rs9398376	6	T	C	-0.00315815	-0.00169562	0.000610472	0.00149477	2.30E-07	0.26	5.78E-05	26.76295158
Depression	Falls	rs13229801	7	G	A	-0.00261934	0.000380872	0.000502328	0.00123003	1.80E-07	0.760001	5.87E-05	27.18998556

Depression	Falls	rs3807866	7	A	G	0.00291681	-0.000210774	0.000490398	0.00120085	2.70E-09	0.86	7.64E-05	35.3768288
Depression	Falls	rs73173375	13	T	A	0.00525169	0.00356341	0.00102519	0.0025103	3.00E-07	0.16	5.67E-05	26.24154748
Atherosclerotic heart disease	Falls	rs2133189	1	T	C	0.00207304	0.000313029	0.000365916	0.0013098	1.50E-08	0.81	6.93E-05	32.09612217
Atherosclerotic heart disease	Falls	rs602633	1	G	T	0.00358677	-0.000495111	0.000400177	0.00143235	3.20E-19	0.73	0.000173	80.33463208
Atherosclerotic heart disease	Falls	rs61772578	1	G	A	0.00348194	-0.00277849	0.000527183	0.00188689	4.00E-11	0.14	9.42E-05	43.62342618
Atherosclerotic heart disease	Falls	rs16986953	2	A	G	0.00433502	0.00194664	0.000659033	0.00235922	4.80E-11	0.41	9.34E-05	43.26810625
Atherosclerotic heart disease	Falls	rs2028900	2	T	C	0.00201822	-0.000643554	0.00033246	0.00118998	1.30E-09	0.59	7.96E-05	36.85175796
Atherosclerotic heart disease	Falls	rs2351524	2	C	T	-0.00346638	0.0054564	0.000494192	0.0017686	2.30E-12	0.002	0.000106	49.19952604
Atherosclerotic heart disease	Falls	rs145626624	3	C	T	0.00517572	0.00236075	0.00102162	0.00365636	4.10E-07	0.52	5.54E-05	25.66627092
Atherosclerotic heart disease	Falls	rs10305838	4	C	T	0.00322435	0.00040191	0.000475195	0.00170087	1.20E-11	0.81	9.94E-05	46.04056358
Atherosclerotic heart disease	Falls	rs4646272	6	G	T	0.00508401	0.00170476	0.00069501	0.00248799	2.60E-13	0.49	0.000116	53.50947444
Atherosclerotic heart disease	Falls	rs55730499	6	T	C	0.00968562	-2.50E-05	0.000610859	0.00218638	1.30E-56	0.99	0.000543	251.4044141
Atherosclerotic heart disease	Falls	rs7766436	6	T	C	0.00189281	0.00183456	0.000360574	0.00129079	1.50E-07	0.16	5.95E-05	27.55657435
Atherosclerotic heart disease	Falls	rs9285476	6	G	C	-0.00236428	0.00073395	0.00036301	0.00129944	7.40E-11	0.57	9.16E-05	42.4190208
Atherosclerotic heart disease	Falls	rs9349379	6	G	A	0.00302667	-0.000766014	0.000336469	0.00120445	2.40E-19	0.52	0.000175	80.91704778
Atherosclerotic heart disease	Falls	rs117507170	7	A	G	0.0052704	0.00327365	0.000974239	0.00348687	6.30E-08	0.35	6.32E-05	29.26551245
Atherosclerotic heart disease	Falls	rs57301765	7	A	G	0.0030837	0.00130275	0.000452774	0.00162052	9.70E-12	0.42	0.0001	46.38539737
Atherosclerotic heart disease	Falls	rs28601761	8	G	C	-0.00208224	0.00107747	0.000338777	0.00121257	7.90E-10	0.37	8.16E-05	37.77754525
Atherosclerotic heart disease	Falls	rs1537373	9	G	T	0.00590598	-3.61E-05	0.000330253	0.00118219	1.60E-71	0.98	0.00069	319.8087935
Atherosclerotic heart disease	Falls	rs1623851	10	G	A	-0.00244291	0.000375321	0.000436659	0.00156305	2.20E-08	0.81	6.76E-05	31.29897411
Atherosclerotic heart disease	Falls	rs9337951	10	A	G	0.00202501	-0.00235018	0.000356069	0.00127449	1.30E-08	0.0649995	6.99E-05	32.34342553
Atherosclerotic heart disease	Falls	rs2128739	11	C	A	-0.00214379	-0.000136657	0.000367429	0.00131515	5.40E-09	0.92	7.35E-05	34.04220008
Atherosclerotic heart disease	Falls	rs4938809	11	C	T	0.00194347	-0.00281037	0.000356285	0.00127528	4.90E-08	0.0280001	6.43E-05	29.75504458
Atherosclerotic heart disease	Falls	rs597808	12	G	A	-0.00206726	0.000275376	0.000331332	0.00118601	4.40E-10	0.82	8.41E-05	38.92812076
Atherosclerotic heart disease	Falls	rs9515203	13	C	T	-0.00219037	-0.0010974	0.000375436	0.00134372	5.40E-09	0.41	7.35E-05	34.0379297
Atherosclerotic heart disease	Falls	rs3783320	14	G	A	0.00198854	-0.00149373	0.000393885	0.00140988	4.50E-07	0.29	5.50E-05	25.48764903

Atherosclerotic heart disease	Falls	rs2071384	15	A	G	-0.00241993	0.000954639	0.000348517	0.00124749	3.80E-12	0.44	0.000104	48.21228033
Atherosclerotic heart disease	Falls	rs2904223	15	A	G	-0.00247783	-0.000575006	0.000337845	0.00120929	2.20E-13	0.630001	0.000116	53.79080229
Atherosclerotic heart disease	Falls	rs62053262	16	G	C	-0.00424186	-0.00254084	0.000748502	0.00267871	1.50E-08	0.34	6.94E-05	32.11639061
Atherosclerotic heart disease	Falls	rs7500448	16	G	A	-0.00203114	0.000689465	0.0003831	0.00137125	1.10E-07	0.62	6.07E-05	28.10964844
Atherosclerotic heart disease	Falls	rs11657636	17	T	C	0.00248748	0.000417905	0.000359998	0.00128867	4.90E-12	0.75	0.000103	47.74402393
Atherosclerotic heart disease	Falls	rs55714120	17	T	G	0.00220267	-0.000433454	0.000351484	0.00125803	3.70E-10	0.73	8.48E-05	39.2724281
Atherosclerotic heart disease	Falls	rs145436496	19	A	G	0.00512481	0.00265895	0.000983928	0.00352122	1.90E-07	0.450001	5.86E-05	27.12869471
Atherosclerotic heart disease	Falls	rs2738447	19	C	A	0.001716	-0.00272073	0.000336717	0.00120525	3.50E-07	0.0239999	5.61E-05	25.97194534
Atherosclerotic heart disease	Falls	rs73015013	19	T	C	-0.00349003	-0.00104687	0.000511267	0.0018301	8.70E-12	0.57	0.000101	46.59751926
Atherosclerotic heart disease	Falls	rs28451064	21	A	G	0.00326133	0.00538472	0.000500094	0.00179007	7.00E-11	0.00259998	9.18E-05	42.52910103
Glaucoma	Falls	rs2814471	1	T	C	-0.00413886	0.00265423	0.000318959	0.00180312	1.70E-38	0.14	0.000364	168.3804828
Glaucoma	Falls	rs4450544	2	T	C	0.00119862	-0.00124832	0.000222018	0.00125502	6.70E-08	0.32	6.30E-05	29.14651725
Glaucoma	Falls	rs6706384	2	T	G	0.00126982	0.00187407	0.000216503	0.00122371	4.50E-09	0.13	7.43E-05	34.39984525
Glaucoma	Falls	rs7626665	3	T	C	0.00107767	-0.000645079	0.000213976	0.00120959	4.70E-07	0.59	5.48E-05	25.36538491
Glaucoma	Falls	rs9309969	3	G	T	-0.00111923	-0.00052798	0.000213376	0.00120617	1.60E-07	0.66	5.94E-05	27.51360805
Glaucoma	Falls	rs55937650	4	T	C	-0.0018759	-0.00329181	0.000213447	0.00120663	1.50E-18	0.0064	0.000167	77.23946491
Glaucoma	Falls	rs76325372	5	C	A	-0.00128709	6.80E-05	0.000236173	0.00133501	5.00E-08	0.96	6.42E-05	29.70006088
Glaucoma	Falls	rs17135234	6	C	A	0.0016986	-0.00257756	0.000305	0.00172433	2.60E-08	0.13	6.70E-05	31.01576952
Glaucoma	Falls	rs3013274	6	A	G	-0.00107787	9.83E-05	0.000213772	0.00120859	4.60E-07	0.94	5.49E-05	25.42325352
Glaucoma	Falls	rs4896201	6	A	G	-0.00119354	0.000582322	0.000215687	0.00121931	3.10E-08	0.630001	6.61E-05	30.62146871
Glaucoma	Falls	rs2526101	7	G	A	0.00111173	0.00262589	0.000213208	0.00120527	1.80E-07	0.0290001	5.87E-05	27.18890129
Glaucoma	Falls	rs4385407	7	A	G	0.00130633	0.000280907	0.000234665	0.00132656	2.60E-08	0.83	6.69E-05	30.98911412
Glaucoma	Falls	rs7788304	7	C	T	-0.00113905	-0.00105841	0.000217503	0.0012295	1.60E-07	0.39	5.92E-05	27.42553282
Glaucoma	Falls	rs1333037	9	T	C	0.00191106	0.000118226	0.000211197	0.00119401	1.40E-19	0.92	0.000177	81.87911789
Glaucoma	Falls	rs2472493	9	A	G	-0.00189042	-0.000332696	0.000212564	0.00120159	5.90E-19	0.780001	0.000171	79.09284157
Glaucoma	Falls	rs55738911	9	A	G	0.00180118	-0.00159124	0.000243349	0.00137566	1.30E-13	0.25	0.000118	54.78417174

Glaucoma	Falls	rs2084080	11	A	G	0.00147967	-0.00141242	0.000284254	0.00160689	1.90E-07	0.38	5.85E-05	27.09671028
Glaucoma	Falls	rs58073046	11	G	A	0.00166205	-0.00276353	0.000326455	0.00184566	3.60E-07	0.13	5.60E-05	25.9203893
Glaucoma	Falls	rs11159095	14	C	G	0.00110094	-0.00126586	0.000210177	0.00118819	1.60E-07	0.29	5.93E-05	27.43828259
Glaucoma	Falls	rs34619158	14	T	C	0.00112736	-0.00134192	0.000216922	0.00122628	2.00E-07	0.27	5.83E-05	27.0095932
Glaucoma	Falls	rs35320790	14	A	C	-0.00165618	-0.000342828	0.000213946	0.00120961	9.90E-15	0.780001	0.000129	59.92481876
Glaucoma	Falls	rs9913911	17	G	A	-0.00173988	-0.000713675	0.000216787	0.00122568	1.00E-15	0.56	0.000139	64.41279151
Glaucoma	Falls	rs971748	20	T	C	0.00110821	-0.000363863	0.000214762	0.00121427	2.50E-07	0.760001	5.75E-05	26.6274299
Glaucoma	Falls	rs2078555	22	G	C	-0.0011814	0.00124521	0.000223059	0.00126112	1.20E-07	0.32	6.06E-05	28.05139291
Stroke	Falls	rs11587860	1	C	G	-0.0689	-0.000394536	0.0098	0.00124487	2.54E-12	0.75	0.000111	49.42950854
Stroke	Falls	rs1537407	1	C	T	0.0662	0.00064463	0.0131	0.00148775	4.48E-07	0.66	5.72E-05	25.53720646
Stroke	Falls	rs17035646	1	A	G	0.0522	0.00173944	0.0096	0.0012589	6.12E-08	0.17	6.62E-05	29.56640625
Stroke	Falls	rs72699046	1	C	G	0.0897	-0.00297266	0.0171	0.0023151	1.53E-07	0.2	6.16E-05	27.51646661
Stroke	Falls	rs2066864	4	A	G	0.0562	-0.000162	0.0106	0.00138592	1.29E-07	0.91	6.29E-05	28.11000356
Stroke	Falls	rs2634074	4	A	T	-0.084	0.00182243	0.0112	0.00149199	6.56E-14	0.22	0.000126	56.25
Stroke	Falls	rs2585193	5	A	G	0.0504	5.98E-05	0.0098	0.00128152	2.45E-07	0.96	5.92E-05	26.44897959
Stroke	Falls	rs11242678	6	T	C	0.0643	0.000841006	0.0105	0.00136674	8.71E-10	0.54	8.39E-05	37.50104308
Stroke	Falls	rs35276016	6	T	C	0.0984	0.00336816	0.0184	0.00226364	9.31E-08	0.14	6.40E-05	28.59924386
Stroke	Falls	rs1549758	7	C	T	-0.0532	-0.0018076	0.0104	0.00127133	3.11E-07	0.16	5.86E-05	26.16715976
Stroke	Falls	rs2107595	7	A	G	0.0803	0.00121832	0.0121	0.00164247	3.59E-11	0.46	9.86E-05	44.04132231
Stroke	Falls	rs42039	7	T	C	-0.0574	-0.000619466	0.011	0.00137825	1.65E-07	0.649999	6.10E-05	27.22942149
Stroke	Falls	rs1537375	9	C	T	0.0519	2.81E-05	0.0091	0.00118215	1.24E-08	0.98	7.28E-05	32.52759329
Stroke	Falls	rs10883926	10	A	G	0.0503	0.00138512	0.0094	0.00120621	8.15E-08	0.25	6.41E-05	28.63388411
Stroke	Falls	rs2284665	10	T	G	-0.0602	-0.002666	0.0111	0.00144272	5.99E-08	0.0649995	6.58E-05	29.41352163
Stroke	Falls	rs36053597	11	T	C	0.061	-0.00311882	0.0121	0.00160034	4.55E-07	0.051	5.69E-05	25.41493067
Stroke	Falls	rs475937	11	C	A	-0.0757	-0.00379312	0.0137	0.00179435	2.92E-08	0.0350002	6.83E-05	30.53167457
Stroke	Falls	rs10774624	12	A	G	-0.0654	-2.58E-05	0.0094	0.00119446	4.04E-12	0.98	0.000108	48.40606609

Stroke	Falls	rs7294375	12	G	T	0.053	0.0001117	0.0104	0.00133784	3.33E-07	0.93	5.81E-05	25.97078402
Stroke	Falls	rs12445022	16	A	G	0.052	-0.000268988	0.0098	0.00126602	1.03E-07	0.83	6.30E-05	28.15493544
Stroke	Falls	rs7219031	17	A	G	0.0596	0.00290442	0.0116	0.00150451	2.59E-07	0.0539995	5.91E-05	26.39833532
Stroke	Falls	rs8103309	19	C	T	-0.0522	0.000713207	0.0103	0.00122916	3.70E-07	0.56	5.75E-05	25.68423037
Bipolar disorder	Falls	rs139498219	2	C	T	0.00297921	0.00118324	0.000584167	0.00554199	3.40E-07	0.83	7.71E-05	26.00927189
Bipolar disorder	Falls	rs112418576	7	G	A	0.00439135	-0.00814455	0.000755317	0.00714069	6.11E-09	0.25	0.0001	33.80162555
Bipolar disorder	Falls	rs140575369	9	G	T	0.00253166	-0.00686637	0.000489568	0.00456509	2.33E-07	0.13	7.93E-05	26.74143531
Bipolar disorder	Falls	rs146989801	19	G	C	0.00273816	-0.00391998	0.000520476	0.00488541	1.43E-07	0.42	8.21E-05	27.67682241
Parkinson's disease	Falls	rs35749011	1	A	G	0.7508	-0.000537573	0.0659	0.00504919	5.02E-30	0.92	0.000269	129.8008985
Parkinson's disease	Falls	rs823106	1	C	G	-0.1492	-0.00280148	0.0239	0.00180354	4.10E-10	0.12	8.07E-05	38.97102642
Parkinson's disease	Falls	rs4613239	2	G	C	0.1784	-0.000545591	0.0248	0.00183153	6.21E-13	0.77	0.000107	51.7471384
Parkinson's disease	Falls	rs6741007	2	G	T	-0.1233	8.97E-05	0.0175	0.00121417	2.09E-12	0.94	0.000103	49.6420898
Parkinson's disease	Falls	rs10513789	3	G	T	-0.1596	0.00131786	0.0219	0.00150257	3.18E-13	0.38	0.00011	53.110152
Parkinson's disease	Falls	rs4488803	3	A	G	-0.1136	0.00036833	0.0199	0.00122478	1.08E-08	0.760001	6.75E-05	32.58745991
Parkinson's disease	Falls	rs34311866	4	C	T	0.2272	-0.00216078	0.0231	0.00155498	7.97E-23	0.16	0.0002	96.73701767
Parkinson's disease	Falls	rs356203	4	T	C	-0.2398	0.00128481	0.0178	0.00122721	3.01E-41	0.3	0.000376	181.4923621
Parkinson's disease	Falls	rs4698412	4	A	G	0.1258	0.0021432	0.0168	0.00118858	7.05E-14	0.0710003	0.000116	56.07157029
Parkinson's disease	Falls	rs7695720	4	C	A	-0.1255	0.000345489	0.0208	0.00142545	1.53E-09	0.81	7.54E-05	36.40497874
Parkinson's disease	Falls	rs75646569	5	G	T	0.1916	-0.00196853	0.0266	0.00203579	5.62E-13	0.33	0.000107	51.88331732
Parkinson's disease	Falls	rs35265698	6	G	C	-0.2	0.00400418	0.0303	0.00150868	3.93E-11	0.008	9.06E-05	43.56871331
Parkinson's disease	Falls	rs858295	7	G	A	-0.1039	5.87E-05	0.0176	0.0012133	3.83E-09	0.96	7.22E-05	34.85023889
Parkinson's disease	Falls	rs620490	8	G	T	-0.1174	-0.00154874	0.019	0.00130171	6.46E-10	0.23	7.91E-05	38.17939058
Parkinson's disease	Falls	rs10756905	9	T	C	0.1011	-0.00100343	0.0196	0.0014113	2.46E-07	0.48	5.51E-05	26.60664827
Parkinson's disease	Falls	rs2208485	9	A	G	-0.0936	0.000379066	0.0182	0.00119203	2.65E-07	0.75	5.48E-05	26.44897959
Parkinson's disease	Falls	rs144814361	10	T	C	0.4411	0.0103881	0.068	0.00471233	9.07E-11	0.0269998	8.72E-05	42.07811635
Parkinson's disease	Falls	rs329647	11	C	G	-0.1133	-0.00239725	0.0178	0.00127866	1.94E-10	0.061	8.39E-05	40.51537053

Parkinson's disease	Falls	rs10847864	12	T	G	0.1274	-6.31E-05	0.0179	0.0012379	9.81E-13	0.96	0.000105	50.65622172
Parkinson's disease	Falls	rs28370649	12	G	A	0.2836	-0.0019323	0.0547	0.00489204	2.20E-07	0.69	5.57E-05	26.88052833
Parkinson's disease	Falls	rs75505347	12	T	C	0.3917	0.00694398	0.0674	0.00481889	6.12E-09	0.15	7.00E-05	33.77437725
Parkinson's disease	Falls	rs4774417	15	A	G	0.1052	0.00229269	0.0192	0.00134624	4.63E-08	0.089	6.22E-05	30.02126736
Parkinson's disease	Falls	rs12934900	16	T	A	0.1215	-0.00340262	0.0184	0.00123048	4.33E-11	0.00569994	9.03E-05	43.60305411
Parkinson's disease	Falls	rs10451230	17	T	A	-0.096	0.0015581	0.0175	0.00119176	4.42E-08	0.19	6.23E-05	30.09306122
Parkinson's disease	Falls	rs58879558	17	C	T	-0.2383	0.00435984	0.025	0.00140426	1.36E-21	0.00189998	0.000188	90.859024
Parkinson's disease	Falls	rs847685	17	C	G	-0.1483	-0.000153471	0.0293	0.0014691	3.98E-07	0.92	5.47E-05	25.61810854
Parkinson's disease	Falls	rs4588066	18	A	G	0.1046	0.00194213	0.0178	0.00127792	4.45E-09	0.13	7.15E-05	34.53212978
Falls	Epilepsy	rs2494196	1	A	C	0.00130899	0.000162695	0.00130899	0.000162695	2.00E-07	0.3	2.31E-06	27.04812046
Falls	Epilepsy	rs7636391	3	A	G	0.00119681	0.000148746	0.00119681	0.000148746	4.30E-07	0.7	3.13E-07	25.57706747
Falls	Epilepsy	rs171697	5	G	C	0.00125879	0.000156436	0.00125879	0.000156436	1.00E-08	0.85	8.07E-08	32.84349151
Falls	Epilepsy	rs686	5	A	G	0.001214	0.000150877	0.001214	0.000150877	2.10E-07	0.25	2.82E-06	26.94952556
Falls	Epilepsy	rs2709062	7	A	G	0.00118653	0.000147464	0.00118653	0.000147464	1.20E-08	0.15	4.44E-06	32.44974598
Falls	Epilepsy	rs17695376	8	C	G	0.00146714	0.000182336	0.00146714	0.000182336	5.20E-08	0.55	7.80E-07	29.64002917
Falls	Epilepsy	rs494221	11	G	A	0.00125012	0.000155355	0.00125012	0.000155355	5.50E-08	0.19	3.63E-06	29.5146906
Falls	Epilepsy	rs71480157	11	C	T	0.00154595	0.000192126	0.00154595	0.000192126	8.60E-09	0.52	8.75E-07	33.13832612
Falls	Epilepsy	rs12884871	14	T	C	0.00129401	0.000160808	0.00129401	0.000160808	8.20E-08	0.46	1.18E-06	28.76495512
Falls	Epilepsy	rs11645704	16	T	G	0.00129264	0.000160654	0.00129264	0.000160654	3.90E-07	0.13	5.05E-06	25.73538614
Falls	Epilepsy	rs12956276	18	A	G	0.00122674	0.000152442	0.00122674	0.000152442	8.80E-08	0.12	5.17E-06	28.62181094
Falls	Epilepsy	rs28633123	19	T	C	0.0014539	0.000180659	0.0014539	0.000180659	1.00E-08	0.11	5.44E-06	32.81747625
Falls	Epilepsy	rs8105753	19	C	A	0.00123595	0.000153567	0.00123595	0.000153567	9.50E-08	0.37	1.76E-06	28.48153822
Falls	Epilepsy	rs2281147	20	A	T	0.00157873	0.000196177	0.00157873	0.000196177	8.10E-08	0.91	2.97E-08	28.78556002
Falls	Fracture	rs2494196	1	A	C	0.00680777	-0.000401153	0.00130899	0.000681601	2.00E-07	0.56	5.86E-05	27.04812046
Falls	Fracture	rs7559374	2	A	C	0.0117244	0.00226596	0.00223021	0.00116102	1.50E-07	0.051	5.99E-05	27.63692602
Falls	Fracture	rs7616516	3	A	G	0.0116807	0.00131294	0.00228799	0.00119143	3.30E-07	0.27	5.64E-05	26.06330558

Falls	Fracture	rs7636391	3	A	G	-0.00605272	-0.00134975	0.00119681	0.000623137	4.30E-07	0.0299999	5.54E-05	25.57706747
Falls	Fracture	rs171697	5	G	C	0.00721403	0.00174792	0.00125879	0.000655398	1.00E-08	0.00769999	7.11E-05	32.84349151
Falls	Fracture	rs686	5	A	G	0.00630223	0.00118899	0.001214	0.000631966	2.10E-07	0.0599998	5.84E-05	26.94952556
Falls	Fracture	rs17695376	8	C	G	-0.0079875	-0.0012826	0.00146714	0.000763916	5.20E-08	0.0929994	6.42E-05	29.64002917
Falls	Fracture	rs10858334	9	G	C	0.00901925	0.00144896	0.00170213	0.000886133	1.20E-07	0.1	6.08E-05	28.07730358
Falls	Fracture	rs494221	11	G	A	-0.00679158	-0.000212569	0.00125012	0.000650858	5.50E-08	0.74	6.39E-05	29.5146906
Falls	Fracture	rs71480157	11	C	T	-0.0088994	-0.000454468	0.00154595	0.000804764	8.60E-09	0.57	7.18E-05	33.13832612
Falls	Fracture	rs12884871	14	T	C	-0.00694016	-0.00130305	0.00129401	0.00067362	8.20E-08	0.0530005	6.23E-05	28.76495512
Falls	Fracture	rs12956276	18	A	G	0.00656298	0.000867192	0.00122674	0.0006386	8.80E-08	0.17	6.20E-05	28.62181094
Falls	Fracture	rs28633123	19	T	C	0.00832889	0.00165314	0.0014539	0.000756908	1.00E-08	0.0290001	7.11E-05	32.81747625
Falls	Fracture	rs8105753	19	C	A	0.00659603	0.00147664	0.00123595	0.000643364	9.50E-08	0.0219999	6.17E-05	28.48153822
Falls	Fracture	rs2281147	20	A	T	-0.00847023	-0.000136493	0.00157873	0.0008218	8.10E-08	0.87	6.23E-05	28.78556002
Falls	Stroke	rs2494196	1	A	C	0.00680777	0.0145	0.00130899	0.01	2.00E-07	0.1482	5.86E-05	27.04812046
Falls	Stroke	rs7559374	2	A	C	0.0117244	0.0188	0.00223021	0.0172	1.50E-07	0.2746	5.99E-05	27.63692602
Falls	Stroke	rs7616516	3	A	G	0.0116807	0.0276	0.00228799	0.0183	3.30E-07	0.1318	5.64E-05	26.06330558
Falls	Stroke	rs7636391	3	A	G	-0.00605272	0.0018	0.00119681	0.0094	4.30E-07	0.8506	5.54E-05	25.57706747
Falls	Stroke	rs171697	5	G	C	0.00721403	0.0206	0.00125879	0.01	1.00E-08	0.0386999	7.11E-05	32.84349151
Falls	Stroke	rs686	5	A	G	0.00630223	-0.0073	0.001214	0.0097	2.10E-07	0.4525	5.84E-05	26.94952556
Falls	Stroke	rs2709062	7	A	G	0.00675903	-0.0018	0.00118653	0.0092	1.20E-08	0.8429	7.03E-05	32.44974598
Falls	Stroke	rs17695376	8	C	G	-0.0079875	-0.0248	0.00146714	0.0114	5.20E-08	0.0291099	6.42E-05	29.64002917
Falls	Stroke	rs10858334	9	G	C	0.00901925	-0.0066	0.00170213	0.0141	1.20E-07	0.6389	6.08E-05	28.07730358
Falls	Stroke	rs494221	11	G	A	-0.00679158	-0.0081	0.00125012	0.0098	5.50E-08	0.41	6.39E-05	29.5146906
Falls	Stroke	rs71480157	11	C	T	-0.0088994	-0.0151	0.00154595	0.0122	8.60E-09	0.2154	7.18E-05	33.13832612
Falls	Stroke	rs12884871	14	T	C	-0.00694016	-6.00E-04	0.00129401	0.01	8.20E-08	0.9496	6.23E-05	28.76495512
Falls	Stroke	rs11645704	16	T	G	0.00655757	0.0046	0.00129264	0.0099	3.90E-07	0.6428	5.57E-05	25.73538614
Falls	Stroke	rs12956276	18	A	G	0.00656298	0.0064	0.00122674	0.0096	8.80E-08	0.5054	6.20E-05	28.62181094

Falls	Stroke	rs28633123	19	T	C	0.00832889	0.0031	0.0014539	0.0118	1.00E-08	0.791199	7.11E-05	32.81747625
Falls	Stroke	rs8105753	19	C	A	0.00659603	0.0069	0.00123595	0.0097	9.50E-08	0.4806	6.17E-05	28.48153822
Falls	Stroke	rs2281147	20	A	T	-0.00847023	-0.0163	0.00157873	0.0124	8.10E-08	0.189	6.23E-05	28.78556002
Falls	Headache	rs2494196	1	A	C	0.00680777	4.78E-05	0.00130899	0.000220543	2.00E-07	0.83	5.86E-05	27.04812046
Falls	Headache	rs7636391	3	A	G	-0.00605272	1.14E-05	0.00119681	0.000201638	4.30E-07	0.95	5.54E-05	25.57706747
Falls	Headache	rs171697	5	G	C	0.00721403	0.000140818	0.00125879	0.00021206	1.00E-08	0.51	7.11E-05	32.84349151
Falls	Headache	rs686	5	A	G	0.00630223	-9.99E-05	0.001214	0.000204526	2.10E-07	0.630001	5.84E-05	26.94952556
Falls	Headache	rs2709062	7	A	G	0.00675903	0.000288202	0.00118653	0.000199898	1.20E-08	0.15	7.03E-05	32.44974598
Falls	Headache	rs17695376	8	C	G	-0.0079875	4.05E-06	0.00146714	0.000247169	5.20E-08	0.99	6.42E-05	29.64002917
Falls	Headache	rs10858334	9	G	C	0.00901925	-0.000101448	0.00170213	0.000286715	1.20E-07	0.719999	6.08E-05	28.07730358
Falls	Headache	rs494221	11	G	A	-0.00679158	0.000318494	0.00125012	0.000210595	5.50E-08	0.13	6.39E-05	29.5146906
Falls	Headache	rs71480157	11	C	T	-0.0088994	0.000193339	0.00154595	0.00026044	8.60E-09	0.46	7.18E-05	33.13832612
Falls	Headache	rs12884871	14	T	C	-0.00694016	-0.000242068	0.00129401	0.000217986	8.20E-08	0.27	6.23E-05	28.76495512
Falls	Headache	rs11645704	16	T	G	0.00655757	-0.000130169	0.00129264	0.000217777	3.90E-07	0.55	5.57E-05	25.73538614
Falls	Headache	rs12956276	18	A	G	0.00656298	0.000499293	0.00122674	0.000206646	8.80E-08	0.016	6.20E-05	28.62181094
Falls	Headache	rs28633123	19	T	C	0.00832889	-0.000111465	0.0014539	0.000244897	1.00E-08	0.649999	7.11E-05	32.81747625
Falls	Headache	rs8105753	19	C	A	0.00659603	0.000273733	0.00123595	0.000208172	9.50E-08	0.19	6.17E-05	28.48153822
Falls	Headache	rs2281147	20	A	T	-0.00847023	-3.46E-05	0.00157873	0.000265932	8.10E-08	0.9	6.23E-05	28.78556002
Falls	Patient death	rs2494196	1	A	C	0.00680777	-7.93E-05	0.00130899	0.000119878	2.00E-07	0.51	5.86E-05	27.04812046
Falls	Patient death	rs7636391	3	A	G	-0.00605272	6.94E-05	0.00119681	0.000109595	4.30E-07	0.53	5.54E-05	25.57706747
Falls	Patient death	rs171697	5	G	C	0.00721403	8.15E-05	0.00125879	0.000115266	1.00E-08	0.48	7.11E-05	32.84349151
Falls	Patient death	rs686	5	A	G	0.00630223	4.84E-05	0.001214	0.000111166	2.10E-07	0.66	5.84E-05	26.94952556
Falls	Patient death	rs2709062	7	A	G	0.00675903	6.07E-05	0.00118653	0.000108653	1.20E-08	0.58	7.03E-05	32.44974598
Falls	Patient death	rs494221	11	G	A	-0.00679158	-4.57E-06	0.00125012	0.000114463	5.50E-08	0.97	6.39E-05	29.5146906
Falls	Patient death	rs12884871	14	T	C	-0.00694016	1.29E-05	0.00129401	0.000118482	8.20E-08	0.91	6.23E-05	28.76495512
Falls	Patient death	rs11645704	16	T	G	0.00655757	-7.70E-05	0.00129264	0.000118379	3.90E-07	0.52	5.57E-05	25.73538614

Falls	Patient death	rs12956276	18	A	G	0.00656298	0.000267548	0.00122674	0.000112321	8.80E-08	0.017	6.20E-05	28.62181094
Falls	Patient death	rs8105753	19	C	A	0.00659603	9.05E-05	0.00123595	0.000113145	9.50E-08	0.42	6.17E-05	28.48153822
Falls	Anxiety disorder	rs2494196	1	A	C	0.00680777	-7.96E-05	0.00130899	0.00013176	2.00E-07	0.55	5.86E-05	27.04812046
Falls	Anxiety disorder	rs7636391	3	A	G	-0.00605272	0.000109765	0.00119681	0.000120463	4.30E-07	0.36	5.54E-05	25.57706747
Falls	Anxiety disorder	rs171697	5	G	C	0.00721403	0.000139823	0.00125879	0.00012669	1.00E-08	0.27	7.11E-05	32.84349151
Falls	Anxiety disorder	rs686	5	A	G	0.00630223	0.000165886	0.001214	0.000122188	2.10E-07	0.17	5.84E-05	26.94952556
Falls	Anxiety disorder	rs2709062	7	A	G	0.00675903	0.000146271	0.00118653	0.000119424	1.20E-08	0.22	7.03E-05	32.44974598
Falls	Anxiety disorder	rs494221	11	G	A	-0.00679158	0.000248507	0.00125012	0.000125814	5.50E-08	0.0479999	6.39E-05	29.5146906
Falls	Anxiety disorder	rs12884871	14	T	C	-0.00694016	0.000194092	0.00129401	0.00013023	8.20E-08	0.14	6.23E-05	28.76495512
Falls	Anxiety disorder	rs11645704	16	T	G	0.00655757	-1.70E-05	0.00129264	0.000130105	3.90E-07	0.9	5.57E-05	25.73538614
Falls	Anxiety disorder	rs12956276	18	A	G	0.00656298	7.93E-05	0.00122674	0.000123455	8.80E-08	0.52	6.20E-05	28.62181094
Falls	Anxiety disorder	rs8105753	19	C	A	0.00659603	-1.25E-05	0.00123595	0.000124366	9.50E-08	0.92	6.17E-05	28.48153822
Falls	Severe stress	rs2494196	1	A	C	0.00680777	-4.78E-06	0.00130899	6.25E-05	2.00E-07	0.939046	5.86E-05	27.04812046
Falls	Severe stress	rs7559374	2	A	C	0.0117244	0.000155462	0.00223021	0.00010611	1.50E-07	0.142896	5.99E-05	27.63692602
Falls	Severe stress	rs7616516	3	A	G	0.0116807	-0.0001415	0.00228799	0.000109243	3.30E-07	0.195224	5.64E-05	26.06330558
Falls	Severe stress	rs7636391	3	A	G	-0.00605272	-5.80E-05	0.00119681	5.73E-05	4.30E-07	0.311534	5.54E-05	25.57706747
Falls	Severe stress	rs171697	5	G	C	0.00721403	-3.12E-05	0.00125879	6.02E-05	1.00E-08	0.60425	7.11E-05	32.84349151
Falls	Severe stress	rs686	5	A	G	0.00630223	-4.79E-05	0.001214	5.81E-05	2.10E-07	0.409519	5.84E-05	26.94952556
Falls	Severe stress	rs2709062	7	A	G	0.00675903	7.58E-05	0.00118653	5.68E-05	1.20E-08	0.181692	7.03E-05	32.44974598
Falls	Severe stress	rs17695376	8	C	G	-0.0079875	-7.09E-05	0.00146714	7.03E-05	5.20E-08	0.313545	6.42E-05	29.64002917
Falls	Severe stress	rs10858334	9	G	C	0.00901925	-2.19E-05	0.00170213	8.14E-05	1.20E-07	0.788248	6.08E-05	28.07730358
Falls	Severe stress	rs494221	11	G	A	-0.00679158	-3.49E-05	0.00125012	5.97E-05	5.50E-08	0.558433	6.39E-05	29.5146906
Falls	Severe stress	rs71480157	11	C	T	-0.0088994	2.00E-05	0.00154595	7.37E-05	8.60E-09	0.785778	7.18E-05	33.13832612
Falls	Severe stress	rs12884871	14	T	C	-0.00694016	6.34E-05	0.00129401	6.17E-05	8.20E-08	0.303591	6.23E-05	28.76495512
Falls	Severe stress	rs11645704	16	T	G	0.00655757	-2.36E-05	0.00129264	6.19E-05	3.90E-07	0.702723	5.57E-05	25.73538614
Falls	Severe stress	rs28633123	19	T	C	0.00832889	0.000155188	0.0014539	6.99E-05	1.00E-08	0.0264527	7.11E-05	32.81747625

Falls	Severe stress	rs8105753	19	C	A	0.00659603	-0.000109349	0.00123595	5.92E-05	9.50E-08	0.0648396	6.17E-05	28.48153822
Falls	Severe stress	rs2281147	20	A	T	-0.00847023	8.81E-05	0.00157873	7.54E-05	8.10E-08	0.242142	6.23E-05	28.78556002
Hip circumference	Fracture	rs10493979	1	G	T	-0.0139097	0.000270328	0.00262114	0.000670887	1.12E-07	0.69	8.37E-05	28.16145508
Hip circumference	Fracture	rs10493988	1	G	A	0.0132618	-0.0014628	0.002503	0.000640996	1.17E-07	0.0219999	8.34E-05	28.07263952
Hip circumference	Fracture	rs112646560	1	T	C	0.0208306	-0.000747613	0.00294004	0.00075246	1.39E-12	0.32	0.000149	50.19923289
Hip circumference	Fracture	rs1127100	1	C	T	0.0149329	0.000270025	0.00252469	0.000645247	3.33E-09	0.68	0.000104	34.98421988
Hip circumference	Fracture	rs11584359	1	T	C	-0.0211394	0.000376222	0.0031669	0.00081106	2.47E-11	0.64	0.000132	44.55706881
Hip circumference	Fracture	rs11803990	1	G	C	0.0252396	-0.000203369	0.00438591	0.00113359	8.69E-09	0.86	9.84E-05	33.11658186
Hip circumference	Fracture	rs12096864	1	C	T	0.0219612	0.000674678	0.00376456	0.000963869	5.43E-09	0.48	0.000101	34.03170339
Hip circumference	Fracture	rs12140153	1	T	G	-0.0248568	0.00147899	0.00417296	0.00108084	2.58E-09	0.17	0.000105	35.48150169
Hip circumference	Fracture	rs1229128	1	A	G	0.0158537	-0.00123248	0.00304025	0.000778407	1.84E-07	0.11	8.08E-05	27.19209553
Hip circumference	Fracture	rs12561919	1	T	C	0.0200114	0.00128064	0.00338817	0.000871474	3.50E-09	0.14	0.000104	34.88386206
Hip circumference	Fracture	rs16825336	1	A	G	0.0213158	-0.00138321	0.00414967	0.0010669	2.80E-07	0.19	7.84E-05	26.38615755
Hip circumference	Fracture	rs17024393	1	C	T	0.0568069	0.00158019	0.0076056	0.00194381	8.09E-14	0.42	0.000166	55.78728401
Hip circumference	Fracture	rs2494196	1	A	C	0.0320476	-0.000401153	0.00265572	0.000681601	1.60E-33	0.56	0.000432	145.6218199
Hip circumference	Fracture	rs2678204	1	G	T	0.0216541	6.40E-05	0.00253869	0.000650419	1.47E-17	0.92	0.000216	72.75467943
Hip circumference	Fracture	rs2761185	1	A	G	-0.0318384	-0.000742309	0.00611646	0.00153685	1.94E-07	0.630001	8.05E-05	27.0958132
Hip circumference	Fracture	rs2802774	1	A	C	0.016994	0.000179422	0.00245316	0.000628339	4.29E-12	0.780001	0.000143	47.98875451
Hip circumference	Fracture	rs2815753	1	A	G	0.0218333	8.18E-05	0.00245234	0.000628979	5.46E-19	0.9	0.000235	79.26425837
Hip circumference	Fracture	rs33955687	1	A	C	-0.0160724	0.00103987	0.00270852	0.000694108	2.96E-09	0.13	0.000105	35.21254144
Hip circumference	Fracture	rs34517439	1	A	C	0.0429378	0.00187516	0.00368662	0.000952984	2.41E-31	0.0490004	0.000403	135.6509463
Hip circumference	Fracture	rs3845344	1	T	C	0.0146464	0.000221026	0.00246158	0.000631005	2.68E-09	0.73	0.000105	35.40249517
Hip circumference	Fracture	rs4471313	1	T	G	-0.0170096	-0.00027465	0.00272413	0.000696001	4.27E-10	0.69	0.000116	38.9881424
Hip circumference	Fracture	rs4660586	1	T	C	-0.019614	-0.000348173	0.00274699	0.000703652	9.34E-13	0.62	0.000151	50.98215453
Hip circumference	Fracture	rs4908677	1	T	C	0.0132201	-0.000189095	0.00242907	0.000621966	5.26E-08	0.760001	8.80E-05	29.62029775
Hip circumference	Fracture	rs543874	1	G	A	0.0442781	-0.000375634	0.00296152	0.000762854	1.59E-50	0.62	0.000664	223.536586

Hip circumference	Fracture	rs588660	1	A	G	0.0188173	-0.000402991	0.00244358	0.000625234	1.36E-14	0.52	0.000176	59.30092857
Hip circumference	Fracture	rs60226453	1	T	C	0.0177113	0.000382406	0.00316301	0.00080581	2.15E-08	0.64	9.31E-05	31.35449055
Hip circumference	Fracture	rs6669341	1	G	A	-0.0146303	0.000158116	0.00244057	0.000624536	2.04E-09	0.8	0.000107	35.93551938
Hip circumference	Fracture	rs7516554	1	T	C	0.0155427	-0.000515975	0.00245713	0.000629832	2.53E-10	0.41	0.000119	40.01258964
Hip circumference	Fracture	rs7548408	1	C	T	0.0150333	0.000448937	0.00243163	0.000622479	6.32E-10	0.47	0.000114	38.22202241
Hip circumference	Fracture	rs76798800	1	T	G	0.0254141	0.000359125	0.0027214	0.000698176	9.82E-21	0.61	0.000259	87.20968303
Hip circumference	Fracture	rs815335	1	T	C	0.0184289	-0.00102455	0.00250163	0.000641496	1.75E-13	0.11	0.000161	54.26910685
Hip circumference	Fracture	rs9424466	1	C	A	0.0138768	-0.00036523	0.00269806	0.000691639	2.70E-07	0.6	7.86E-05	26.45303164
Hip circumference	Fracture	rs1014291	2	T	G	-0.0160298	-1.43E-05	0.00243785	0.000624341	4.86E-11	0.98	0.000128	43.23567537
Hip circumference	Fracture	rs10210468	2	C	T	-0.0150017	0.000515963	0.00244268	0.000625735	8.19E-10	0.41	0.000112	37.7179256
Hip circumference	Fracture	rs1118151	2	G	T	0.016606	0.00045459	0.00269097	0.000689047	6.79E-10	0.51	0.000113	38.08135167
Hip circumference	Fracture	rs11688707	2	A	G	0.0130416	0.00116476	0.00252167	0.000646738	2.32E-07	0.0719996	7.95E-05	26.74762638
Hip circumference	Fracture	rs12467963	2	T	A	-0.013934	-0.000699019	0.00247214	0.000633186	1.74E-08	0.27	9.44E-05	31.76914223
Hip circumference	Fracture	rs12475388	2	A	G	-0.0134418	-0.00141551	0.00241781	0.000619643	2.71E-08	0.0219999	9.18E-05	30.90797267
Hip circumference	Fracture	rs12619178	2	T	C	-0.0158059	-0.000483837	0.00245114	0.000628221	1.13E-10	0.44	0.000124	41.58169853
Hip circumference	Fracture	rs12714415	2	C	T	-0.0490852	0.000916037	0.00331126	0.00084684	1.07E-49	0.28	0.000652	219.7427544
Hip circumference	Fracture	rs13389219	2	T	C	0.0263086	0.00030358	0.0024607	0.000630362	1.13E-26	0.630001	0.000339	114.3083978
Hip circumference	Fracture	rs13410783	2	G	A	0.0146689	-0.000680673	0.00249694	0.00063833	4.24E-09	0.29	0.000103	34.51269573
Hip circumference	Fracture	rs1528450	2	C	T	0.0172654	0.000173603	0.0024864	0.000637082	3.82E-12	0.79	0.000143	48.21823337
Hip circumference	Fracture	rs2193618	2	C	T	0.0133907	-0.000528575	0.00247204	0.000633239	6.07E-08	0.4	8.72E-05	29.34239595
Hip circumference	Fracture	rs2244786	2	A	G	0.0139551	-0.00143312	0.00251229	0.000644062	2.78E-08	0.0259998	9.17E-05	30.85505796
Hip circumference	Fracture	rs2693823	2	A	T	-0.0150177	0.000888112	0.00276473	0.000705011	5.58E-08	0.21	8.76E-05	29.50539267
Hip circumference	Fracture	rs2861690	2	G	C	-0.0171381	-0.00148117	0.00246592	0.000631279	3.66E-12	0.0189998	0.000143	48.30225198
Hip circumference	Fracture	rs34168749	2	T	C	-0.0125537	-0.00041991	0.0024143	0.000618653	2.00E-07	0.5	8.03E-05	27.03715701
Hip circumference	Fracture	rs35882248	2	T	C	0.0170391	-4.97E-05	0.00259225	0.000662494	4.94E-11	0.94	0.000128	43.20554992
Hip circumference	Fracture	rs4430895	2	T	C	0.0241932	0.000528978	0.00240643	0.000616616	8.93E-24	0.39	0.0003	101.0741657

Hip circumference	Fracture	rs4482463	2	A	C	-0.029946	0.000195287	0.00455065	0.00115913	4.69E-11	0.87	0.000129	43.30427499
Hip circumference	Fracture	rs4670612	2	G	A	0.0163808	-0.000695857	0.00253152	0.000649631	9.77E-11	0.28	0.000124	41.87043666
Hip circumference	Fracture	rs4832298	2	T	C	-0.013755	0.000893042	0.00259071	0.00066361	1.10E-07	0.18	8.37E-05	28.18925424
Hip circumference	Fracture	rs58584712	2	A	G	0.0198104	0.000847376	0.00295089	0.000754789	1.90E-11	0.26	0.000134	45.06926214
Hip circumference	Fracture	rs62166769	2	A	T	0.0127581	-0.000152006	0.00249432	0.000639215	3.14E-07	0.81	7.77E-05	26.16180268
Hip circumference	Fracture	rs62183012	2	C	T	-0.0134016	0.00130854	0.00265696	0.000681126	4.56E-07	0.0549997	7.56E-05	25.44153263
Hip circumference	Fracture	rs6437277	2	G	A	-0.0150561	9.80E-07	0.00286809	0.000735074	1.53E-07	1	8.19E-05	27.55747554
Hip circumference	Fracture	rs6707036	2	G	A	-0.0146292	0.000191496	0.00255395	0.000655121	1.02E-08	0.77	9.75E-05	32.81076629
Hip circumference	Fracture	rs6739755	2	G	A	-0.0152363	-0.000656753	0.00246322	0.000631167	6.20E-10	0.3	0.000114	38.26067487
Hip circumference	Fracture	rs6747657	2	A	G	0.0140974	0.000600343	0.00266546	0.000684014	1.23E-07	0.38	8.31E-05	27.9726561
Hip circumference	Fracture	rs72917533	2	C	T	-0.0164346	-0.000763169	0.00309747	0.000794682	1.12E-07	0.34	8.36E-05	28.15166326
Hip circumference	Fracture	rs75543804	2	T	G	-0.040796	-0.00080656	0.00679915	0.00175201	1.97E-09	0.649999	0.000107	36.00194145
Hip circumference	Fracture	rs77165542	2	T	C	-0.0864376	-0.000167412	0.00657899	0.00168418	2.03E-39	0.92	0.000513	172.6183445
Hip circumference	Fracture	rs968379	2	T	C	-0.0206395	-0.0023172	0.00286037	0.000735193	5.38E-13	0.0016	0.000155	52.06596419
Hip circumference	Fracture	rs11915747	3	G	C	-0.0187962	0.00116072	0.00251523	0.000644679	7.86E-14	0.0719996	0.000166	55.84505284
Hip circumference	Fracture	rs13085031	3	T	C	0.0131865	-0.000297884	0.00246501	0.000631144	8.83E-08	0.64	8.50E-05	28.61684211
Hip circumference	Fracture	rs1406779	3	T	C	0.0201178	0.000773196	0.00258241	0.000660389	6.70E-15	0.24	0.00018	60.68908407
Hip circumference	Fracture	rs1727901	3	T	C	0.0188821	0.00143769	0.00272522	0.000699481	4.26E-12	0.04	0.000143	48.00621343
Hip circumference	Fracture	rs2034768	3	G	A	-0.016569	-0.000112462	0.002406	0.000615988	5.73E-12	0.86	0.000141	47.42434593
Hip circumference	Fracture	rs2270894	3	G	C	-0.0199916	0.0016343	0.00309373	0.00079473	1.03E-10	0.04	0.000124	41.75709618
Hip circumference	Fracture	rs2371767	3	C	G	0.0198003	-0.000595065	0.00270203	0.000686748	2.34E-13	0.39	0.00016	53.69863024
Hip circumference	Fracture	rs34373881	3	A	G	-0.0149443	-0.00147838	0.00268972	0.000691205	2.76E-08	0.032	9.17E-05	30.87002704
Hip circumference	Fracture	rs35779991	3	C	T	0.0143334	0.000312069	0.00242195	0.000620805	3.26E-09	0.62	0.000104	35.02418962
Hip circumference	Fracture	rs4017425	3	T	C	-0.0138473	-0.000211154	0.00241466	0.000618072	9.78E-09	0.73	9.77E-05	32.88654326
Hip circumference	Fracture	rs55932154	3	G	A	-0.0236291	-0.0020671	0.0037729	0.000967375	3.78E-10	0.0329997	0.000117	39.22326766
Hip circumference	Fracture	rs62243489	3	G	T	-0.0153338	-0.000894238	0.00276351	0.000706495	2.88E-08	0.21	9.15E-05	30.78771856

Hip circumference	Fracture	rs62246314	3	A	G	0.0247126	0.000271161	0.00398491	0.00101531	5.60E-10	0.79	0.000114	38.45916448
Hip circumference	Fracture	rs6789488	3	C	T	0.0163656	-0.000520409	0.00278041	0.000711039	3.96E-09	0.46	0.000103	34.64544793
Hip circumference	Fracture	rs6810023	3	A	G	0.0170797	-0.000302821	0.00335675	0.000861847	3.62E-07	0.73	7.69E-05	25.88942954
Hip circumference	Fracture	rs724016	3	G	A	0.0264891	-0.00011233	0.0024177	0.000619399	6.26E-28	0.86	0.000357	120.0409945
Hip circumference	Fracture	rs73175572	3	G	A	0.0264617	-0.00128399	0.00385671	0.000988176	6.84E-12	0.19	0.00014	47.07621256
Hip circumference	Fracture	rs7426945	3	G	A	0.0156387	0.0010982	0.0024133	0.000618886	9.17E-11	0.0759994	0.000125	41.99317109
Hip circumference	Fracture	rs7610647	3	G	A	-0.0139905	-0.000985788	0.00265614	0.000679355	1.39E-07	0.15	8.24E-05	27.74371071
Hip circumference	Fracture	rs79375047	3	T	C	0.0158983	0.000323706	0.00309756	0.000797022	2.86E-07	0.68	7.83E-05	26.34279916
Hip circumference	Fracture	rs79597869	3	G	A	0.0122375	0.000615946	0.00240601	0.000616212	3.65E-07	0.32	7.69E-05	25.86965003
Hip circumference	Fracture	rs8192675	3	C	T	0.0172691	0.000202449	0.00265297	0.000678859	7.56E-11	0.77	0.000126	42.37158703
Hip circumference	Fracture	rs838204	3	G	A	0.013251	0.000747466	0.00254985	0.000653723	2.03E-07	0.25	8.02E-05	27.00648371
Hip circumference	Fracture	rs869400	3	G	T	0.0196495	0.00077837	0.00311102	0.00079443	2.69E-10	0.33	0.000119	39.89306471
Hip circumference	Fracture	rs9808900	3	T	G	0.0269504	0.00167083	0.00301663	0.000771841	4.13E-19	0.0299999	0.000237	79.81533473
Hip circumference	Fracture	rs9814633	3	A	G	0.0147487	0.000725713	0.00253421	0.000648374	5.90E-09	0.26	0.000101	33.87055266
Hip circumference	Fracture	rs9843653	3	C	T	0.0205097	-9.40E-05	0.00240714	0.000616619	1.60E-17	0.88	0.000216	72.59653899
Hip circumference	Fracture	rs10938397	4	G	A	0.0231136	-0.00130513	0.002432	0.000622581	2.03E-21	0.0359998	0.000268	90.32501558
Hip circumference	Fracture	rs1296328	4	C	A	-0.0155122	-0.000512169	0.00243358	0.000623287	1.84E-10	0.41	0.000121	40.6308152
Hip circumference	Fracture	rs13107325	4	T	C	0.042561	0.00318705	0.00457779	0.00117081	1.45E-20	0.00649995	0.000257	86.43943404
Hip circumference	Fracture	rs13151185	4	T	G	-0.0136099	-0.000154843	0.00241049	0.000617224	1.64E-08	0.8	9.47E-05	31.87859704
Hip circumference	Fracture	rs2102278	4	G	A	0.0158475	0.000217614	0.00257945	0.000659803	8.07E-10	0.74	0.000112	37.74568341
Hip circumference	Fracture	rs2318543	4	G	A	-0.0179454	-0.000198092	0.00292494	0.000749674	8.51E-10	0.79	0.000112	37.64197161
Hip circumference	Fracture	rs28418580	4	T	C	-0.0174415	0.000395916	0.00242792	0.000621133	6.80E-13	0.52	0.000153	51.60584805
Hip circumference	Fracture	rs34049648	4	A	G	0.0176063	0.00101175	0.00254462	0.000652098	4.56E-12	0.12	0.000142	47.87296462
Hip circumference	Fracture	rs34811474	4	A	G	-0.0205406	0.000451268	0.00284677	0.000731146	5.39E-13	0.54	0.000155	52.06207696
Hip circumference	Fracture	rs4240326	4	G	A	-0.0278879	-0.000426861	0.00241489	0.000618759	7.63E-31	0.49	0.000396	133.3634798
Hip circumference	Fracture	rs6535240	4	G	A	0.0144793	0.00151478	0.00259902	0.000665891	2.53E-08	0.0230001	9.22E-05	31.03672512

Hip circumference	Fracture	rs66679256	4	T	C	0.015685	0.000149421	0.00242434	0.000620364	9.83E-11	0.81	0.000124	41.85833909
Hip circumference	Fracture	rs6821305	4	C	A	0.0150058	-0.000438065	0.00245839	0.00062864	1.04E-09	0.49	0.000111	37.25776047
Hip circumference	Fracture	rs6837528	4	A	G	-0.0134485	0.000451422	0.00266145	0.000681356	4.35E-07	0.51	7.59E-05	25.53354243
Hip circumference	Fracture	rs6840236	4	C	T	0.0166995	0.00155645	0.00241801	0.000618631	4.98E-12	0.012	0.000142	47.69696562
Hip circumference	Fracture	rs73213484	4	T	A	-0.0195843	0.00016734	0.00347756	0.000885401	1.79E-08	0.85	9.42E-05	31.71515533
Hip circumference	Fracture	rs750090	4	C	T	-0.0169135	0.000447438	0.00253756	0.000649828	2.65E-11	0.49	0.000132	44.42570576
Hip circumference	Fracture	rs11745618	5	G	A	0.0125425	-0.00103812	0.00243148	0.000623166	2.49E-07	0.0959997	7.90E-05	26.60889355
Hip circumference	Fracture	rs12519997	5	A	G	-0.014272	-0.000987793	0.00242205	0.000619976	3.81E-09	0.11	0.000103	34.72189868
Hip circumference	Fracture	rs1428120	5	T	G	-0.0136416	0.00130127	0.0024288	0.000621946	1.95E-08	0.0359998	9.37E-05	31.54620444
Hip circumference	Fracture	rs1477290	5	C	T	0.0281297	0.000519339	0.00354094	0.000903743	1.96E-15	0.57	0.000187	63.10925673
Hip circumference	Fracture	rs1582931	5	A	G	-0.0196312	-0.0012049	0.00242829	0.000622151	6.27E-16	0.0530005	0.000194	65.35707274
Hip circumference	Fracture	rs185299	5	A	G	0.0144078	0.00024357	0.00271402	0.000694834	1.11E-07	0.73	8.37E-05	28.18183417
Hip circumference	Fracture	rs2307111	5	C	T	-0.0282307	0.000724303	0.00246445	0.000630462	2.24E-30	0.25	0.00039	131.2209782
Hip circumference	Fracture	rs252749	5	A	G	-0.0230008	-0.000143139	0.00278435	0.000714062	1.45E-16	0.84	0.000203	68.23987626
Hip circumference	Fracture	rs33967909	5	A	G	0.0154853	0.000528688	0.00293324	0.000748835	1.30E-07	0.48	8.28E-05	27.87045439
Hip circumference	Fracture	rs34629844	5	G	A	0.0219304	0.000437943	0.00358667	0.000920313	9.70E-10	0.630001	0.000111	37.38610882
Hip circumference	Fracture	rs3811951	5	G	A	0.0146228	0.000580883	0.00266992	0.000683902	4.33E-08	0.4	8.91E-05	29.99608551
Hip circumference	Fracture	rs3943933	5	A	T	0.0132294	0.00080316	0.00240628	0.000616129	3.85E-08	0.19	8.98E-05	30.22650771
Hip circumference	Fracture	rs40071	5	C	T	-0.0174246	0.000479754	0.00314153	0.00080496	2.92E-08	0.55	9.14E-05	30.76402897
Hip circumference	Fracture	rs4073717	5	T	G	-0.0175965	-0.00101884	0.00300343	0.000768606	4.67E-09	0.18	0.000102	34.32555428
Hip circumference	Fracture	rs4267859	5	A	C	-0.0130751	-0.000443956	0.00248944	0.000638194	1.50E-07	0.49	8.19E-05	27.58587165
Hip circumference	Fracture	rs4866585	5	G	T	0.0134649	-0.000227411	0.0024895	0.000638434	6.35E-08	0.719999	8.69E-05	29.25378084
Hip circumference	Fracture	rs4921301	5	T	C	-0.0156418	-0.00109652	0.00298724	0.000765157	1.64E-07	0.15	8.14E-05	27.41783921
Hip circumference	Fracture	rs59738707	5	A	G	-0.0232805	-0.000322894	0.0036192	0.000926778	1.26E-10	0.73	0.000123	41.37704203
Hip circumference	Fracture	rs6867299	5	C	T	0.0205436	-0.000279151	0.00250483	0.000641906	2.38E-16	0.66	0.0002	67.26615266
Hip circumference	Fracture	rs7442885	5	G	C	-0.020481	-0.000173894	0.00296022	0.000752174	4.56E-12	0.82	0.000142	47.86899814

Hip circumference	Fracture	rs74473266	5	T	C	-0.0256909	-0.00249799	0.00511014	0.00131087	4.97E-07	0.0569994	7.51E-05	25.27510919
Hip circumference	Fracture	rs75949361	5	T	C	0.0436087	-0.0033166	0.00678949	0.00175851	1.34E-10	0.0589997	0.000123	41.25456143
Hip circumference	Fracture	rs7703744	5	G	C	0.0143594	-0.00100721	0.00271623	0.000695741	1.25E-07	0.15	8.30E-05	27.94727729
Hip circumference	Fracture	rs7714611	5	G	A	-0.0129498	-0.00106424	0.00255816	0.000655922	4.15E-07	0.1	7.61E-05	25.62540361
Hip circumference	Fracture	rs9314057	5	C	T	-0.0136589	-5.37E-05	0.00241745	0.000618526	1.60E-08	0.93	9.48E-05	31.92393738
Hip circumference	Fracture	rs10947137	6	C	A	-0.0244192	-0.00071019	0.00458477	0.00117434	1.00E-07	0.55	8.43E-05	28.36793717
Hip circumference	Fracture	rs12209223	6	A	C	0.0224965	0.00175132	0.00398646	0.00102562	1.67E-08	0.0879995	9.46E-05	31.84601464
Hip circumference	Fracture	rs12528644	6	A	C	0.0227005	0.000584228	0.00267839	0.000684904	2.35E-17	0.39	0.000213	71.83286965
Hip circumference	Fracture	rs1294437	6	T	C	0.0186801	-0.000579867	0.00254412	0.000651883	2.10E-13	0.37	0.00016	53.91172269
Hip circumference	Fracture	rs1847912	6	G	A	-0.0150781	2.01E-05	0.00279356	0.000716233	6.76E-08	0.98	8.65E-05	29.13246487
Hip circumference	Fracture	rs2253310	6	G	C	0.0205891	0.00044859	0.00248737	0.000636414	1.26E-16	0.48	0.000204	68.51630625
Hip circumference	Fracture	rs2499468	6	A	C	0.0142064	0.0012229	0.00253046	0.00064692	1.98E-08	0.0589997	9.36E-05	31.51876005
Hip circumference	Fracture	rs2814943	6	A	G	0.0550792	0.00284536	0.00345869	0.000885514	4.47E-57	0.00129999	0.000753	253.6015893
Hip circumference	Fracture	rs28366156	6	C	T	-0.0295856	0.000389282	0.00355966	0.000913776	9.50E-17	0.67	0.000205	69.07863156
Hip circumference	Fracture	rs3734554	6	T	C	0.0201484	-0.000411999	0.00260948	0.000668535	1.16E-14	0.54	0.000177	59.61742076
Hip circumference	Fracture	rs390192	6	G	A	-0.0140741	-0.000198718	0.00242328	0.000620276	6.33E-09	0.75	0.0001	33.73137688
Hip circumference	Fracture	rs41271299	6	T	C	0.0407137	0.00323552	0.00543493	0.00139591	6.85E-14	0.02	0.000167	56.11684351
Hip circumference	Fracture	rs4467770	6	A	G	0.0166846	-0.000860786	0.00271997	0.000697566	8.57E-10	0.22	0.000112	37.62732725
Hip circumference	Fracture	rs4870057	6	G	A	0.0138845	0.000221941	0.00256765	0.00065841	6.40E-08	0.74	8.69E-05	29.24077247
Hip circumference	Fracture	rs62396185	6	C	G	-0.0365089	-0.000298099	0.00275766	0.00070271	5.34E-40	0.67	0.00052	175.2734183
Hip circumference	Fracture	rs62405860	6	C	T	0.0203123	0.000340077	0.00280058	0.000716446	4.09E-13	0.64	0.000156	52.60442021
Hip circumference	Fracture	rs62425398	6	A	C	0.023635	2.54E-05	0.00390514	0.00100489	1.43E-09	0.98	0.000109	36.63009097
Hip circumference	Fracture	rs675162	6	G	A	0.0179101	0.000239877	0.00241142	0.000617583	1.11E-13	0.7	0.000164	55.16330832
Hip circumference	Fracture	rs6907872	6	T	C	0.0147591	0.000659032	0.0026284	0.000673553	1.96E-08	0.33	9.37E-05	31.53093386
Hip circumference	Fracture	rs72892910	6	T	G	0.0355509	-0.000950084	0.00320796	0.00081719	1.55E-28	0.24	0.000365	122.8127088
Hip circumference	Fracture	rs7740107	6	A	T	-0.0229511	-0.00044945	0.002723	0.000698622	3.51E-17	0.52	0.000211	71.04143539

Hip circumference	Fracture	rs854917	6	T	C	-0.0147852	-2.74E-05	0.0027489	0.000702004	7.51E-08	0.97	8.59E-05	28.92920671
Hip circumference	Fracture	rs9378684	6	T	C	0.0198865	-6.57E-05	0.00302803	0.00077606	5.13E-11	0.93	0.000128	43.13167875
Hip circumference	Fracture	rs9489620	6	C	G	0.01267	0.000452785	0.00241932	0.00061956	1.63E-07	0.46	8.15E-05	27.42626061
Hip circumference	Fracture	rs9496567	6	A	G	0.021977	-6.72E-05	0.00281121	0.000719271	5.40E-15	0.93	0.000182	61.11534086
Hip circumference	Fracture	rs962554	6	C	T	-0.0218562	-0.000726753	0.0026826	0.000684564	3.73E-16	0.29	0.000197	66.38003419
Hip circumference	Fracture	rs998584	6	A	C	-0.0211236	-0.00199454	0.00241248	0.000617496	2.03E-18	0.0012	0.000228	76.66699245
Hip circumference	Fracture	rs10236214	7	T	C	0.0181559	-0.00164825	0.00252278	0.00064574	6.18E-13	0.0109999	0.000154	51.79368431
Hip circumference	Fracture	rs10237317	7	G	A	0.014219	-0.000993603	0.00244995	0.000627548	6.49E-09	0.11	0.0001	33.68400058
Hip circumference	Fracture	rs10269774	7	A	G	0.0234509	0.00133092	0.0025724	0.000656847	7.81E-20	0.0430002	0.000247	83.10784625
Hip circumference	Fracture	rs113852095	7	T	C	-0.0167002	-6.32E-05	0.00314782	0.000806801	1.13E-07	0.94	8.36E-05	28.14644591
Hip circumference	Fracture	rs11766945	7	A	G	-0.0197938	-0.00135921	0.00302094	0.000769504	5.68E-11	0.0769999	0.000128	42.93131153
Hip circumference	Fracture	rs1182199	7	A	C	-0.0279324	-0.00138875	0.00261283	0.000669568	1.14E-26	0.0379997	0.000339	114.2863065
Hip circumference	Fracture	rs12701265	7	A	G	0.0146579	0.00146232	0.00246003	0.000629929	2.55E-09	0.02	0.000105	35.50280788
Hip circumference	Fracture	rs17149254	7	C	T	-0.0194615	0.000142475	0.00311973	0.000796893	4.43E-10	0.86	0.000116	38.9151405
Hip circumference	Fracture	rs227940	7	G	T	0.0129923	-0.000204513	0.0024136	0.000617863	7.33E-08	0.74	8.61E-05	28.97620369
Hip circumference	Fracture	rs2289379	7	T	C	-0.0125447	-0.000782844	0.00246821	0.000632208	3.73E-07	0.22	7.67E-05	25.83189963
Hip circumference	Fracture	rs34748838	7	T	C	0.0204746	8.40E-05	0.00240761	0.000615982	1.84E-17	0.89	0.000215	72.32002622
Hip circumference	Fracture	rs36078773	7	G	T	0.0165822	5.22E-05	0.00243452	0.000623956	9.69E-12	0.93	0.000138	46.39354987
Hip circumference	Fracture	rs3729793	7	G	C	0.0215897	-0.000369526	0.00413262	0.0010574	1.75E-07	0.73	8.11E-05	27.29243401
Hip circumference	Fracture	rs3807566	7	T	G	-0.0159279	0.00103164	0.00242654	0.000621729	5.24E-11	0.0969996	0.000128	43.08659071
Hip circumference	Fracture	rs4722398	7	T	C	0.0222577	0.00157697	0.00350131	0.000895823	2.06E-10	0.0779992	0.00012	40.4109854
Hip circumference	Fracture	rs57116196	7	T	C	0.016147	-8.86E-05	0.00298851	0.000761029	6.56E-08	0.91	8.67E-05	29.19269997
Hip circumference	Fracture	rs58862095	7	T	C	-0.0188631	-0.000324527	0.00244247	0.000625498	1.14E-14	0.6	0.000177	59.64412311
Hip circumference	Fracture	rs6973656	7	G	A	0.0188205	-0.000659046	0.00245506	0.000628975	1.78E-14	0.29	0.000175	58.7676187
Hip circumference	Fracture	rs982692	7	C	T	0.0144682	0.000148654	0.00250216	0.000641319	7.38E-09	0.82	9.93E-05	33.43480949
Hip circumference	Fracture	rs10100245	8	A	G	0.0204889	0.000313914	0.00242571	0.000621603	3.01E-17	0.61	0.000212	71.34434061

Hip circumference	Fracture	rs10103997	8	G	C	0.0162422	-0.000397339	0.00288595	0.000739658	1.82E-08	0.59	9.41E-05	31.67467028
Hip circumference	Fracture	rs113364497	8	T	C	-0.0157612	1.29E-05	0.00282326	0.000721864	2.37E-08	0.99	9.26E-05	31.16569454
Hip circumference	Fracture	rs11997077	8	G	A	-0.0128247	-0.00163572	0.00249347	0.000638276	2.70E-07	0.01	7.86E-05	26.45368237
Hip circumference	Fracture	rs12543555	8	G	A	-0.0157172	0.000168834	0.00297541	0.000762728	1.28E-07	0.82	8.29E-05	27.90337415
Hip circumference	Fracture	rs12680342	8	G	T	-0.0186937	-0.00013488	0.00285757	0.000731802	6.09E-11	0.85	0.000127	42.79536964
Hip circumference	Fracture	rs13264909	8	T	A	-0.0157479	-0.000735628	0.00243238	0.000623244	9.54E-11	0.24	0.000125	41.91625262
Hip circumference	Fracture	rs2737250	8	G	A	-0.0213899	-0.00161122	0.00252036	0.000647024	2.13E-17	0.0129999	0.000214	72.02650664
Hip circumference	Fracture	rs2954021	8	G	A	0.0177343	0.00048791	0.00240615	0.000615905	1.71E-13	0.43	0.000161	54.32286992
Hip circumference	Fracture	rs310307	8	G	A	-0.0125543	-1.76E-05	0.00242099	0.000620273	2.15E-07	0.98	7.99E-05	26.89050843
Hip circumference	Fracture	rs4872142	8	G	C	-0.0206355	-0.000206343	0.00309464	0.000792107	2.59E-11	0.79	0.000132	44.46412222
Hip circumference	Fracture	rs6470771	8	C	A	-0.0176853	-2.74E-05	0.00320267	0.000819683	3.35E-08	0.97	9.06E-05	30.49302285
Hip circumference	Fracture	rs6601527	8	A	C	-0.0155086	0.000611163	0.00244837	0.000628118	2.39E-10	0.33	0.000119	40.12278685
Hip circumference	Fracture	rs6998644	8	T	C	-0.0128742	-0.000315791	0.00242764	0.000621399	1.14E-07	0.61	8.35E-05	28.12366614
Hip circumference	Fracture	rs6999725	8	T	C	-0.0404537	-9.91E-05	0.00670288	0.0017142	1.59E-09	0.95	0.000108	36.42450094
Hip circumference	Fracture	rs72656010	8	C	T	-0.0239918	-0.0011638	0.00357594	0.000912665	1.96E-11	0.2	0.000134	45.01375246
Hip circumference	Fracture	rs7460093	8	A	G	0.0150887	0.000456824	0.00243191	0.000622388	5.49E-10	0.46	0.000114	38.4953835
Hip circumference	Fracture	rs77978038	8	G	T	0.0145795	-0.000540771	0.00286142	0.000733997	3.49E-07	0.46	7.71E-05	25.96103746
Hip circumference	Fracture	rs7833077	8	C	G	-0.0132735	1.43E-05	0.00253124	0.000649207	1.57E-07	0.98	8.17E-05	27.49819951
Hip circumference	Fracture	rs7845090	8	A	G	-0.0226011	0.000455684	0.00266783	0.000681419	2.43E-17	0.5	0.000213	71.76998408
Hip circumference	Fracture	rs78565420	8	T	C	0.0276211	0.000971307	0.00546097	0.00141521	4.24E-07	0.49	7.60E-05	25.58246325
Hip circumference	Fracture	rs894347	8	G	A	-0.0182603	-0.000925551	0.0024579	0.000629908	1.09E-13	0.14	0.000164	55.19343169
Hip circumference	Fracture	rs10118701	9	G	A	0.0183313	0.000518111	0.00257954	0.000658168	1.19E-12	0.43	0.00015	50.50123471
Hip circumference	Fracture	rs10756798	9	T	C	-0.0154182	0.000809237	0.00251451	0.00064237	8.70E-10	0.21	0.000112	37.59764263
Hip circumference	Fracture	rs10820852	9	A	C	-0.0162227	-0.000186768	0.00270056	0.000689484	1.89E-09	0.79	0.000107	36.08598902
Hip circumference	Fracture	rs12346647	9	C	T	0.0156951	-6.69E-05	0.00302959	0.000775662	2.21E-07	0.93	7.97E-05	26.83863704
Hip circumference	Fracture	rs17770336	9	T	C	0.0205165	0.000623863	0.00256585	0.000657591	1.29E-15	0.34	0.00019	63.93578789

Hip circumference	Fracture	rs28377268	9	T	G	0.0253898	0.00117601	0.00388572	0.000994966	6.41E-11	0.24	0.000127	42.69485612
Hip circumference	Fracture	rs4297095	9	A	G	-0.0222453	0.000769186	0.0039494	0.00101892	1.78E-08	0.450001	9.42E-05	31.72592479
Hip circumference	Fracture	rs4741546	9	T	C	-0.0177033	-0.00074823	0.00246644	0.000632612	7.11E-13	0.24	0.000153	51.51899089
Hip circumference	Fracture	rs937482	9	G	A	-0.0125853	0.000191495	0.0024067	0.000616519	1.70E-07	0.760001	8.12E-05	27.34533379
Hip circumference	Fracture	rs10883553	10	A	C	0.0160261	6.23E-05	0.00242185	0.00061993	3.66E-11	0.92	0.00013	43.78861555
Hip circumference	Fracture	rs10887571	10	T	C	0.0145648	0.000274126	0.00243391	0.000623901	2.18E-09	0.66	0.000106	35.80964541
Hip circumference	Fracture	rs11146442	10	A	C	-0.0209335	-0.00177424	0.00384937	0.000987627	5.39E-08	0.0719996	8.79E-05	29.57361256
Hip circumference	Fracture	rs12254441	10	T	C	-0.0149948	-0.000463324	0.00257352	0.000659605	5.66E-09	0.48	0.000101	33.9489432
Hip circumference	Fracture	rs12414412	10	G	C	0.0231133	0.000118227	0.00434374	0.00110467	1.03E-07	0.91	8.41E-05	28.31367667
Hip circumference	Fracture	rs12765337	10	C	G	0.0155046	-0.000650739	0.00253466	0.000647977	9.54E-10	0.32	0.000111	37.41809821
Hip circumference	Fracture	rs12779865	10	C	T	0.0226272	0.000789587	0.00255912	0.000654934	9.47E-19	0.23	0.000232	78.17723918
Hip circumference	Fracture	rs2439823	10	G	A	0.0188359	0.00155348	0.00242211	0.000620184	7.47E-15	0.012	0.00018	60.4762741
Hip circumference	Fracture	rs6585201	10	A	G	-0.0208428	0.00090526	0.00241636	0.000619161	6.40E-18	0.14	0.000221	74.40272582
Hip circumference	Fracture	rs7087701	10	G	C	-0.0129751	-0.000259027	0.00247335	0.00063351	1.56E-07	0.68	8.18E-05	27.52011683
Hip circumference	Fracture	rs7893571	10	T	G	0.0156277	0.00128231	0.00255052	0.000654558	8.95E-10	0.05	0.000112	37.54331907
Hip circumference	Fracture	rs7915723	10	A	C	-0.0137936	0.00042535	0.0024247	0.000621099	1.28E-08	0.49	9.61E-05	32.3622889
Hip circumference	Fracture	rs79969674	10	T	C	0.0265356	-0.000321059	0.00462325	0.00118635	9.50E-09	0.79	9.79E-05	32.94299401
Hip circumference	Fracture	rs845084	10	A	G	0.0180027	-0.000839495	0.00275957	0.000706301	6.87E-11	0.23	0.000126	42.5591023
Hip circumference	Fracture	rs9415106	10	A	G	-0.0151806	-0.00093209	0.00266693	0.000682221	1.26E-08	0.17	9.62E-05	32.40071847
Hip circumference	Fracture	rs11030119	11	A	G	0.0314539	-7.50E-05	0.002604	0.00066816	1.38E-33	0.91	0.000433	145.903948
Hip circumference	Fracture	rs12805742	11	T	C	-0.0185191	5.75E-06	0.00285874	0.000733506	9.30E-11	0.99	0.000125	41.96531011
Hip circumference	Fracture	rs140201358	11	G	C	-0.0637486	-0.00295611	0.010272	0.00264688	5.44E-10	0.26	0.000114	38.51512234
Hip circumference	Fracture	rs143840904	11	T	C	-0.0483109	0.00277196	0.00902762	0.00228546	8.73E-08	0.23	8.51E-05	28.63806802
Hip circumference	Fracture	rs1662185	11	G	A	0.0170127	-0.000337071	0.00265128	0.000676748	1.39E-10	0.62	0.000122	41.17516006
Hip circumference	Fracture	rs17245511	11	A	G	-0.0199228	-0.00126988	0.00338136	0.000866298	3.82E-09	0.14	0.000103	34.71506068
Hip circumference	Fracture	rs2187449	11	A	G	0.0163764	7.62E-05	0.00286336	0.000733693	1.07E-08	0.92	9.72E-05	32.71033317

Hip circumference	Fracture	rs35099456	11	C	G	-0.0406467	-0.000710164	0.00498534	0.00128393	3.55E-16	0.58	0.000197	66.47540917
Hip circumference	Fracture	rs4148172	11	G	A	-0.0235908	0.003523	0.00469185	0.00119067	4.96E-07	0.00309999	7.51E-05	25.28116594
Hip circumference	Fracture	rs4936671	11	G	C	-0.0127849	-0.000380369	0.00252321	0.000646297	4.05E-07	0.56	7.63E-05	25.67366538
Hip circumference	Fracture	rs667515	11	C	G	-0.0139118	0.000522647	0.00247623	0.000634671	1.93E-08	0.41	9.38E-05	31.56346615
Hip circumference	Fracture	rs7116641	11	G	T	0.0218983	-0.00086066	0.00258946	0.000663261	2.76E-17	0.19	0.000212	71.51586384
Hip circumference	Fracture	rs7124681	11	A	C	0.019971	-0.00214814	0.00244272	0.000625061	2.95E-16	0.000589997	0.000199	66.84243261
Hip circumference	Fracture	rs74749286	11	A	G	0.0306898	0.0014744	0.00389923	0.00100018	3.54E-15	0.14	0.000184	61.94844508
Hip circumference	Fracture	rs7480253	11	T	C	0.0125489	-0.000986977	0.0024362	0.000623784	2.59E-07	0.11	7.88E-05	26.53294393
Hip circumference	Fracture	rs7930275	11	T	C	0.0186124	-0.00045006	0.0029418	0.000755135	2.50E-10	0.55	0.000119	40.0293435
Hip circumference	Fracture	rs7940866	11	A	T	0.0129611	-4.39E-05	0.00241693	0.000619132	8.21E-08	0.94	8.54E-05	28.75779463
Hip circumference	Fracture	rs10777859	12	G	A	-0.0154525	-0.000342142	0.00240837	0.000616957	1.40E-10	0.58	0.000122	41.16717703
Hip circumference	Fracture	rs11056870	12	T	G	0.0173557	-0.000949371	0.00341687	0.000875357	3.79E-07	0.28	7.66E-05	25.80045457
Hip circumference	Fracture	rs11109097	12	T	C	-0.0156389	-0.00107359	0.0026263	0.00067257	2.61E-09	0.11	0.000105	35.45877582
Hip circumference	Fracture	rs12300276	12	A	G	0.0159752	0.00126469	0.00283175	0.000728754	1.69E-08	0.0830004	9.45E-05	31.82605358
Hip circumference	Fracture	rs1271309	12	G	A	-0.0219913	-0.000559704	0.00325813	0.000837366	1.48E-11	0.5	0.000135	45.55803551
Hip circumference	Fracture	rs1458156	12	T	C	0.0133122	0.000147602	0.0024078	0.000616451	3.23E-08	0.81	9.08E-05	30.56742444
Hip circumference	Fracture	rs147730268	12	T	G	-0.0522646	0.00055661	0.00433365	0.00111597	1.74E-33	0.62	0.000432	145.4479469
Hip circumference	Fracture	rs1964599	12	T	C	-0.022999	-0.00162679	0.0025863	0.000661029	6.00E-19	0.0140001	0.000235	79.07880664
Hip circumference	Fracture	rs1979440	12	C	T	-0.0149999	-0.000887459	0.00245372	0.000627064	9.78E-10	0.16	0.000111	37.37031184
Hip circumference	Fracture	rs2013002	12	C	T	0.018078	-0.00179808	0.00245987	0.000628512	2.00E-13	0.00420001	0.00016	54.01028291
Hip circumference	Fracture	rs2129869	12	T	A	-0.0214884	-0.000141974	0.00291524	0.000744125	1.70E-13	0.85	0.000161	54.33248024
Hip circumference	Fracture	rs2897968	12	A	G	0.0137469	5.31E-05	0.00246483	0.00063182	2.45E-08	0.93	9.24E-05	31.10538667
Hip circumference	Fracture	rs34911908	12	A	G	0.0130448	0.000219707	0.00248022	0.00063502	1.44E-07	0.73	8.22E-05	27.66269189
Hip circumference	Fracture	rs36120387	12	T	C	-0.0203173	-0.000462293	0.00388325	0.000995562	1.68E-07	0.64	8.13E-05	27.37419047
Hip circumference	Fracture	rs3730071	12	A	C	-0.0378377	0.00313648	0.00701291	0.00179861	6.84E-08	0.0810009	8.65E-05	29.11071874
Hip circumference	Fracture	rs4842681	12	G	C	0.0152955	0.0004231	0.00268676	0.000688431	1.25E-08	0.54	9.63E-05	32.40929685

Hip circumference	Fracture	rs55726687	12	A	G	0.0213623	-0.00138446	0.00294561	0.000755705	4.11E-13	0.0669993	0.000156	52.59512946
Hip circumference	Fracture	rs6539064	12	G	C	-0.0187741	0.000130859	0.00276939	0.000708059	1.21E-11	0.85	0.000137	45.9568247
Hip circumference	Fracture	rs66630777	12	T	G	-0.0150299	0.000361191	0.0027754	0.000712576	6.12E-08	0.61	8.71E-05	29.32655263
Hip circumference	Fracture	rs697883	12	A	G	-0.0223877	-0.0011524	0.00429796	0.00110127	1.90E-07	0.3	8.06E-05	27.13277533
Hip circumference	Fracture	rs7132908	12	A	G	0.0248529	-0.00123598	0.00247489	0.000633305	1.00E-23	0.051	0.0003	100.8422072
Hip circumference	Fracture	rs7305790	12	C	A	0.013906	-0.000454102	0.00268774	0.00068635	2.29E-07	0.51	7.95E-05	26.76886397
Hip circumference	Fracture	rs76895963	12	G	T	0.0962201	0.00802766	0.00932168	0.00238506	5.64E-25	0.000759994	0.000316	106.5474911
Hip circumference	Fracture	rs78470967	12	A	T	0.0359219	0.0022329	0.00606978	0.00155561	3.26E-09	0.15	0.000104	35.02456084
Hip circumference	Fracture	rs882378	12	C	A	0.0154797	0.0010524	0.00261008	0.000669855	3.02E-09	0.12	0.000104	35.17365032
Hip circumference	Fracture	rs11839227	13	C	T	-0.0181298	-0.000325509	0.00311049	0.000798125	5.59E-09	0.68	0.000101	33.97257041
Hip circumference	Fracture	rs12877270	13	A	G	0.0151117	-0.000658546	0.00244173	0.000624382	6.06E-10	0.29	0.000114	38.30287457
Hip circumference	Fracture	rs1441264	13	A	G	0.0168087	0.000336339	0.00249936	0.000640506	1.76E-11	0.6	0.000134	45.22833725
Hip circumference	Fracture	rs1886558	13	G	A	-0.0159051	-0.000333712	0.00248456	0.00063666	1.54E-10	0.6	0.000122	40.980177
Hip circumference	Fracture	rs41284816	13	T	G	0.0791573	0.000231467	0.00891062	0.00230512	6.51E-19	0.92	0.000234	78.91618788
Hip circumference	Fracture	rs7982447	13	C	T	0.0162648	0.00180191	0.0029811	0.000763868	4.87E-08	0.0179999	8.84E-05	29.76763731
Hip circumference	Fracture	rs9512696	13	G	A	0.0176296	-0.00153564	0.00254397	0.000651499	4.22E-12	0.0179999	0.000143	48.02428926
Hip circumference	Fracture	rs9513030	13	C	G	-0.0132237	8.12E-05	0.00248635	0.000636543	1.05E-07	0.9	8.40E-05	28.28664557
Hip circumference	Fracture	rs9565536	13	A	T	-0.0143477	-0.000217077	0.00269818	0.000690033	1.05E-07	0.75	8.40E-05	28.27631018
Hip circumference	Fracture	rs9603697	13	T	C	0.0139508	-0.000141287	0.00256358	0.000657195	5.27E-08	0.83	8.80E-05	29.61450486
Hip circumference	Fracture	rs1285997	14	G	C	0.0234272	-0.000705804	0.0026491	0.000679712	9.33E-19	0.3	0.000232	78.20671877
Hip circumference	Fracture	rs12883788	14	T	C	0.0154765	0.00103713	0.00242117	0.000620009	1.64E-10	0.0940005	0.000121	40.85967764
Hip circumference	Fracture	rs1955695	14	G	A	-0.0191353	0.00106566	0.00248689	0.000635465	1.42E-14	0.0940005	0.000176	59.2048655
Hip circumference	Fracture	rs28479795	14	T	C	0.0244818	-0.000165732	0.00289895	0.000742899	3.05E-17	0.82	0.000212	71.31899944
Hip circumference	Fracture	rs3803286	14	G	A	-0.0175828	-0.000702847	0.00254731	0.00065286	5.12E-12	0.28	0.000142	47.64446682
Hip circumference	Fracture	rs6575340	14	A	G	0.0204335	-0.00105105	0.0025066	0.000641476	3.60E-16	0.1	0.000197	66.45313187
Hip circumference	Fracture	rs71413981	14	A	G	0.0191825	0.000418473	0.00325375	0.00083657	3.74E-09	0.62	0.000103	34.7569814

Hip circumference	Fracture	rs7145337	14	T	C	-0.0159056	-0.000277588	0.00265409	0.000678677	2.06E-09	0.68	0.000107	35.91441705
Hip circumference	Fracture	rs8011368	14	T	C	-0.016411	1.64E-05	0.00270853	0.00069426	1.37E-09	0.98	0.000109	36.71155585
Hip circumference	Fracture	rs9323375	14	A	T	0.0164338	-0.000291309	0.00284639	0.000729541	7.77E-09	0.69	9.90E-05	33.33398108
Hip circumference	Fracture	rs9788550	14	C	G	-0.0207023	0.00149685	0.00279459	0.000716073	1.29E-13	0.0369999	0.000163	54.87834355
Hip circumference	Fracture	rs1559676	15	G	C	-0.0142302	4.10E-05	0.00265959	0.000680486	8.78E-08	0.95	8.50E-05	28.62810657
Hip circumference	Fracture	rs2715439	15	T	C	0.0157972	-0.00028775	0.00241641	0.000619422	6.27E-11	0.64	0.000127	42.73847135
Hip circumference	Fracture	rs34769775	15	T	C	-0.0165941	-0.0011105	0.00262638	0.000673951	2.65E-10	0.0990011	0.000119	39.92016069
Hip circumference	Fracture	rs35874463	15	G	A	0.0325725	0.00443511	0.00514563	0.00131967	2.45E-10	0.000779992	0.000119	40.07052901
Hip circumference	Fracture	rs4776970	15	T	A	-0.0201543	-0.000687375	0.002508	0.000641145	9.31E-16	0.28	0.000192	64.5773729
Hip circumference	Fracture	rs4777541	15	T	C	0.0191721	-0.000849152	0.00284958	0.000727864	1.72E-11	0.24	0.000134	45.26657785
Hip circumference	Fracture	rs4843158	15	C	G	0.0202544	0.00182189	0.00258511	0.000663192	4.70E-15	0.00599998	0.000182	61.38760779
Hip circumference	Fracture	rs4966012	15	G	C	0.0149488	1.67E-05	0.00257511	0.000659532	6.44E-09	0.98	0.0001	33.69931649
Hip circumference	Fracture	rs55707100	15	T	C	-0.0400543	0.0030602	0.00774721	0.00193211	2.34E-07	0.11	7.94E-05	26.73053407
Hip circumference	Fracture	rs8023263	15	T	G	0.0148049	-0.00185789	0.00240753	0.000617298	7.79E-10	0.00259998	0.000112	37.81529925
Hip circumference	Fracture	rs8042404	15	A	G	0.0170942	-0.000445488	0.00270872	0.000692414	2.78E-10	0.52	0.000118	39.82624242
Hip circumference	Fracture	rs11150461	16	G	C	-0.0150251	-3.81E-05	0.00271243	0.000694424	3.04E-08	0.96	9.12E-05	30.68440201
Hip circumference	Fracture	rs1150188	16	C	G	-0.0144553	-0.0005136	0.0024709	0.00063353	4.91E-09	0.42	0.000102	34.22503333
Hip circumference	Fracture	rs117342986	16	T	C	0.0410233	0.00230912	0.00782839	0.00201587	1.60E-07	0.25	8.16E-05	27.46099563
Hip circumference	Fracture	rs12920259	16	A	G	-0.0164136	-0.00171857	0.00247121	0.000632485	3.10E-11	0.00659994	0.000131	44.11521351
Hip circumference	Fracture	rs13333747	16	C	T	-0.0239707	-0.00084191	0.00313212	0.000800913	1.97E-14	0.29	0.000174	58.57127093
Hip circumference	Fracture	rs25849	16	G	C	0.020294	0.00138026	0.00266649	0.000682353	2.73E-14	0.0430002	0.000172	57.92357968
Hip circumference	Fracture	rs35057083	16	T	C	0.0144062	0.000459902	0.00260593	0.000665517	3.24E-08	0.49	9.08E-05	30.56141008
Hip circumference	Fracture	rs4402589	16	G	T	0.0295957	0.00149849	0.00241926	0.000619067	2.10E-34	0.015	0.000444	149.6553142
Hip circumference	Fracture	rs4985407	16	G	A	0.0153191	-0.000632667	0.00240871	0.000617251	2.02E-10	0.31	0.00012	40.448039
Hip circumference	Fracture	rs56094641	16	G	A	0.0610681	-0.00110399	0.00245004	0.000627387	5.26E-137	0.0779992	0.001842	621.2729808
Hip circumference	Fracture	rs62037365	16	G	C	0.0322128	0.00100169	0.00245225	0.000628831	2.09E-39	0.11	0.000512	172.5549674

Hip circumference	Fracture	rs72801854	16	A	G	0.0183289	0.000463766	0.00261408	0.000669631	2.36E-12	0.49	0.000146	49.162624
Hip circumference	Fracture	rs756717	16	A	G	-0.015681	0.00075611	0.00248428	0.000636592	2.76E-10	0.23	0.000118	39.84248553
Hip circumference	Fracture	rs879620	16	T	C	0.027011	0.000667307	0.00248095	0.000634343	1.34E-27	0.29	0.000352	118.5346447
Hip circumference	Fracture	rs10153248	17	G	A	-0.0170889	0.00157185	0.0024225	0.000620095	1.74E-12	0.0109999	0.000148	49.76232294
Hip circumference	Fracture	rs113866544	17	C	T	0.0327216	0.00129784	0.00479193	0.00122153	8.60E-12	0.29	0.000139	46.62814434
Hip circumference	Fracture	rs1396513	17	T	C	0.0183151	0.000804246	0.00240892	0.000616875	2.90E-14	0.19	0.000172	57.80612182
Hip circumference	Fracture	rs1918249	17	A	T	0.0159582	-0.000108783	0.00281683	0.000717443	1.47E-08	0.88	9.53E-05	32.09567639
Hip circumference	Fracture	rs2355374	17	T	G	0.0128665	0.000522552	0.00243068	0.00062235	1.20E-07	0.4	8.32E-05	28.01981565
Hip circumference	Fracture	rs3826408	17	T	C	0.0151967	0.000359633	0.00241335	0.00061758	3.04E-10	0.56	0.000118	39.65134822
Hip circumference	Fracture	rs4790292	17	A	C	-0.027819	0.0017191	0.00334073	0.000857121	8.31E-17	0.0449997	0.000206	69.34262448
Hip circumference	Fracture	rs4794222	17	G	A	-0.0174748	0.0002308	0.00276456	0.000710034	2.60E-10	0.75	0.000119	39.95511526
Hip circumference	Fracture	rs4889867	17	T	C	-0.0165547	0.000558073	0.00242054	0.000620182	7.97E-12	0.37	0.000139	46.77546378
Hip circumference	Fracture	rs56288810	17	G	A	0.0183373	-0.00134415	0.00294765	0.000755656	4.95E-10	0.0749998	0.000115	38.70071163
Hip circumference	Fracture	rs6501601	17	A	G	-0.01902	0.00171808	0.00246888	0.000632717	1.32E-14	0.00659994	0.000176	59.3500463
Hip circumference	Fracture	rs7226064	17	G	A	-0.01458	-0.000779392	0.00243911	0.000624417	2.27E-09	0.21	0.000106	35.73158444
Hip circumference	Fracture	rs731758	17	G	C	-0.0176334	0.000836686	0.00248153	0.000636097	1.20E-12	0.19	0.00015	50.49321905
Hip circumference	Fracture	rs11661691	18	G	T	0.0130311	0.000338217	0.00240751	0.000616747	6.21E-08	0.58	8.70E-05	29.2971892
Hip circumference	Fracture	rs11664106	18	T	A	0.015224	-3.49E-05	0.002547	0.000652045	2.27E-09	0.96	0.000106	35.7272559
Hip circumference	Fracture	rs12607512	18	G	A	0.0131918	-0.000326902	0.00241734	0.000619602	4.84E-08	0.6	8.85E-05	29.7805449
Hip circumference	Fracture	rs12965488	18	T	C	0.0168958	-6.56E-05	0.00312596	0.000799132	6.49E-08	0.93	8.68E-05	29.21397728
Hip circumference	Fracture	rs1618725	18	T	C	-0.021172	-0.00115144	0.00240567	0.000616029	1.36E-18	0.0619998	0.00023	77.45539373
Hip circumference	Fracture	rs273759	18	G	A	-0.0129908	0.000618377	0.00241253	0.000618041	7.26E-08	0.32	8.61E-05	28.99521599
Hip circumference	Fracture	rs57636386	18	C	T	-0.036841	0.00172047	0.004358	0.00111468	2.83E-17	0.12	0.000212	71.46417233
Hip circumference	Fracture	rs6567160	18	C	T	0.0496794	-0.000447508	0.00283765	0.000728569	1.35E-68	0.54	0.00091	306.5032107
Hip circumference	Fracture	rs7238896	18	G	A	0.0212081	0.000593005	0.00347746	0.00089184	1.07E-09	0.51	0.00011	37.194544
Hip circumference	Fracture	rs7241211	18	T	C	0.0130235	0.000875102	0.00243868	0.000623907	9.28E-08	0.16	8.47E-05	28.51975534

Hip circumference	Fracture	rs8087074	18	T	G	0.0144204	-0.00127905	0.00275049	0.000703431	1.58E-07	0.0690001	8.17E-05	27.48745136
Hip circumference	Fracture	rs8097809	18	A	G	-0.017963	1.40E-05	0.00355561	0.000903696	4.37E-07	0.99	7.58E-05	25.52286961
Hip circumference	Fracture	rs9967367	18	T	C	-0.0164671	-0.000573002	0.00265273	0.000680319	5.39E-10	0.4	0.000114	38.53435978
Hip circumference	Fracture	rs10404726	19	T	C	-0.0166254	-0.00144064	0.00240995	0.000618011	5.26E-12	0.02	0.000141	47.59136285
Hip circumference	Fracture	rs11882409	19	A	C	0.0181856	0.00128169	0.00270443	0.000692616	1.77E-11	0.064	0.000134	45.21721177
Hip circumference	Fracture	rs1231281	19	A	G	-0.013948	-0.000134453	0.00240776	0.000615563	6.92E-09	0.83	9.97E-05	33.5581095
Hip circumference	Fracture	rs12972720	19	C	G	0.0177427	3.77E-05	0.00243123	0.000623391	2.93E-13	0.95	0.000158	53.25830331
Hip circumference	Fracture	rs141622900	19	A	G	0.0316845	-0.00197389	0.00551034	0.00141513	8.93E-09	0.16	9.82E-05	33.06259391
Hip circumference	Fracture	rs2238689	19	C	T	-0.0126136	-0.000485626	0.00245659	0.000628616	2.83E-07	0.44	7.83E-05	26.36408784
Hip circumference	Fracture	rs273505	19	C	T	0.0147072	-0.000916377	0.00243691	0.000623503	1.59E-09	0.14	0.000108	36.42344472
Hip circumference	Fracture	rs3218036	19	A	G	0.0194082	6.57E-05	0.0025688	0.000658298	4.19E-14	0.92	0.00017	57.08341315
Hip circumference	Fracture	rs34013042	19	T	C	0.0154229	0.000653508	0.00275189	0.000704172	2.09E-08	0.35	9.33E-05	31.41014529
Hip circumference	Fracture	rs350832	19	A	G	0.0151016	0.00114446	0.00287737	0.000736534	1.53E-07	0.12	8.18E-05	27.54574375
Hip circumference	Fracture	rs3810291	19	A	G	0.023107	0.00211877	0.0025705	0.000656728	2.50E-19	0.00129999	0.00024	80.80754491
Hip circumference	Fracture	rs1293395	20	T	G	-0.0274278	0.00062999	0.00453926	0.00116246	1.52E-09	0.59	0.000108	36.50999975
Hip circumference	Fracture	rs143384	20	G	A	0.0281276	-0.000164079	0.00244924	0.000626742	1.60E-30	0.79	0.000392	131.8872059
Hip circumference	Fracture	rs2236519	20	A	G	-0.0208582	0.000710638	0.00248544	0.000636836	4.79E-17	0.26	0.000209	70.42828092
Hip circumference	Fracture	rs3746759	20	G	T	-0.017084	0.000476545	0.00299536	0.000761481	1.17E-08	0.53	9.66E-05	32.52977607
Hip circumference	Fracture	rs55650227	20	C	G	-0.0198726	0.000344302	0.00307086	0.000786056	9.73E-11	0.66	0.000124	41.87832926
Hip circumference	Fracture	rs55886426	20	G	C	-0.0337871	-0.000703435	0.00552551	0.00143181	9.68E-10	0.62	0.000111	37.3901404
Hip circumference	Fracture	rs6029180	20	G	A	0.0136196	0.000999282	0.00259026	0.000663155	1.46E-07	0.13	8.21E-05	27.64661645
Hip circumference	Fracture	rs6080646	20	A	G	-0.0133714	3.66E-06	0.00241122	0.000617622	2.93E-08	1	9.14E-05	30.75247591
Hip circumference	Fracture	rs6111562	20	T	G	0.0334476	-0.000492879	0.00616057	0.00157996	5.66E-08	0.760001	8.76E-05	29.47732898
Hip circumference	Fracture	rs6142059	20	C	T	0.0159821	0.000525478	0.00240831	0.000617129	3.22E-11	0.39	0.000131	44.03955373
Hip circumference	Fracture	rs7274811	20	T	G	-0.0211475	0.000101059	0.00275032	0.000703006	1.49E-14	0.89	0.000176	59.12233982
Hip circumference	Fracture	rs10427502	21	A	G	-0.0129865	0.00097903	0.00248172	0.000635634	1.67E-07	0.12	8.13E-05	27.38285195

Hip circumference	Fracture	rs60984707	21	T	C	-0.018209	-0.00150573	0.00313296	0.000800574	6.18E-09	0.0599998	0.0001	33.7802214
Hip circumference	Fracture	rs76040172	21	A	G	-0.034453	0.000721935	0.0053139	0.0013709	8.97E-11	0.6	0.000125	42.03657594
Hip circumference	Fracture	rs8133137	21	G	A	0.0150007	0.000676067	0.00255038	0.00065181	4.06E-09	0.3	0.000103	34.59499429
Hip circumference	Fracture	rs11704728	22	T	C	0.0152795	-0.000532357	0.00303796	0.000777717	4.92E-07	0.49	7.51E-05	25.29613573
Hip circumference	Fracture	rs133015	22	G	C	0.0129392	0.000735806	0.00242369	0.000621201	9.37E-08	0.24	8.47E-05	28.50104013
Hip circumference	Fracture	rs138767	22	C	T	0.0146128	0.000409504	0.0025195	0.000646008	6.64E-09	0.53	9.99E-05	33.63861879
Hip circumference	Fracture	rs140947018	22	A	G	0.0369121	0.000149014	0.00727511	0.00186527	3.90E-07	0.94	7.65E-05	25.7429539
Hip circumference	Fracture	rs1569497	22	G	A	0.0148474	0.000420117	0.00241868	0.000619551	8.33E-10	0.5	0.000112	37.68287163
Sleeplessness	Fracture	rs12049261	1	C	G	0.0111868	0.000666664	0.00163048	0.000676698	6.80E-12	0.32	0.000102	47.07396667
Sleeplessness	Fracture	rs2644128	1	G	C	0.0106284	0.000284878	0.00149103	0.000618811	1.00E-12	0.649999	0.00011	50.81161726
Sleeplessness	Fracture	rs2803296	1	C	G	-0.00862206	-0.00033527	0.00149047	0.000618572	7.30E-09	0.59	7.24E-05	33.46382677
Sleeplessness	Fracture	rs35267450	1	C	T	0.00892042	-0.000527657	0.00164053	0.000680859	5.40E-08	0.44	6.39E-05	29.56665898
Sleeplessness	Fracture	rs6690017	1	G	T	-0.0102669	-0.000893798	0.00151022	0.000626771	1.10E-11	0.15	1.00E-04	46.21662517
Sleeplessness	Fracture	rs76265753	1	T	C	0.0211056	0.00224687	0.00399311	0.00165686	1.30E-07	0.18	6.04E-05	27.93655551
Sleeplessness	Fracture	rs113851554	2	T	G	0.0467802	-0.000878747	0.00331329	0.00137531	2.90E-45	0.52	0.000431	199.344955
Sleeplessness	Fracture	rs12470989	2	G	A	-0.0102429	-0.00098837	0.00184485	0.000765626	2.80E-08	0.2	6.67E-05	30.82646636
Sleeplessness	Fracture	rs1867814	2	A	G	-0.00767576	0.000657209	0.0014862	0.000616815	2.40E-07	0.29	5.77E-05	26.67400696
Sleeplessness	Fracture	rs4572538	2	T	C	-0.00960596	0.000474923	0.00156161	0.000648044	7.70E-10	0.46	8.18E-05	37.83871548
Sleeplessness	Fracture	rs4577309	2	G	A	-0.00854833	-0.000296622	0.00149214	0.000619264	1.00E-08	0.630001	7.10E-05	32.8203655
Sleeplessness	Fracture	rs56093896	2	A	C	-0.0124111	0.00106771	0.00181352	0.000752582	7.70E-12	0.16	0.000101	46.83557439
Sleeplessness	Fracture	rs56365214	2	A	C	-0.0147948	0.000732792	0.00205225	0.000851596	5.60E-13	0.39	0.000112	51.97059256
Sleeplessness	Fracture	rs6543087	2	T	A	0.00766484	0.00148703	0.00151726	0.000629616	4.40E-07	0.0179999	5.52E-05	25.5203232
Sleeplessness	Fracture	rs78887635	2	C	T	-0.0116184	0.00115656	0.00224665	0.000932348	2.30E-07	0.21	5.78E-05	26.74371951
Sleeplessness	Fracture	rs2014830	3	T	C	-0.0116018	-9.38E-05	0.0016233	0.000673561	8.90E-13	0.89	0.00011	51.08026854
Sleeplessness	Fracture	rs705219	3	A	T	0.013423	0.000396146	0.00235299	0.000976252	1.20E-08	0.68	7.04E-05	32.54310725
Sleeplessness	Fracture	rs75709417	3	T	C	0.0123444	-0.00117377	0.00228118	0.000946614	6.30E-08	0.21	6.33E-05	29.28335694

Sleeplessness	Fracture	rs7625896	3	G	A	-0.00819859	-0.000349776	0.00156815	0.000650658	1.70E-07	0.59	5.91E-05	27.33399636
Sleeplessness	Fracture	rs9845387	3	A	C	-0.0218562	-0.00355618	0.00377648	0.00156701	7.10E-09	0.0230001	7.24E-05	33.49461068
Sleeplessness	Fracture	rs11097861	4	G	A	0.0100437	-0.000346581	0.00164946	0.000684268	1.10E-09	0.61	8.02E-05	37.07694031
Sleeplessness	Fracture	rs1988337	4	G	A	0.00838726	2.47E-05	0.00149645	0.000620758	2.10E-08	0.97	6.79E-05	31.41346124
Sleeplessness	Fracture	rs2604551	4	G	T	-0.00848084	-0.00148194	0.00155238	0.000644121	4.70E-08	0.021	6.45E-05	29.84569262
Sleeplessness	Fracture	rs6833329	4	G	A	-0.00820472	3.53E-05	0.00153119	0.000635251	8.40E-08	0.96	6.21E-05	28.7123911
Sleeplessness	Fracture	rs1083562	5	C	T	-0.00855653	7.17E-05	0.0016541	0.000686274	2.30E-07	0.92	5.79E-05	26.75912027
Sleeplessness	Fracture	rs1430205	5	T	C	0.00947493	-8.39E-05	0.00149107	0.000618573	2.10E-10	0.89	8.73E-05	40.37903684
Sleeplessness	Fracture	rs1592757	5	C	G	0.0102216	0.00196193	0.00154996	0.000643037	4.30E-11	0.00230001	9.41E-05	43.49073854
Sleeplessness	Fracture	rs2270914	5	T	C	-0.00922378	0.000120386	0.00175421	0.000727746	1.50E-07	0.87	5.98E-05	27.64742617
Sleeplessness	Fracture	rs36116812	5	C	T	0.00777747	0.000331688	0.00148243	0.000614998	1.60E-07	0.59	5.95E-05	27.52506154
Sleeplessness	Fracture	rs7711696	5	T	G	0.0111716	0.00184102	0.00161138	0.000668492	4.10E-12	0.00589997	0.000104	48.06564967
Sleeplessness	Fracture	rs10947690	6	G	A	0.00919149	0.00102826	0.00169355	0.000702577	5.70E-08	0.14	6.37E-05	29.45613704
Sleeplessness	Fracture	rs11963889	6	C	T	-0.0075944	8.79E-05	0.00148676	0.00061676	3.30E-07	0.89	5.64E-05	26.09186959
Sleeplessness	Fracture	rs17466209	6	T	A	0.00884283	-0.000207981	0.00166381	0.000690178	1.10E-07	0.760001	6.11E-05	28.24717961
Sleeplessness	Fracture	rs240112	6	A	C	-0.0076731	0.00145494	0.00149592	0.000620578	2.90E-07	0.0189998	5.69E-05	26.31025027
Sleeplessness	Fracture	rs314280	6	G	A	0.00971363	-0.00104496	0.00149134	0.00061871	7.30E-11	0.0909997	9.18E-05	42.42382091
Sleeplessness	Fracture	rs6938026	6	G	A	0.00984256	0.000346391	0.00182573	0.000757399	7.00E-08	0.649999	6.29E-05	29.06317374
Sleeplessness	Fracture	rs10235198	7	T	C	0.01254	0.00111316	0.00233378	0.000968112	7.70E-08	0.25	6.24E-05	28.87189207
Sleeplessness	Fracture	rs10276441	7	A	G	0.0096237	-0.000530141	0.00181232	0.000751801	1.10E-07	0.48	6.10E-05	28.19774548
Sleeplessness	Fracture	rs1731951	7	A	T	-0.00787651	0.000629667	0.00150645	0.000624929	1.70E-07	0.31	5.91E-05	27.33746336
Sleeplessness	Fracture	rs6975972	7	G	A	-0.00902066	0.00148044	0.00150428	0.000624012	2.00E-09	0.0179999	7.78E-05	35.9599654
Sleeplessness	Fracture	rs8180817	7	C	G	-0.0100553	-0.00167783	0.00150974	0.000626254	2.70E-11	0.00739997	9.59E-05	44.35940796
Sleeplessness	Fracture	rs17151854	8	T	G	0.0129893	0.000786445	0.00207434	0.000860677	3.80E-10	0.36	8.48E-05	39.21133337
Sleeplessness	Fracture	rs4831364	8	C	T	-0.0135102	0.00258089	0.00260316	0.00108009	2.10E-07	0.017	5.83E-05	26.93530092
Sleeplessness	Fracture	rs11790060	9	C	T	-0.0103391	-0.000604394	0.00157854	0.000654824	5.80E-11	0.36	9.28E-05	42.89970335

Sleeplessness	Fracture	rs72704320	9	C	G	0.0103811	0.000580302	0.0019475	0.000807862	9.80E-08	0.47	6.15E-05	28.41396338
Sleeplessness	Fracture	rs17709610	10	G	A	-0.0099161	-0.000591342	0.00162078	0.000672297	9.50E-10	0.38	8.10E-05	37.43119202
Sleeplessness	Fracture	rs224032	10	A	G	0.00839066	0.000348666	0.00149108	0.000618547	1.80E-08	0.57	6.85E-05	31.66579213
Sleeplessness	Fracture	rs2297787	10	A	T	-0.0178001	-0.00168213	0.00274996	0.0011407	9.60E-11	0.14	9.06E-05	41.89788793
Sleeplessness	Fracture	rs4748580	10	T	C	0.00796249	0.00111064	0.00154673	0.000641588	2.60E-07	0.0830004	5.73E-05	26.50139783
Sleeplessness	Fracture	rs10838708	11	A	G	-0.00947656	-0.00117991	0.00150271	0.000623336	2.90E-10	0.0580003	8.60E-05	39.76958694
Sleeplessness	Fracture	rs518143	11	G	A	-0.00789994	0.000634955	0.00155741	0.000646029	3.90E-07	0.33	5.56E-05	25.73011148
Sleeplessness	Fracture	rs533757	11	C	T	0.00764807	-0.000125338	0.00149524	0.000620217	3.10E-07	0.84	5.66E-05	26.16265988
Sleeplessness	Fracture	rs544566	11	C	T	-0.008001	0.000259769	0.00149998	0.000622252	9.60E-08	0.68	6.15E-05	28.45231472
Sleeplessness	Fracture	rs6486359	11	C	T	-0.00777985	-0.00170853	0.00150217	0.00062312	2.20E-07	0.0061	5.80E-05	26.82281032
Sleeplessness	Fracture	rs72924721	11	T	C	0.016478	0.000971778	0.00288103	0.00119518	1.10E-08	0.42	7.07E-05	32.71247742
Sleeplessness	Fracture	rs324017	12	C	A	-0.00988248	9.31E-05	0.00163145	0.000676775	1.40E-09	0.89	7.94E-05	36.69309554
Sleeplessness	Fracture	rs4767643	12	C	G	-0.00762513	-0.000733158	0.00150129	0.000622735	3.80E-07	0.24	5.58E-05	25.79676937
Sleeplessness	Fracture	rs68094047	12	T	C	0.0103356	0.0015703	0.0017167	0.000712198	1.70E-09	0.0269998	7.84E-05	36.24787673
Sleeplessness	Fracture	rs931221	12	A	T	0.0106361	0.000851829	0.00175313	0.000727249	1.30E-09	0.24	7.96E-05	36.80752203
Sleeplessness	Fracture	rs1547630	13	A	G	0.00910814	0.000776899	0.00156447	0.000648846	5.80E-09	0.23	7.33E-05	33.89416316
Sleeplessness	Fracture	rs2451437	13	T	A	-0.00790356	-0.000202122	0.00152888	0.000634156	2.30E-07	0.75	5.78E-05	26.72383061
Sleeplessness	Fracture	rs6561715	13	A	T	-0.011623	-0.000290243	0.00154234	0.000639703	4.80E-14	0.649999	0.000123	56.79057052
Sleeplessness	Fracture	rs9570080	13	C	T	-0.0106379	-0.000822909	0.00157858	0.000654693	1.60E-11	0.21	9.82E-05	45.41283501
Sleeplessness	Fracture	rs9576155	13	A	G	0.0081654	0.000197199	0.00156892	0.000650765	1.90E-07	0.760001	5.86E-05	27.0865274
Sleeplessness	Fracture	rs11628001	14	A	C	-0.00885303	-0.000364096	0.00169038	0.000701115	1.60E-07	0.6	5.93E-05	27.4293288
Sleeplessness	Fracture	rs11635495	15	C	T	0.00937342	0.0013056	0.00148509	0.000615954	2.80E-10	0.0340001	8.62E-05	39.83736519
Sleeplessness	Fracture	rs176644	15	T	G	0.00805211	0.0012084	0.0015188	0.000629835	1.10E-07	0.0549997	6.08E-05	28.10724125
Sleeplessness	Fracture	rs4886860	15	C	G	-0.011796	-0.00145661	0.00175566	0.000728183	1.80E-11	0.0449997	9.76E-05	45.1428214
Sleeplessness	Fracture	rs872369	15	G	A	-0.00781943	-0.000362038	0.00150709	0.000625035	2.10E-07	0.56	5.82E-05	26.9197992
Sleeplessness	Fracture	rs1125988	16	G	C	-0.00781557	-0.00129534	0.00148983	0.000618006	1.60E-07	0.0359998	5.95E-05	27.51996543

Sleeplessness	Fracture	rs17139246	16	C	T	0.00786398	-0.000262082	0.00153586	0.000637127	3.10E-07	0.68	5.67E-05	26.21691229
Sleeplessness	Fracture	rs2045458	16	T	A	0.00856982	-0.000529946	0.00161985	0.00067182	1.20E-07	0.43	6.05E-05	27.9894136
Sleeplessness	Fracture	rs2062113	16	C	T	-0.00961678	-0.000405599	0.00150275	0.000623298	1.60E-10	0.52	8.86E-05	40.95301577
Sleeplessness	Fracture	rs35198836	16	T	C	0.00882425	-0.000443227	0.00165213	0.000685325	9.20E-08	0.52	6.17E-05	28.52772718
Sleeplessness	Fracture	rs8074498	17	A	T	0.00810568	0.00137719	0.00151731	0.000629334	9.20E-08	0.0290001	6.17E-05	28.53844257
Sleeplessness	Fracture	rs9894577	17	A	G	0.0132051	-0.00116066	0.00159687	0.00066231	1.30E-16	0.08	0.000148	68.38238831
Sleeplessness	Fracture	rs9906181	17	G	A	-0.00915026	-0.000526971	0.00163869	0.000679701	2.40E-08	0.44	6.74E-05	31.17979547
Sleeplessness	Fracture	rs11152363	18	A	G	0.0156393	0.000937919	0.00192518	0.00079837	4.50E-16	0.24	0.000143	65.99209845
Sleeplessness	Fracture	rs2110119	19	A	G	-0.00816352	-0.000613599	0.0016033	0.000664898	3.50E-07	0.36	5.61E-05	25.92539231
Sleeplessness	Fracture	rs56330606	19	G	A	0.00930932	0.00130992	0.00153044	0.000634719	1.20E-09	0.0389996	8.00E-05	37.00013444
Sleeplessness	Fracture	rs13040828	20	T	A	-0.00772419	0.00138048	0.00152564	0.000632742	4.10E-07	0.0290001	5.54E-05	25.63313723
Sleeplessness	Fracture	rs6119267	20	G	C	0.00846101	0.0016892	0.00160721	0.000666544	1.40E-07	0.0109999	5.99E-05	27.71399695
Sleeplessness	Fracture	rs12484368	22	T	C	-0.0316884	-0.00287631	0.00607756	0.00252015	1.80E-07	0.25	5.88E-05	27.18579971
Osteoporosis	Fracture	rs2566755	1	C	T	-0.0020807	-0.00153076	0.00036342	0.000743615	1.03E-08	0.04	9.72E-05	32.77942161
Osteoporosis	Fracture	rs28402693	3	T	C	0.00226354	0.00104075	0.000449257	0.000923632	4.70E-07	0.26	7.53E-05	25.38555376
Osteoporosis	Fracture	rs7683315	4	A	T	0.00206293	0.000652743	0.000318431	0.000651355	9.28E-11	0.32	0.000124	41.9699355
Osteoporosis	Fracture	rs74777717	7	G	C	0.00301364	0.00211239	0.000578604	0.0011951	1.91E-07	0.0769999	8.05E-05	27.12813137
BMI	Stroke	rs11165643	1	C	T	-0.0221	0.0052	0.003	0.0093	1.43E-13	0.5736	0.000229	54.26777778
BMI	Stroke	rs17381664	1	C	T	0.0201	0.0235	0.0031	0.0096	4.57E-11	0.0142298	0.000178	42.04058273
BMI	Stroke	rs2820292	1	A	C	-0.0181	-0.0145	0.0029	0.0092	5.45E-10	0.1141	0.000164	38.9548157
BMI	Stroke	rs543874	1	G	A	0.0497	-8.00E-04	0.0037	0.0116	2.29E-40	0.9442	0.000761	180.4302411
BMI	Stroke	rs657452	1	A	G	0.0227	0.0021	0.0031	0.0095	2.12E-13	0.8275	0.000226	53.6201873
BMI	Stroke	rs7531118	1	T	C	-0.0331	0.0077	0.003	0.0093	1.88E-28	0.4082	0.000514	121.7344444
BMI	Stroke	rs7550711	1	T	C	0.0659	-0.0125	0.0087	0.0286	5.06E-14	0.661899	0.000242	57.37627163
BMI	Stroke	rs977747	1	T	G	0.0168	0.008	0.003	0.0093	2.18E-08	0.3938	0.000132	31.36
BMI	Stroke	rs1016287	2	T	C	0.0228	0.0205	0.0033	0.0102	4.36E-12	0.0455596	0.000202	47.73553719

BMI	Stroke	rs10182181	2	A	G	-0.0309	-0.005	0.0029	0.0095	8.07E-26	0.5967	0.000479	113.5326992
BMI	Stroke	rs10929925	2	C	A	0.0157	0.0158	0.0029	0.0092	9.43E-08	0.08687	0.000124	29.30915577
BMI	Stroke	rs12986742	2	C	T	0.0207	-0.005	0.0036	0.0091	8.92E-09	0.586001	0.00014	33.0625
BMI	Stroke	rs13021737	2	A	G	-0.0604	-0.006	0.0039	0.0122	5.44E-54	0.6222	0.001012	239.8527285
BMI	Stroke	rs1460676	2	T	C	-0.0209	-0.0172	0.0038	0.0124	4.98E-08	0.1677	0.000128	30.25
BMI	Stroke	rs1528435	2	T	C	0.0175	0.0038	0.003	0.0093	4.77E-09	0.687	0.000144	34.02777778
BMI	Stroke	rs1554622	2	T	G	-0.0158	-0.0159	0.0029	0.009	5.94E-08	0.0782997	0.000125	29.68370987
BMI	Stroke	rs17203016	2	G	A	0.0211	0.0023	0.0038	0.0114	3.41E-08	0.8416	0.00013	30.83171745
BMI	Stroke	rs1979755	2	G	C	-0.0187	-0.0103	0.0037	0.0097	4.33E-07	0.2915	0.000108	25.54346238
BMI	Stroke	rs2890652	2	T	C	-0.0279	-0.0034	0.0049	0.0122	1.24E-08	0.7802	0.000137	32.42024157
BMI	Stroke	rs6713510	2	A	G	0.0164	-5.00E-04	0.0029	0.0094	1.97E-08	0.9602	0.000135	31.98097503
BMI	Stroke	rs7599312	2	G	A	0.0214	-0.0093	0.0033	0.0103	4.73E-11	0.3676	0.000178	42.05325987
BMI	Stroke	rs972540	2	A	G	-0.0169	-0.0038	0.0033	0.0103	3.95E-07	0.7097	0.000111	26.22681359
BMI	Stroke	rs13078960	3	T	G	-0.029	-0.0055	0.0038	0.0114	1.42E-14	0.6278	0.000246	58.24099723
BMI	Stroke	rs1516725	3	T	C	-0.0448	-0.0051	0.0044	0.0135	1.39E-24	0.7059	0.000438	103.6694215
BMI	Stroke	rs16851483	3	G	T	-0.0478	-0.0102	0.0075	0.0184	1.85E-10	0.5784	0.000172	40.61937778
BMI	Stroke	rs2365389	3	C	T	0.0195	0.0115	0.003	0.0093	1.35E-10	0.2187	0.000178	42.25
BMI	Stroke	rs3849570	3	A	C	0.0183	0.0088	0.0033	0.0096	1.93E-08	0.3612	0.00013	30.75206612
BMI	Stroke	rs6804842	3	A	G	-0.0183	0.0015	0.003	0.0093	8.02E-10	0.8727	0.000157	37.21
BMI	Stroke	rs7613875	3	A	C	0.0156	0.0205	0.0031	0.0098	4.55E-07	0.0373499	0.000107	25.32362123
BMI	Stroke	rs7649970	3	T	C	0.0231	2.00E-04	0.0043	0.0139	1.01E-07	0.987	0.000122	28.85938345
BMI	Stroke	rs876424	3	G	T	0.0188	0.0086	0.0037	0.0094	3.75E-07	0.3608	0.000109	25.81738495
BMI	Stroke	rs11727676	4	C	T	-0.0365	0.0147	0.0063	0.0177	6.25E-09	0.4047	0.000142	33.56638952
BMI	Stroke	rs13107325	4	C	T	-0.0472	0.001	0.0066	0.0196	1.06E-12	0.9589	0.000216	51.14416896
BMI	Stroke	rs13130484	4	C	T	-0.0398	-0.0022	0.003	0.0093	8.01E-41	0.8121	0.000743	176.0044444
BMI	Stroke	rs17001654	4	C	G	-0.0304	0.002	0.0052	0.0129	5.03E-09	0.876	0.000144	34.17751479

BMI	Stroke	rs11951673	5	T	C	-0.0154	0.0236	0.003	0.0093	2.75E-07	0.01136	0.000111	26.35111111
BMI	Stroke	rs2112347	5	G	T	-0.0254	-0.0213	0.003	0.01	1.96E-17	0.0342997	0.000303	71.68444444
BMI	Stroke	rs6864049	5	A	G	-0.0154	-0.0165	0.003	0.0092	2.12E-07	0.0714299	0.000111	26.35111111
BMI	Stroke	rs7715256	5	G	T	0.0168	0.0109	0.0029	0.009	8.85E-09	0.2291	0.000142	33.56004756
BMI	Stroke	rs13191362	6	A	G	0.0285	-0.0205	0.0047	0.014	1.09E-09	0.1427	0.000155	36.77003169
BMI	Stroke	rs13201877	6	A	G	-0.0236	-0.0162	0.0043	0.0133	4.29E-08	0.222	0.000127	30.12222823
BMI	Stroke	rs2228213	6	G	A	0.0156	0.0158	0.0031	0.0097	3.27E-07	0.1015	0.000107	25.32362123
BMI	Stroke	rs3800229	6	T	G	0.0175	-0.013	0.0032	0.0102	4.95E-08	0.2004	0.000126	29.90722656
BMI	Stroke	rs539958	6	C	T	-0.0149	-0.0066	0.0029	0.0088	3.52E-07	0.4545	0.000111	26.39833532
BMI	Stroke	rs6457796	6	T	C	-0.0209	-0.0175	0.0033	0.0103	2.54E-10	0.08908	0.000169	40.11111111
BMI	Stroke	rs9374842	6	T	C	0.0196	0.0058	0.0034	0.0103	7.20E-09	0.575999	0.00014	33.23183391
BMI	Stroke	rs943005	6	T	C	0.0444	0.0174	0.0038	0.0121	4.52E-31	0.1514	0.000576	136.5207756
BMI	Stroke	rs10499694	7	G	A	-0.0147	0.0053	0.0029	0.0091	4.72E-07	0.5559	0.000109	25.69441141
BMI	Stroke	rs2245368	7	T	C	-0.0288	0.0103	0.0053	0.0147	7.01E-08	0.483499	0.000125	29.52794589
BMI	Stroke	rs9641123	7	G	C	-0.0193	0.0075	0.0037	0.0094	1.83E-07	0.4251	0.000115	27.20891161
BMI	Stroke	rs2033732	8	C	T	0.0176	0.008	0.0034	0.0107	2.26E-07	0.4523	0.000113	26.79584775
BMI	Stroke	rs2060604	8	T	C	0.0203	-0.0167	0.003	0.0091	9.46E-12	0.0673798	0.000193	45.78777778
BMI	Stroke	rs6990042	8	G	T	0.0155	-0.0016	0.0031	0.0102	4.48E-07	0.8772	0.000106	25
BMI	Stroke	rs7844647	8	T	C	0.0171	-0.0027	0.0033	0.0102	2.69E-07	0.7931	0.000113	26.85123967
BMI	Stroke	rs10733682	9	A	G	0.0188	-0.0112	0.003	0.0092	2.46E-10	0.2223	0.000166	39.27111111
BMI	Stroke	rs12352785	9	A	C	0.016	0.0034	0.0032	0.0097	4.87E-07	0.7257	0.000106	25
BMI	Stroke	rs1928295	9	C	T	-0.0182	-0.001	0.0029	0.0091	4.32E-10	0.9097	0.000166	39.38644471
BMI	Stroke	rs2183825	9	C	T	0.0241	-4.00E-04	0.0032	0.0097	2.22E-14	0.9634	0.000239	56.71972656
BMI	Stroke	rs2270204	9	T	G	-0.018	-0.0078	0.0035	0.0109	3.22E-07	0.4729	0.000112	26.44897959
BMI	Stroke	rs4740619	9	T	C	0.017	0.009	0.0029	0.0091	6.36E-09	0.3212	0.000145	34.36385256
BMI	Stroke	rs6477694	9	C	T	0.0169	0.0101	0.003	0.0095	1.71E-08	0.288	0.000134	31.73444444

BMI	Stroke	rs11191343	10	G	A	-0.0258	0.0047	0.0051	0.016	4.86E-07	0.7691	0.000108	25.5916955
BMI	Stroke	rs17094222	10	C	T	0.0249	0.0114	0.0037	0.0113	2.19E-11	0.3147	0.000191	45.28926224
BMI	Stroke	rs7899106	10	A	G	-0.0379	0.0162	0.0067	0.0211	1.27E-08	0.4417	0.000135	31.99844063
BMI	Stroke	rs7903146	10	T	C	-0.0235	0.0287	0.0033	0.0108	1.10E-12	0.00797793	0.000214	50.71166208
BMI	Stroke	rs10840100	11	G	A	0.0206	-0.006	0.003	0.0096	6.67E-12	0.528	0.000199	47.15111111
BMI	Stroke	rs11030104	11	A	G	0.0416	0.0217	0.0037	0.0116	6.66E-30	0.0617106	0.000534	126.4105186
BMI	Stroke	rs12286929	11	G	A	0.0211	0.0053	0.0029	0.0091	5.44E-13	0.5589	0.000224	52.93816885
BMI	Stroke	rs3817334	11	C	T	-0.0256	-0.0144	0.003	0.0094	1.17E-17	0.1263	0.000307	72.81777778
BMI	Stroke	rs11057405	12	A	G	-0.0304	0.0295	0.0053	0.0163	1.22E-08	0.0712492	0.000139	32.8999644
BMI	Stroke	rs11170468	12	C	A	-0.0186	-6.00E-04	0.0035	0.0108	1.01E-07	0.9582	0.000119	28.24163265
BMI	Stroke	rs11611246	12	T	G	0.0202	0.0146	0.0039	0.0116	1.70E-07	0.2071	0.000113	26.82708744
BMI	Stroke	rs2579106	12	C	T	0.0212	-0.0069	0.0041	0.0106	2.33E-07	0.5145	0.000113	26.73646639
BMI	Stroke	rs7138803	12	G	A	-0.032	-0.0023	0.003	0.0095	5.12E-26	0.806	0.00048	113.7777778
BMI	Stroke	rs12429545	13	G	A	-0.0324	-0.0286	0.0044	0.0138	3.15E-13	0.0377503	0.000229	54.2231405
BMI	Stroke	rs1441264	13	A	G	0.0172	-0.0092	0.0031	0.0095	2.96E-08	0.3342	0.00013	30.78459938
BMI	Stroke	rs9540493	13	G	A	-0.0182	-0.0109	0.0031	0.0094	3.95E-09	0.2442	0.000146	34.46826223
BMI	Stroke	rs9579083	13	G	C	-0.0295	0.0108	0.0046	0.0116	1.43E-10	0.3494	0.000174	41.12712665
BMI	Stroke	rs10132280	14	A	C	-0.0221	0.0171	0.0033	0.01	1.40E-11	0.0888198	0.000189	44.84940312
BMI	Stroke	rs12887636	14	G	T	-0.0169	-0.001	0.0032	0.0097	1.35E-07	0.9189	0.000118	27.89160156
BMI	Stroke	rs3783890	14	C	T	-0.0198	-0.0149	0.0038	0.0119	1.40E-07	0.2081	0.000115	27.14958449
BMI	Stroke	rs709400	14	A	G	0.016	0.0125	0.003	0.0095	1.38E-07	0.1902	0.00012	28.44444444
BMI	Stroke	rs7144011	14	T	G	0.0274	0.0194	0.0035	0.0112	6.05E-15	0.0827104	0.000259	61.28653061
BMI	Stroke	rs13329567	15	T	C	-0.0307	-0.0196	0.0035	0.0111	1.53E-18	0.0766196	0.000325	76.93795918
BMI	Stroke	rs3736485	15	A	G	0.016	-0.0104	0.0029	0.0092	4.52E-08	0.2587	0.000129	30.43995244
BMI	Stroke	rs4984406	15	T	C	0.0156	-0.0033	0.0029	0.0091	7.57E-08	0.719701	0.000122	28.93697979
BMI	Stroke	rs12448257	16	G	A	-0.0246	2.00E-04	0.0037	0.0114	3.90E-11	0.9895	0.000187	44.20452885

BMI	Stroke	rs1421085	16	C	T	0.0803	0.0027	0.003	0.0095	2.17E-158	0.774801	0.003017	716.4544444
BMI	Stroke	rs2303223	16	A	G	-0.019	0.0124	0.0031	0.0094	5.13E-10	0.1854	0.000159	37.56503642
BMI	Stroke	rs2307022	16	G	A	-0.0159	-0.0035	0.0031	0.0096	4.32E-07	0.7149	0.000111	26.3069719
BMI	Stroke	rs3888190	16	A	C	0.0311	-0.0035	0.003	0.0092	3.45E-25	0.706601	0.000454	107.4677778
BMI	Stroke	rs879620	16	C	T	-0.0244	-0.0115	0.0039	0.0097	3.94E-10	0.2331	0.000165	39.1426693
BMI	Stroke	rs9926784	16	T	C	0.0249	0.0073	0.0038	0.0121	8.55E-11	0.5446	0.000181	42.93698061
BMI	Stroke	rs1000940	17	G	A	0.0184	-0.0201	0.0033	0.0099	1.81E-08	0.0431003	0.000131	31.08907254
BMI	Stroke	rs12150665	17	T	C	0.016	-0.022	0.003	0.0092	8.06E-08	0.0166702	0.00012	28.44444444
BMI	Stroke	rs12940622	17	A	G	-0.0183	-7.00E-04	0.0029	0.0091	3.64E-10	0.9382	0.000168	39.82045184
BMI	Stroke	rs4986044	17	C	T	0.0155	0.0205	0.003	0.0097	1.79E-07	0.0346298	0.000113	26.69444444
BMI	Stroke	rs6504108	17	C	T	0.0172	-8.00E-04	0.0032	0.0097	1.12E-07	0.9373	0.000122	28.890625
BMI	Stroke	rs7223966	17	G	A	0.0171	0.0159	0.0033	0.0101	1.38E-07	0.1178	0.000113	26.85123967
BMI	Stroke	rs17066856	18	C	T	-0.0371	0.0315	0.005	0.0157	2.00E-13	0.0449697	0.000232	55.0564
BMI	Stroke	rs6567160	18	C	T	0.0562	-6.00E-04	0.0035	0.0106	6.68E-59	0.9543	0.001088	257.8318367
BMI	Stroke	rs7239883	18	G	A	0.0152	0.0062	0.003	0.0094	3.14E-07	0.5078	0.000108	25.67111111
BMI	Stroke	rs11672550	19	T	C	0.0211	-0.0051	0.004	0.0099	1.33E-07	0.6101	0.000118	27.825625
BMI	Stroke	rs11672660	19	C	T	0.0339	-0.0108	0.0038	0.0113	7.91E-19	0.3429	0.000336	79.58518006
BMI	Stroke	rs11880870	19	G	A	-0.0176	-0.0169	0.0034	0.0091	2.39E-07	0.0638793	0.000113	26.79584775
BMI	Stroke	rs14810	19	C	G	-0.0183	-0.0014	0.0033	0.0098	1.92E-08	0.887	0.00013	30.75206612
BMI	Stroke	rs17724992	19	A	G	0.0196	0.0262	0.0034	0.0104	7.79E-09	0.012	0.00014	33.23183391
BMI	Stroke	rs405509	19	T	G	-0.0166	0.0079	0.0032	0.0102	2.65E-07	0.4409	0.000114	26.91015625
BMI	Stroke	rs9304665	19	A	T	0.0243	-0.0102	0.0043	0.0109	1.59E-08	0.3505	0.000135	31.93564089
BMI	Stroke	rs6091540	20	C	T	0.0185	0.0112	0.0033	0.0101	2.14E-08	0.2697	0.000133	31.42791552
BMI	Stroke	rs8123881	20	G	A	0.0216	0.021	0.0043	0.0135	3.91E-07	0.119	0.000107	25.23309897
BMI	Stroke	rs2836754	21	C	T	0.0169	0.003	0.003	0.0097	1.61E-08	0.760699	0.000134	31.73444444
BMI	Stroke	rs427943	21	C	A	0.0182	-0.0176	0.0035	0.0095	1.59E-07	0.0651298	0.000114	27.04

Hip circumference	Stroke	rs10493979	1	G	T	-0.0139097	-0.007	0.00262114	0.0098	1.12E-07	0.4757	8.37E-05	28.16145508
Hip circumference	Stroke	rs10493988	1	G	A	0.0132618	-0.0079	0.002503	0.0094	1.17E-07	0.4029	8.34E-05	28.07263952
Hip circumference	Stroke	rs112646560	1	T	C	0.0208306	0.0016	0.00294004	0.011	1.39E-12	0.8839	0.000149	50.19923289
Hip circumference	Stroke	rs1127100	1	C	T	0.0149329	0.0032	0.00252469	0.0097	3.33E-09	0.741899	0.000104	34.98421988
Hip circumference	Stroke	rs11584359	1	T	C	-0.0211394	-0.0039	0.0031669	0.0126	2.47E-11	0.7579	0.000132	44.55706881
Hip circumference	Stroke	rs11803990	1	G	C	0.0252396	-0.022	0.00438591	0.0171	8.69E-09	0.1971	9.84E-05	33.11658186
Hip circumference	Stroke	rs12096864	1	C	T	0.0219612	0.0155	0.00376456	0.0138	5.43E-09	0.2632	0.000101	34.03170339
Hip circumference	Stroke	rs12097230	1	A	G	-0.0128953	-0.0061	0.00242728	0.0092	1.08E-07	0.5092	8.38E-05	28.22429777
Hip circumference	Stroke	rs12140153	1	T	G	-0.0248568	-0.0119	0.00417296	0.017	2.58E-09	0.4833	0.000105	35.48150169
Hip circumference	Stroke	rs1229128	1	A	G	0.0158537	-0.0126	0.00304025	0.0118	1.84E-07	0.286	8.08E-05	27.19209553
Hip circumference	Stroke	rs12561919	1	T	C	0.0200114	-0.0122	0.00338817	0.0132	3.50E-09	0.3542	0.000104	34.88386206
Hip circumference	Stroke	rs16825336	1	A	G	0.0213158	-0.0113	0.00414967	0.016	2.80E-07	0.4773	7.84E-05	26.38615755
Hip circumference	Stroke	rs17024393	1	C	T	0.0568069	0.002	0.0076056	0.0275	8.09E-14	0.9408	0.000166	55.78728401
Hip circumference	Stroke	rs2494196	1	A	C	0.0320476	0.0145	0.00265572	0.01	1.60E-33	0.1482	0.000432	145.6218199
Hip circumference	Stroke	rs2678204	1	G	T	0.0216541	0.0173	0.00253869	0.0098	1.47E-17	0.0779992	0.000216	72.75467943
Hip circumference	Stroke	rs2761185	1	A	G	-0.0318384	0.0137	0.00611646	0.0222	1.94E-07	0.5359	8.05E-05	27.0958132
Hip circumference	Stroke	rs2802774	1	A	C	0.016994	-0.0192	0.00245316	0.0117	4.29E-12	0.0993002	0.000143	47.98875451
Hip circumference	Stroke	rs2815753	1	A	G	0.0218333	-0.013	0.00245234	0.0092	5.46E-19	0.1558	0.000235	79.26425837
Hip circumference	Stroke	rs33955687	1	A	C	-0.0160724	0.0147	0.00270852	0.01	2.96E-09	0.1434	0.000105	35.21254144
Hip circumference	Stroke	rs34517439	1	A	C	0.0429378	0.024	0.00368662	0.0151	2.41E-31	0.1131	0.000403	135.6509463
Hip circumference	Stroke	rs3845344	1	T	C	0.0146464	-0.0136	0.00246158	0.0095	2.68E-09	0.1492	0.000105	35.40249517
Hip circumference	Stroke	rs4471313	1	T	G	-0.0170096	0.0101	0.00272413	0.0101	4.27E-10	0.3185	0.000116	38.9881424
Hip circumference	Stroke	rs4660586	1	T	C	-0.019614	-0.0093	0.00274699	0.0103	9.34E-13	0.3644	0.000151	50.98215453
Hip circumference	Stroke	rs4908677	1	T	C	0.0132201	-0.004	0.00242907	0.0093	5.26E-08	0.667	8.80E-05	29.62029775
Hip circumference	Stroke	rs543874	1	G	A	0.0442781	-8.00E-04	0.00296152	0.0116	1.59E-50	0.9442	0.000664	223.536586
Hip circumference	Stroke	rs588660	1	A	G	0.0188173	-0.0024	0.00244358	0.0093	1.36E-14	0.792399	0.000176	59.30092857

Hip circumference	Stroke	rs60226453	1	T	C	0.0177113	-0.0083	0.00316301	0.0156	2.15E-08	0.595701	9.31E-05	31.35449055
Hip circumference	Stroke	rs6669341	1	G	A	-0.0146303	-0.0046	0.00244057	0.0092	2.04E-09	0.618399	0.000107	35.93551938
Hip circumference	Stroke	rs7516554	1	T	C	0.0155427	-0.0054	0.00245713	0.0094	2.53E-10	0.5681	0.000119	40.01258964
Hip circumference	Stroke	rs7548408	1	C	T	0.0150333	0.0288	0.00243163	0.0092	6.32E-10	0.00177101	0.000114	38.22202241
Hip circumference	Stroke	rs76798800	1	T	G	0.0254141	0.0161	0.0027214	0.0103	9.82E-21	0.1203	0.000259	87.20968303
Hip circumference	Stroke	rs815335	1	T	C	0.0184289	-0.0122	0.00250163	0.0097	1.75E-13	0.2064	0.000161	54.26910685
Hip circumference	Stroke	rs9424466	1	C	A	0.0138768	0.0089	0.00269806	0.0104	2.70E-07	0.3944	7.86E-05	26.45303164
Hip circumference	Stroke	rs1014291	2	T	G	-0.0160298	-6.00E-04	0.00243785	0.0094	4.86E-11	0.9496	0.000128	43.23567537
Hip circumference	Stroke	rs10210468	2	C	T	-0.0150017	-0.0018	0.00244268	0.0093	8.19E-10	0.8463	0.000112	37.7179256
Hip circumference	Stroke	rs1118151	2	G	T	0.016606	0.0038	0.00269097	0.0101	6.79E-10	0.7103	0.000113	38.08135167
Hip circumference	Stroke	rs11688707	2	A	G	0.0130416	-0.0068	0.00252167	0.01	2.32E-07	0.4938	7.95E-05	26.74762638
Hip circumference	Stroke	rs12467963	2	T	A	-0.013934	0.0145	0.00247214	0.0095	1.74E-08	0.1268	9.44E-05	31.76914223
Hip circumference	Stroke	rs12475388	2	A	G	-0.0134418	-0.0086	0.00241781	0.0098	2.71E-08	0.3791	9.18E-05	30.90797267
Hip circumference	Stroke	rs12619178	2	T	C	-0.0158059	-2.00E-04	0.00245114	0.0093	1.13E-10	0.982	0.000124	41.58169853
Hip circumference	Stroke	rs12714415	2	C	T	-0.0490852	-0.0045	0.00331126	0.0122	1.07E-49	0.7115	0.000652	219.7427544
Hip circumference	Stroke	rs13389219	2	T	C	0.0263086	-0.014	0.0024607	0.0094	1.13E-26	0.1393	0.000339	114.3083978
Hip circumference	Stroke	rs13410783	2	G	A	0.0146689	-0.0018	0.00249694	0.0101	4.24E-09	0.8575	0.000103	34.51269573
Hip circumference	Stroke	rs1528450	2	C	T	0.0172654	0.0174	0.0024864	0.0094	3.82E-12	0.0639499	0.000143	48.21823337
Hip circumference	Stroke	rs2193618	2	C	T	0.0133907	-0.0064	0.00247204	0.0093	6.07E-08	0.492	8.72E-05	29.34239595
Hip circumference	Stroke	rs2244786	2	A	G	0.0139551	0.0175	0.00251229	0.0095	2.78E-08	0.0661394	9.17E-05	30.85505796
Hip circumference	Stroke	rs2693823	2	A	T	-0.0150177	-0.0173	0.00276473	0.0109	5.58E-08	0.1108	8.76E-05	29.50539267
Hip circumference	Stroke	rs2861690	2	G	C	-0.0171381	-0.0107	0.00246592	0.0094	3.66E-12	0.2545	0.000143	48.30225198
Hip circumference	Stroke	rs34168749	2	T	C	-0.0125537	-0.0048	0.0024143	0.0092	2.00E-07	0.5977	8.03E-05	27.03715701
Hip circumference	Stroke	rs35882248	2	T	C	0.0170391	0.0079	0.00259225	0.0097	4.94E-11	0.4194	0.000128	43.20554992
Hip circumference	Stroke	rs4430895	2	T	C	0.0241932	0.0035	0.00240643	0.0096	8.93E-24	0.716199	0.0003	101.0741657
Hip circumference	Stroke	rs4482463	2	A	C	-0.029946	-0.0425	0.00455065	0.0175	4.69E-11	0.01495	0.000129	43.30427499

Hip circumference	Stroke	rs4670612	2	G	A	0.0163808	0.0108	0.00253152	0.0102	9.77E-11	0.2883	0.000124	41.87043666
Hip circumference	Stroke	rs4832298	2	T	C	-0.013755	0.0013	0.00259071	0.0099	1.10E-07	0.8948	8.37E-05	28.18925424
Hip circumference	Stroke	rs58584712	2	A	G	0.0198104	-0.0022	0.00295089	0.015	1.90E-11	0.8818	0.000134	45.06926214
Hip circumference	Stroke	rs62166769	2	A	T	0.0127581	-0.0057	0.00249432	0.0095	3.14E-07	0.5499	7.77E-05	26.16180268
Hip circumference	Stroke	rs62183012	2	C	T	-0.0134016	0.0017	0.00265696	0.01	4.56E-07	0.8639	7.56E-05	25.44153263
Hip circumference	Stroke	rs6437277	2	G	A	-0.0150561	-0.0183	0.00286809	0.0141	1.53E-07	0.1947	8.19E-05	27.55747554
Hip circumference	Stroke	rs6707036	2	G	A	-0.0146292	-0.0269	0.00255395	0.0098	1.02E-08	0.00591902	9.75E-05	32.81076629
Hip circumference	Stroke	rs6739755	2	G	A	-0.0152363	-0.0171	0.00246322	0.0094	6.20E-10	0.06952	0.000114	38.26067487
Hip circumference	Stroke	rs6747657	2	A	G	0.0140974	0.0052	0.00266546	0.01	1.23E-07	0.6016	8.31E-05	27.9726561
Hip circumference	Stroke	rs72917533	2	C	T	-0.0164346	7.00E-04	0.00309747	0.012	1.12E-07	0.9515	8.36E-05	28.15166326
Hip circumference	Stroke	rs75543804	2	T	G	-0.040796	-0.0143	0.00679915	0.0283	1.97E-09	0.6131	0.000107	36.00194145
Hip circumference	Stroke	rs77165542	2	T	C	-0.0864376	0.0171	0.00657899	0.0326	2.03E-39	0.599601	0.000513	172.6183445
Hip circumference	Stroke	rs968379	2	T	C	-0.0206395	-0.0045	0.00286037	0.0111	5.38E-13	0.6819	0.000155	52.06596419
Hip circumference	Stroke	rs11915747	3	G	C	-0.0187962	-0.0061	0.00251523	0.0095	7.86E-14	0.5209	0.000166	55.84505284
Hip circumference	Stroke	rs13085031	3	T	C	0.0131865	-0.0045	0.00246501	0.0093	8.83E-08	0.6259	8.50E-05	28.61684211
Hip circumference	Stroke	rs1406779	3	T	C	0.0201178	-0.0024	0.00258241	0.01	6.70E-15	0.8081	0.00018	60.68908407
Hip circumference	Stroke	rs1727901	3	T	C	0.0188821	0.0174	0.00272522	0.0107	4.26E-12	0.1049	0.000143	48.00621343
Hip circumference	Stroke	rs2034768	3	G	A	-0.016569	-0.0031	0.002406	0.0092	5.73E-12	0.7383	0.000141	47.42434593
Hip circumference	Stroke	rs2270894	3	G	C	-0.0199916	-0.0074	0.00309373	0.0139	1.03E-10	0.5956	0.000124	41.75709618
Hip circumference	Stroke	rs2371767	3	C	G	0.0198003	-0.034	0.00270203	0.0102	2.34E-13	0.000873394	0.00016	53.69863024
Hip circumference	Stroke	rs34373881	3	A	G	-0.0149443	-0.0055	0.00268972	0.0103	2.76E-08	0.5909	9.17E-05	30.87002704
Hip circumference	Stroke	rs35779991	3	C	T	0.0143334	0.0012	0.00242195	0.0092	3.26E-09	0.8946	0.000104	35.02418962
Hip circumference	Stroke	rs4017425	3	T	C	-0.0138473	0.0146	0.00241466	0.0092	9.78E-09	0.1102	9.77E-05	32.88654326
Hip circumference	Stroke	rs55932154	3	G	A	-0.0236291	0.0138	0.0037729	0.0149	3.78E-10	0.3553	0.000117	39.22326766
Hip circumference	Stroke	rs62243489	3	G	T	-0.0153338	-0.0021	0.00276351	0.0105	2.88E-08	0.8445	9.15E-05	30.78771856
Hip circumference	Stroke	rs62246314	3	A	G	0.0247126	0.0202	0.00398491	0.015	5.60E-10	0.179	0.000114	38.45916448

Hip circumference	Stroke	rs6789488	3	C	T	0.0163656	-0.002	0.00278041	0.0106	3.96E-09	0.8477	0.000103	34.64544793
Hip circumference	Stroke	rs6810023	3	A	G	0.0170797	-0.0143	0.00335675	0.013	3.62E-07	0.2705	7.69E-05	25.88942954
Hip circumference	Stroke	rs724016	3	G	A	0.0264891	0.0166	0.0024177	0.0093	6.26E-28	0.0759591	0.000357	120.0409945
Hip circumference	Stroke	rs73175572	3	G	A	0.0264617	-0.0315	0.00385671	0.0148	6.84E-12	0.0332698	0.00014	47.07621256
Hip circumference	Stroke	rs7426945	3	G	A	0.0156387	0.0134	0.0024133	0.0094	9.17E-11	0.1536	0.000125	41.99317109
Hip circumference	Stroke	rs7610647	3	G	A	-0.0139905	-0.0067	0.00265614	0.0102	1.39E-07	0.5097	8.24E-05	27.74371071
Hip circumference	Stroke	rs79375047	3	T	C	0.0158983	0.0042	0.00309756	0.012	2.86E-07	0.723801	7.83E-05	26.34279916
Hip circumference	Stroke	rs79597869	3	G	A	0.0122375	-0.0017	0.00240601	0.0093	3.65E-07	0.8557	7.69E-05	25.86965003
Hip circumference	Stroke	rs8192675	3	C	T	0.0172691	-7.00E-04	0.00265297	0.0099	7.56E-11	0.9418	0.000126	42.37158703
Hip circumference	Stroke	rs838204	3	G	A	0.013251	0.0113	0.00254985	0.0097	2.03E-07	0.2441	8.02E-05	27.00648371
Hip circumference	Stroke	rs869400	3	G	T	0.0196495	0.0171	0.00311102	0.0117	2.69E-10	0.1446	0.000119	39.89306471
Hip circumference	Stroke	rs9808900	3	T	G	0.0269504	0.0095	0.00301663	0.0116	4.13E-19	0.4098	0.000237	79.81533473
Hip circumference	Stroke	rs9814633	3	A	G	0.0147487	-0.0057	0.00253421	0.0096	5.90E-09	0.554799	0.000101	33.87055266
Hip circumference	Stroke	rs9843653	3	C	T	0.0205097	0.0141	0.00240714	0.0092	1.60E-17	0.1257	0.000216	72.59653899
Hip circumference	Stroke	rs10938397	4	G	A	0.0231136	0.0018	0.002432	0.0095	2.03E-21	0.8532	0.000268	90.32501558
Hip circumference	Stroke	rs1296328	4	C	A	-0.0155122	-0.0071	0.00243358	0.0097	1.84E-10	0.4661	0.000121	40.6308152
Hip circumference	Stroke	rs13107325	4	T	C	0.042561	-0.001	0.00457779	0.0196	1.45E-20	0.9589	0.000257	86.43943404
Hip circumference	Stroke	rs13151185	4	T	G	-0.0136099	-0.002	0.00241049	0.0093	1.64E-08	0.8267	9.47E-05	31.87859704
Hip circumference	Stroke	rs2102278	4	G	A	0.0158475	0.0328	0.00257945	0.0101	8.07E-10	0.00112401	0.000112	37.74568341
Hip circumference	Stroke	rs2318543	4	G	A	-0.0179454	-0.0084	0.00292494	0.0112	8.51E-10	0.4525	0.000112	37.64197161
Hip circumference	Stroke	rs28418580	4	T	C	-0.0174415	0.0089	0.00242792	0.0093	6.80E-13	0.3398	0.000153	51.60584805
Hip circumference	Stroke	rs34049648	4	A	G	0.0176063	0.0103	0.00254462	0.0096	4.56E-12	0.2865	0.000142	47.87296462
Hip circumference	Stroke	rs34811474	4	A	G	-0.0205406	-0.0155	0.00284677	0.0131	5.39E-13	0.2383	0.000155	52.06207696
Hip circumference	Stroke	rs4240326	4	G	A	-0.0278879	0.0028	0.00241489	0.0092	7.63E-31	0.7613	0.000396	133.3634798
Hip circumference	Stroke	rs6535240	4	G	A	0.0144793	-0.0077	0.00259902	0.0098	2.53E-08	0.4319	9.22E-05	31.03672512
Hip circumference	Stroke	rs66679256	4	T	C	0.015685	0.0066	0.00242434	0.0093	9.83E-11	0.478	0.000124	41.85833909

Hip circumference	Stroke	rs6821305	4	C	A	0.0150058	-0.0012	0.00245839	0.0093	1.04E-09	0.8969	0.000111	37.25776047
Hip circumference	Stroke	rs6837528	4	A	G	-0.0134485	-1.00E-04	0.00266145	0.0104	4.35E-07	0.9917	7.59E-05	25.53354243
Hip circumference	Stroke	rs6840236	4	C	T	0.0166995	0.0071	0.00241801	0.0092	4.98E-12	0.4425	0.000142	47.69696562
Hip circumference	Stroke	rs73213484	4	T	A	-0.0195843	0.0046	0.00347756	0.0127	1.79E-08	0.7168	9.42E-05	31.71515533
Hip circumference	Stroke	rs750090	4	C	T	-0.0169135	0.0145	0.00253756	0.0098	2.65E-11	0.1375	0.000132	44.42570576
Hip circumference	Stroke	rs11745618	5	G	A	0.0125425	-0.0033	0.00243148	0.0094	2.49E-07	0.724001	7.90E-05	26.60889355
Hip circumference	Stroke	rs12519997	5	A	G	-0.014272	-0.0123	0.00242205	0.0092	3.81E-09	0.1796	0.000103	34.72189868
Hip circumference	Stroke	rs1428120	5	T	G	-0.0136416	-0.0122	0.0024288	0.0094	1.95E-08	0.1936	9.37E-05	31.54620444
Hip circumference	Stroke	rs1477290	5	C	T	0.0281297	0.011	0.00354094	0.0131	1.96E-15	0.3996	0.000187	63.10925673
Hip circumference	Stroke	rs1582931	5	A	G	-0.0196312	-0.0299	0.00242829	0.0092	6.27E-16	0.001191	0.000194	65.35707274
Hip circumference	Stroke	rs185299	5	A	G	0.0144078	-0.0089	0.00271402	0.013	1.11E-07	0.494399	8.37E-05	28.18183417
Hip circumference	Stroke	rs2307111	5	C	T	-0.0282307	-0.018	0.00246445	0.0095	2.24E-30	0.0568905	0.00039	131.2209782
Hip circumference	Stroke	rs252749	5	A	G	-0.0230008	-0.0037	0.00278435	0.0109	1.45E-16	0.732799	0.000203	68.23987626
Hip circumference	Stroke	rs33967909	5	A	G	0.0154853	5.00E-04	0.00293324	0.0108	1.30E-07	0.9602	8.28E-05	27.87045439
Hip circumference	Stroke	rs34629844	5	G	A	0.0219304	0.0101	0.00358667	0.0146	9.70E-10	0.4885	0.000111	37.38610882
Hip circumference	Stroke	rs3811951	5	G	A	0.0146228	0.0114	0.00266992	0.0102	4.33E-08	0.2663	8.91E-05	29.99608551
Hip circumference	Stroke	rs3943933	5	A	T	0.0132294	0.0191	0.00240628	0.0091	3.85E-08	0.0360197	8.98E-05	30.22650771
Hip circumference	Stroke	rs40071	5	C	T	-0.0174246	-0.0334	0.00314153	0.0123	2.92E-08	0.00675694	9.14E-05	30.76402897
Hip circumference	Stroke	rs4073717	5	T	G	-0.0175965	-0.0071	0.00300343	0.0117	4.67E-09	0.544999	0.000102	34.32555428
Hip circumference	Stroke	rs4267859	5	A	C	-0.0130751	0.0175	0.00248944	0.0092	1.50E-07	0.0573601	8.19E-05	27.58587165
Hip circumference	Stroke	rs4866585	5	G	T	0.0134649	0.023	0.0024895	0.0097	6.35E-08	0.0171498	8.69E-05	29.25378084
Hip circumference	Stroke	rs4921301	5	T	C	-0.0156418	-0.0079	0.00298724	0.0114	1.64E-07	0.4913	8.14E-05	27.41783921
Hip circumference	Stroke	rs59738707	5	A	G	-0.0232805	0.0087	0.0036192	0.0134	1.26E-10	0.515	0.000123	41.37704203
Hip circumference	Stroke	rs6867299	5	C	T	0.0205436	-0.0051	0.00250483	0.0096	2.38E-16	0.5962	0.0002	67.26615266
Hip circumference	Stroke	rs7442885	5	G	C	-0.020481	-0.0199	0.00296022	0.0111	4.56E-12	0.0718903	0.000142	47.86899814
Hip circumference	Stroke	rs74473266	5	T	C	-0.0256909	0.0083	0.00511014	0.0198	4.97E-07	0.673101	7.51E-05	25.27510919

Hip circumference	Stroke	rs75949361	5	T	C	0.0436087	0.0332	0.00678949	0.0233	1.34E-10	0.1548	0.000123	41.25456143
Hip circumference	Stroke	rs7703744	5	G	C	0.0143594	-0.0016	0.00271623	0.0103	1.25E-07	0.8795	8.30E-05	27.94727729
Hip circumference	Stroke	rs7714611	5	G	A	-0.0129498	-0.0026	0.00255816	0.0097	4.15E-07	0.787601	7.61E-05	25.62540361
Hip circumference	Stroke	rs9314057	5	C	T	-0.0136589	0.0032	0.00241745	0.0092	1.60E-08	0.7279	9.48E-05	31.92393738
Hip circumference	Stroke	rs10947137	6	C	A	-0.0244192	0.0042	0.00458477	0.0178	1.00E-07	0.8147	8.43E-05	28.36793717
Hip circumference	Stroke	rs12209223	6	A	C	0.0224965	0.0399	0.00398646	0.0151	1.67E-08	0.00833393	9.46E-05	31.84601464
Hip circumference	Stroke	rs12528644	6	A	C	0.0227005	0.008	0.00267839	0.0104	2.35E-17	0.4436	0.000213	71.83286965
Hip circumference	Stroke	rs1294437	6	T	C	0.0186801	0.0173	0.00254412	0.01	2.10E-13	0.0846798	0.00016	53.91172269
Hip circumference	Stroke	rs1847912	6	G	A	-0.0150781	0.003	0.00279356	0.0108	6.76E-08	0.783599	8.65E-05	29.13246487
Hip circumference	Stroke	rs2253310	6	G	C	0.0205891	-0.0096	0.00248737	0.0092	1.26E-16	0.2941	0.000204	68.51630625
Hip circumference	Stroke	rs2499468	6	A	C	0.0142064	-0.0035	0.00253046	0.0096	1.98E-08	0.7175	9.36E-05	31.51876005
Hip circumference	Stroke	rs2814943	6	A	G	0.0550792	0.0109	0.00345869	0.0132	4.47E-57	0.4092	0.000753	253.6015893
Hip circumference	Stroke	rs28366156	6	C	T	-0.0295856	0.0342	0.00355966	0.0179	9.50E-17	0.0560402	0.000205	69.07863156
Hip circumference	Stroke	rs3734554	6	T	C	0.0201484	-0.0019	0.00260948	0.0109	1.16E-14	0.8617	0.000177	59.61742076
Hip circumference	Stroke	rs390192	6	G	A	-0.0140741	-0.0126	0.00242328	0.0094	6.33E-09	0.1783	0.0001	33.73137688
Hip circumference	Stroke	rs41271299	6	T	C	0.0407137	0.0086	0.00543493	0.0298	6.85E-14	0.773701	0.000167	56.11684351
Hip circumference	Stroke	rs4467770	6	A	G	0.0166846	0.0078	0.00271997	0.0105	8.57E-10	0.4562	0.000112	37.62732725
Hip circumference	Stroke	rs4870057	6	G	A	0.0138845	-7.00E-04	0.00256765	0.01	6.40E-08	0.9454	8.69E-05	29.24077247
Hip circumference	Stroke	rs577721086	6	C	T	-0.0699953	-2.00E-04	0.00554393	0.0242	1.56E-36	0.9928	0.000473	159.4051258
Hip circumference	Stroke	rs62396185	6	C	G	-0.0365089	-0.0017	0.00275766	0.0101	5.34E-40	0.864	0.00052	175.2734183
Hip circumference	Stroke	rs62405860	6	C	T	0.0203123	-0.0076	0.00280058	0.0113	4.09E-13	0.5022	0.000156	52.60442021
Hip circumference	Stroke	rs62425398	6	A	C	0.023635	0.013	0.00390514	0.0156	1.43E-09	0.4044	0.000109	36.63009097
Hip circumference	Stroke	rs675162	6	G	A	0.0179101	0.0069	0.00241142	0.009	1.11E-13	0.4471	0.000164	55.16330832
Hip circumference	Stroke	rs6907872	6	T	C	0.0147591	-0.0216	0.0026284	0.01	1.96E-08	0.0310799	9.37E-05	31.53093386
Hip circumference	Stroke	rs72892910	6	T	G	0.0355509	0.0226	0.00320796	0.012	1.55E-28	0.0606401	0.000365	122.8127088
Hip circumference	Stroke	rs7740107	6	A	T	-0.0229511	0.0225	0.002723	0.0106	3.51E-17	0.0333603	0.000211	71.04143539

Hip circumference	Stroke	rs9378684	6	T	C	0.0198865	0.0157	0.00302803	0.0112	5.13E-11	0.1627	0.000128	43.13167875
Hip circumference	Stroke	rs9489620	6	C	G	0.01267	0.0182	0.00241932	0.0101	1.63E-07	0.0706708	8.15E-05	27.42626061
Hip circumference	Stroke	rs9496567	6	A	G	0.021977	0.0144	0.00281121	0.0112	5.40E-15	0.1968	0.000182	61.11534086
Hip circumference	Stroke	rs962554	6	C	T	-0.0218562	0.0034	0.0026826	0.0102	3.73E-16	0.737099	0.000197	66.38003419
Hip circumference	Stroke	rs998584	6	A	C	-0.0211236	0.012	0.00241248	0.0098	2.03E-18	0.2208	0.000228	76.66699245
Hip circumference	Stroke	rs10236214	7	T	C	0.0181559	-0.0309	0.00252278	0.0095	6.18E-13	0.00121001	0.000154	51.79368431
Hip circumference	Stroke	rs10237317	7	G	A	0.014219	0.0259	0.00244995	0.0095	6.49E-09	0.00635799	0.0001	33.68400058
Hip circumference	Stroke	rs113852095	7	T	C	-0.0167002	-0.0097	0.00314782	0.0117	1.13E-07	0.4055	8.36E-05	28.14644591
Hip circumference	Stroke	rs11766945	7	A	G	-0.0197938	-0.0173	0.00302094	0.0112	5.68E-11	0.1226	0.000128	42.93131153
Hip circumference	Stroke	rs1182199	7	A	C	-0.0279324	5.00E-04	0.00261283	0.0116	1.14E-26	0.9681	0.000339	114.2863065
Hip circumference	Stroke	rs12701265	7	A	G	0.0146579	-0.0064	0.00246003	0.0094	2.55E-09	0.4987	0.000105	35.50280788
Hip circumference	Stroke	rs17149254	7	C	T	-0.0194615	0.0162	0.00311973	0.0166	4.43E-10	0.328	0.000116	38.9151405
Hip circumference	Stroke	rs227940	7	G	T	0.0129923	0.0079	0.0024136	0.0093	7.33E-08	0.3945	8.61E-05	28.97620369
Hip circumference	Stroke	rs2289379	7	T	C	-0.0125447	0.0036	0.00246821	0.0094	3.73E-07	0.7002	7.67E-05	25.83189963
Hip circumference	Stroke	rs34748838	7	T	C	0.0204746	-0.0118	0.00240761	0.0097	1.84E-17	0.2228	0.000215	72.32002622
Hip circumference	Stroke	rs36078773	7	G	T	0.0165822	-0.0039	0.00243452	0.0099	9.69E-12	0.695401	0.000138	46.39354987
Hip circumference	Stroke	rs3729793	7	G	C	0.0215897	-0.0135	0.00413262	0.0157	1.75E-07	0.3908	8.11E-05	27.29243401
Hip circumference	Stroke	rs3807566	7	T	G	-0.0159279	0.0046	0.00242654	0.0091	5.24E-11	0.6152	0.000128	43.08659071
Hip circumference	Stroke	rs4722398	7	T	C	0.0222577	0.0073	0.00350131	0.0136	2.06E-10	0.5903	0.00012	40.4109854
Hip circumference	Stroke	rs57116196	7	T	C	0.016147	0.0125	0.00298851	0.0111	6.56E-08	0.2611	8.67E-05	29.19269997
Hip circumference	Stroke	rs58862095	7	T	C	-0.0188631	-0.0271	0.00244247	0.0115	1.14E-14	0.0180198	0.000177	59.64412311
Hip circumference	Stroke	rs6973656	7	G	A	0.0188205	0.0122	0.00245506	0.0093	1.78E-14	0.1881	0.000175	58.7676187
Hip circumference	Stroke	rs982692	7	C	T	0.0144682	-0.0078	0.00250216	0.0097	7.38E-09	0.4211	9.93E-05	33.43480949
Hip circumference	Stroke	rs10100245	8	A	G	0.0204889	-1.00E-04	0.00242571	0.0092	3.01E-17	0.993	0.000212	71.34434061
Hip circumference	Stroke	rs10103997	8	G	C	0.0162422	0.0366	0.00288595	0.0109	1.82E-08	0.000796196	9.41E-05	31.67467028
Hip circumference	Stroke	rs113364497	8	T	C	-0.0157612	0.0215	0.00282326	0.0118	2.37E-08	0.0684495	9.26E-05	31.16569454

Hip circumference	Stroke	rs11997077	8	G	A	-0.0128247	0.0042	0.00249347	0.0098	2.70E-07	0.6687	7.86E-05	26.45368237
Hip circumference	Stroke	rs12543555	8	G	A	-0.0157172	-0.0172	0.00297541	0.0114	1.28E-07	0.1335	8.29E-05	27.90337415
Hip circumference	Stroke	rs12680342	8	G	T	-0.0186937	-0.0088	0.00285757	0.0109	6.09E-11	0.4155	0.000127	42.79536964
Hip circumference	Stroke	rs13264909	8	T	A	-0.0157479	-0.0056	0.00243238	0.009	9.54E-11	0.5351	0.000125	41.91625262
Hip circumference	Stroke	rs2737250	8	G	A	-0.0213899	0.0086	0.00252036	0.0096	2.13E-17	0.3725	0.000214	72.02650664
Hip circumference	Stroke	rs2954021	8	G	A	0.0177343	-0.0014	0.00240615	0.009	1.71E-13	0.874	0.000161	54.32286992
Hip circumference	Stroke	rs310307	8	G	A	-0.0125543	0.0125	0.00242099	0.0093	2.15E-07	0.1822	7.99E-05	26.89050843
Hip circumference	Stroke	rs4872142	8	G	C	-0.0206355	6.00E-04	0.00309464	0.0119	2.59E-11	0.9599	0.000132	44.46412222
Hip circumference	Stroke	rs6470771	8	C	A	-0.0176853	-0.0198	0.00320267	0.0124	3.35E-08	0.1103	9.06E-05	30.49302285
Hip circumference	Stroke	rs6601527	8	A	C	-0.0155086	-0.015	0.00244837	0.0096	2.39E-10	0.1166	0.000119	40.12278685
Hip circumference	Stroke	rs6998644	8	T	C	-0.0128742	-0.0068	0.00242764	0.0091	1.14E-07	0.4583	8.35E-05	28.12366614
Hip circumference	Stroke	rs6999725	8	T	C	-0.0404537	-0.0484	0.00670288	0.0265	1.59E-09	0.0682905	0.000108	36.42450094
Hip circumference	Stroke	rs72656010	8	C	T	-0.0239918	-4.00E-04	0.00357594	0.0136	1.96E-11	0.9773	0.000134	45.01375246
Hip circumference	Stroke	rs7460093	8	A	G	0.0150887	0.0105	0.00243191	0.0103	5.49E-10	0.3103	0.000114	38.4953835
Hip circumference	Stroke	rs77978038	8	G	T	0.0145795	0.0054	0.00286142	0.011	3.49E-07	0.6261	7.71E-05	25.96103746
Hip circumference	Stroke	rs7833077	8	C	G	-0.0132735	4.00E-04	0.00253124	0.0096	1.57E-07	0.9692	8.17E-05	27.49819951
Hip circumference	Stroke	rs7845090	8	A	G	-0.0226011	-0.0093	0.00266783	0.0105	2.43E-17	0.3759	0.000213	71.76998408
Hip circumference	Stroke	rs78565420	8	T	C	0.0276211	0.0179	0.00546097	0.0225	4.24E-07	0.4282	7.60E-05	25.58246325
Hip circumference	Stroke	rs894347	8	G	A	-0.0182603	0.0101	0.0024579	0.0092	1.09E-13	0.2727	0.000164	55.19343169
Hip circumference	Stroke	rs10118701	9	G	A	0.0183313	0.0114	0.00257954	0.0096	1.19E-12	0.2373	0.00015	50.50123471
Hip circumference	Stroke	rs10756798	9	T	C	-0.0154182	1.00E-04	0.00251451	0.0096	8.70E-10	0.9924	0.000112	37.59764263
Hip circumference	Stroke	rs10820852	9	A	C	-0.0162227	0.0059	0.00270056	0.0106	1.89E-09	0.581201	0.000107	36.08598902
Hip circumference	Stroke	rs12346647	9	C	T	0.0156951	0.0026	0.00302959	0.0115	2.21E-07	0.8233	7.97E-05	26.83863704
Hip circumference	Stroke	rs17770336	9	T	C	0.0205165	0.0021	0.00256585	0.0099	1.29E-15	0.8329	0.00019	63.93578789
Hip circumference	Stroke	rs28377268	9	T	G	0.0253898	0.0399	0.00388572	0.0143	6.41E-11	0.00507797	0.000127	42.69485612
Hip circumference	Stroke	rs4297095	9	A	G	-0.0222453	5.00E-04	0.0039494	0.0152	1.78E-08	0.9751	9.42E-05	31.72592479

Hip circumference	Stroke	rs4741546	9	T	C	-0.0177033	-0.0075	0.00246644	0.0093	7.11E-13	0.4232	0.000153	51.51899089
Hip circumference	Stroke	rs937482	9	G	A	-0.0125853	0.0064	0.0024067	0.0092	1.70E-07	0.4869	8.12E-05	27.34533379
Hip circumference	Stroke	rs10883553	10	A	C	0.0160261	0.0209	0.00242185	0.0093	3.66E-11	0.0250599	0.00013	43.78861555
Hip circumference	Stroke	rs10887571	10	T	C	0.0145648	-0.0064	0.00243391	0.0096	2.18E-09	0.5085	0.000106	35.80964541
Hip circumference	Stroke	rs11146442	10	A	C	-0.0209335	0.0121	0.00384937	0.0146	5.39E-08	0.4097	8.79E-05	29.57361256
Hip circumference	Stroke	rs12254441	10	T	C	-0.0149948	-0.0019	0.00257352	0.0102	5.66E-09	0.8488	0.000101	33.9489432
Hip circumference	Stroke	rs12414412	10	G	C	0.0231133	-0.0069	0.00434374	0.0157	1.03E-07	0.6591	8.41E-05	28.31367667
Hip circumference	Stroke	rs12765337	10	C	G	0.0155046	0.0103	0.00253466	0.0093	9.54E-10	0.2709	0.000111	37.41809821
Hip circumference	Stroke	rs12779865	10	C	T	0.0226272	-0.0103	0.00255912	0.0097	9.47E-19	0.2914	0.000232	78.17723918
Hip circumference	Stroke	rs2439823	10	G	A	0.0188359	-0.0127	0.00242211	0.0093	7.47E-15	0.1713	0.00018	60.4762741
Hip circumference	Stroke	rs6585201	10	A	G	-0.0208428	0.0098	0.00241636	0.0096	6.40E-18	0.3067	0.000221	74.40272582
Hip circumference	Stroke	rs7087701	10	G	C	-0.0129751	0.0136	0.00247335	0.0096	1.56E-07	0.1552	8.18E-05	27.52011683
Hip circumference	Stroke	rs7893571	10	T	G	0.0156277	0.004	0.00255052	0.0097	8.95E-10	0.675601	0.000112	37.54331907
Hip circumference	Stroke	rs7915723	10	A	C	-0.0137936	-0.0108	0.0024247	0.0094	1.28E-08	0.2536	9.61E-05	32.3622889
Hip circumference	Stroke	rs79969674	10	T	C	0.0265356	0.0337	0.00462325	0.0176	9.50E-09	0.0549794	9.79E-05	32.94299401
Hip circumference	Stroke	rs845084	10	A	G	0.0180027	0.0034	0.00275957	0.0102	6.87E-11	0.738901	0.000126	42.5591023
Hip circumference	Stroke	rs9415106	10	A	G	-0.0151806	-0.0127	0.00266693	0.01	1.26E-08	0.205	9.62E-05	32.40071847
Hip circumference	Stroke	rs11030119	11	A	G	0.0314539	0.018	0.002604	0.01	1.38E-33	0.0723502	0.000433	145.903948
Hip circumference	Stroke	rs12805742	11	T	C	-0.0185191	-0.0045	0.00285874	0.011	9.30E-11	0.6797	0.000125	41.96531011
Hip circumference	Stroke	rs140201358	11	G	C	-0.0637486	-0.032	0.010272	0.0711	5.44E-10	0.6524	0.000114	38.51512234
Hip circumference	Stroke	rs143840904	11	T	C	-0.0483109	0.0267	0.00902762	0.0478	8.73E-08	0.5765	8.51E-05	28.63806802
Hip circumference	Stroke	rs1662185	11	G	A	0.0170127	-0.0102	0.00265128	0.01	1.39E-10	0.3057	0.000122	41.17516006
Hip circumference	Stroke	rs17245511	11	A	G	-0.0199228	0.0137	0.00338136	0.0131	3.82E-09	0.2982	0.000103	34.71506068
Hip circumference	Stroke	rs2187449	11	A	G	0.0163764	-0.0165	0.00286336	0.0118	1.07E-08	0.1613	9.72E-05	32.71033317
Hip circumference	Stroke	rs35099456	11	C	G	-0.0406467	0.0249	0.00498534	0.0221	3.55E-16	0.2587	0.000197	66.47540917
Hip circumference	Stroke	rs4148172	11	G	A	-0.0235908	0.0511	0.00469185	0.0194	4.96E-07	0.00862899	7.51E-05	25.28116594

Hip circumference	Stroke	rs4936671	11	G	C	-0.0127849	0.008	0.00252321	0.0095	4.05E-07	0.3982	7.63E-05	25.67366538
Hip circumference	Stroke	rs667515	11	C	G	-0.0139118	0.0278	0.00247623	0.0104	1.93E-08	0.00767503	9.38E-05	31.56346615
Hip circumference	Stroke	rs7116641	11	G	T	0.0218983	0.032	0.00258946	0.0097	2.76E-17	0.00101	0.000212	71.51586384
Hip circumference	Stroke	rs7124681	11	A	C	0.019971	0.0189	0.00244272	0.0092	2.95E-16	0.04062	0.000199	66.84243261
Hip circumference	Stroke	rs74749286	11	A	G	0.0306898	-0.0123	0.00389923	0.015	3.54E-15	0.4107	0.000184	61.94844508
Hip circumference	Stroke	rs7480253	11	T	C	0.0125489	0.0149	0.0024362	0.0094	2.59E-07	0.1132	7.88E-05	26.53294393
Hip circumference	Stroke	rs7930275	11	T	C	0.0186124	-0.0085	0.0029418	0.0119	2.50E-10	0.4749	0.000119	40.0293435
Hip circumference	Stroke	rs7940866	11	A	T	0.0129611	0.013	0.00241693	0.0098	8.21E-08	0.1859	8.54E-05	28.75779463
Hip circumference	Stroke	rs10777859	12	G	A	-0.0154525	-0.0067	0.00240837	0.0091	1.40E-10	0.4614	0.000122	41.16717703
Hip circumference	Stroke	rs11056870	12	T	G	0.0173557	0.0145	0.00341687	0.0129	3.79E-07	0.2633	7.66E-05	25.80045457
Hip circumference	Stroke	rs11109097	12	T	C	-0.0156389	-0.002	0.0026263	0.01	2.61E-09	0.8395	0.000105	35.45877582
Hip circumference	Stroke	rs12300276	12	A	G	0.0159752	-0.0054	0.00283175	0.011	1.69E-08	0.6277	9.45E-05	31.82605358
Hip circumference	Stroke	rs1271309	12	G	A	-0.0219913	0.009	0.00325813	0.0128	1.48E-11	0.4833	0.000135	45.55803551
Hip circumference	Stroke	rs1458156	12	T	C	0.0133122	0.0016	0.0024078	0.0093	3.23E-08	0.8651	9.08E-05	30.56742444
Hip circumference	Stroke	rs147730268	12	T	G	-0.0522646	0.0273	0.00433365	0.0183	1.74E-33	0.1372	0.000432	145.4479469
Hip circumference	Stroke	rs1964599	12	T	C	-0.022999	0.0117	0.0025863	0.0106	6.00E-19	0.2677	0.000235	79.07880664
Hip circumference	Stroke	rs1979440	12	C	T	-0.0149999	0.0132	0.00245372	0.0093	9.78E-10	0.1565	0.000111	37.37031184
Hip circumference	Stroke	rs2129869	12	T	A	-0.0214884	-0.0096	0.00291524	0.0112	1.70E-13	0.3898	0.000161	54.33248024
Hip circumference	Stroke	rs2897968	12	A	G	0.0137469	0.0036	0.00246483	0.0093	2.45E-08	0.694201	9.24E-05	31.10538667
Hip circumference	Stroke	rs34911908	12	A	G	0.0130448	0.0038	0.00248022	0.0095	1.44E-07	0.689	8.22E-05	27.66269189
Hip circumference	Stroke	rs36120387	12	T	C	-0.0203173	0.0481	0.00388325	0.0159	1.68E-07	0.00252	8.13E-05	27.37419047
Hip circumference	Stroke	rs3730071	12	A	C	-0.0378377	0.0424	0.00701291	0.0294	6.84E-08	0.1498	8.65E-05	29.11071874
Hip circumference	Stroke	rs4842681	12	G	C	0.0152955	0.024	0.00268676	0.0101	1.25E-08	0.0170899	9.63E-05	32.40929685
Hip circumference	Stroke	rs55726687	12	A	G	0.0213623	0.0079	0.00294561	0.0117	4.11E-13	0.5009	0.000156	52.59512946
Hip circumference	Stroke	rs6539064	12	G	C	-0.0187741	0.0105	0.00276939	0.0103	1.21E-11	0.3093	0.000137	45.9568247
Hip circumference	Stroke	rs66630777	12	T	G	-0.0150299	0.0021	0.0027754	0.0106	6.12E-08	0.8445	8.71E-05	29.32655263

Hip circumference	Stroke	rs697883	12	A	G	-0.0223877	0.0418	0.00429796	0.0174	1.90E-07	0.0162499	8.06E-05	27.13277533
Hip circumference	Stroke	rs7132908	12	A	G	0.0248529	1.00E-04	0.00247489	0.0103	1.00E-23	0.9933	0.0003	100.8422072
Hip circumference	Stroke	rs7305790	12	C	A	0.013906	0.0021	0.00268774	0.0101	2.29E-07	0.8331	7.95E-05	26.76886397
Hip circumference	Stroke	rs76895963	12	G	T	0.0962201	-0.0588	0.00932168	0.0511	5.64E-25	0.2495	0.000316	106.5474911
Hip circumference	Stroke	rs78470967	12	A	T	0.0359219	-0.0051	0.00606978	0.0266	3.26E-09	0.8485	0.000104	35.02456084
Hip circumference	Stroke	rs882378	12	C	A	0.0154797	0.0093	0.00261008	0.0113	3.02E-09	0.4099	0.000104	35.17365032
Hip circumference	Stroke	rs11839227	13	C	T	-0.0181298	-0.004	0.00311049	0.0118	5.59E-09	0.734301	0.000101	33.97257041
Hip circumference	Stroke	rs12877270	13	A	G	0.0151117	0.0032	0.00244173	0.0093	6.06E-10	0.7284	0.000114	38.30287457
Hip circumference	Stroke	rs1441264	13	A	G	0.0168087	-0.0092	0.00249936	0.0095	1.76E-11	0.3342	0.000134	45.22833725
Hip circumference	Stroke	rs1886558	13	G	A	-0.0159051	-0.0058	0.00248456	0.0096	1.54E-10	0.550399	0.000122	40.980177
Hip circumference	Stroke	rs41284816	13	T	G	0.0791573	0.0047	0.00891062	0.0318	6.51E-19	0.8819	0.000234	78.91618788
Hip circumference	Stroke	rs7982447	13	C	T	0.0162648	-0.0091	0.0029811	0.0114	4.87E-08	0.4231	8.84E-05	29.76763731
Hip circumference	Stroke	rs9512696	13	G	A	0.0176296	-0.0156	0.00254397	0.0096	4.22E-12	0.1065	0.000143	48.02428926
Hip circumference	Stroke	rs9513030	13	C	G	-0.0132237	0.0136	0.00248635	0.0097	1.05E-07	0.158	8.40E-05	28.28664557
Hip circumference	Stroke	rs9565536	13	A	T	-0.0143477	0.0024	0.00269818	0.0102	1.05E-07	0.812	8.40E-05	28.27631018
Hip circumference	Stroke	rs9603697	13	T	C	0.0139508	0.0201	0.00256358	0.0098	5.27E-08	0.0397402	8.80E-05	29.61450486
Hip circumference	Stroke	rs1285997	14	G	C	0.0234272	-0.0218	0.0026491	0.0103	9.33E-19	0.0342098	0.000232	78.20671877
Hip circumference	Stroke	rs12883788	14	T	C	0.0154765	-0.0105	0.00242117	0.0096	1.64E-10	0.2762	0.000121	40.85967764
Hip circumference	Stroke	rs1955695	14	G	A	-0.0191353	0.009	0.00248689	0.0094	1.42E-14	0.3394	0.000176	59.2048655
Hip circumference	Stroke	rs28479795	14	T	C	0.0244818	0.0173	0.00289895	0.0111	3.05E-17	0.1197	0.000212	71.31899944
Hip circumference	Stroke	rs3803286	14	G	A	-0.0175828	0.0099	0.00254731	0.0096	5.12E-12	0.3014	0.000142	47.64446682
Hip circumference	Stroke	rs6575340	14	A	G	0.0204335	0.0099	0.0025066	0.0095	3.60E-16	0.297	0.000197	66.45313187
Hip circumference	Stroke	rs71413981	14	A	G	0.0191825	-0.0299	0.00325375	0.0134	3.74E-09	0.0253402	0.000103	34.7569814
Hip circumference	Stroke	rs7145337	14	T	C	-0.0159056	0.0083	0.00265409	0.0102	2.06E-09	0.4128	0.000107	35.91441705
Hip circumference	Stroke	rs8011368	14	T	C	-0.016411	0.0216	0.00270853	0.0106	1.37E-09	0.0418004	0.000109	36.71155585
Hip circumference	Stroke	rs9323375	14	A	T	0.0164338	-0.0174	0.00284639	0.0111	7.77E-09	0.1171	9.90E-05	33.33398108

Hip circumference	Stroke	rs9788550	14	C	G	-0.0207023	-0.0072	0.00279459	0.0107	1.29E-13	0.502	0.000163	54.87834355
Hip circumference	Stroke	rs1559676	15	G	C	-0.0142302	-0.008	0.00265959	0.01	8.78E-08	0.4235	8.50E-05	28.62810657
Hip circumference	Stroke	rs2715439	15	T	C	0.0157972	0.0079	0.00241641	0.0092	6.27E-11	0.3919	0.000127	42.73847135
Hip circumference	Stroke	rs34769775	15	T	C	-0.0165941	-0.0162	0.00262638	0.0103	2.65E-10	0.1149	0.000119	39.92016069
Hip circumference	Stroke	rs35874463	15	G	A	0.0325725	0.0673	0.00514563	0.025	2.45E-10	0.00699198	0.000119	40.07052901
Hip circumference	Stroke	rs4776970	15	T	A	-0.0201543	-0.021	0.002508	0.0096	9.31E-16	0.0278298	0.000192	64.5773729
Hip circumference	Stroke	rs4777541	15	T	C	0.0191721	0.0176	0.00284958	0.0111	1.72E-11	0.1139	0.000134	45.26657785
Hip circumference	Stroke	rs4843158	15	C	G	0.0202544	0.0104	0.00258511	0.0096	4.70E-15	0.2809	0.000182	61.38760779
Hip circumference	Stroke	rs4966012	15	G	C	0.0149488	0.0089	0.00257511	0.01	6.44E-09	0.3687	0.0001	33.69931649
Hip circumference	Stroke	rs55707100	15	T	C	-0.0400543	0.0553	0.00774721	0.0291	2.34E-07	0.0575904	7.94E-05	26.73053407
Hip circumference	Stroke	rs8023263	15	T	G	0.0148049	0.0089	0.00240753	0.0094	7.79E-10	0.3445	0.000112	37.81529925
Hip circumference	Stroke	rs8042404	15	A	G	0.0170942	-0.001	0.00270872	0.0101	2.78E-10	0.9186	0.000118	39.82624242
Hip circumference	Stroke	rs11150461	16	G	C	-0.0150251	-0.0121	0.00271243	0.0108	3.04E-08	0.2602	9.12E-05	30.68440201
Hip circumference	Stroke	rs1150188	16	C	G	-0.0144553	-0.0054	0.0024709	0.0095	4.91E-09	0.5692	0.000102	34.22503333
Hip circumference	Stroke	rs117342986	16	T	C	0.0410233	0.019	0.00782839	0.03	1.60E-07	0.5253	8.16E-05	27.46099563
Hip circumference	Stroke	rs12920259	16	A	G	-0.0164136	4.00E-04	0.00247121	0.0094	3.10E-11	0.9692	0.000131	44.11521351
Hip circumference	Stroke	rs13333747	16	C	T	-0.0239707	-0.0309	0.00313212	0.0128	1.97E-14	0.0161499	0.000174	58.57127093
Hip circumference	Stroke	rs25849	16	G	C	0.020294	0.0057	0.00266649	0.0114	2.73E-14	0.6167	0.000172	57.92357968
Hip circumference	Stroke	rs35057083	16	T	C	0.0144062	0.0034	0.00260593	0.0106	3.24E-08	0.7487	9.08E-05	30.56141008
Hip circumference	Stroke	rs4402589	16	G	T	0.0295957	-0.0167	0.00241926	0.0093	2.10E-34	0.0717993	0.000444	149.6553142
Hip circumference	Stroke	rs4985407	16	G	A	0.0153191	-0.0023	0.00240871	0.0096	2.02E-10	0.8116	0.00012	40.448039
Hip circumference	Stroke	rs56094641	16	G	A	0.0610681	0.0061	0.00245004	0.0092	5.26E-137	0.512201	0.001842	621.2729808
Hip circumference	Stroke	rs62037365	16	G	C	0.0322128	-0.0037	0.00245225	0.0092	2.09E-39	0.691301	0.000512	172.5549674
Hip circumference	Stroke	rs72801854	16	A	G	0.0183289	-0.0067	0.00261408	0.01	2.36E-12	0.5002	0.000146	49.162624
Hip circumference	Stroke	rs756717	16	A	G	-0.015681	0.0114	0.00248428	0.0096	2.76E-10	0.2363	0.000118	39.84248553
Hip circumference	Stroke	rs879620	16	T	C	0.027011	0.0115	0.00248095	0.0097	1.34E-27	0.2331	0.000352	118.5346447

Hip circumference	Stroke	rs10153248	17	G	A	-0.0170889	0.0164	0.0024225	0.0094	1.74E-12	0.08077	0.000148	49.76232294
Hip circumference	Stroke	rs113866544	17	C	T	0.0327216	-0.0071	0.00479193	0.0173	8.60E-12	0.6814	0.000139	46.62814434
Hip circumference	Stroke	rs1396513	17	T	C	0.0183151	0.0113	0.00240892	0.0099	2.90E-14	0.2552	0.000172	57.80612182
Hip circumference	Stroke	rs1918249	17	A	T	0.0159582	-0.0264	0.00281683	0.0104	1.47E-08	0.0113201	9.53E-05	32.09567639
Hip circumference	Stroke	rs2355374	17	T	G	0.0128665	0.0062	0.00243068	0.0093	1.20E-07	0.5031	8.32E-05	28.01981565
Hip circumference	Stroke	rs3826408	17	T	C	0.0151967	0.0101	0.00241335	0.0093	3.04E-10	0.278	0.000118	39.65134822
Hip circumference	Stroke	rs4790292	17	A	C	-0.027819	-0.0163	0.00334073	0.0131	8.31E-17	0.2131	0.000206	69.34262448
Hip circumference	Stroke	rs4794222	17	G	A	-0.0174748	0.0012	0.00276456	0.0108	2.60E-10	0.9146	0.000119	39.95511526
Hip circumference	Stroke	rs4889867	17	T	C	-0.0165547	-0.0051	0.00242054	0.0101	7.97E-12	0.6141	0.000139	46.77546378
Hip circumference	Stroke	rs56288810	17	G	A	0.0183373	0.0033	0.00294765	0.0113	4.95E-10	0.766999	0.000115	38.70071163
Hip circumference	Stroke	rs58551145	17	G	A	0.0238057	0.0097	0.00303824	0.0115	4.69E-15	0.3988	0.000182	61.39284484
Hip circumference	Stroke	rs6501601	17	A	G	-0.01902	-0.0061	0.00246888	0.01	1.32E-14	0.5465	0.000176	59.3500463
Hip circumference	Stroke	rs7226064	17	G	A	-0.01458	-0.0017	0.00243911	0.0093	2.27E-09	0.8529	0.000106	35.73158444
Hip circumference	Stroke	rs731758	17	G	C	-0.0176334	-5.00E-04	0.00248153	0.0096	1.20E-12	0.9584	0.00015	50.49321905
Hip circumference	Stroke	rs11661691	18	G	T	0.0130311	-0.0052	0.00240751	0.0095	6.21E-08	0.581	8.70E-05	29.2971892
Hip circumference	Stroke	rs11664106	18	T	A	0.015224	0.0057	0.002547	0.0111	2.27E-09	0.6096	0.000106	35.7272559
Hip circumference	Stroke	rs12607512	18	G	A	0.0131918	0.0046	0.00241734	0.0092	4.84E-08	0.6194	8.85E-05	29.7805449
Hip circumference	Stroke	rs12965488	18	T	C	0.0168958	-0.0017	0.00312596	0.0119	6.49E-08	0.8896	8.68E-05	29.21397728
Hip circumference	Stroke	rs1618725	18	T	C	-0.021172	-0.0229	0.00240567	0.0091	1.36E-18	0.0120901	0.00023	77.45539373
Hip circumference	Stroke	rs273759	18	G	A	-0.0129908	0.0034	0.00241253	0.0092	7.26E-08	0.7117	8.61E-05	28.99521599
Hip circumference	Stroke	rs57636386	18	C	T	-0.036841	0.0231	0.004358	0.0164	2.83E-17	0.1593	0.000212	71.46417233
Hip circumference	Stroke	rs6567160	18	C	T	0.0496794	-6.00E-04	0.00283765	0.0106	1.35E-68	0.9543	0.00091	306.5032107
Hip circumference	Stroke	rs7238896	18	G	A	0.0212081	0.0039	0.00347746	0.0131	1.07E-09	0.764901	0.00011	37.194544
Hip circumference	Stroke	rs7241211	18	T	C	0.0130235	0.0148	0.00243868	0.0094	9.28E-08	0.1133	8.47E-05	28.51975534
Hip circumference	Stroke	rs8087074	18	T	G	0.0144204	0.0099	0.00275049	0.0106	1.58E-07	0.3465	8.17E-05	27.48745136
Hip circumference	Stroke	rs8097809	18	A	G	-0.017963	0.001	0.00355561	0.0132	4.37E-07	0.9385	7.58E-05	25.52286961

Hip circumference	Stroke	rs9967367	18	T	C	-0.0164671	-0.0089	0.00265273	0.0101	5.39E-10	0.3751	0.000114	38.53435978
Hip circumference	Stroke	rs10404726	19	T	C	-0.0166254	-0.0216	0.00240995	0.0092	5.26E-12	0.0186801	0.000141	47.59136285
Hip circumference	Stroke	rs11882409	19	A	C	0.0181856	0.0059	0.00270443	0.0103	1.77E-11	0.5652	0.000134	45.21721177
Hip circumference	Stroke	rs1231281	19	A	G	-0.013948	0.0082	0.00240776	0.0093	6.92E-09	0.3771	9.97E-05	33.5581095
Hip circumference	Stroke	rs12972720	19	C	G	0.0177427	-1.00E-04	0.00243123	0.0094	2.93E-13	0.9911	0.000158	53.25830331
Hip circumference	Stroke	rs141622900	19	A	G	0.0316845	-0.0524	0.00551034	0.0238	8.93E-09	0.0272797	9.82E-05	33.06259391
Hip circumference	Stroke	rs2238689	19	C	T	-0.0126136	0.0225	0.00245659	0.0095	2.83E-07	0.0179602	7.83E-05	26.36408784
Hip circumference	Stroke	rs273505	19	C	T	0.0147072	0.0174	0.00243691	0.0095	1.59E-09	0.0669993	0.000108	36.42344472
Hip circumference	Stroke	rs3218036	19	A	G	0.0194082	-0.003	0.0025688	0.0098	4.19E-14	0.755499	0.00017	57.08341315
Hip circumference	Stroke	rs34013042	19	T	C	0.0154229	-0.0028	0.00275189	0.0103	2.09E-08	0.7833	9.33E-05	31.41014529
Hip circumference	Stroke	rs350832	19	A	G	0.0151016	0.0326	0.00287737	0.0114	1.53E-07	0.00415002	8.18E-05	27.54574375
Hip circumference	Stroke	rs3810291	19	A	G	0.023107	-0.0041	0.0025705	0.0105	2.50E-19	0.698601	0.00024	80.80754491
Hip circumference	Stroke	rs1293395	20	T	G	-0.0274278	-0.0419	0.00453926	0.0173	1.52E-09	0.0158099	0.000108	36.50999975
Hip circumference	Stroke	rs2236519	20	A	G	-0.0208582	0.0074	0.00248544	0.0095	4.79E-17	0.4383	0.000209	70.42828092
Hip circumference	Stroke	rs3746759	20	G	T	-0.017084	0.0096	0.00299536	0.0116	1.17E-08	0.4068	9.66E-05	32.52977607
Hip circumference	Stroke	rs55650227	20	C	G	-0.0198726	-0.0202	0.00307086	0.0117	9.73E-11	0.0860003	0.000124	41.87832926
Hip circumference	Stroke	rs55886426	20	G	C	-0.0337871	-0.0428	0.00552551	0.0285	9.68E-10	0.1326	0.000111	37.3901404
Hip circumference	Stroke	rs6029180	20	G	A	0.0136196	0.0341	0.00259026	0.01	1.46E-07	0.000685299	8.21E-05	27.64661645
Hip circumference	Stroke	rs6080646	20	A	G	-0.0133714	1.00E-04	0.00241122	0.0092	2.93E-08	0.9873	9.14E-05	30.75247591
Hip circumference	Stroke	rs6111562	20	T	G	0.0334476	-0.0429	0.00616057	0.0258	5.66E-08	0.0956203	8.76E-05	29.47732898
Hip circumference	Stroke	rs6142059	20	C	T	0.0159821	0.0072	0.00240831	0.0093	3.22E-11	0.4396	0.000131	44.03955373
Hip circumference	Stroke	rs7274811	20	T	G	-0.0211475	0.0014	0.00275032	0.011	1.49E-14	0.896	0.000176	59.12233982
Hip circumference	Stroke	rs10427502	21	A	G	-0.0129865	0.0076	0.00248172	0.0095	1.67E-07	0.4219	8.13E-05	27.38285195
Hip circumference	Stroke	rs60984707	21	T	C	-0.018209	0.001	0.00313296	0.0118	6.18E-09	0.9321	0.0001	33.7802214
Hip circumference	Stroke	rs76040172	21	A	G	-0.034453	0.0038	0.0053139	0.0212	8.97E-11	0.8595	0.000125	42.03657594
Hip circumference	Stroke	rs8133137	21	G	A	0.0150007	-0.0112	0.00255038	0.0097	4.06E-09	0.2462	0.000103	34.59499429

Hip circumference	Stroke	rs11704728	22	T	C	0.0152795	0.0054	0.00303796	0.0114	4.92E-07	0.6336	7.51E-05	25.29613573
Hip circumference	Stroke	rs133015	22	G	C	0.0129392	-0.0119	0.00242369	0.0093	9.37E-08	0.1988	8.47E-05	28.50104013
Hip circumference	Stroke	rs138767	22	C	T	0.0146128	0.0018	0.0025195	0.0096	6.64E-09	0.8506	9.99E-05	33.63861879
Hip circumference	Stroke	rs140947018	22	A	G	0.0369121	-0.0034	0.00727511	0.0284	3.90E-07	0.9058	7.65E-05	25.7429539
Hip circumference	Stroke	rs1569497	22	G	A	0.0148474	-0.0086	0.00241868	0.0091	8.33E-10	0.3442	0.000112	37.68287163
Waist circumference	Stroke	rs11165493	1	A	G	0.0118676	-0.013	0.002282	0.0111	1.99E-07	0.2393	8.03E-05	27.04546916
Waist circumference	Stroke	rs11208779	1	C	G	0.0129711	0.0296	0.00216129	0.0093	1.96E-09	0.001424	0.000107	36.01865794
Waist circumference	Stroke	rs112566467	1	T	C	0.0181268	0.0034	0.00264517	0.0111	7.25E-12	0.759401	0.000139	46.96075916
Waist circumference	Stroke	rs12037698	1	A	G	-0.0165932	0.0087	0.00308427	0.0127	7.46E-08	0.4923	8.60E-05	28.94379786
Waist circumference	Stroke	rs12060713	1	A	G	0.0122165	-0.0037	0.00220788	0.0092	3.15E-08	0.6893	9.09E-05	30.61559243
Waist circumference	Stroke	rs12096864	1	C	T	0.0215521	0.0155	0.00336684	0.0138	1.54E-10	0.2632	0.000122	40.97644057
Waist circumference	Stroke	rs12128526	1	A	G	0.0119797	0.0012	0.00216039	0.0092	2.94E-08	0.8972	9.13E-05	30.74875727
Waist circumference	Stroke	rs12140153	1	T	G	-0.0252672	-0.0119	0.00373213	0.017	1.29E-11	0.4833	0.000136	45.83536668
Waist circumference	Stroke	rs12725767	1	G	A	0.0118961	-0.0112	0.00225266	0.0098	1.29E-07	0.2532	8.28E-05	27.88803518
Waist circumference	Stroke	rs1490382	1	G	A	-0.0113387	-0.014	0.00221817	0.0096	3.19E-07	0.1445	7.76E-05	26.1298475
Waist circumference	Stroke	rs241461	1	A	T	-0.0181483	5.00E-04	0.00230559	0.0099	3.52E-15	0.957	0.000184	61.95947652
Waist circumference	Stroke	rs2678204	1	G	T	0.0194317	0.0173	0.00227049	0.0098	1.15E-17	0.0779992	0.000218	73.24574605
Waist circumference	Stroke	rs3028171	1	C	A	0.0142348	-0.0201	0.00267155	0.0125	9.92E-08	0.1074	8.43E-05	28.39070165
Waist circumference	Stroke	rs309535	1	G	A	-0.0152186	-0.0088	0.0029032	0.0126	1.59E-07	0.4829	8.16E-05	27.47865645
Waist circumference	Stroke	rs3766823	1	A	G	0.0155791	-0.0035	0.00285308	0.0123	4.75E-08	0.773599	8.86E-05	29.81651158
Waist circumference	Stroke	rs3935032	1	T	C	-0.014629	0.0382	0.00225489	0.0188	8.73E-11	0.0417503	0.000125	42.08996442
Waist circumference	Stroke	rs41279738	1	G	T	0.05059	0.003	0.00679322	0.0276	9.56E-14	0.9133	0.000165	55.45976177
Waist circumference	Stroke	rs4322261	1	A	G	-0.0183121	-0.0123	0.00290548	0.012	2.93E-10	0.3034	0.000118	39.72286113
Waist circumference	Stroke	rs4562625	1	G	C	-0.0116097	-0.0214	0.00220784	0.0093	1.45E-07	0.0217801	8.21E-05	27.65074267
Waist circumference	Stroke	rs4652839	1	G	C	0.0120158	0.0115	0.00229333	0.0096	1.61E-07	0.2328	8.15E-05	27.45189173
Waist circumference	Stroke	rs4926726	1	A	G	-0.0113354	-0.0068	0.00215892	0.0091	1.52E-07	0.4593	8.19E-05	27.56770424

Waist circumference	Stroke	rs539515	1	C	A	0.0353614	-0.0022	0.00265034	0.0122	1.35E-40	0.8593	0.000529	178.0146397
Waist circumference	Stroke	rs588660	1	A	G	0.0144747	-0.0024	0.00218549	0.0093	3.52E-11	0.792399	0.00013	43.86533955
Waist circumference	Stroke	rs61813324	1	T	C	0.0201339	0.0056	0.00319021	0.0152	2.77E-10	0.710699	0.000118	39.83063869
Waist circumference	Stroke	rs61826867	1	G	A	0.0189886	-0.0351	0.00343971	0.0146	3.38E-08	0.0161499	9.05E-05	30.47489614
Waist circumference	Stroke	rs6687953	1	G	A	0.0132664	-0.0059	0.00220383	0.0093	1.75E-09	0.5267	0.000108	36.23681294
Waist circumference	Stroke	rs71658797	1	A	T	0.0294912	0.0245	0.00327841	0.0148	2.36E-19	0.097481	0.00024	80.92046268
Waist circumference	Stroke	rs7531118	1	C	T	0.015718	-0.0077	0.00216988	0.0093	4.37E-13	0.4082	0.000156	52.47145838
Waist circumference	Stroke	rs815163	1	C	T	-0.0135052	0.0073	0.00216641	0.0093	4.55E-10	0.432	0.000115	38.86160533
Waist circumference	Stroke	rs10172196	2	A	G	0.0140208	-0.0014	0.00234352	0.0105	2.20E-09	0.8929	0.000106	35.79383735
Waist circumference	Stroke	rs10803762	2	A	G	0.0144744	0.0214	0.00231166	0.0097	3.82E-10	0.0277703	0.000116	39.20606214
Waist circumference	Stroke	rs114964326	2	A	G	-0.0342563	0.0138	0.00640531	0.0342	8.89E-08	0.6866	8.50E-05	28.60227646
Waist circumference	Stroke	rs12619178	2	T	C	-0.0150737	-2.00E-04	0.00219213	0.0093	6.15E-12	0.982	0.00014	47.28322867
Waist circumference	Stroke	rs13022337	2	G	A	0.0382185	0.0056	0.00284978	0.0121	5.34E-41	0.6413	0.000534	179.8558661
Waist circumference	Stroke	rs13420048	2	A	C	-0.0139516	0.0032	0.00224737	0.0096	5.37E-10	0.7383	0.000114	38.53886092
Waist circumference	Stroke	rs13423444	2	A	G	0.0178103	0.0024	0.00311058	0.0135	1.03E-08	0.8601	9.74E-05	32.78383227
Waist circumference	Stroke	rs13427822	2	G	A	-0.0159741	0.0063	0.002443	0.0105	6.21E-11	0.5485	0.000127	42.75489692
Waist circumference	Stroke	rs1405261	2	A	T	-0.0113254	-0.0229	0.00217773	0.0095	1.99E-07	0.0164301	8.03E-05	27.04575015
Waist circumference	Stroke	rs1609303	2	A	T	0.0171103	0.0053	0.002238	0.0095	2.09E-14	0.5762	0.000174	58.4514224
Waist circumference	Stroke	rs1881934	2	T	A	0.0121349	0.0172	0.00227206	0.0099	9.25E-08	0.0810793	8.47E-05	28.52547019
Waist circumference	Stroke	rs2015769	2	G	A	-0.0114133	0.0104	0.00216238	0.0093	1.31E-07	0.2613	8.27E-05	27.85854524
Waist circumference	Stroke	rs2196150	2	T	G	-0.0111685	-0.0142	0.00220711	0.0101	4.19E-07	0.1599	7.61E-05	25.60599994
Waist circumference	Stroke	rs2433733	2	A	G	-0.0168831	-0.0076	0.00230959	0.0097	2.68E-13	0.4322	0.000159	53.43608164
Waist circumference	Stroke	rs2861692	2	C	T	-0.0177333	0.003	0.00240824	0.0102	1.79E-13	0.772499	0.000161	54.22250672
Waist circumference	Stroke	rs4482463	2	A	C	-0.0288006	-0.0425	0.00406987	0.0175	1.48E-12	0.01495	0.000149	50.07742585
Waist circumference	Stroke	rs4549080	2	T	C	0.0125556	0.0108	0.00226903	0.0096	3.14E-08	0.2603	9.09E-05	30.61924423
Waist circumference	Stroke	rs4670172	2	A	T	0.0116004	0.0139	0.0022548	0.01	2.68E-07	0.1644	7.86E-05	26.46853333

Waist circumference	Stroke	rs4671328	2	G	T	-0.0163995	0.0011	0.00218011	0.0092	5.39E-14	0.9057	0.000168	56.58540131
Waist circumference	Stroke	rs62106258	2	C	T	-0.0680054	0.0255	0.00500793	0.0277	5.43E-42	0.3567	0.000547	184.4039839
Waist circumference	Stroke	rs6433243	2	C	T	-0.0143354	0.0028	0.00225296	0.0096	1.98E-10	0.768399	0.00012	40.48672696
Waist circumference	Stroke	rs6711584	2	A	G	0.0117155	0.013	0.00216817	0.0092	6.54E-08	0.1576	8.67E-05	29.19678251
Waist circumference	Stroke	rs6739755	2	G	A	-0.016792	-0.0171	0.0022029	0.0094	2.49E-14	0.06952	0.000173	58.10523812
Waist circumference	Stroke	rs72844755	2	C	A	0.0200084	-0.0136	0.00397385	0.0162	4.78E-07	0.3988	7.53E-05	25.35139035
Waist circumference	Stroke	rs72917544	2	A	G	-0.0168827	0.0011	0.00278077	0.0121	1.27E-09	0.928	0.000109	36.85985883
Waist circumference	Stroke	rs73985439	2	C	A	0.014277	0.0146	0.00233516	0.0101	9.73E-10	0.1497	0.000111	37.38011502
Waist circumference	Stroke	rs76286777	2	C	T	0.0263558	0.0101	0.00260034	0.0118	3.87E-24	0.3924	0.000305	102.7287807
Waist circumference	Stroke	rs80330591	2	A	G	-0.0166223	0.0335	0.00303827	0.0127	4.48E-08	0.00841105	8.89E-05	29.93157028
Waist circumference	Stroke	rs1154988	3	A	T	0.0175068	0.0014	0.00257031	0.0111	9.70E-12	0.8989	0.000138	46.39193945
Waist circumference	Stroke	rs11921483	3	A	C	-0.0110359	-0.0178	0.00216076	0.0091	3.27E-07	0.0494903	7.75E-05	26.08569727
Waist circumference	Stroke	rs13322435	3	G	A	-0.0163963	-0.0217	0.00220729	0.0095	1.10E-13	0.0223702	0.000164	55.17888499
Waist circumference	Stroke	rs1454687	3	G	C	-0.0176908	-0.005	0.00215185	0.0094	2.02E-16	0.5923	0.000201	67.58821181
Waist circumference	Stroke	rs1515733	3	C	G	0.012473	-0.001	0.00214986	0.009	6.57E-09	0.9109	1.00E-04	33.66057155
Waist circumference	Stroke	rs17639996	3	A	G	-0.0165482	0.0084	0.00301196	0.0131	3.93E-08	0.520701	8.97E-05	30.18583003
Waist circumference	Stroke	rs2016469	3	A	G	0.0123857	0.0067	0.00224103	0.0095	3.26E-08	0.4821	9.07E-05	30.54539688
Waist circumference	Stroke	rs2035831	3	C	G	-0.0118235	-0.0011	0.00229195	0.0096	2.49E-07	0.9124	7.90E-05	26.61226463
Waist circumference	Stroke	rs2455821	3	A	C	0.0152548	0.0132	0.00242133	0.0104	2.98E-10	0.2031	0.000118	39.69219081
Waist circumference	Stroke	rs4856407	3	T	C	0.0154749	0.0055	0.00223286	0.0095	4.20E-12	0.5633	0.000143	48.03222612
Waist circumference	Stroke	rs59815219	3	T	C	0.0113077	-9.00E-04	0.00215144	0.0092	1.47E-07	0.9228	8.21E-05	27.62422343
Waist circumference	Stroke	rs62246314	3	A	G	0.0216879	0.0202	0.00356414	0.015	1.17E-09	0.179	0.00011	37.02759412
Waist circumference	Stroke	rs62261725	3	G	A	-0.0181763	-0.0058	0.00229544	0.0098	2.41E-15	0.5495	0.000186	62.70166503
Waist circumference	Stroke	rs73140125	3	G	A	-0.0192219	0.0073	0.0032274	0.0143	2.59E-09	0.609101	0.000105	35.47211124
Waist circumference	Stroke	rs7610647	3	G	A	-0.0121287	-0.0067	0.00237559	0.0102	3.30E-07	0.5097	7.74E-05	26.06666864
Waist circumference	Stroke	rs7635592	3	T	C	0.0213705	-0.0122	0.00267244	0.0123	1.28E-15	0.3216	0.00019	63.94600831

Waist circumference	Stroke	rs8192675	3	C	T	0.0153014	-7.00E-04	0.00237273	0.0099	1.13E-10	0.9418	0.000124	41.5877758
Waist circumference	Stroke	rs869400	3	G	T	0.0201061	0.0171	0.00278235	0.0117	4.97E-13	0.1446	0.000155	52.21943348
Waist circumference	Stroke	rs9289630	3	C	G	0.0155282	0.0114	0.00221351	0.0097	2.30E-12	0.2417	0.000146	49.21293373
Waist circumference	Stroke	rs9814633	3	A	G	0.0117553	-0.0057	0.0022665	0.0096	2.14E-07	0.554799	7.99E-05	26.90022962
Waist circumference	Stroke	rs9843653	3	C	T	0.0209096	0.0141	0.00215278	0.0092	2.68E-22	0.1257	0.00028	94.33919143
Waist circumference	Stroke	rs9849919	3	T	C	-0.0114633	0.0137	0.00226642	0.0097	4.24E-07	0.1574	7.60E-05	25.58223762
Waist circumference	Stroke	rs9867068	3	G	C	0.0181689	0.0076	0.00249685	0.0116	3.43E-13	0.5112	0.000157	52.95078026
Waist circumference	Stroke	rs9968060	3	T	C	0.0150834	0.0023	0.00228856	0.0096	4.38E-11	0.8152	0.000129	43.43840706
Waist circumference	Stroke	rs10938398	4	A	G	0.0207366	0.0011	0.00217734	0.0094	1.68E-21	0.9056	0.000269	90.70319779
Waist circumference	Stroke	rs11099020	4	T	C	-0.0126065	-0.0096	0.00224865	0.0096	2.07E-08	0.316	9.34E-05	31.43006867
Waist circumference	Stroke	rs12506689	4	A	G	0.0117896	0.0015	0.00223518	0.0097	1.33E-07	0.8737	8.26E-05	27.82102557
Waist circumference	Stroke	rs1383723	4	T	A	-0.017439	-0.0067	0.00261578	0.0113	2.62E-11	0.552	0.000132	44.4468232
Waist circumference	Stroke	rs1472872	4	G	A	-0.0219381	0.012	0.003991	0.0171	3.87E-08	0.4848	8.97E-05	30.21583276
Waist circumference	Stroke	rs1724557	4	A	C	-0.0140573	-9.00E-04	0.00219535	0.0092	1.52E-10	0.9219	0.000122	41.00117371
Waist circumference	Stroke	rs2102278	4	G	A	0.0123714	0.0328	0.00230699	0.0101	8.21E-08	0.00112401	8.54E-05	28.75717856
Waist circumference	Stroke	rs2192527	4	G	A	0.0149708	0.0077	0.00216127	0.0092	4.31E-12	0.3991	0.000143	47.98129515
Waist circumference	Stroke	rs2798304	4	C	T	0.0110971	-0.0033	0.00216567	0.0093	2.99E-07	0.719999	7.80E-05	26.25635516
Waist circumference	Stroke	rs4419475	4	T	A	0.0117043	0.0167	0.00218769	0.0094	8.80E-08	0.0769804	8.50E-05	28.62327523
Waist circumference	Stroke	rs4527444	4	G	A	0.0111772	-0.0035	0.00215952	0.0092	2.27E-07	0.7007	7.96E-05	26.78869657
Waist circumference	Stroke	rs6536575	4	C	T	0.0115685	-0.0222	0.00215288	0.0092	7.73E-08	0.0156401	8.58E-05	28.87449886
Waist circumference	Stroke	rs73213484	4	T	A	-0.0190998	0.0046	0.00311019	0.0127	8.21E-10	0.7168	0.000112	37.71236755
Waist circumference	Stroke	rs7377083	4	A	C	0.0144262	0.0055	0.00218612	0.0098	4.15E-11	0.578	0.000129	43.5467655
Waist circumference	Stroke	rs750090	4	C	T	-0.0125527	0.0145	0.00226957	0.0098	3.19E-08	0.1375	9.09E-05	30.5905394
Waist circumference	Stroke	rs75152244	4	G	A	0.0165384	-0.0051	0.00315871	0.0139	1.64E-07	0.7137	8.14E-05	27.41368844
Waist circumference	Stroke	rs809955	4	A	G	-0.0123432	0.0083	0.00223476	0.0097	3.33E-08	0.3931	9.06E-05	30.50659619
Waist circumference	Stroke	rs12186509	5	G	T	-0.0131437	0.004	0.00245425	0.0104	8.54E-08	0.703399	8.52E-05	28.68122368

Waist circumference	Stroke	rs146311547	5	G	A	0.0177425	0.0018	0.0032254	0.0153	3.78E-08	0.9044	8.99E-05	30.25954996
Waist circumference	Stroke	rs1503527	5	T	C	0.0111228	0.0191	0.00215212	0.0093	2.36E-07	0.0404902	7.93E-05	26.71131626
Waist circumference	Stroke	rs1582931	5	A	G	-0.0146874	-0.0299	0.00217176	0.0092	1.35E-11	0.001191	0.000136	45.73684317
Waist circumference	Stroke	rs1985524	5	C	G	-0.0113177	-0.0178	0.00216006	0.0091	1.61E-07	0.0506198	8.15E-05	27.45267864
Waist circumference	Stroke	rs2126165	5	G	A	-0.0143063	0.0098	0.00215364	0.0092	3.08E-11	0.2863	0.000131	44.12741002
Waist circumference	Stroke	rs2307111	5	C	T	-0.0245649	-0.018	0.00220411	0.0095	7.66E-29	0.0568905	0.000369	124.2119761
Waist circumference	Stroke	rs245775	5	G	A	0.0161351	0.0052	0.0024218	0.0104	2.70E-11	0.6151	0.000132	44.3881222
Waist circumference	Stroke	rs248142	5	C	T	0.0198573	-0.0276	0.00366822	0.016	6.19E-08	0.0848496	8.70E-05	29.30418486
Waist circumference	Stroke	rs254024	5	T	G	0.0140302	0.015	0.00216628	0.0094	9.39E-11	0.1097	0.000125	41.94677182
Waist circumference	Stroke	rs286818	5	A	T	-0.0182155	-0.0325	0.00286927	0.0125	2.18E-10	0.00932911	0.00012	40.30318411
Waist circumference	Stroke	rs34483452	5	A	C	0.0251173	0.014	0.0031711	0.0144	2.37E-15	0.3292	0.000186	62.73732978
Waist circumference	Stroke	rs66637616	5	A	T	0.0118443	0.0078	0.00225343	0.0097	1.47E-07	0.423	8.21E-05	27.62680449
Waist circumference	Stroke	rs67632512	5	A	C	0.0191952	0.0251	0.00338782	0.0146	1.46E-08	0.0867501	9.54E-05	32.10292676
Waist circumference	Stroke	rs67913249	5	G	C	-0.0121729	-0.0019	0.00227292	0.0098	8.53E-08	0.8472	8.52E-05	28.68268533
Waist circumference	Stroke	rs6873229	5	T	C	0.0256501	0.0085	0.00482223	0.023	1.04E-07	0.710101	8.40E-05	28.29321398
Waist circumference	Stroke	rs7442885	5	G	C	-0.0221708	-0.0199	0.00264733	0.0111	5.55E-17	0.0718903	0.000208	70.13689995
Waist circumference	Stroke	rs7728095	5	G	A	0.0143	-4.00E-04	0.00222461	0.0099	1.29E-10	0.9641	0.000123	41.3203798
Waist circumference	Stroke	rs1159974	6	C	T	0.0126505	0.0095	0.0021527	0.0093	4.19E-09	0.3057	0.000103	34.53411734
Waist circumference	Stroke	rs11757278	6	C	T	-0.0140028	-0.0015	0.00233576	0.0099	2.04E-09	0.8825	0.000107	35.93960818
Waist circumference	Stroke	rs1184570	6	T	C	-0.0134818	-0.0145	0.00215343	0.0093	3.84E-10	0.1186	0.000116	39.19532251
Waist circumference	Stroke	rs13210406	6	G	C	-0.0145777	-0.0031	0.00237523	0.01	8.40E-10	0.7552	0.000112	37.66748961
Waist circumference	Stroke	rs1321519	6	G	A	0.0148054	-0.0064	0.00225862	0.0098	5.57E-11	0.513499	0.000128	42.96887187
Waist circumference	Stroke	rs17708311	6	C	G	-0.0254059	-0.001	0.00429829	0.0197	3.41E-09	0.9605	0.000104	34.93636706
Waist circumference	Stroke	rs2183947	6	A	G	-0.0240585	-0.004	0.00257399	0.0104	9.09E-21	0.7053	0.000259	87.36216381
Waist circumference	Stroke	rs2253310	6	G	C	0.0191052	-0.0096	0.00222456	0.0092	8.86E-18	0.2941	0.000219	73.75898454
Waist circumference	Stroke	rs2814943	6	A	G	0.0343785	0.0109	0.00309386	0.0132	1.11E-28	0.4092	0.000367	123.4731512

Waist circumference	Stroke	rs28366156	6	C	T	-0.0212419	0.0342	0.0031838	0.0179	2.53E-11	0.0560402	0.000132	44.51385053
Waist circumference	Stroke	rs34045288	6	T	C	0.0224668	-9.00E-04	0.00227876	0.0096	6.30E-23	0.922	0.000289	97.20425217
Waist circumference	Stroke	rs36007635	6	A	G	-0.0157308	0.024	0.00311724	0.0131	4.50E-07	0.0668005	7.56E-05	25.46602366
Waist circumference	Stroke	rs3757050	6	G	C	-0.011177	-0.0181	0.00222199	0.0095	4.90E-07	0.0565705	7.52E-05	25.3026671
Waist circumference	Stroke	rs4467770	6	A	G	0.0140217	0.0078	0.00243256	0.0105	8.21E-09	0.4562	9.87E-05	33.22570614
Waist circumference	Stroke	rs584170	6	A	G	-0.011424	-0.0058	0.00220623	0.0096	2.24E-07	0.5441	7.96E-05	26.81234614
Waist circumference	Stroke	rs62421910	6	G	A	0.0225431	0.0083	0.0041117	0.0198	4.19E-08	0.6757	8.93E-05	30.05968569
Waist circumference	Stroke	rs72892910	6	T	G	0.0318365	0.0226	0.00286885	0.012	1.31E-28	0.0606401	0.000366	123.1501516
Waist circumference	Stroke	rs7752202	6	T	C	0.0205575	-0.0081	0.00306988	0.0146	2.14E-11	0.580801	0.000133	44.84332419
Waist circumference	Stroke	rs9370243	6	T	G	0.0220827	0.0399	0.00394402	0.0168	2.16E-08	0.0176999	9.31E-05	31.34917584
Waist circumference	Stroke	rs9376507	6	T	A	0.0125447	0.0054	0.00240493	0.0103	1.83E-07	0.6038	8.08E-05	27.20919417
Waist circumference	Stroke	rs9378684	6	T	C	0.0166383	0.0157	0.00270816	0.0112	8.07E-10	0.1627	0.000112	37.74585463
Waist circumference	Stroke	rs9402104	6	A	G	0.0120592	-0.0046	0.00219525	0.0097	3.95E-08	0.6392	8.96E-05	30.17651092
Waist circumference	Stroke	rs9448745	6	T	A	-0.0118233	-0.0147	0.00217383	0.0094	5.36E-08	0.119	8.79E-05	29.5819135
Waist circumference	Stroke	rs9688977	6	C	T	0.0186951	0.0355	0.00304984	0.013	8.80E-10	0.00612802	0.000112	37.57521488
Waist circumference	Stroke	rs10236214	7	T	C	0.0145481	-0.0309	0.00225631	0.0095	1.14E-10	0.00121001	0.000123	41.57334974
Waist circumference	Stroke	rs10237306	7	T	G	0.0133558	0.0104	0.00221471	0.0095	1.64E-09	0.2724	0.000108	36.36688312
Waist circumference	Stroke	rs113852095	7	T	C	-0.0149999	-0.0097	0.00281533	0.0117	9.94E-08	0.4055	8.43E-05	28.38690937
Waist circumference	Stroke	rs11764337	7	T	C	-0.0152814	-0.0148	0.00278852	0.0117	4.25E-08	0.2032	8.92E-05	30.03162001
Waist circumference	Stroke	rs1182199	7	A	C	-0.0160465	5.00E-04	0.002337	0.0116	6.60E-12	0.9681	0.00014	47.14582243
Waist circumference	Stroke	rs12375196	7	A	C	0.0133717	-0.0029	0.00218693	0.0093	9.70E-10	0.7535	0.000111	37.38552506
Waist circumference	Stroke	rs1470749	7	T	G	-0.0135366	0.0042	0.0021534	0.0092	3.26E-10	0.649201	0.000117	39.51570862
Waist circumference	Stroke	rs17149254	7	C	T	-0.0170287	0.0033	0.00279014	0.0158	1.04E-09	0.8349	0.000111	37.24868954
Waist circumference	Stroke	rs17167306	7	C	A	-0.0170305	0.0114	0.00312606	0.0137	5.10E-08	0.4062	8.82E-05	29.6797459
Waist circumference	Stroke	rs1922879	7	A	G	-0.0115729	-0.0059	0.00228302	0.0098	4.00E-07	0.546799	7.63E-05	25.69596717
Waist circumference	Stroke	rs2188306	7	A	G	0.0199256	0.006	0.00391319	0.0167	3.55E-07	0.7199	7.70E-05	25.92751806

Waist circumference	Stroke	rs2289379	7	T	C	-0.0117374	0.0036	0.00220747	0.0094	1.05E-07	0.7002	8.40E-05	28.27184754
Waist circumference	Stroke	rs2404324	7	G	A	-0.0193061	-0.0258	0.00297384	0.0129	8.48E-11	0.0463095	0.000125	42.14576152
Waist circumference	Stroke	rs4718964	7	T	G	0.0146209	0.0232	0.00219068	0.0094	2.49E-11	0.01305	0.000132	44.54411418
Waist circumference	Stroke	rs4722398	7	T	C	0.0184664	0.0073	0.00313148	0.0136	3.70E-09	0.5903	0.000103	34.77484417
Waist circumference	Stroke	rs541577	7	G	A	-0.0121652	-0.0014	0.00222647	0.0095	4.66E-08	0.8856	8.87E-05	29.85415691
Waist circumference	Stroke	rs58862095	7	T	C	-0.0180642	-0.0271	0.0021844	0.0115	1.35E-16	0.0180198	0.000203	68.38693349
Waist circumference	Stroke	rs73068448	7	T	C	-0.0147499	-0.0021	0.00289425	0.0124	3.47E-07	0.8682	7.71E-05	25.97204029
Waist circumference	Stroke	rs10100245	8	A	G	0.0150449	-1.00E-04	0.00216949	0.0092	4.08E-12	0.993	0.000143	48.09094438
Waist circumference	Stroke	rs10957088	8	C	T	0.0163282	0.0185	0.00293621	0.0125	2.68E-08	0.14	9.19E-05	30.9244793
Waist circumference	Stroke	rs12679106	8	T	G	-0.0218821	-0.0073	0.00238599	0.0098	4.71E-20	0.4602	0.00025	84.10866863
Waist circumference	Stroke	rs12680342	8	G	T	-0.0143433	-0.0088	0.00255577	0.0109	2.00E-08	0.4155	9.36E-05	31.49594389
Waist circumference	Stroke	rs13264909	8	T	A	-0.0126861	-0.0056	0.00217545	0.009	5.50E-09	0.5351	0.000101	34.00619563
Waist circumference	Stroke	rs1559900	8	T	C	0.0130192	-0.0032	0.00238216	0.0102	4.62E-08	0.751201	8.87E-05	29.86941669
Waist circumference	Stroke	rs1566085	8	T	G	-0.0127734	-0.0152	0.00217593	0.0092	4.35E-09	0.0986711	0.000102	34.46062778
Waist circumference	Stroke	rs17716502	8	T	C	-0.0178862	0.0068	0.00267351	0.0116	2.23E-11	0.5575	0.000133	44.75819247
Waist circumference	Stroke	rs2725371	8	G	A	-0.017926	0.0129	0.00234606	0.01	2.16E-14	0.1997	0.000173	58.38328862
Waist circumference	Stroke	rs2919389	8	T	C	0.0114175	-0.0049	0.00220486	0.0093	2.24E-07	0.595799	7.96E-05	26.81513595
Waist circumference	Stroke	rs4072917	8	A	G	0.012243	-0.0099	0.00216259	0.009	1.50E-08	0.2723	9.52E-05	32.04994718
Waist circumference	Stroke	rs4623474	8	A	G	0.0179592	-0.0036	0.0033222	0.0143	6.46E-08	0.8012	8.68E-05	29.22284039
Waist circumference	Stroke	rs59104534	8	T	C	0.0122998	0.0043	0.00235384	0.0101	1.74E-07	0.671901	8.11E-05	27.30500258
Waist circumference	Stroke	rs7827410	8	C	A	-0.0147987	-0.013	0.00293537	0.0123	4.62E-07	0.2912	7.55E-05	25.41683267
Waist circumference	Stroke	rs1019240	9	T	A	0.0125924	-0.0015	0.00225438	0.0097	2.33E-08	0.8798	9.27E-05	31.20058777
Waist circumference	Stroke	rs10992841	9	T	C	-0.0136489	0.0073	0.00232451	0.0096	4.32E-09	0.4475	0.000102	34.47723794
Waist circumference	Stroke	rs12001437	9	C	T	0.0112911	0.0046	0.00223129	0.0094	4.19E-07	0.625	7.61E-05	25.60710424
Waist circumference	Stroke	rs12335914	9	C	G	0.0139242	-0.0202	0.0021576	0.0092	1.09E-10	0.0288802	0.000124	41.64843036
Waist circumference	Stroke	rs1411432	9	C	A	0.0164376	-0.0078	0.00278338	0.0122	3.52E-09	0.520701	0.000104	34.87641295

Waist circumference	Stroke	rs16916303	9	G	A	-0.0179697	-0.0203	0.00334806	0.0145	8.00E-08	0.163	8.56E-05	28.80681137
Waist circumference	Stroke	rs1752169	9	A	C	0.0149735	0.0125	0.0024928	0.0103	1.90E-09	0.2255	0.000107	36.08043641
Waist circumference	Stroke	rs2417998	9	G	C	-0.0158889	9.00E-04	0.00236952	0.0099	2.01E-11	0.9282	0.000134	44.96420364
Waist circumference	Stroke	rs2482704	9	T	G	-0.0129521	0.0036	0.00217695	0.0094	2.69E-09	0.7022	0.000105	35.39838668
Waist circumference	Stroke	rs4741546	9	T	C	-0.0154638	-0.0075	0.0022059	0.0093	2.38E-12	0.4232	0.000146	49.1429029
Waist circumference	Stroke	rs10787738	10	T	C	0.0174812	0.0018	0.00250799	0.0108	3.17E-12	0.8671	0.000144	48.58373307
Waist circumference	Stroke	rs10887578	10	C	G	0.0113203	0.0192	0.00216271	0.0092	1.66E-07	0.0363496	8.14E-05	27.39802741
Waist circumference	Stroke	rs10995427	10	A	G	-0.0120931	0.0011	0.00225224	0.0099	7.91E-08	0.9086	8.56E-05	28.83008705
Waist circumference	Stroke	rs11012732	10	G	A	0.021629	-0.0116	0.00228772	0.0098	3.27E-21	0.2364	0.000265	89.38551432
Waist circumference	Stroke	rs11594905	10	A	G	0.0169306	0.002	0.00312358	0.0159	5.96E-08	0.9006	8.73E-05	29.37916387
Waist circumference	Stroke	rs17399739	10	G	A	0.0225668	-0.003	0.00422808	0.0176	9.44E-08	0.8629	8.46E-05	28.48744947
Waist circumference	Stroke	rs2172131	10	C	T	-0.0139997	-0.0093	0.00218023	0.0094	1.35E-10	0.3229	0.000122	41.2318514
Waist circumference	Stroke	rs2439823	10	G	A	0.0158365	-0.0127	0.00216629	0.0093	2.67E-13	0.1713	0.000159	53.44230981
Waist circumference	Stroke	rs2492462	10	G	A	0.0159865	0.0192	0.00285442	0.0123	2.14E-08	0.1194	9.32E-05	31.36685907
Waist circumference	Stroke	rs61871615	10	T	C	-0.0205876	0.0313	0.00391527	0.0214	1.46E-07	0.1432	8.21E-05	27.64954634
Waist circumference	Stroke	rs7094644	10	A	G	0.0144656	0.0088	0.00233716	0.0099	6.05E-10	0.3744	0.000114	38.30857647
Waist circumference	Stroke	rs71495049	10	A	G	0.0228314	0.0304	0.00387652	0.0163	3.87E-09	0.0611604	0.000103	34.68814115
Waist circumference	Stroke	rs10128597	11	A	G	-0.0163094	0.0042	0.00242631	0.0103	1.80E-11	0.683901	0.000134	45.18386653
Waist circumference	Stroke	rs10898330	11	T	C	-0.0114708	0.0041	0.00216356	0.0093	1.15E-07	0.6594	8.35E-05	28.10926748
Waist circumference	Stroke	rs11601136	11	C	T	0.0116305	-0.0083	0.00218558	0.0097	1.03E-07	0.3931	8.41E-05	28.31805074
Waist circumference	Stroke	rs11824092	11	C	T	0.0134159	0.0106	0.0022508	0.0095	2.52E-09	0.2677	0.000106	35.52759521
Waist circumference	Stroke	rs12806052	11	T	C	-0.0188997	0.0018	0.00289848	0.0132	7.01E-11	0.8904	0.000126	42.51764535
Waist circumference	Stroke	rs1782508	11	G	C	-0.0150543	-0.0079	0.00226128	0.0098	2.79E-11	0.4182	0.000132	44.32129583
Waist circumference	Stroke	rs35023999	11	C	A	-0.0114946	-0.0033	0.00215371	0.0091	9.45E-08	0.7181	8.46E-05	28.4848067
Waist circumference	Stroke	rs3802858	11	C	T	-0.0120538	-0.0035	0.00217553	0.0092	3.02E-08	0.7011	9.12E-05	30.69854624
Waist circumference	Stroke	rs58568715	11	G	A	0.0172649	0.023	0.00291834	0.0125	3.30E-09	0.0665595	0.000104	34.99905827

Waist circumference	Stroke	rs59227842	11	G	A	0.0192801	0.0303	0.00234316	0.0099	1.91E-16	0.002168	0.000201	67.70405403
Waist circumference	Stroke	rs61888762	11	G	C	0.0269155	0.0126	0.00230029	0.0099	1.28E-31	0.2016	0.000407	136.9114346
Waist circumference	Stroke	rs61903695	11	G	A	0.0124889	0.0094	0.00246411	0.0107	4.02E-07	0.3818	7.63E-05	25.68787587
Waist circumference	Stroke	rs7117842	11	C	T	0.0114149	0.0051	0.0022369	0.0095	3.35E-07	0.5946	7.73E-05	26.04060581
Waist circumference	Stroke	rs72915955	11	A	G	-0.0149273	-0.0038	0.00293417	0.0129	3.63E-07	0.769899	7.69E-05	25.88165104
Waist circumference	Stroke	rs7925100	11	A	G	0.0137291	2.00E-04	0.00219878	0.0094	4.27E-10	0.9816	0.000116	38.98706846
Waist circumference	Stroke	rs7930006	11	T	C	-0.0133751	-0.0021	0.002166	0.0099	6.62E-10	0.8321	0.000113	38.13091009
Waist circumference	Stroke	rs7948120	11	T	C	-0.0141338	0.0158	0.00246739	0.0108	1.02E-08	0.1443	9.75E-05	32.81272378
Waist circumference	Stroke	rs7952436	11	T	C	-0.0260472	-0.0118	0.00389664	0.0213	2.32E-11	0.578599	0.000133	44.68291738
Waist circumference	Stroke	rs868784	11	A	G	-0.0122956	0.0067	0.00222281	0.0094	3.18E-08	0.480799	9.09E-05	30.59812177
Waist circumference	Stroke	rs12367809	12	T	C	0.0218046	0.0036	0.00223898	0.0095	2.08E-22	0.7051	0.000282	94.84093344
Waist circumference	Stroke	rs12817084	12	C	T	-0.018729	-2.00E-04	0.00349746	0.0149	8.56E-08	0.9919	8.52E-05	28.67633643
Waist circumference	Stroke	rs147786161	12	G	A	0.0118984	-0.0232	0.00218105	0.0095	4.89E-08	0.0147901	8.84E-05	29.76088806
Waist circumference	Stroke	rs1901241	12	G	A	0.0148449	0.0166	0.00294595	0.0126	4.68E-07	0.1857	7.54E-05	25.39240345
Waist circumference	Stroke	rs1904387	12	T	A	0.0136914	0.0063	0.00246018	0.0104	2.62E-08	0.5461	9.20E-05	30.97147938
Waist circumference	Stroke	rs2012464	12	G	A	-0.0116268	-0.0016	0.00227764	0.0095	3.31E-07	0.8637	7.74E-05	26.05854971
Waist circumference	Stroke	rs2242259	12	C	T	-0.0142362	0.0119	0.00216726	0.009	5.08E-11	0.19	0.000128	43.14854248
Waist circumference	Stroke	rs2373980	12	A	T	-0.0117812	0.0118	0.00218603	0.0096	7.08E-08	0.219	8.63E-05	29.04469512
Waist circumference	Stroke	rs2608703	12	A	C	0.014196	0.0061	0.00215852	0.0094	4.82E-11	0.5182	0.000128	43.25335775
Waist circumference	Stroke	rs3764002	12	T	C	-0.0150535	7.00E-04	0.00244769	0.0108	7.75E-10	0.9514	0.000112	37.8235377
Waist circumference	Stroke	rs55726687	12	A	G	0.0179296	0.0079	0.00263446	0.0117	1.01E-11	0.5009	0.000138	46.31887299
Waist circumference	Stroke	rs56362718	12	C	T	0.0151009	0.008	0.00232596	0.0112	8.46E-11	0.4774	0.000125	42.15034946
Waist circumference	Stroke	rs704061	12	C	T	0.0162902	0.0135	0.00216115	0.0094	4.79E-14	0.1512	0.000169	56.81761638
Waist circumference	Stroke	rs76895963	12	G	T	0.0548145	-0.0588	0.00833756	0.0511	4.89E-11	0.2495	0.000128	43.22280717
Waist circumference	Stroke	rs76929617	12	G	A	-0.0430735	-0.0247	0.00556915	0.0413	1.04E-14	0.550201	0.000178	59.8194608
Waist circumference	Stroke	rs894736	12	G	A	0.0175573	-0.0241	0.00224469	0.0096	5.23E-15	0.0119201	0.000182	61.17904822

Waist circumference	Stroke	rs1218824	13	A	G	0.0129214	-0.0159	0.00227409	0.0099	1.33E-08	0.1069	9.59E-05	32.28522704
Waist circumference	Stroke	rs12877270	13	A	G	0.0133426	0.0032	0.00218377	0.0093	9.98E-10	0.7284	0.000111	37.33078667
Waist circumference	Stroke	rs1379828	13	T	C	-0.0166737	0.0026	0.00268642	0.0114	5.42E-10	0.821	0.000114	38.52264888
Waist circumference	Stroke	rs1441264	13	A	G	0.0161052	-0.0092	0.00223532	0.0095	5.82E-13	0.3342	0.000154	51.91021613
Waist circumference	Stroke	rs17060974	13	G	T	0.0139841	0.0039	0.00256015	0.0107	4.71E-08	0.715	8.86E-05	29.83583652
Waist circumference	Stroke	rs1928496	13	T	C	0.0144603	0.0083	0.00245655	0.0107	3.95E-09	0.4386	0.000103	34.65001206
Waist circumference	Stroke	rs2121058	13	C	T	-0.0178087	-0.0131	0.00256163	0.0107	3.61E-12	0.2186	0.000144	48.3316514
Waist circumference	Stroke	rs525101	13	C	T	0.0129213	-0.0042	0.00222956	0.0097	6.82E-09	0.6672	9.98E-05	33.5872225
Waist circumference	Stroke	rs9522279	13	T	C	0.0130818	0.01	0.00218009	0.0092	1.97E-09	0.2786	0.000107	36.00693583
Waist circumference	Stroke	rs9528841	13	A	T	-0.0136429	0.0107	0.00257213	0.0108	1.13E-07	0.3212	8.36E-05	28.13374599
Waist circumference	Stroke	rs10146816	14	G	A	-0.0220638	0.0101	0.0042027	0.0189	1.52E-07	0.5942	8.19E-05	27.56156311
Waist circumference	Stroke	rs12881629	14	G	A	0.0220789	0.0069	0.00390935	0.0177	1.63E-08	0.6964	9.47E-05	31.89670022
Waist circumference	Stroke	rs17115183	14	T	C	0.0113637	0.0172	0.00219929	0.0094	2.38E-07	0.0660693	7.93E-05	26.69774132
Waist circumference	Stroke	rs217671	14	G	A	0.0138369	-0.0178	0.00241684	0.0106	1.03E-08	0.0928004	9.74E-05	32.7779512
Waist circumference	Stroke	rs2370982	14	T	C	0.0205483	0.0146	0.0026361	0.0112	6.46E-15	0.1916	0.00018	60.76143655
Waist circumference	Stroke	rs3803286	14	G	A	-0.0166452	0.0099	0.00227816	0.0096	2.75E-13	0.3014	0.000159	53.38381307
Waist circumference	Stroke	rs4981693	14	A	G	0.0176133	0.0097	0.00256987	0.0107	7.20E-12	0.3643	0.00014	46.97417436
Waist circumference	Stroke	rs61992671	14	G	A	-0.0129318	0.0119	0.00224606	0.0115	8.54E-09	0.3002	9.85E-05	33.14936789
Waist circumference	Stroke	rs6575340	14	A	G	0.0175066	0.0099	0.00224179	0.0095	5.77E-15	0.297	0.000181	60.98369892
Waist circumference	Stroke	rs7154982	14	A	G	-0.0151095	0.0104	0.00242686	0.01	4.79E-10	0.2999	0.000115	38.76240714
Waist circumference	Stroke	rs11636611	15	T	C	0.0125288	0.0051	0.00215496	0.0094	6.11E-09	0.5866	0.0001	33.80185439
Waist circumference	Stroke	rs12102086	15	A	G	-0.0175068	-3.00E-04	0.0026132	0.0112	2.10E-11	0.9822	0.000133	44.88159078
Waist circumference	Stroke	rs138847555	15	C	T	-0.04557	-0.0192	0.00858346	0.0454	1.10E-07	0.6724	8.37E-05	28.18598925
Waist circumference	Stroke	rs17296856	15	C	A	-0.0128729	-0.0045	0.00239446	0.0102	7.62E-08	0.6566	8.58E-05	28.90264697
Waist circumference	Stroke	rs2470167	15	A	G	0.0148673	-0.0165	0.00267827	0.0117	2.84E-08	0.1601	9.15E-05	30.81452634
Waist circumference	Stroke	rs2682909	15	C	G	-0.0116422	-0.007	0.00224186	0.0095	2.07E-07	0.4629	8.01E-05	26.96827294

Waist circumference	Stroke	rs34994596	15	C	T	-0.016193	-0.0163	0.00234759	0.0103	5.29E-12	0.1128	0.000141	47.57843252
Waist circumference	Stroke	rs3784692	15	T	C	0.0195289	0.0162	0.0021979	0.0094	6.41E-19	0.0848594	0.000234	78.94774053
Waist circumference	Stroke	rs56803094	15	G	A	-0.0157525	-0.0073	0.0025732	0.011	9.26E-10	0.5031	0.000111	37.47588444
Waist circumference	Stroke	rs7171864	15	A	G	0.0136184	-0.0019	0.00228283	0.0099	2.44E-09	0.8447	0.000106	35.58811865
Waist circumference	Stroke	rs7183417	15	T	C	0.0113996	0.0132	0.00217352	0.0092	1.57E-07	0.1521	8.17E-05	27.50755207
Waist circumference	Stroke	rs11642015	16	T	C	0.0563414	0.005	0.00219182	0.0094	1.41E-145	0.5974	0.001959	660.7626675
Waist circumference	Stroke	rs11646719	16	G	C	0.0117176	0.0105	0.00228695	0.0098	3.00E-07	0.2842	7.80E-05	26.25209685
Waist circumference	Stroke	rs12103006	16	G	A	0.0154631	0.0095	0.00217666	0.0095	1.21E-12	0.3162	0.00015	50.46751679
Waist circumference	Stroke	rs12926311	16	C	G	-0.0141836	-0.0029	0.00225378	0.0097	3.11E-10	0.767701	0.000118	39.60499006
Waist circumference	Stroke	rs13333747	16	C	T	-0.0230214	-0.0309	0.00280123	0.0128	2.07E-16	0.0161499	0.000201	67.54075702
Waist circumference	Stroke	rs2032912	16	T	G	-0.0173898	-0.0126	0.00219463	0.0093	2.31E-15	0.1785	0.000186	62.78654005
Waist circumference	Stroke	rs2660241	16	C	T	0.0146515	-0.0062	0.00224209	0.0095	6.38E-11	0.513701	0.000127	42.70297139
Waist circumference	Stroke	rs3814883	16	T	C	0.027979	-0.0178	0.00215875	0.0094	2.09E-38	0.05844	0.000499	167.9808117
Waist circumference	Stroke	rs629471	16	G	A	0.0124677	0.0044	0.00237824	0.0103	1.59E-07	0.6707	8.16E-05	27.48282223
Waist circumference	Stroke	rs71396924	16	T	G	0.0140609	0.0105	0.00271593	0.0115	2.25E-07	0.3601	7.96E-05	26.80335106
Waist circumference	Stroke	rs7206608	16	G	C	0.0130663	0.0195	0.00230276	0.01	1.39E-08	0.0509003	9.56E-05	32.19644317
Waist circumference	Stroke	rs72793809	16	T	C	0.0265338	-0.0034	0.00219042	0.0095	9.10E-34	0.7199	0.000436	146.7385118
Waist circumference	Stroke	rs7500458	16	G	A	0.0122029	0.001	0.00237307	0.0104	2.72E-07	0.9215	7.85E-05	26.44265182
Waist circumference	Stroke	rs756717	16	A	G	-0.0114252	0.0114	0.0022219	0.0096	2.72E-07	0.2363	7.85E-05	26.44104434
Waist circumference	Stroke	rs879620	16	T	C	0.0209893	0.0115	0.00221895	0.0097	3.13E-21	0.2331	0.000266	89.47482905
Waist circumference	Stroke	rs11078883	17	G	C	0.0114574	0.0128	0.00226725	0.0096	4.34E-07	0.1851	7.59E-05	25.53720305
Waist circumference	Stroke	rs11150745	17	G	A	-0.0163767	0.0043	0.00230943	0.0099	1.33E-12	0.662101	0.000149	50.28554722
Waist circumference	Stroke	rs11653367	17	G	A	-0.0180212	-0.0169	0.00230317	0.015	5.11E-15	0.2611	0.000182	61.22311508
Waist circumference	Stroke	rs1914888	17	G	A	-0.0121519	-0.0178	0.00215881	0.0101	1.81E-08	0.0788497	9.41E-05	31.68542463
Waist circumference	Stroke	rs2020942	17	T	C	0.0111769	0.02	0.00220343	0.0094	3.93E-07	0.0327703	7.64E-05	25.73026235
Waist circumference	Stroke	rs208015	17	C	T	-0.0315168	0.0056	0.004285	0.0178	1.91E-13	0.7527	0.000161	54.09817059

Waist circumference	Stroke	rs2306593	17	T	C	-0.0169304	0.0256	0.00215553	0.0094	4.03E-15	0.00637602	0.000183	61.69163702
Waist circumference	Stroke	rs35850753	17	T	C	0.0415087	0.0395	0.00778073	0.0331	9.57E-08	0.2335	8.45E-05	28.46017551
Waist circumference	Stroke	rs3826408	17	T	C	0.0124231	0.0101	0.0021584	0.0093	8.64E-09	0.278	9.84E-05	33.12806713
Waist circumference	Stroke	rs4790841	17	T	C	-0.0251653	-0.0087	0.00298062	0.013	3.11E-17	0.5032	0.000212	71.28382603
Waist circumference	Stroke	rs62071997	17	C	T	0.0164525	0.0027	0.00261513	0.0112	3.15E-10	0.8124	0.000118	39.58013155
Waist circumference	Stroke	rs80135947	17	C	A	0.0251166	0.0097	0.00271445	0.0115	2.20E-20	0.4007	0.000254	85.61660561
Waist circumference	Stroke	rs9916444	17	G	C	0.0123828	-0.0047	0.0022766	0.0098	5.36E-08	0.631801	8.79E-05	29.58450138
Waist circumference	Stroke	rs1652376	18	T	G	-0.0194886	-0.0222	0.00215595	0.0091	1.58E-19	0.01521	0.000243	81.71163762
Waist circumference	Stroke	rs1834144	18	A	C	-0.0146621	-0.0105	0.00222893	0.0096	4.77E-11	0.2747	0.000129	43.27125539
Waist circumference	Stroke	rs1942826	18	A	G	0.0206123	0.0045	0.00326444	0.0129	2.72E-10	0.7281	0.000118	39.86901701
Waist circumference	Stroke	rs57636386	18	C	T	-0.0327533	0.0231	0.00389755	0.0164	4.35E-17	0.1593	0.00021	70.61984104
Waist circumference	Stroke	rs66922415	18	G	A	0.0433724	0.002	0.00253565	0.0106	1.45E-65	0.8538	0.000868	292.5824652
Waist circumference	Stroke	rs7239114	18	A	G	0.0126298	0.0157	0.00217368	0.0094	6.24E-09	0.0937001	0.0001	33.75994479
Waist circumference	Stroke	rs8097672	18	T	A	0.0181817	0.0042	0.00306494	0.0128	2.99E-09	0.742899	0.000105	35.19046625
Waist circumference	Stroke	rs10423928	19	A	T	-0.0260287	0.0149	0.00271482	0.0114	9.07E-22	0.1903	0.000273	91.92272193
Waist circumference	Stroke	rs111640872	19	C	G	0.0195885	-0.0079	0.00229116	0.0098	1.24E-17	0.4225	0.000217	73.0956487
Waist circumference	Stroke	rs11666480	19	G	C	0.0154676	-6.00E-04	0.00216606	0.0095	9.29E-13	0.9529	0.000151	50.99233509
Waist circumference	Stroke	rs11878477	19	G	A	-0.0144808	-0.0189	0.00215797	0.0092	1.94E-11	0.0410696	0.000134	45.02920765
Waist circumference	Stroke	rs1964415	19	C	T	0.0125154	-0.0122	0.00234357	0.0104	9.28E-08	0.2389	8.47E-05	28.51895507
Waist circumference	Stroke	rs2903738	19	T	A	-0.0154642	-0.0059	0.00259614	0.0111	2.58E-09	0.5931	0.000105	35.48123259
Waist circumference	Stroke	rs350832	19	A	G	0.016099	0.0326	0.00257337	0.0114	3.95E-10	0.00415002	0.000116	39.13752278
Waist circumference	Stroke	rs35343344	19	A	C	-0.0168531	-0.0252	0.00247495	0.011	9.81E-12	0.0223501	0.000138	46.36889392
Waist circumference	Stroke	rs429358	19	C	T	-0.0241834	0.0127	0.00296137	0.0134	3.19E-16	0.3418	0.000198	66.68825807
Waist circumference	Stroke	rs62120394	19	A	G	0.020784	0.0148	0.00237797	0.0103	2.34E-18	0.15	0.000227	76.39158566
Waist circumference	Stroke	rs7259070	19	C	T	0.017366	-0.0031	0.0022141	0.0113	4.40E-15	0.7819	0.000183	61.51841387
Waist circumference	Stroke	rs1056441	20	C	T	0.0128077	0.0305	0.00229345	0.0101	2.35E-08	0.00257401	9.26E-05	31.18629193

Waist circumference	Stroke	rs11474838	20	G	T	0.0133441	-0.0025	0.00221385	0.0135	1.67E-09	0.8535	0.000108	36.33140492
Waist circumference	Stroke	rs6030803	20	C	T	-0.0176618	-0.0331	0.0032673	0.014	6.46E-08	0.0182398	8.68E-05	29.2207864
Waist circumference	Stroke	rs6096886	20	G	A	-0.0239161	-0.0211	0.00274229	0.0118	2.76E-18	0.0738006	0.000226	76.05958674
Waist circumference	Stroke	rs13047416	21	G	C	-0.0132224	0.0137	0.00222813	0.0096	2.95E-09	0.1543	0.000105	35.21595985
Waist circumference	Stroke	rs76040172	21	A	G	-0.0337348	0.0038	0.00475228	0.0212	1.26E-12	0.8595	0.00015	50.39091421
Waist circumference	Stroke	rs139915	22	T	C	-0.0113399	0.0258	0.00219324	0.0096	2.34E-07	0.00736207	7.94E-05	26.73290369
Waist circumference	Stroke	rs444102	22	C	G	0.0149052	0.0088	0.00285704	0.0123	1.82E-07	0.4734	8.08E-05	27.21717051
Waist circumference	Stroke	rs9610311	22	C	T	0.0136957	0.0167	0.00239488	0.0107	1.07E-08	0.1176	9.71E-05	32.70400605
Waist circumference	Epilepsy	rs11165493	1	A	G	0.0118676	-6.14E-05	0.002282	0.000155965	1.99E-07	0.69	8.03E-05	37.19813504
Waist circumference	Epilepsy	rs11208779	1	C	G	0.0129711	0.000270607	0.00216129	0.000147657	1.96E-09	0.0669993	0.000107	49.53979146
Waist circumference	Epilepsy	rs112566467	1	T	C	0.0181268	0.000260597	0.00264517	0.00018086	7.25E-12	0.15	0.000139	64.58947524
Waist circumference	Epilepsy	rs12060713	1	A	G	0.0122165	-0.000136277	0.00220788	0.000150964	3.15E-08	0.37	9.09E-05	42.10845576
Waist circumference	Epilepsy	rs12128526	1	A	G	0.0119797	1.85E-06	0.00216039	0.000147641	2.94E-08	0.99	9.13E-05	42.29160967
Waist circumference	Epilepsy	rs12725767	1	G	A	0.0118961	-0.000321594	0.00225266	0.000154034	1.29E-07	0.0369999	8.28E-05	38.35699401
Waist circumference	Epilepsy	rs1490382	1	G	A	-0.0113387	-0.000119625	0.00221817	0.000151527	3.19E-07	0.43	7.76E-05	35.93879589
Waist circumference	Epilepsy	rs241461	1	A	T	-0.0181483	-7.36E-05	0.00230559	0.00015776	3.52E-15	0.64	0.000184	85.21859839
Waist circumference	Epilepsy	rs2678204	1	G	T	0.0194317	-0.000125694	0.00227049	0.000155266	1.15E-17	0.42	0.000218	100.7416487
Waist circumference	Epilepsy	rs3028171	1	C	A	0.0142348	0.000148437	0.00267155	0.000182762	9.92E-08	0.42	8.43E-05	39.0483577
Waist circumference	Epilepsy	rs309535	1	G	A	-0.0152186	0.00021889	0.0029032	0.000197833	1.59E-07	0.27	8.16E-05	37.79393758
Waist circumference	Epilepsy	rs3766823	1	A	G	0.0155791	0.000268921	0.00285308	0.000194981	4.75E-08	0.17	8.86E-05	41.00940595
Waist circumference	Epilepsy	rs3935032	1	T	C	-0.014629	-5.22E-05	0.00225489	0.000154263	8.73E-11	0.74	0.000125	57.89022076
Waist circumference	Epilepsy	rs4322261	1	A	G	-0.0183121	-0.000213891	0.00290548	0.000199103	2.93E-10	0.28	0.000118	54.63452468
Waist circumference	Epilepsy	rs4562625	1	G	C	-0.0116097	-0.000171263	0.00220784	0.000151064	1.45E-07	0.26	8.21E-05	38.03062367
Waist circumference	Epilepsy	rs4652839	1	G	C	0.0120158	0.000127341	0.00229333	0.00015668	1.61E-07	0.42	8.15E-05	37.75712559
Waist circumference	Epilepsy	rs4926726	1	A	G	-0.0113354	-0.000197432	0.00215892	0.000147469	1.52E-07	0.18	8.19E-05	37.91641324
Waist circumference	Epilepsy	rs539515	1	C	A	0.0353614	0.000424646	0.00265034	0.00018221	1.35E-40	0.02	0.000529	244.8399976

Waist circumference	Epilepsy	rs588660	1	A	G	0.0144747	-6.46E-05	0.00218549	0.000149248	3.52E-11	0.67	0.00013	60.33205837
Waist circumference	Epilepsy	rs6687953	1	G	A	0.0132664	7.58E-05	0.00220383	0.00015058	1.75E-09	0.61	0.000108	49.8398402
Waist circumference	Epilepsy	rs7531118	1	C	T	0.015718	6.96E-05	0.00216988	0.000148456	4.37E-13	0.64	0.000156	72.16884954
Waist circumference	Epilepsy	rs815163	1	C	T	-0.0135052	-0.000259046	0.00216641	0.000148132	4.55E-10	0.08	0.000115	53.44995992
Waist circumference	Epilepsy	rs10172196	2	A	G	0.0140208	-1.85E-05	0.00234352	0.000160023	2.20E-09	0.91	0.000106	49.2305749
Waist circumference	Epilepsy	rs10803762	2	A	G	0.0144744	0.000190167	0.00231166	0.000157758	3.82E-10	0.23	0.000116	53.92372324
Waist circumference	Epilepsy	rs12619178	2	T	C	-0.0150737	8.44E-05	0.00219213	0.000149966	6.15E-12	0.57	0.00014	65.03299738
Waist circumference	Epilepsy	rs13022337	2	G	A	0.0382185	9.39E-05	0.00284978	0.000194716	5.34E-41	0.630001	0.000534	247.3724067
Waist circumference	Epilepsy	rs13420048	2	A	C	-0.0139516	-0.000158256	0.00224737	0.000153206	5.37E-10	0.3	0.000114	53.00605969
Waist circumference	Epilepsy	rs13427822	2	G	A	-0.0159741	-7.00E-05	0.002443	0.000167284	6.21E-11	0.68	0.000127	58.80476393
Waist circumference	Epilepsy	rs1405261	2	A	T	-0.0113254	0.000231182	0.00217773	0.000148707	1.99E-07	0.12	8.03E-05	37.1985215
Waist circumference	Epilepsy	rs1609303	2	A	T	0.0171103	-3.60E-05	0.002238	0.000153057	2.09E-14	0.81	0.000174	80.39364711
Waist circumference	Epilepsy	rs1881934	2	T	A	0.0121349	4.88E-05	0.00227206	0.000155167	9.25E-08	0.75	8.47E-05	39.23371734
Waist circumference	Epilepsy	rs2015769	2	G	A	-0.0114133	4.85E-05	0.00216238	0.000147821	1.31E-07	0.74	8.27E-05	38.31643377
Waist circumference	Epilepsy	rs2196150	2	T	G	-0.0111685	-0.000128158	0.00220711	0.000151036	4.19E-07	0.4	7.61E-05	35.21829989
Waist circumference	Epilepsy	rs2433733	2	A	G	-0.0168831	0.000375784	0.00230959	0.000157404	2.68E-13	0.017	0.000159	73.49558512
Waist circumference	Epilepsy	rs2861692	2	C	T	-0.0177333	-0.000151324	0.00240824	0.000164595	1.79E-13	0.36	0.000161	74.57722827
Waist circumference	Epilepsy	rs4549080	2	T	C	0.0125556	-3.11E-05	0.00226903	0.000154896	3.14E-08	0.84	9.09E-05	42.11347841
Waist circumference	Epilepsy	rs4670172	2	A	T	0.0116004	0.000249193	0.0022548	0.000154299	2.68E-07	0.11	7.86E-05	36.40462184
Waist circumference	Epilepsy	rs4671328	2	G	T	-0.0163995	-0.000156332	0.00218011	0.000149035	5.39E-14	0.29	0.000168	77.82713573
Waist circumference	Epilepsy	rs6433243	2	C	T	-0.0143354	0.000192306	0.00225296	0.00015409	1.98E-10	0.21	0.00012	55.68514
Waist circumference	Epilepsy	rs6711584	2	A	G	0.0117155	0.000127161	0.00216817	0.00014842	6.54E-08	0.39	8.67E-05	40.15703525
Waist circumference	Epilepsy	rs6739755	2	G	A	-0.016792	-9.19E-05	0.0022029	0.000150684	2.49E-14	0.54	0.000173	79.91750785
Waist circumference	Epilepsy	rs72917544	2	A	G	-0.0168827	2.48E-05	0.00278077	0.000190516	1.27E-09	0.9	0.000109	50.69677284
Waist circumference	Epilepsy	rs73985439	2	C	A	0.014277	-0.000137093	0.00233516	0.000159747	9.73E-10	0.39	0.000111	51.41232929
Waist circumference	Epilepsy	rs76286777	2	C	T	0.0263558	0.000135958	0.00260034	0.00017814	3.87E-24	0.450001	0.000305	141.2923929

Waist circumference	Epilepsy	rs80330591	2	A	G	-0.0166223	-2.49E-05	0.00303827	0.000204537	4.48E-08	0.9	8.89E-05	41.16765684
Waist circumference	Epilepsy	rs1154988	3	A	T	0.0175068	0.0002571	0.00257031	0.000175418	9.70E-12	0.14	0.000138	63.80712489
Waist circumference	Epilepsy	rs11921483	3	A	C	-0.0110359	0.000128193	0.00216076	0.000147831	3.27E-07	0.39	7.75E-05	35.87807199
Waist circumference	Epilepsy	rs13322435	3	G	A	-0.0163963	3.46E-06	0.00220729	0.00015059	1.10E-13	0.98	0.000164	75.89262375
Waist circumference	Epilepsy	rs1454687	3	G	C	-0.0176908	-2.52E-06	0.00215185	0.000147082	2.02E-16	0.99	0.000201	92.9603186
Waist circumference	Epilepsy	rs1515733	3	C	G	0.012473	-0.000173722	0.00214986	0.000147012	6.57E-09	0.24	1E-04	46.29649716
Waist circumference	Epilepsy	rs2016469	3	A	G	0.0123857	0.000334933	0.00224103	0.0001533	3.26E-08	0.0290001	9.07E-05	42.01190933
Waist circumference	Epilepsy	rs2035831	3	C	G	-0.0118235	3.39E-05	0.00229195	0.000156602	2.49E-07	0.83	7.9E-05	36.60230878
Waist circumference	Epilepsy	rs2455821	3	A	C	0.0152548	0.000142359	0.00242133	0.000165692	2.98E-10	0.39	0.000118	54.59234096
Waist circumference	Epilepsy	rs4856407	3	T	C	0.0154749	0.000180273	0.00223286	0.000152792	4.20E-12	0.24	0.000143	66.06316284
Waist circumference	Epilepsy	rs59815219	3	T	C	0.0113077	0.000147044	0.00215144	0.000147069	1.47E-07	0.32	8.21E-05	37.99414932
Waist circumference	Epilepsy	rs62261725	3	G	A	-0.0181763	2.98E-05	0.00229544	0.000157106	2.41E-15	0.85	0.000186	86.23939889
Waist circumference	Epilepsy	rs7610647	3	G	A	-0.0121287	-0.000184992	0.00237559	0.000162167	3.30E-07	0.25	7.74E-05	35.85190015
Waist circumference	Epilepsy	rs7635592	3	T	C	0.0213705	-0.000230124	0.00267244	0.000182222	1.28E-15	0.21	0.00019	87.95085928
Waist circumference	Epilepsy	rs8192675	3	C	T	0.0153014	0.000511183	0.00237273	0.000162021	1.13E-10	0.0016	0.000124	57.1995143
Waist circumference	Epilepsy	rs869400	3	G	T	0.0201061	0.000215151	0.00278235	0.000189641	4.97E-13	0.26	0.000155	71.82221638
Waist circumference	Epilepsy	rs9289630	3	C	G	0.0155282	-0.000174196	0.00221351	0.000151352	2.30E-12	0.25	0.000146	67.68709922
Waist circumference	Epilepsy	rs9814633	3	A	G	0.0117553	-0.00010622	0.0022665	0.000154772	2.14E-07	0.49	7.99E-05	36.99837367
Waist circumference	Epilepsy	rs9843653	3	C	T	0.0209096	-8.32E-07	0.00215278	0.00014717	2.68E-22	1	0.00028	129.7534149
Waist circumference	Epilepsy	rs9849919	3	T	C	-0.0114633	-0.000123413	0.00226642	0.000154916	4.24E-07	0.43	7.6E-05	35.18561738
Waist circumference	Epilepsy	rs9867068	3	G	C	0.0181689	-0.000107895	0.00249685	0.000170812	3.43E-13	0.53	0.000157	72.82810524
Waist circumference	Epilepsy	rs9968060	3	T	C	0.0150834	-4.26E-05	0.00228856	0.000156308	4.38E-11	0.79	0.000129	59.74485864
Waist circumference	Epilepsy	rs10938398	4	A	G	0.0207366	7.96E-05	0.00217734	0.000148763	1.68E-21	0.59	0.000269	124.7524966
Waist circumference	Epilepsy	rs11099020	4	T	C	-0.0126065	0.0001879	0.00224865	0.000153456	2.07E-08	0.22	9.34E-05	43.22868025
Waist circumference	Epilepsy	rs12506689	4	A	G	0.0117896	-0.000107317	0.00223518	0.00015273	1.33E-07	0.48	8.26E-05	38.2648295
Waist circumference	Epilepsy	rs1383723	4	T	A	-0.017439	-0.000149869	0.00261578	0.000178945	2.62E-11	0.4	0.000132	61.13182662

Waist circumference	Epilepsy	rs1724557	4	A	C	-0.0140573	3.52E-05	0.00219535	0.000150066	1.52E-10	0.81	0.000122	56.3927062
Waist circumference	Epilepsy	rs2102278	4	G	A	0.0123714	1.07E-05	0.00230699	0.000157493	8.21E-08	0.95	8.54E-05	39.55240728
Waist circumference	Epilepsy	rs2192527	4	G	A	0.0149708	0.000202317	0.00216127	0.000147598	4.31E-12	0.17	0.000143	65.99311278
Waist circumference	Epilepsy	rs2798304	4	C	T	0.0110971	0.000108715	0.00216567	0.000148136	2.99E-07	0.46	7.8E-05	36.11279357
Waist circumference	Epilepsy	rs4419475	4	T	A	0.0117043	0.000145518	0.00218769	0.000149632	8.80E-08	0.33	8.5E-05	39.36823765
Waist circumference	Epilepsy	rs4527444	4	G	A	0.0111772	-1.93E-05	0.00215952	0.000147678	2.27E-07	0.9	7.96E-05	36.84497195
Waist circumference	Epilepsy	rs6536575	4	C	T	0.0115685	0.000295093	0.00215288	0.000147192	7.73E-08	0.0449997	8.58E-05	39.71376874
Waist circumference	Epilepsy	rs7377083	4	A	C	0.0144262	0.000328724	0.00218612	0.000149602	4.15E-11	0.0280001	0.000129	59.89389402
Waist circumference	Epilepsy	rs750090	4	C	T	-0.0125527	0.000377694	0.00226957	0.000155109	3.19E-08	0.015	9.09E-05	42.07399801
Waist circumference	Epilepsy	rs809955	4	A	G	-0.0123432	1.20E-05	0.00223476	0.000152822	3.33E-08	0.94	9.06E-05	41.95854315
Waist circumference	Epilepsy	rs12186509	5	G	T	-0.0131437	0.000373382	0.00245425	0.000167928	8.54E-08	0.0259998	8.52E-05	39.44793952
Waist circumference	Epilepsy	rs1503527	5	T	C	0.0111228	5.57E-05	0.00215212	0.00014707	2.36E-07	0.709999	7.93E-05	36.73854366
Waist circumference	Epilepsy	rs1582931	5	A	G	-0.0146874	-0.000387408	0.00217176	0.000148502	1.35E-11	0.00909997	0.000136	62.90611038
Waist circumference	Epilepsy	rs1985524	5	C	G	-0.0113177	-0.000162977	0.00216006	0.000147668	1.61E-07	0.27	8.15E-05	37.7582079
Waist circumference	Epilepsy	rs2126165	5	G	A	-0.0143063	-0.000201443	0.00215364	0.00014712	3.08E-11	0.17	0.000131	60.69250812
Waist circumference	Epilepsy	rs2307111	5	C	T	-0.0245649	0.000106659	0.00220411	0.000150484	7.66E-29	0.48	0.000369	170.8402185
Waist circumference	Epilepsy	rs245775	5	G	A	0.0161351	-5.17E-05	0.0024218	0.000165611	2.70E-11	0.75	0.000132	61.05108971
Waist circumference	Epilepsy	rs254024	5	T	G	0.0140302	5.93E-05	0.00216628	0.000148038	9.39E-11	0.69	0.000125	57.69327473
Waist circumference	Epilepsy	rs286818	5	A	T	-0.0182155	2.14E-06	0.00286927	0.000196241	2.18E-10	0.99	0.00012	55.43269655
Waist circumference	Epilepsy	rs66637616	5	A	T	0.0118443	-2.42E-05	0.00225343	0.000154193	1.47E-07	0.88	8.21E-05	37.99769928
Waist circumference	Epilepsy	rs67913249	5	G	C	-0.0121729	2.55E-05	0.00227292	0.000155566	8.53E-08	0.87	8.52E-05	39.44994985
Waist circumference	Epilepsy	rs7442885	5	G	C	-0.0221708	1.34E-05	0.00264733	0.000179547	5.55E-17	0.94	0.000208	96.46576512
Waist circumference	Epilepsy	rs7728095	5	G	A	0.0143	-0.000142839	0.00222461	0.000152112	1.29E-10	0.35	0.000123	56.83173985
Waist circumference	Epilepsy	rs1159974	6	C	T	0.0126505	-9.13E-05	0.0021527	0.000147018	4.19E-09	0.53	0.000103	47.49796547
Waist circumference	Epilepsy	rs11757278	6	C	T	-0.0140028	8.86E-05	0.00233576	0.000159934	2.04E-09	0.58	0.000107	49.43106701
Waist circumference	Epilepsy	rs1184570	6	T	C	-0.0134818	-8.24E-05	0.00215343	0.000147122	3.84E-10	0.58	0.000116	53.90895203

Waist circumference	Epilepsy	rs13210406	6	G	C	-0.0145777	4.20E-05	0.00237523	0.000162168	8.40E-10	0.8	0.000112	51.80758214
Waist circumference	Epilepsy	rs1321519	6	G	A	0.0148054	-0.000108261	0.00225862	0.000154507	5.57E-11	0.48	0.000128	59.09906346
Waist circumference	Epilepsy	rs2183947	6	A	G	-0.0240585	-0.000316687	0.00257399	0.000175725	9.09E-21	0.0719996	0.000259	120.1572636
Waist circumference	Epilepsy	rs2253310	6	G	C	0.0191052	-0.000182916	0.00222456	0.000151937	8.86E-18	0.23	0.000219	101.447553
Waist circumference	Epilepsy	rs2814943	6	A	G	0.0343785	8.01E-05	0.00309386	0.000199058	1.11E-28	0.69	0.000367	169.8240443
Waist circumference	Epilepsy	rs34045288	6	T	C	0.0224668	-0.000114118	0.00227876	0.000155862	6.30E-23	0.46	0.000289	133.693998
Waist circumference	Epilepsy	rs3757050	6	G	C	-0.011177	6.62E-05	0.00222199	0.000152032	4.90E-07	0.66	7.52E-05	34.80109819
Waist circumference	Epilepsy	rs4467770	6	A	G	0.0140217	0.000176657	0.00243256	0.000166516	8.21E-09	0.29	9.87E-05	45.69838654
Waist circumference	Epilepsy	rs584170	6	A	G	-0.011424	-5.72E-05	0.00220623	0.000150625	2.24E-07	0.7	7.96E-05	36.87749938
Waist circumference	Epilepsy	rs72892910	6	T	G	0.0318365	0.000137009	0.00286885	0.00019507	1.31E-28	0.48	0.000366	169.379793
Waist circumference	Epilepsy	rs9376507	6	T	A	0.0125447	8.51E-05	0.00240493	0.000164145	1.83E-07	0.6	8.08E-05	37.42332118
Waist circumference	Epilepsy	rs9378684	6	T	C	0.0166383	-7.30E-05	0.00270816	0.000185277	8.07E-10	0.69	0.000112	51.9153648
Waist circumference	Epilepsy	rs9402104	6	A	G	0.0120592	5.04E-05	0.00219525	0.000149869	3.95E-08	0.74	8.96E-05	41.50454635
Waist circumference	Epilepsy	rs9448745	6	T	A	-0.0118233	0.000173694	0.00217383	0.000148404	5.36E-08	0.24	8.79E-05	40.68674152
Waist circumference	Epilepsy	rs9688977	6	C	T	0.0186951	7.07E-05	0.00304984	0.00019717	8.80E-10	0.719999	0.000112	51.68066818
Waist circumference	Epilepsy	rs10236214	7	T	C	0.0145481	-0.00012984	0.00225631	0.000154146	1.14E-10	0.4	0.000123	57.17967281
Waist circumference	Epilepsy	rs10237306	7	T	G	0.0133558	-2.63E-05	0.00221471	0.000151048	1.64E-09	0.86	0.000108	50.01873774
Waist circumference	Epilepsy	rs10269774	7	A	G	0.0132003	-4.98E-05	0.00230082	0.000156781	9.63E-09	0.75	9.78E-05	45.27192508
Waist circumference	Epilepsy	rs113852095	7	T	C	-0.0149999	-0.000133049	0.00281533	0.000192622	9.94E-08	0.49	8.43E-05	39.04314182
Waist circumference	Epilepsy	rs11764337	7	T	C	-0.0152814	-0.000328384	0.00278852	0.000189462	4.25E-08	0.0830004	8.92E-05	41.30526447
Waist circumference	Epilepsy	rs1182199	7	A	C	-0.0160465	-0.000195758	0.002337	0.000159833	6.60E-12	0.22	0.00014	64.84400987
Waist circumference	Epilepsy	rs12375196	7	A	C	0.0133717	-4.21E-05	0.00218693	0.000149613	9.70E-10	0.780001	0.000111	51.41977022
Waist circumference	Epilepsy	rs1470749	7	T	G	-0.0135366	-9.87E-05	0.0021534	0.000147219	3.26E-10	0.5	0.000117	54.34960868
Waist circumference	Epilepsy	rs17149254	7	C	T	-0.0170287	-0.00013256	0.00279014	0.000190214	1.04E-09	0.49	0.000111	51.23156768
Waist circumference	Epilepsy	rs1922879	7	A	G	-0.0115729	-0.000122573	0.00228302	0.000155964	4.00E-07	0.43	7.63E-05	35.34204015
Waist circumference	Epilepsy	rs2289379	7	T	C	-0.0117374	7.37E-05	0.00220747	0.000150917	1.05E-07	0.630001	8.4E-05	38.88488664

Waist circumference	Epilepsy	rs2404324	7	G	A	-0.0193061	-8.91E-05	0.00297384	0.000203244	8.48E-11	0.66	0.000125	57.96696367
Waist circumference	Epilepsy	rs4718964	7	T	G	0.0146209	-2.96E-05	0.00219068	0.000149747	2.49E-11	0.84	0.000132	61.2656399
Waist circumference	Epilepsy	rs541577	7	G	A	-0.0121652	-2.88E-05	0.00222647	0.000151938	4.66E-08	0.85	8.87E-05	41.06118306
Waist circumference	Epilepsy	rs58862095	7	T	C	-0.0180642	-9.50E-06	0.0021844	0.000149311	1.35E-16	0.95	0.000203	94.05887441
Waist circumference	Epilepsy	rs73068448	7	T	C	-0.0147499	0.000377189	0.00289425	0.000198324	3.47E-07	0.0569994	7.71E-05	35.72174904
Waist circumference	Epilepsy	rs10100245	8	A	G	0.0150449	-0.00013476	0.00216949	0.000148376	4.08E-12	0.36	0.000143	66.1439235
Waist circumference	Epilepsy	rs10957088	8	C	T	0.0163282	0.000197325	0.00293621	0.000200793	2.68E-08	0.33	9.19E-05	42.53329643
Waist circumference	Epilepsy	rs12679106	8	T	G	-0.0218821	0.000129598	0.00238599	0.000162605	4.71E-20	0.43	0.00025	115.6824308
Waist circumference	Epilepsy	rs12680342	8	G	T	-0.0143433	0.000103783	0.00255577	0.000174711	2.00E-08	0.55	9.36E-05	43.31928454
Waist circumference	Epilepsy	rs13264909	8	T	A	-0.0126861	7.94E-05	0.00217545	0.000148764	5.50E-09	0.59	0.000101	46.77186591
Waist circumference	Epilepsy	rs1559900	8	T	C	0.0130192	-0.000455534	0.00238216	0.000162599	4.62E-08	0.0051	8.87E-05	41.08217125
Waist circumference	Epilepsy	rs1566085	8	T	G	-0.0127734	-7.14E-05	0.00217593	0.000148846	4.35E-09	0.630001	0.000102	47.39688848
Waist circumference	Epilepsy	rs17716502	8	T	C	-0.0178862	2.15E-05	0.00267351	0.000183743	2.23E-11	0.91	0.000133	61.56008157
Waist circumference	Epilepsy	rs2725371	8	G	A	-0.017926	-0.000320959	0.00234606	0.000160448	2.16E-14	0.0449997	0.000173	80.29993643
Waist circumference	Epilepsy	rs2919389	8	T	C	0.0114175	1.81E-05	0.00220486	0.000150777	2.24E-07	0.9	7.96E-05	36.88133648
Waist circumference	Epilepsy	rs4072917	8	A	G	0.012243	9.55E-05	0.00216259	0.000147858	1.50E-08	0.52	9.52E-05	44.0812565
Waist circumference	Epilepsy	rs59104534	8	T	C	0.0122998	-7.97E-05	0.00235384	0.000161216	1.74E-07	0.62	8.11E-05	37.55509535
Waist circumference	Epilepsy	rs7827410	8	C	A	-0.0147987	-0.000225813	0.00293537	0.000200658	4.62E-07	0.26	7.55E-05	34.95812065
Waist circumference	Epilepsy	rs1019240	9	T	A	0.0125924	-5.04E-05	0.00225438	0.000153999	2.33E-08	0.74	9.27E-05	42.91305396
Waist circumference	Epilepsy	rs10992841	9	T	C	-0.0136489	0.000157695	0.00232451	0.000158456	4.32E-09	0.32	0.000102	47.41973396
Waist circumference	Epilepsy	rs12001437	9	C	T	0.0112911	2.29E-06	0.00223129	0.000152436	4.19E-07	0.99	7.61E-05	35.21981874
Waist circumference	Epilepsy	rs12335914	9	C	G	0.0139242	-7.24E-05	0.0021576	0.000147465	1.09E-10	0.62	0.000124	57.28293813
Waist circumference	Epilepsy	rs1411432	9	C	A	0.0164376	-4.80E-05	0.00278338	0.000189476	3.52E-09	0.8	0.000104	47.96875628
Waist circumference	Epilepsy	rs1412239	9	G	C	0.019727	-0.000165718	0.00229433	0.000156945	8.13E-18	0.29	0.00022	101.680335
Waist circumference	Epilepsy	rs1752169	9	A	C	0.0149735	-0.000110049	0.0024928	0.000169816	1.90E-09	0.52	0.000107	49.62476109
Waist circumference	Epilepsy	rs2417998	9	G	C	-0.0158889	0.000253541	0.00236952	0.00016241	2.01E-11	0.12	0.000134	61.84342778

Waist circumference	Epilepsy	rs2482704	9	T	G	-0.0129521	0.000238324	0.00217695	0.000148387	2.69E-09	0.11	0.000105	48.68667502
Waist circumference	Epilepsy	rs4741546	9	T	C	-0.0154638	-0.000177157	0.0022059	0.000151003	2.38E-12	0.24	0.000146	67.59077934
Waist circumference	Epilepsy	rs10787738	10	T	C	0.0174812	-3.47E-05	0.00250799	0.000171382	3.17E-12	0.84	0.000144	66.82170137
Waist circumference	Epilepsy	rs10887578	10	C	G	0.0113203	-4.10E-05	0.00216271	0.000147907	1.66E-07	0.780001	8.14E-05	37.68304101
Waist circumference	Epilepsy	rs10995427	10	A	G	-0.0120931	-9.02E-05	0.00225224	0.000153837	7.91E-08	0.56	8.56E-05	39.65268507
Waist circumference	Epilepsy	rs11012732	10	G	A	0.021629	-1.22E-05	0.00228772	0.000156231	3.27E-21	0.94	0.000265	122.9401647
Waist circumference	Epilepsy	rs2172131	10	C	T	-0.0139997	2.26E-05	0.00218023	0.000148991	1.35E-10	0.88	0.000122	56.70997857
Waist circumference	Epilepsy	rs2439823	10	G	A	0.0158365	0.000231618	0.00216629	0.000148035	2.67E-13	0.12	0.000159	73.5041513
Waist circumference	Epilepsy	rs2492462	10	G	A	0.0159865	0.00012519	0.00285442	0.000195433	2.14E-08	0.52	9.32E-05	43.14174225
Waist circumference	Epilepsy	rs7094644	10	A	G	0.0144656	-6.73E-05	0.00233716	0.00016022	6.05E-10	0.67	0.000114	52.68932819
Waist circumference	Epilepsy	rs10128597	11	A	G	-0.0163094	0.000101318	0.00242631	0.000165509	1.80E-11	0.54	0.000134	62.14555047
Waist circumference	Epilepsy	rs10898330	11	T	C	-0.0114708	1.96E-06	0.00216356	0.000147888	1.15E-07	0.99	8.35E-05	38.66127525
Waist circumference	Epilepsy	rs11039266	11	G	T	-0.021681	-0.000265707	0.00239802	0.000163714	1.56E-19	0.1	0.000243	112.4293408
Waist circumference	Epilepsy	rs11601136	11	C	T	0.0116305	0.000240372	0.00218558	0.0001495	1.03E-07	0.11	8.41E-05	38.94843418
Waist circumference	Epilepsy	rs11824092	11	C	T	0.0134159	-9.45E-05	0.0022508	0.000153763	2.52E-09	0.54	0.000106	48.86438746
Waist circumference	Epilepsy	rs12806052	11	T	C	-0.0188997	-8.48E-05	0.00289848	0.000199017	7.01E-11	0.67	0.000126	58.4784499
Waist circumference	Epilepsy	rs1782508	11	G	C	-0.0150543	-7.77E-05	0.00226128	0.000154499	2.79E-11	0.62	0.000132	60.95917721
Waist circumference	Epilepsy	rs35023999	11	C	A	-0.0114946	4.86E-05	0.00215371	0.000147109	9.45E-08	0.74	8.46E-05	39.17778907
Waist circumference	Epilepsy	rs3802858	11	C	T	-0.0120538	-0.000254324	0.00217553	0.000148573	3.02E-08	0.0870001	9.12E-05	42.2225498
Waist circumference	Epilepsy	rs58568715	11	G	A	0.0172649	-0.000146065	0.00291834	0.00019982	3.30E-09	0.46	0.000104	48.13744173
Waist circumference	Epilepsy	rs59227842	11	G	A	0.0192801	0.000122227	0.00234316	0.000160121	1.91E-16	0.450001	0.000201	93.11964712
Waist circumference	Epilepsy	rs61888762	11	G	C	0.0269155	-8.21E-05	0.00230029	0.000157534	1.28E-31	0.6	0.000407	188.3069582
Waist circumference	Epilepsy	rs61903695	11	G	A	0.0124889	-0.00010916	0.00246411	0.000168908	4.02E-07	0.52	7.63E-05	35.33091143
Waist circumference	Epilepsy	rs7117842	11	C	T	0.0114149	-7.83E-05	0.0022369	0.000153074	3.35E-07	0.61	7.73E-05	35.81605353
Waist circumference	Epilepsy	rs72915955	11	A	G	-0.0149273	-0.000146607	0.00293417	0.000201238	3.63E-07	0.47	7.69E-05	35.59742835
Waist circumference	Epilepsy	rs7925100	11	A	G	0.0137291	-0.000312957	0.00219878	0.000150378	4.27E-10	0.0369999	0.000116	53.62252098

Waist circumference	Epilepsy	rs7930006	11	T	C	-0.0133751	-7.30E-05	0.002166	0.000148026	6.62E-10	0.62	0.000113	52.44496718
Waist circumference	Epilepsy	rs7948120	11	T	C	-0.0141338	-0.000377525	0.00246739	0.000168401	1.02E-08	0.025	9.75E-05	45.1303737
Waist circumference	Epilepsy	rs868784	11	A	G	-0.0122956	0.000130972	0.00222281	0.000151877	3.18E-08	0.39	9.09E-05	42.08442674
Waist circumference	Epilepsy	rs12367809	12	T	C	0.0218046	-0.000237103	0.00223898	0.000152886	2.08E-22	0.12	0.000282	130.4435071
Waist circumference	Epilepsy	rs147786161	12	G	A	0.0118984	9.08E-05	0.00218105	0.000148992	4.89E-08	0.54	8.84E-05	40.93290178
Waist circumference	Epilepsy	rs1901241	12	G	A	0.0148449	5.68E-05	0.00294595	0.000200989	4.68E-07	0.780001	7.54E-05	34.92452088
Waist circumference	Epilepsy	rs1904387	12	T	A	0.0136914	0.00013496	0.00246018	0.00016773	2.62E-08	0.42	9.2E-05	42.59793999
Waist circumference	Epilepsy	rs2012464	12	G	A	-0.0116268	0.000152809	0.00227764	0.000155727	3.31E-07	0.33	7.74E-05	35.84073344
Waist circumference	Epilepsy	rs2242259	12	C	T	-0.0142362	-6.84E-05	0.00216726	0.000148238	5.08E-11	0.64	0.000128	59.34618108
Waist circumference	Epilepsy	rs2373980	12	A	T	-0.0117812	-0.000168466	0.00218603	0.000149544	7.08E-08	0.26	8.63E-05	39.9478554
Waist circumference	Epilepsy	rs2608703	12	A	C	0.014196	5.37E-05	0.00215852	0.000147588	4.82E-11	0.719999	0.000128	59.49034321
Waist circumference	Epilepsy	rs3764002	12	T	C	-0.0150535	-0.000169781	0.00244769	0.00016721	7.75E-10	0.31	0.000112	52.02220951
Waist circumference	Epilepsy	rs55726687	12	A	G	0.0179296	0.00027298	0.00263446	0.000180378	1.01E-11	0.13	0.000138	63.70662983
Waist circumference	Epilepsy	rs56362718	12	C	T	0.0151009	-0.000212445	0.00232596	0.000159329	8.46E-11	0.18	0.000125	57.97327389
Waist circumference	Epilepsy	rs704061	12	C	T	0.0162902	0.000152093	0.00216115	0.000147618	4.79E-14	0.3	0.000169	78.14652259
Waist circumference	Epilepsy	rs894736	12	G	A	0.0175573	-7.20E-06	0.00224469	0.000153485	5.23E-15	0.96	0.000182	84.14520317
Waist circumference	Epilepsy	rs1218824	13	A	G	0.0129214	-0.000199161	0.00227409	0.000155443	1.33E-08	0.2	9.59E-05	44.40485865
Waist circumference	Epilepsy	rs12877270	13	A	G	0.0133426	0.000120246	0.00218377	0.000149051	9.98E-10	0.42	0.000111	51.34448345
Waist circumference	Epilepsy	rs1379828	13	T	C	-0.0166737	0.000233912	0.00268642	0.000183686	5.42E-10	0.2	0.000114	52.98376178
Waist circumference	Epilepsy	rs1441264	13	A	G	0.0161052	0.000208039	0.00223532	0.000152869	5.82E-13	0.17	0.000154	71.39692116
Waist circumference	Epilepsy	rs17060974	13	G	T	0.0139841	7.48E-05	0.00256015	0.000174713	4.71E-08	0.67	8.86E-05	41.03598534
Waist circumference	Epilepsy	rs1928496	13	T	C	0.0144603	-0.000110134	0.00245655	0.000168158	3.95E-09	0.51	0.000103	47.65736619
Waist circumference	Epilepsy	rs2121058	13	C	T	-0.0178087	1.57E-05	0.00256163	0.000175466	3.61E-12	0.93	0.000144	66.47499013
Waist circumference	Epilepsy	rs525101	13	C	T	0.0129213	-3.04E-05	0.00222956	0.0001525	6.82E-09	0.84	9.98E-05	46.19561342
Waist circumference	Epilepsy	rs9522279	13	T	C	0.0130818	-5.28E-05	0.00218009	0.000149088	1.97E-09	0.719999	0.000107	49.52366895
Waist circumference	Epilepsy	rs9528841	13	A	T	-0.0136429	7.99E-05	0.00257213	0.000175774	1.13E-07	0.649999	8.36E-05	38.69494281

Waist circumference	Epilepsy	rs17115183	14	T	C	0.0113637	0.000168074	0.00219929	0.000150377	2.38E-07	0.26	7.93E-05	36.71987278
Waist circumference	Epilepsy	rs217671	14	G	A	0.0138369	-0.000192232	0.00241684	0.000165218	1.03E-08	0.24	9.74E-05	45.08254775
Waist circumference	Epilepsy	rs2370982	14	T	C	0.0205483	-0.000273715	0.0026361	0.000180413	6.46E-15	0.13	0.00018	83.57082322
Waist circumference	Epilepsy	rs3803286	14	G	A	-0.0166452	-2.35E-05	0.00227816	0.000155807	2.75E-13	0.88	0.000159	73.42369532
Waist circumference	Epilepsy	rs4981693	14	A	G	0.0176133	0.000108562	0.00256987	0.000175693	7.20E-12	0.54	0.00014	64.60792641
Waist circumference	Epilepsy	rs61992671	14	G	A	-0.0129318	-3.82E-05	0.00224606	0.000153668	8.54E-09	0.8	9.85E-05	45.59339148
Waist circumference	Epilepsy	rs6575340	14	A	G	0.0175066	0.000228217	0.00224179	0.000153122	5.77E-15	0.14	0.000181	83.8765212
Waist circumference	Epilepsy	rs7154982	14	A	G	-0.0151095	5.56E-05	0.00242686	0.000165681	4.79E-10	0.74	0.000115	53.31352348
Waist circumference	Epilepsy	rs11636611	15	T	C	0.0125288	-3.93E-05	0.00215496	0.000147156	6.11E-09	0.79	0.0001	46.49081651
Waist circumference	Epilepsy	rs12102086	15	A	G	-0.0175068	-1.22E-05	0.0026132	0.000179215	2.10E-11	0.95	0.000133	61.72980268
Waist circumference	Epilepsy	rs17296856	15	C	A	-0.0128729	5.49E-05	0.00239446	0.000163847	7.62E-08	0.74	8.58E-05	39.75248344
Waist circumference	Epilepsy	rs2470167	15	A	G	0.0148673	-0.000417626	0.00267827	0.000183068	2.84E-08	0.0230001	9.15E-05	42.38206796
Waist circumference	Epilepsy	rs2682909	15	C	G	-0.0116422	0.000227256	0.00224186	0.000152916	2.07E-07	0.14	8.01E-05	37.09195994
Waist circumference	Epilepsy	rs34994596	15	C	T	-0.016193	0.000133732	0.00234759	0.00016076	5.29E-12	0.41	0.000141	65.43901853
Waist circumference	Epilepsy	rs3784692	15	T	C	0.0195289	-0.000163851	0.0021979	0.000149925	6.41E-19	0.27	0.000234	108.584129
Waist circumference	Epilepsy	rs56803094	15	G	A	-0.0157525	0.000207226	0.0025732	0.000176136	9.26E-10	0.24	0.000111	51.54404983
Waist circumference	Epilepsy	rs7171864	15	A	G	0.0136184	0.000282441	0.00228283	0.000155861	2.44E-09	0.0700003	0.000106	48.94763095
Waist circumference	Epilepsy	rs7183417	15	T	C	0.0113996	0.000142301	0.00217352	0.000148518	1.57E-07	0.34	8.17E-05	37.8336804
Waist circumference	Epilepsy	rs11642015	16	T	C	0.0563414	-0.000156379	0.00219182	0.000149815	1.41E-145	0.3	0.001959	908.8080073
Waist circumference	Epilepsy	rs11646719	16	G	C	0.0117176	0.000512783	0.00228695	0.000156153	3.00E-07	0.001	7.8E-05	36.10693673
Waist circumference	Epilepsy	rs12103006	16	G	A	0.0154631	0.000325778	0.00217666	0.000148576	1.21E-12	0.0280001	0.00015	69.41264333
Waist circumference	Epilepsy	rs12926311	16	C	G	-0.0141836	1.89E-05	0.00225378	0.000154266	3.11E-10	0.9	0.000118	54.4724057
Waist circumference	Epilepsy	rs13333747	16	C	T	-0.0230214	-0.000293301	0.00280123	0.000191181	2.07E-16	0.12	0.000201	92.89504964
Waist circumference	Epilepsy	rs2032912	16	T	G	-0.0173898	-0.000361072	0.00219463	0.000149984	2.31E-15	0.016	0.000186	86.35613535
Waist circumference	Epilepsy	rs2660241	16	C	T	0.0146515	0.000137275	0.00224209	0.000152896	6.38E-11	0.37	0.000127	58.73334594
Waist circumference	Epilepsy	rs3814883	16	T	C	0.027979	6.14E-05	0.00215875	0.000147552	2.09E-38	0.68	0.000499	231.039546

Waist circumference	Epilepsy	rs629471	16	G	A	0.0124677	-0.000240097	0.00237824	0.000162266	1.59E-07	0.14	8.16E-05	37.79966717
Waist circumference	Epilepsy	rs71396924	16	T	G	0.0140609	5.56E-05	0.00271593	0.000185682	2.25E-07	0.760001	7.96E-05	36.86512762
Waist circumference	Epilepsy	rs7206608	16	G	C	0.0130663	-0.00035873	0.00230276	0.000157496	1.39E-08	0.0230001	9.56E-05	44.28274599
Waist circumference	Epilepsy	rs72793809	16	T	C	0.0265338	2.97E-05	0.00219042	0.000149895	9.10E-34	0.84	0.000436	201.8230464
Waist circumference	Epilepsy	rs7500458	16	G	A	0.0122029	-5.25E-05	0.00237307	0.000162101	2.72E-07	0.75	7.85E-05	36.3690246
Waist circumference	Epilepsy	rs756717	16	A	G	-0.0114252	-9.94E-05	0.0022219	0.000151955	2.72E-07	0.51	7.85E-05	36.36681369
Waist circumference	Epilepsy	rs879620	16	T	C	0.0209893	1.65E-05	0.00221895	0.000151404	3.13E-21	0.91	0.000266	123.0630075
Waist circumference	Epilepsy	rs11078883	17	G	C	0.0114574	0.000233667	0.00226725	0.000155078	4.34E-07	0.13	7.59E-05	35.12367717
Waist circumference	Epilepsy	rs11150745	17	G	A	-0.0163767	6.84E-05	0.00230943	0.000158105	1.33E-12	0.67	0.000149	69.16236375
Waist circumference	Epilepsy	rs11653367	17	G	A	-0.0180212	-6.39E-05	0.00230317	0.000157356	5.11E-15	0.68	0.000182	84.20581239
Waist circumference	Epilepsy	rs1914888	17	G	A	-0.0121519	-0.000125838	0.00215881	0.000147414	1.81E-08	0.39	9.41E-05	43.57989493
Waist circumference	Epilepsy	rs2020942	17	T	C	0.0111769	-5.91E-05	0.00220343	0.000150691	3.93E-07	0.69	7.64E-05	35.38920948
Waist circumference	Epilepsy	rs2306593	17	T	C	-0.0169304	-2.25E-05	0.00215553	0.000147361	4.03E-15	0.88	0.000183	84.85021395
Waist circumference	Epilepsy	rs3826408	17	T	C	0.0124231	2.35E-05	0.0021584	0.000147418	8.64E-09	0.87	9.84E-05	45.56409458
Waist circumference	Epilepsy	rs4790841	17	T	C	-0.0251653	-0.000169349	0.00298062	0.0002042	3.11E-17	0.41	0.000212	98.04323863
Waist circumference	Epilepsy	rs62071997	17	C	T	0.0164525	-2.71E-05	0.00261513	0.000178811	3.15E-10	0.88	0.000118	54.43821549
Waist circumference	Epilepsy	rs80135947	17	C	A	0.0251166	0.00010957	0.00271445	0.000185505	2.20E-20	0.55	0.000254	117.7564359
Waist circumference	Epilepsy	rs9916444	17	G	C	0.0123828	0.00015618	0.0022766	0.000155162	5.36E-08	0.31	8.79E-05	40.69030087
Waist circumference	Epilepsy	rs1652376	18	T	G	-0.0194886	-6.85E-05	0.00215595	0.000147423	1.58E-19	0.64	0.000243	112.3855723
Waist circumference	Epilepsy	rs1834144	18	A	C	-0.0146621	-0.000112358	0.00222893	0.000152369	4.77E-11	0.46	0.000129	59.51495949
Waist circumference	Epilepsy	rs66922415	18	G	A	0.0433724	-0.000126746	0.00253565	0.000173739	1.45E-65	0.47	0.000868	402.4157239
Waist circumference	Epilepsy	rs7239114	18	A	G	0.0126298	0.000320622	0.00217368	0.000148642	6.24E-09	0.0309999	0.0001	46.43317436
Waist circumference	Epilepsy	rs8097672	18	T	A	0.0181817	0.000169064	0.00306494	0.000206039	2.99E-09	0.41	0.000105	48.40070283
Waist circumference	Epilepsy	rs10423928	19	A	T	-0.0260287	0.00028244	0.00271482	0.000185409	9.07E-22	0.13	0.000273	126.429821
Waist circumference	Epilepsy	rs111640872	19	C	G	0.0195885	0.000188636	0.00229116	0.000156718	1.24E-17	0.23	0.000217	100.5352059
Waist circumference	Epilepsy	rs11666480	19	G	C	0.0154676	-0.00019051	0.00216606	0.000148236	9.29E-13	0.2	0.000151	70.13447448

Waist circumference	Epilepsy	rs11878477	19	G	A	-0.0144808	-0.000162083	0.00215797	0.000147636	1.94E-11	0.27	0.000134	61.93283381
Waist circumference	Epilepsy	rs1964415	19	C	T	0.0125154	0.000213464	0.00234357	0.000160041	9.28E-08	0.18	8.47E-05	39.22475649
Waist circumference	Epilepsy	rs2903738	19	T	A	-0.0154642	3.45E-05	0.00259614	0.000176977	2.58E-09	0.85	0.000105	48.80062066
Waist circumference	Epilepsy	rs350832	19	A	G	0.016099	0.000236873	0.00257337	0.000175791	3.95E-10	0.18	0.000116	53.82945471
Waist circumference	Epilepsy	rs35343344	19	A	C	-0.0168531	-0.000333584	0.00247495	0.000169235	9.81E-12	0.0490004	0.000138	63.77542823
Waist circumference	Epilepsy	rs429358	19	C	T	-0.0241834	9.21E-05	0.00296137	0.000203617	3.19E-16	0.649999	0.000198	91.722529
Waist circumference	Epilepsy	rs62120394	19	A	G	0.020784	2.08E-05	0.00237797	0.000162195	2.34E-18	0.9	0.000227	105.0684128
Waist circumference	Epilepsy	rs7259070	19	C	T	0.017366	-0.000127596	0.0022141	0.000151102	4.40E-15	0.4	0.000183	84.61196413
Waist circumference	Epilepsy	rs1056441	20	C	T	0.0128077	-0.000127815	0.00229345	0.000157012	2.35E-08	0.42	9.26E-05	42.89339155
Waist circumference	Epilepsy	rs11474838	20	G	T	0.0133441	0.000101929	0.00221385	0.000151341	1.67E-09	0.5	0.000108	49.9699413
Waist circumference	Epilepsy	rs6096886	20	G	A	-0.0239161	-0.000102028	0.00274229	0.000187309	2.76E-18	0.59	0.000226	104.611784
Waist circumference	Epilepsy	rs13047416	21	G	C	-0.0132224	0.000200785	0.00222813	0.000152026	2.95E-09	0.19	0.000105	48.43576654
Waist circumference	Epilepsy	rs139915	22	T	C	-0.0113399	-0.000205412	0.00219324	0.000150007	2.34E-07	0.17	7.94E-05	36.76823484
Waist circumference	Epilepsy	rs444102	22	C	G	0.0149052	0.00020852	0.00285704	0.000195245	1.82E-07	0.29	8.08E-05	37.43429179
Waist circumference	Epilepsy	rs9610311	22	C	T	0.0136957	-8.28E-05	0.00239488	0.000163679	1.07E-08	0.61	9.71E-05	44.98084415

Table S2. Cochran's Q test for heterogeneity among MR analyses.

Exposure	Outcome	MR-IVW			MR-Egger		
		Q	Q-df	Q-*P*val	Q	Q-df	Q-*P*val
Weight	Falls	32.58124	19	0.02685342	31.32371	18	0.02640084
Height	Falls	684.736	430	6.02E-14	685.9213	431	6.10E-14
Sitting height	Falls	785.0767	511	6.20E-14	784.9385	510	5.10E-14
Waist-hip ratio	Falls	21.79952	21	4.11E-01	21.17223	20	3.87E-01
Hip circumference	Falls	633.5493	359	1.76E-17	633.0813	358	1.46E-17
Rheumatoid Arthritis	Falls	0.6255656	2	0.7314088	0.6182633	1	0.4316934
Sleeplessness	Falls	108.0201	73	4.86E-03	106.1411	72	5.49E-03
BMI	Falls	169.8249	109	1.73E-04	169.7736	108	1.37E-04
Waist circumference	Falls	629.1879	301	2.49E-25	627.1105	300	2.97E-25
Osteoporosis	Falls	10.54659	11	0.4819917	10.36752	10	0.4088625

Type 2 diabetes	Falls	328.423	151	3.42E-15	325.7753	150	4.77E-15
Cataract	Falls	12.16699	8	1.44E-01	10.3519	7	1.69E-01
Parkinson's disease	Falls	50.85785	25	1.67E-03	48.86399	24	1.97E-03
Alzheimer's disease	Falls	1.660175	4	7.98E-01	1.321548	3	7.24E-01
Atherosclerotic heart disease	Falls	45.84745	33	6.77E-02	42.13948	32	1.08E-01
Depression	Falls	2.8881052	4	5.77E-01	0.6942453	3	8.75E-01
Glaucoma	Falls	28.45807	22	1.61E-01	27.70628	21	1.49E-01
High blood pressure	Falls	306.8051	200	1.75E-06	303.6043	199	2.50E-06
Bipolar disorder	Falls	1.53083	2	4.65E-01	1.538022	3	6.74E-01
Stroke	Falls	24.65518	21	2.62E-01	24.46841	20	2.23E-01
BMI	Stroke	153.8308	111	0.004471057	151.8324	110	0.00509223
Hip circumference	Stroke	551.018	362	5.27E-10	550.4747	361	4.67E-10
Waist circumference	Stroke	417.6409	303	1.34E-05	416.7676	302	1.28E-05

Waist circumfernece	Epilepsy	283.1332	255	0.1089843	281.7033	254	0.1119131
Hip circumference	Fracture	563.6026	362	5.48E-11	563.5804	362	5.48E-11
Sleeplessness	Fracture	131.4295	76	8.28E-05	131.0044	75	6.77E-05
Osteoporosis	Fracture	1.0036783	3	0.8003619	0.7959273	2	0.6716864
Falls	Fracture	11.83312	14	0.6197022	11.71142	13	0.5514373
Falls	Epilepsy	8.886311	13	0.7814747	7.853142	12	0.796499
Falls	Sever stress	19.99655	15	0.172065	19.84731	14	0.1350292
Falls	Patient death	7.673498	9	0.5673547	7.556208	8	0.4779747
Falls	Headache	14.50731	14	0.4374484	13.13487	13	0.4374484
Falls	Anxiety disorder	12.26555	9	0.1987496	12.26504	8	0.1397643
Falls	Stroke	11.518639	16	0.7763986	9.746017	15	0.8354047

The Q statistic has a chi-squared distribution on N-1 degrees of freedom (df) under the null hypothesis that all genetic variants are valid IVs and the same causal effect is identified by all variants. N is the number of genetic variants.

Table S3. Causal relationship of exposures on falls without outliers.

		MR-Egger with outliers		MR-Weighted median with outliers		MR-IVW with outliers	
Exposure	Outcome	OR(95%CI)	*P*	OR(95%CI)	*P*	OR(95%CI)	*P*
Hip circumference	Falls	1.043(1.013–1.074)	4.54E-03	1.046(1.034–1.059)	3.09E-13	1.048(1.038–1.058)	1.51E-21
Sleeplessness	Falls	1.212(1.022–1.438)	3.07E-02	1.129(1.077–1.183)	4.00E-07	1.155(1.113–1.198)	2.54E-14

Table S4. Causal relationships of exposures on falls estimated by multivariable MR.

Esposure	N.SNPs	MVMR–IVW	
		OR(95%CI)	*P*
BMI	37	1.016(0.982–1.052)	0.350
Waist circumference	123	1.009(0.963–1.058)	0.705
Hip circumference	164	1.033(0.999–1.070)	0.061
Osteoporosis	4	1.602(0.964–2.662)	0.069
Sleeplessness	16	1.139(1.063–1.220)	<0.001

Table S5. Causal relationships of exposures on outcomes estimated by using MR-Egger, MR-Weighted median, and MR-IVW.

Exposure	Outcome	MR–Egger		MR–Weighted median		MR–IVW		MR–Egger Intercept	Intercept *P* Value
		OR(95%CI)	*P*	OR(95%CI)	*P*	OR(95%CI)	*P*		
Hip circumference	Fracture	1.008(0.994–1.021)	0.261	1.004(0.998–1.010)	0.208	1.007(1.002–1.012)	0.003	0	0.905
Waist circumference		0.999(0.982–1.016)	0.922	0.999(0.992–1.007)	0.920	1.005(0.999–1.010)	0.095	0.0001	0.491
Rheumatoid Arthritis		0.381(0.048–3.032)	0.529	0.888(0.533–1.479)	0.649	0.964(0.565–1.646)	0.894	0.0016	0.529
Sleeplessness		1.014(0.947–1.086)	0.698	1.036(1.011–1.061)	0.005	1.031(1.009–1.052)	0.004	0.0002	0.623
BMI		0.992(0.976–1.007)	0.283	0.992(0.983–1.001)	0.073	0.998(0.992–1.004)	0.527	0.0002	0.371
Osteoporosis		3.162(0.486–5.838)	0.488	1.682(1.093–2.586)	0.018	1.706(1.195–2.437)	0.003	-0.0014	0.693
Hip circumference	Stroke	1.050(0.857–1.286)	0.639	1.097(0.992–1.214)	0.070	1.112(1.037–1.193)	0.003	0.0012	0.551
Waist circumference		1.123(0.869–1.450)	0.377	1.164(1.026–1.322)	0.019	1.238(1.139–1.347)	<0.001	0.0018	0.427
Sleeplessness		0.777(0.307–1.966)	0.596	1.399(0.968–2.023)	0.074	1.250(0.950–1.646)	0.111	0.0051	0.296
BMI		0.970(0.778–1.209)	0.787	1.038(0.909–1.187)	0.581	1.097(1.002–1.202)	0.045	0.0034	0.231
Osteoporosis		0.021(0–2.842380e+08)	0.751	0.309(0.007–1.351)	0.543	1.093(0.532–2.245)	0.954	0.0082	0.744
Hip circumference	Epilepsy	0.999(0.995–1.002)	0.396	0.999(0.998–1.001)	0.914	1.001(0.999–1.002)	0.280	0	0.215
Waist circumference		0.999(0.996–1.002)	0.734	1.001(0.999–1.003)	0.414	1.002(1.000–1.003)	0.018	0	0.257
BMI		0.998(0.995–1.001)	0.272	0.999(0.997–1.002)	0.602	0.999(0.996–1.001)	0.827	0	0.269
Rheumatoid Arthritis		0.778(0.508–1.191)	0.454	0.898(0.795–1.015)	0.085	0.899(0.801–1.008)	0.067	0.0003	0.615
Sleeplessness		0.999(0.976–1.025)	0.997	1.003(0.997–1.008)	0.354	1.003(0.999–1.007)	0.200	0	0.827
Osteoporosis		1.403(0.841–-2.339)	0.227	1.007(0.942–1.076)	0.844	1.018(0.956–1.083)	0.581	-0.0007	0.247

(A)

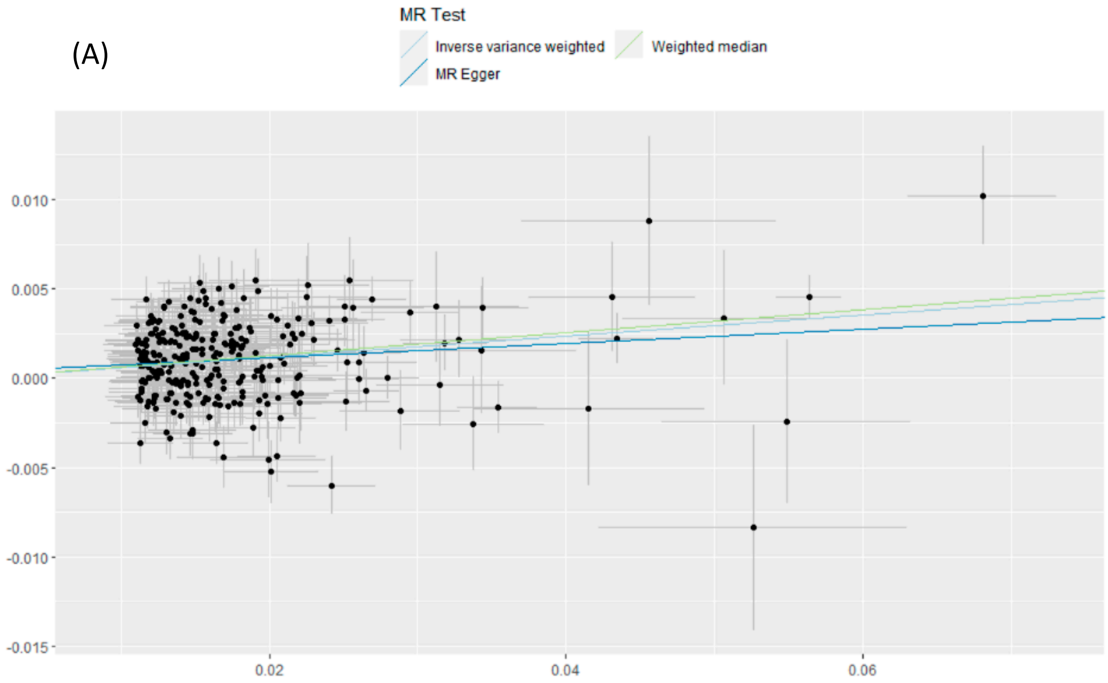

MR Test
Inverse variance weighted
Weighted median
MR Egger
0.010
0.005
0.000
-0.005
-0.010
-0.015
0.02
0.04
0.06

(B)

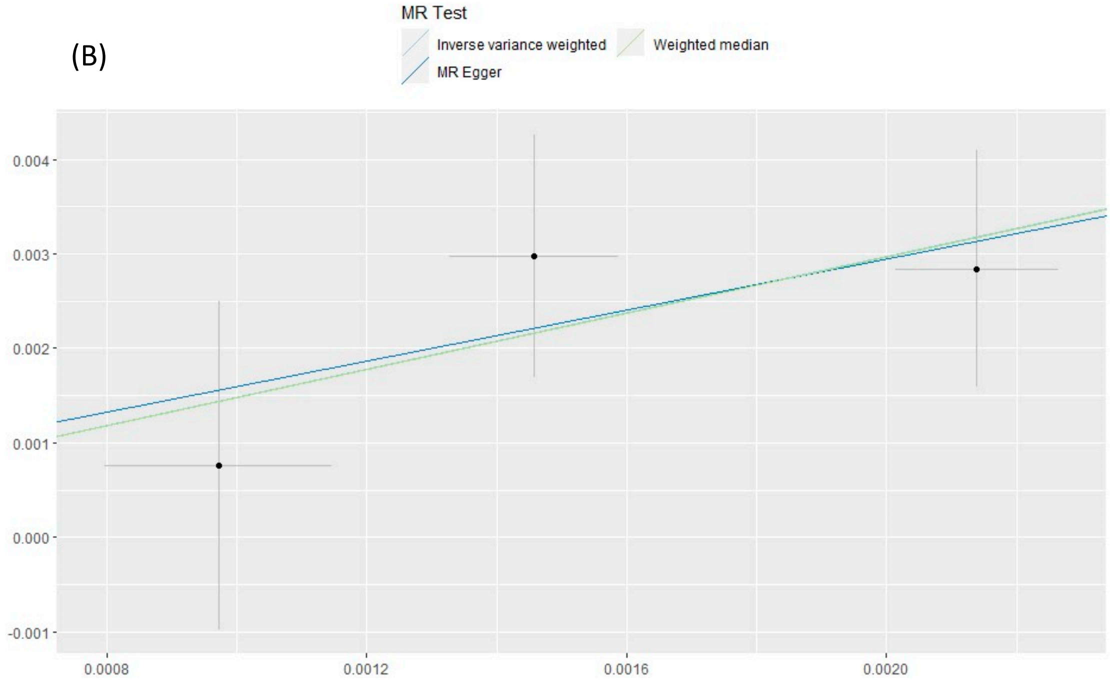

MR Test
Inverse variance weighted
Weighted median
MR Egger
0.004
0.003
0.002
0.001
0.000
-0.001
0.0008
0.0012
0.0016
0.0020

(C)

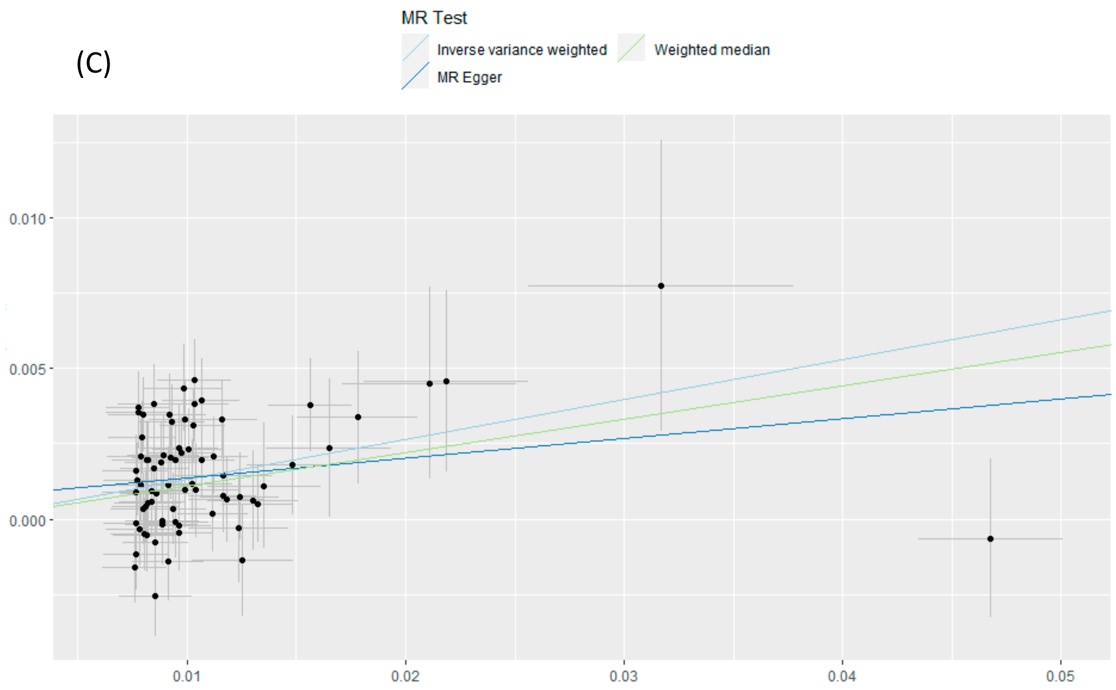
MR Test
Inverse variance weighted
Weighted median
MR Egger
0.010
0.005
0.000
0.01
0.02
0.03
0.04
0.05

(D)

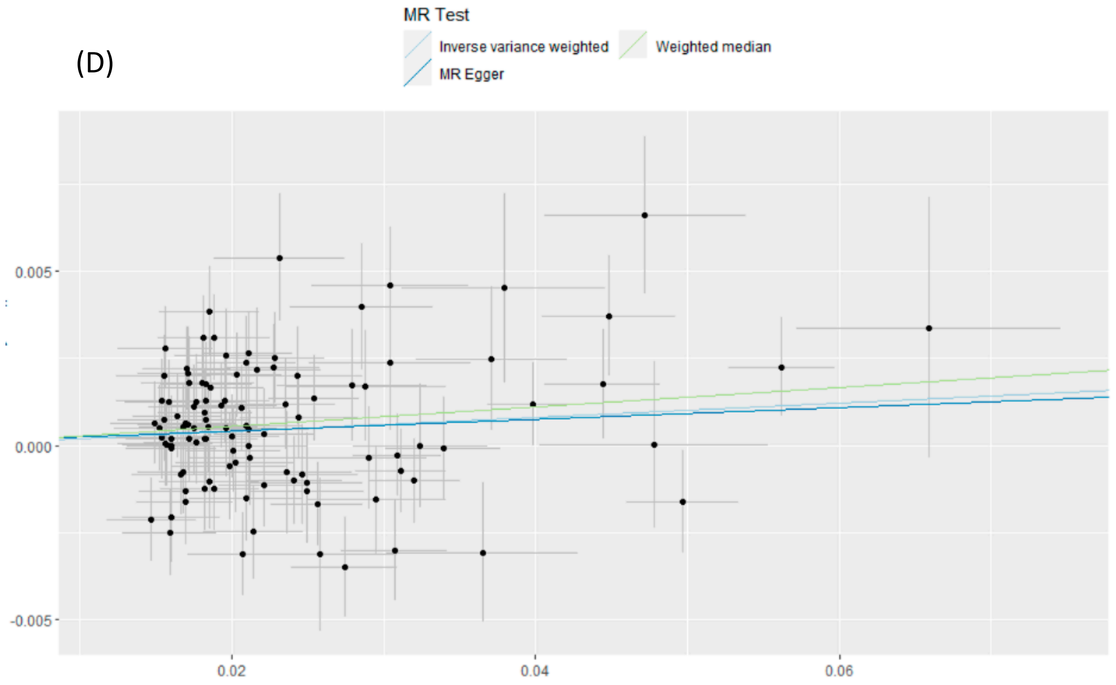
MR Test
Inverse variance weighted
Weighted median
MR Egger
0.005
0.000
-0.005
0.02
0.04
0.06

(E)

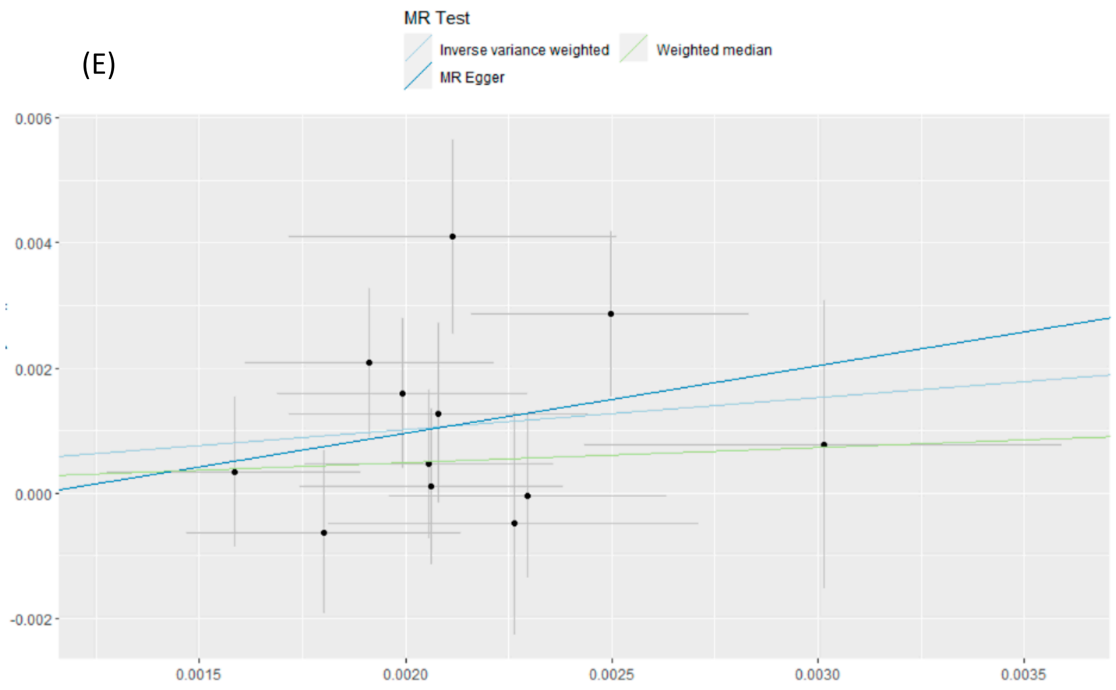
MR Test
Inverse variance weighted
Weighted median
MR Egger
0.006
0.004
0.002
0.000
-0.002
0.0015
0.0020
0.0025
0.0030
0.0035

(F)

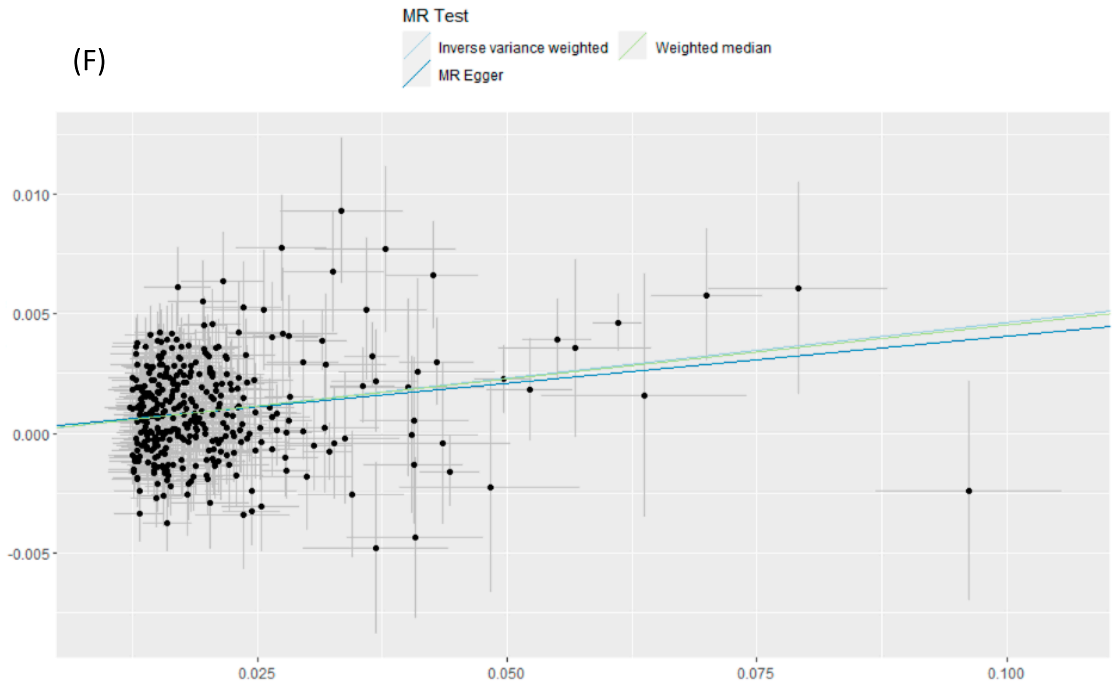
MR Test
Inverse variance weighted
Weighted median
MR Egger
0.010
0.005
0.000
-0.005
0.025
0.050
0.075
0.100

(G)

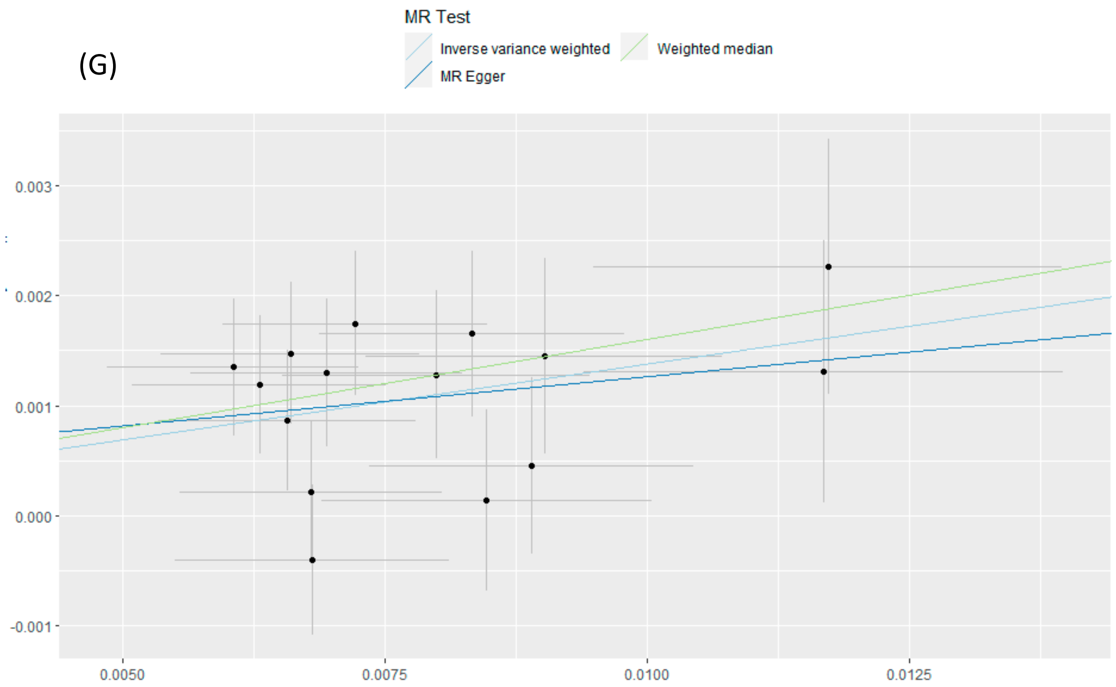
MR Test
Inverse variance weighted
Weighted median
MR Egger
0.003
0.002
0.001
0.000
-0.001
0.0050
0.0075
0.0100
0.0125

(H)

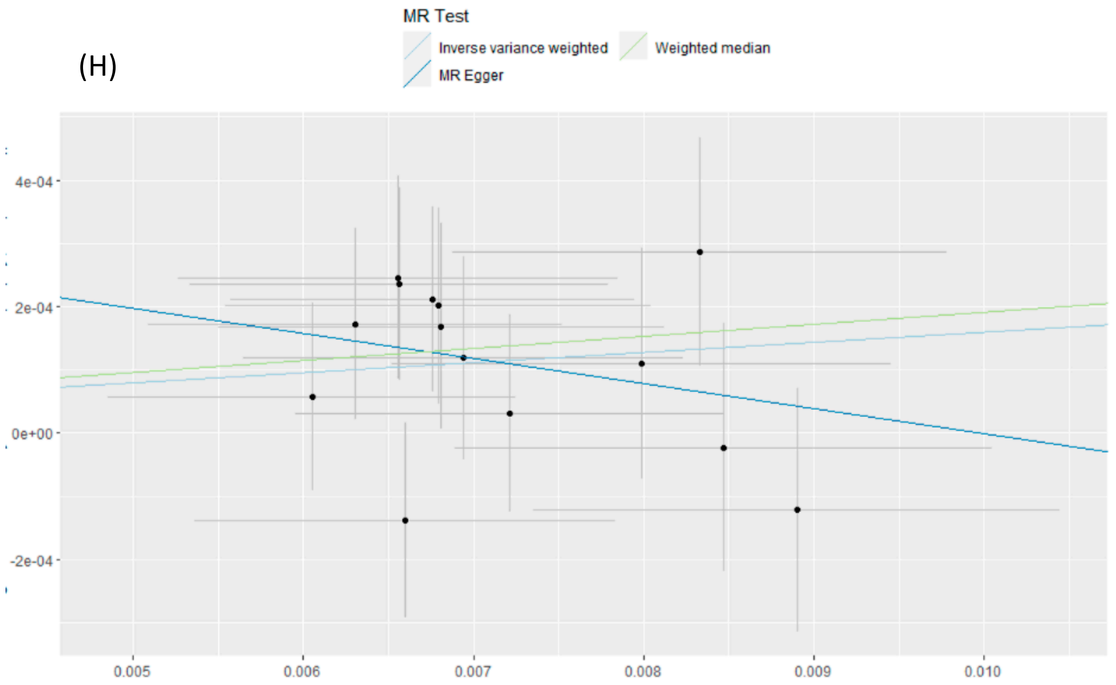
MR Test
Inverse variance weighted
Weighted median
MR Egger
4e-04
2e-04
0e+00
-2e-04
0.005
0.006
0.007
0.008
0.009
0.010

(I)

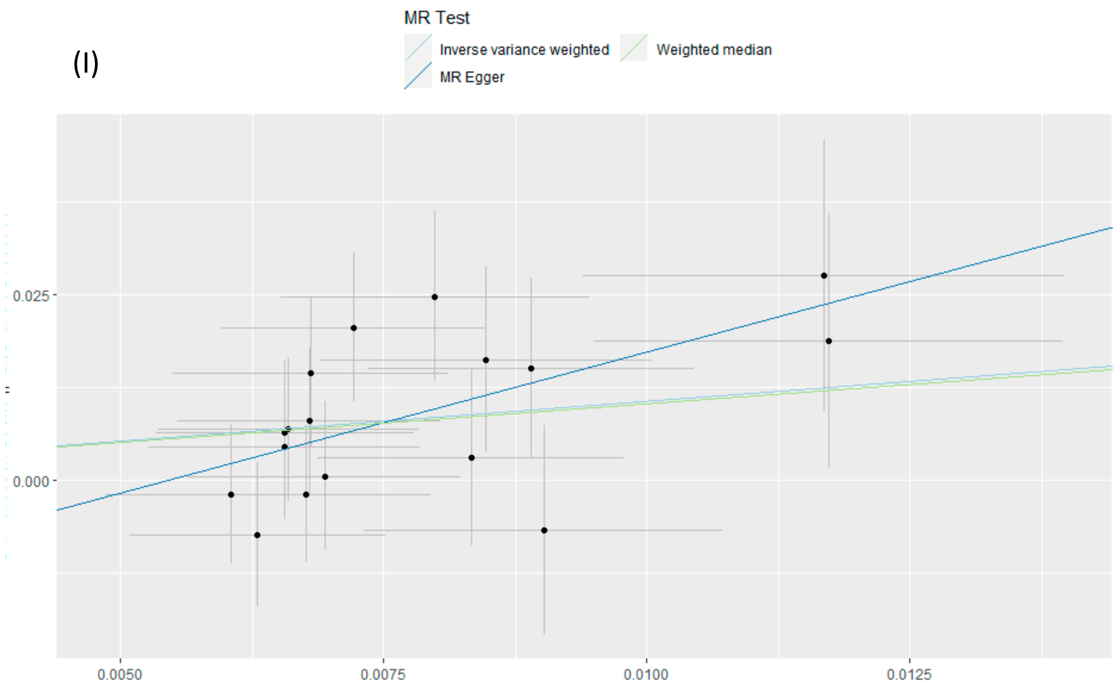

Figure S1 Scatter plots for effect sizes of SNPs for exposures and outcomes.

(A-F) Scatter plots for waist circumference, rheumatoid arthritis, sleeplessness, BMI, osteoporosis, and hip circumference on falls. The x–axis represents the effect size of SNPs on waist circumference, rheumatoid arthritis, sleeplessness, BMI, osteoporosis, and hip circumference; the y-axis represents the effect size of SNPs on falls.

(G-I) Scatter plots for falls on fracture, epilepsy and stroke. The x–axis represents the effect size of SNPs on falls; the y-axis represents the effect size of SNPs on fracture, epilepsy and stroke.

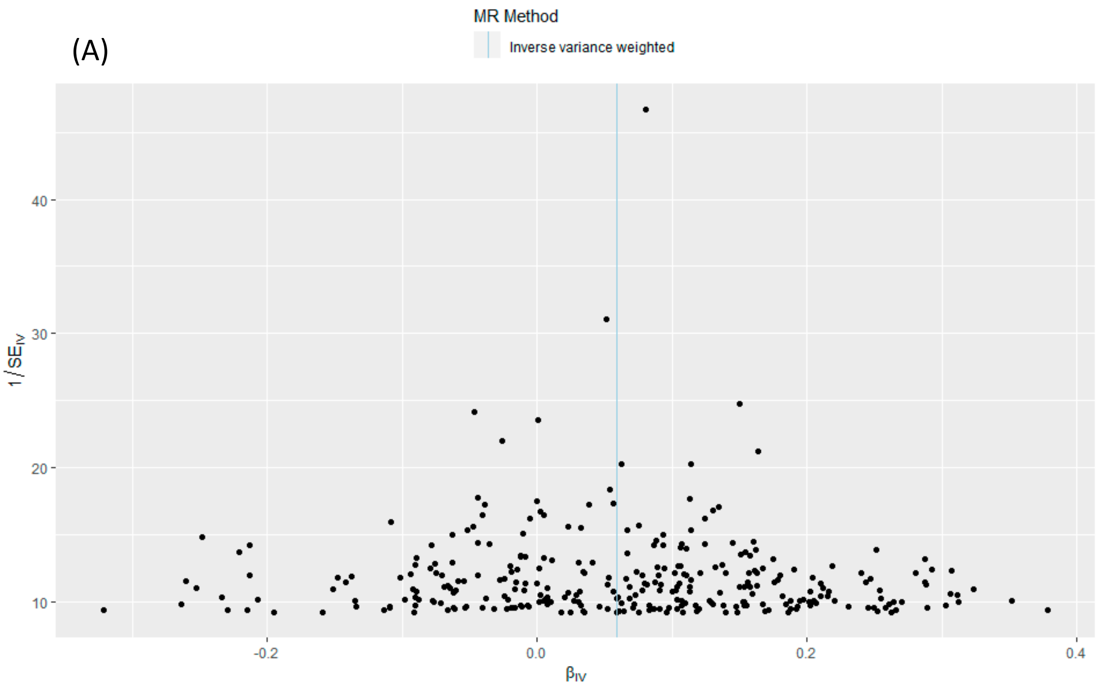

(A)
MR Method
Inverse variance weighted
1/SE$_{IV}$
40
30
20
10
-0.2
0.0
0.2
0.4
β$_{IV}$

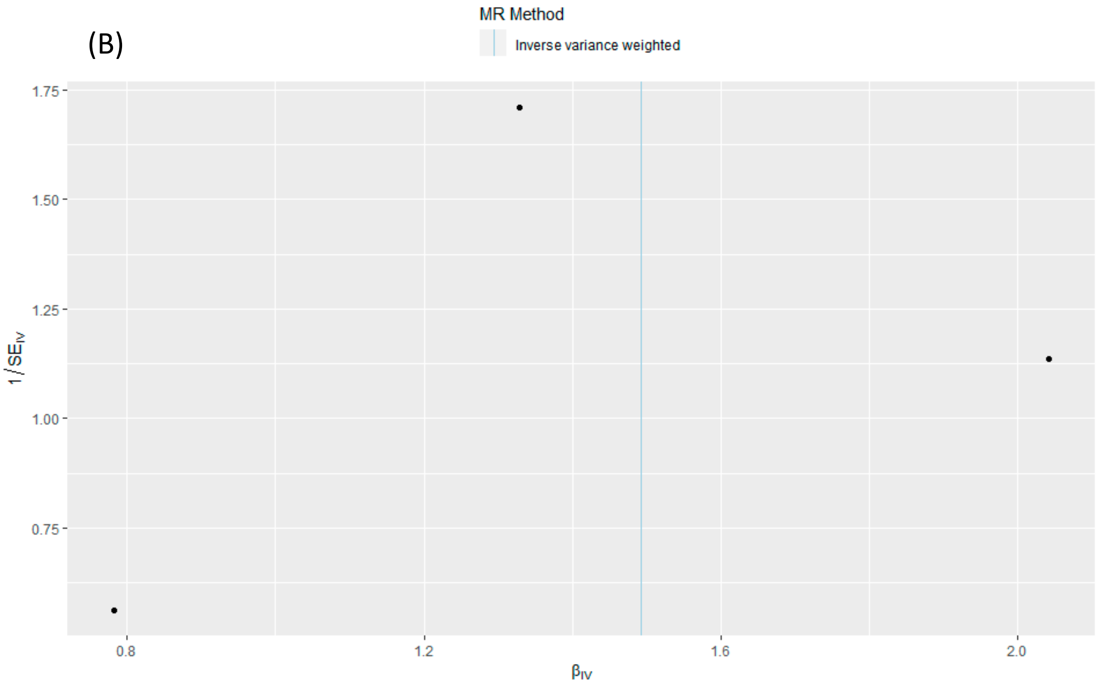

(B)
MR Method
Inverse variance weighted
1/SE$_{IV}$
1.75
1.50
1.25
1.00
0.75
0.8
1.2
1.6
2.0
β$_{IV}$

(C)

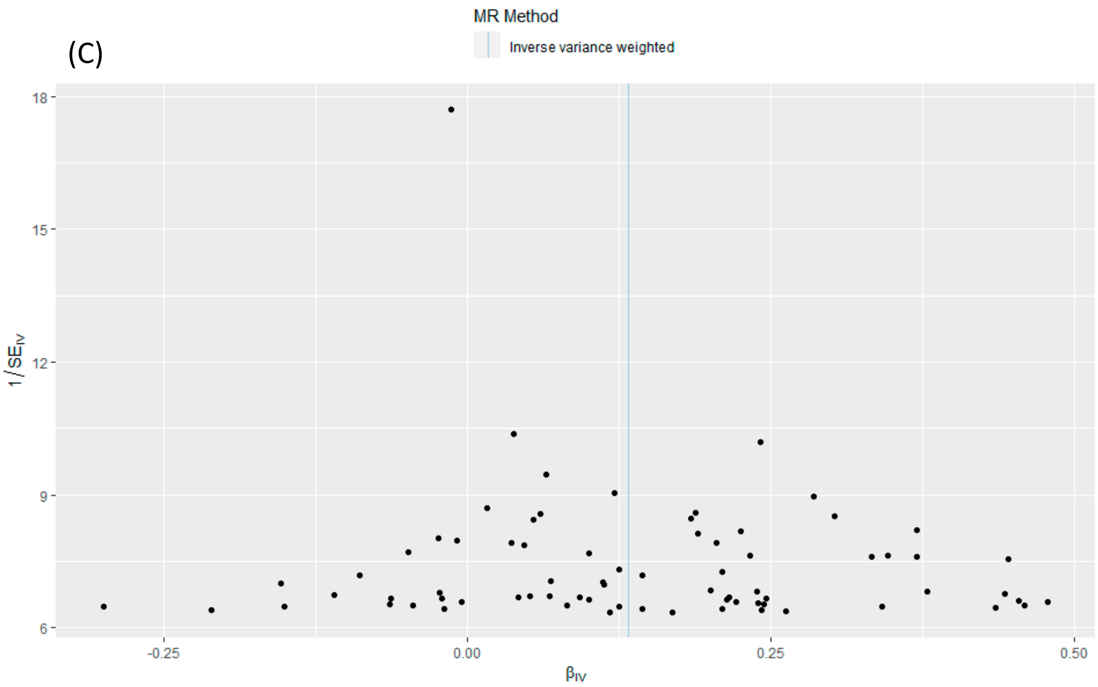
MR Method
Inverse variance weighted
18
15
12
9
6
1/SE$_{IV}$
-0.25
0.00
0.25
0.50
β$_{IV}$

(D)

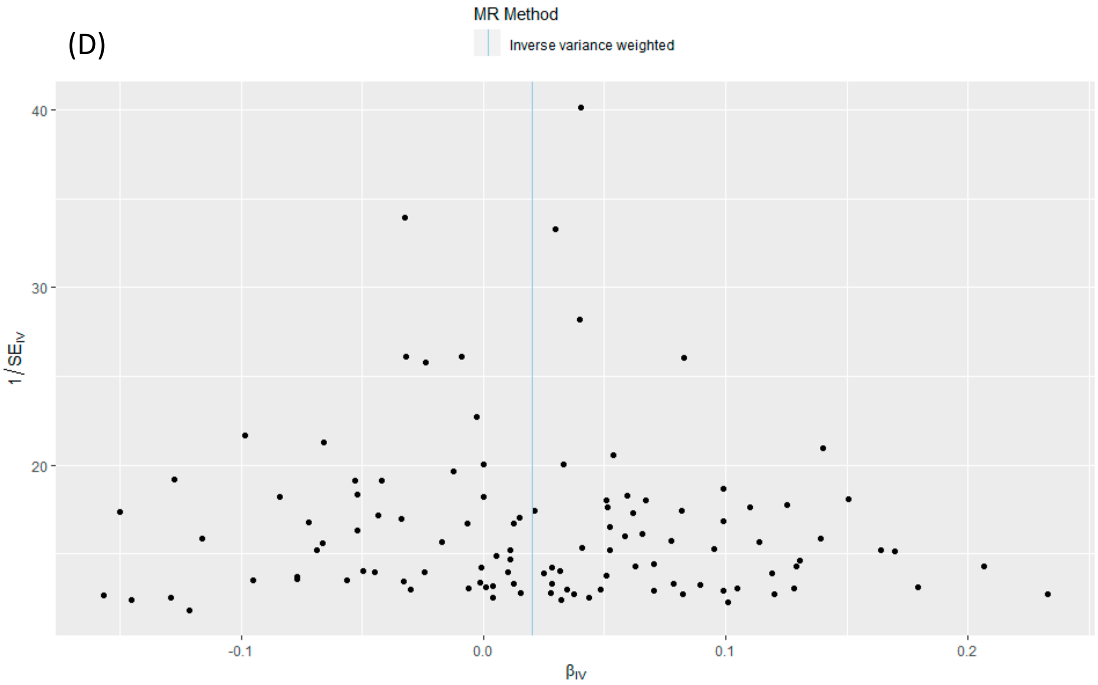
MR Method
Inverse variance weighted
40
30
20
1/SE$_{IV}$
-0.1
0.0
0.1
0.2
β$_{IV}$

(E)

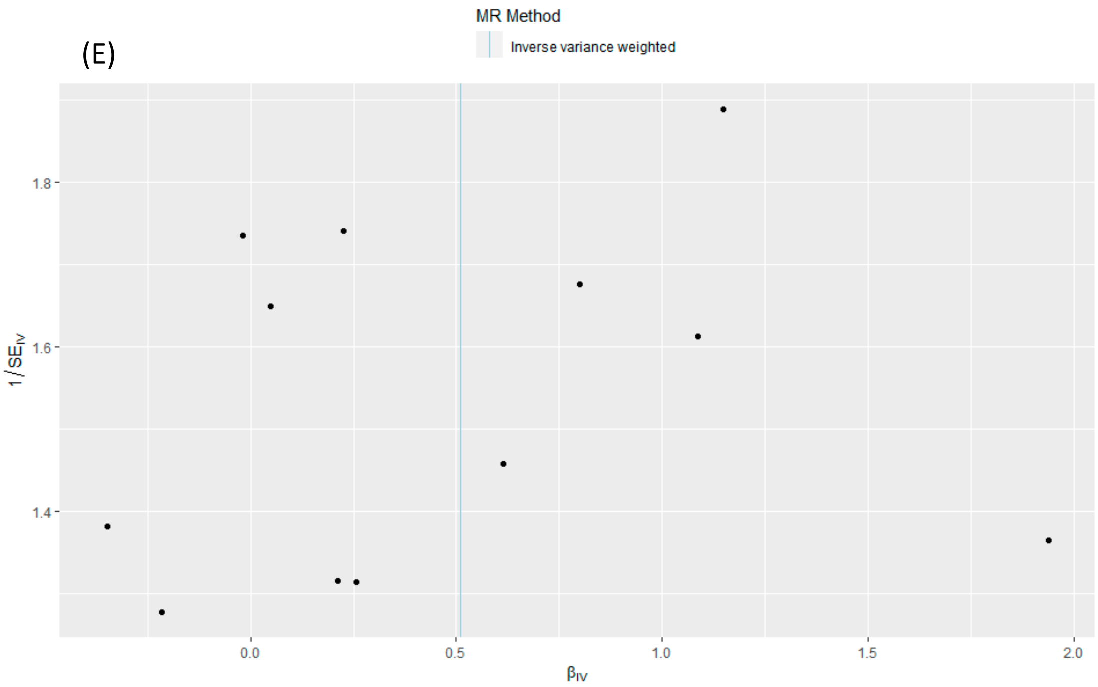
MR Method
Inverse variance weighted
1.8
1.6
1.4
0.0
0.5
1.0
1.5
2.0
β_{IV}
$1/SE_{IV}$

(F)

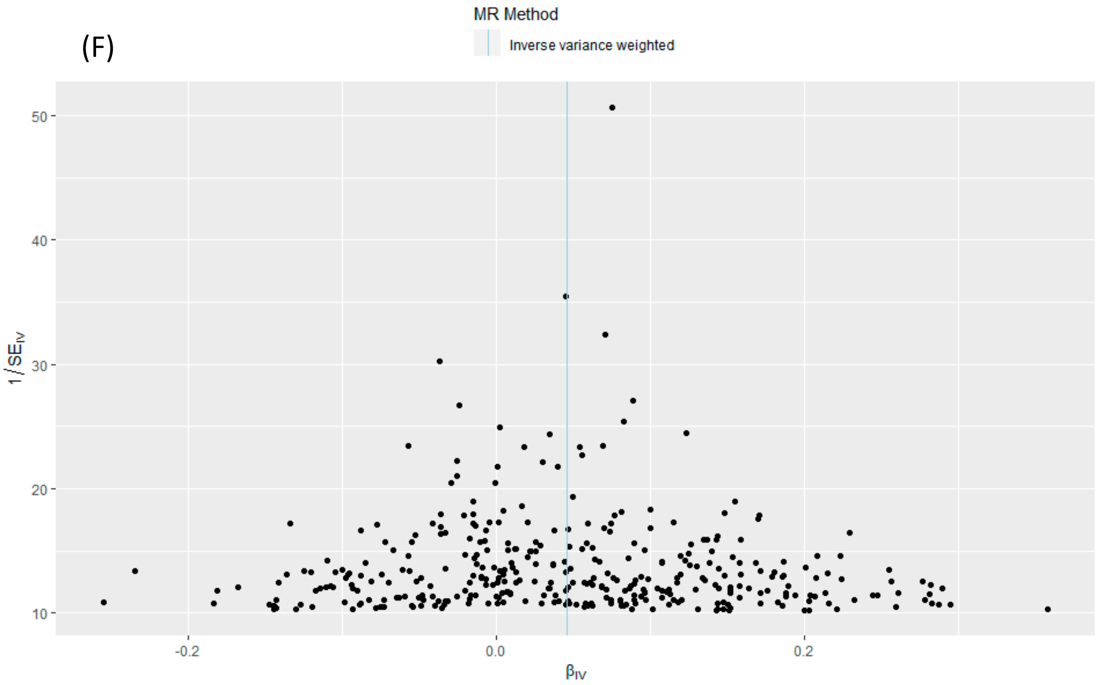
MR Method
Inverse variance weighted
50
40
30
20
10
-0.2
0.0
0.2
β_{IV}
$1/SE_{IV}$

(G)

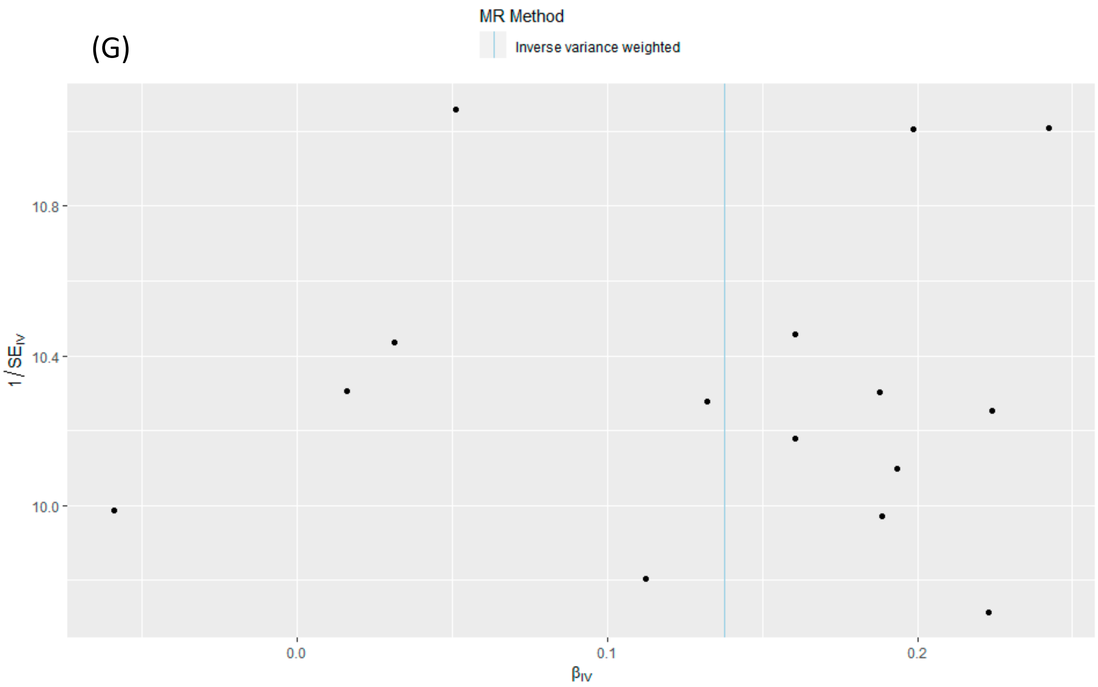

MR Method
Inverse variance weighted
10.8
10.4
10.0
1/SE$_{IV}$
0.0
0.1
0.2
β$_{IV}$

(H)

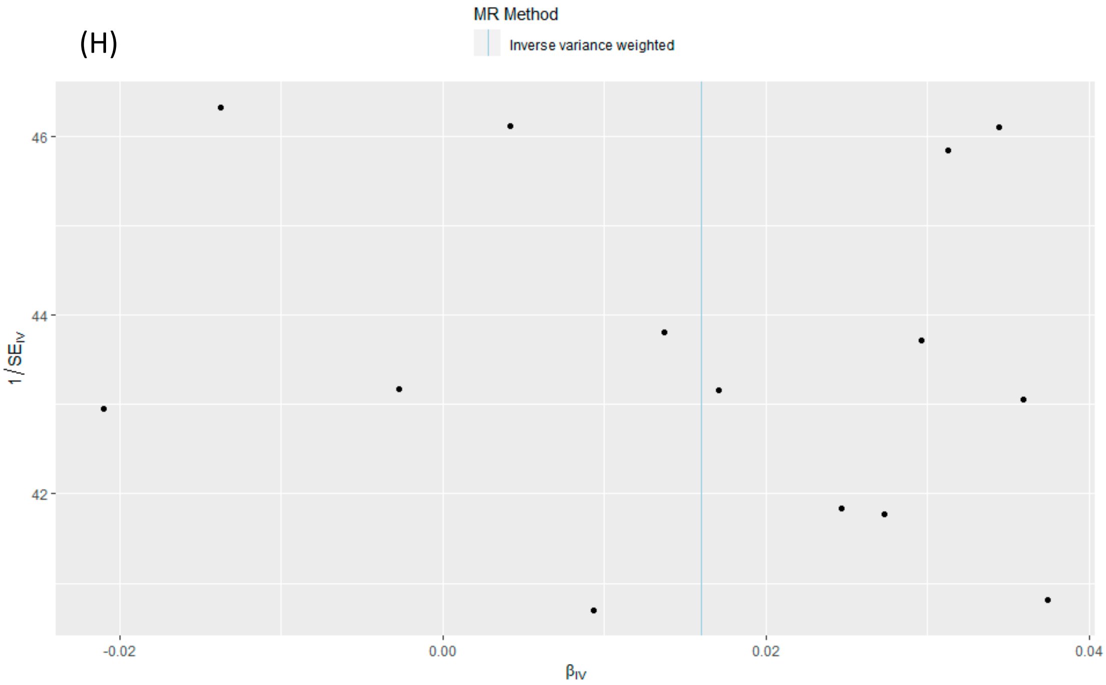

MR Method
Inverse variance weighted
46
44
42
1/SE$_{IV}$
-0.02
0.00
0.02
0.04
β$_{IV}$

(I)

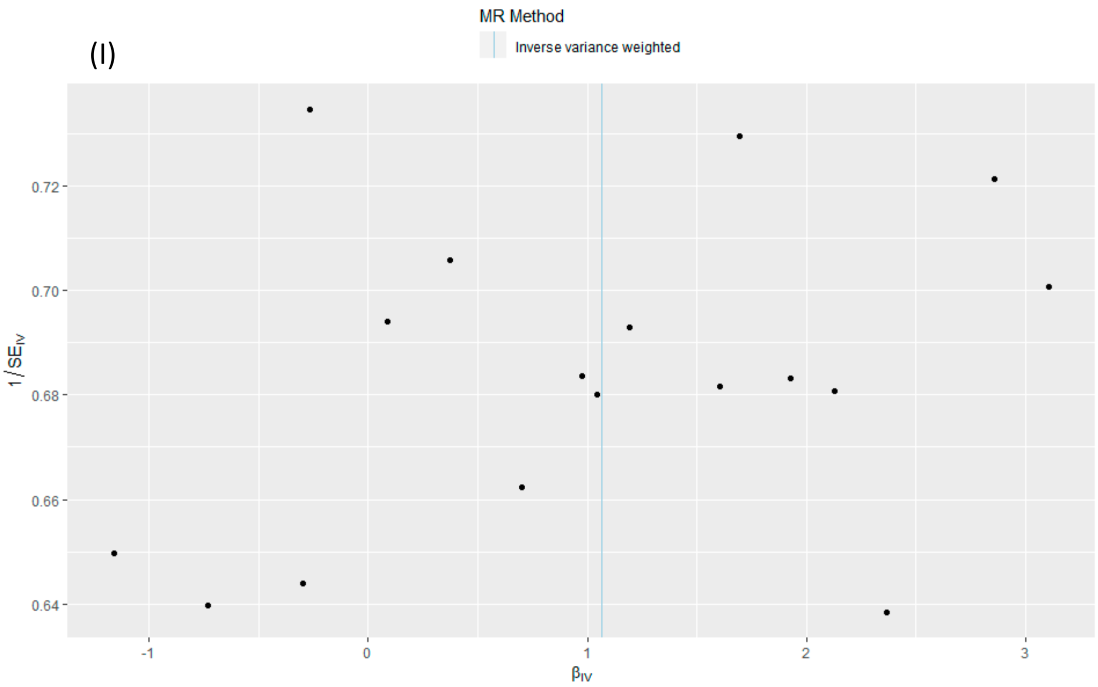

Figure S2 Funnel plots to show symmetrical distribution of individual variant estimates around the point estimate.

(A-F) Funnel plots for waist circumference, rheumatoid arthritis, sleeplessness, BMI, osteoporosis, and hip circumference on falls. The x–axis represents the MR estimate of individual variants; the y–axis represents the inverse of their standard error. Effects were estimated for waist circumference, rheumatoid arthritis, sleeplessness, BMI, osteoporosis, and hip circumference on falls.

(G-I) Funnel plots for falls on fracture, epilepsy and stroke. The x–axis represents the MR estimate of individual variants; the y–axis represents the inverse of their standard error. Effects were estimated for falls on fracture, epilepsy and stroke.

(A)

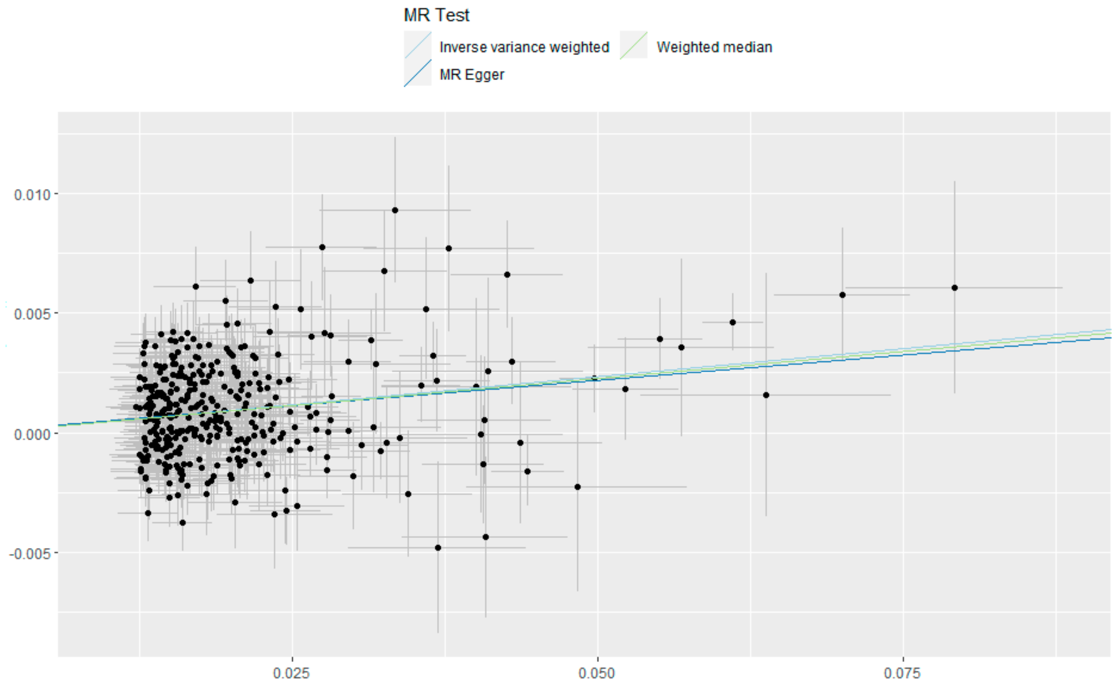

(B)

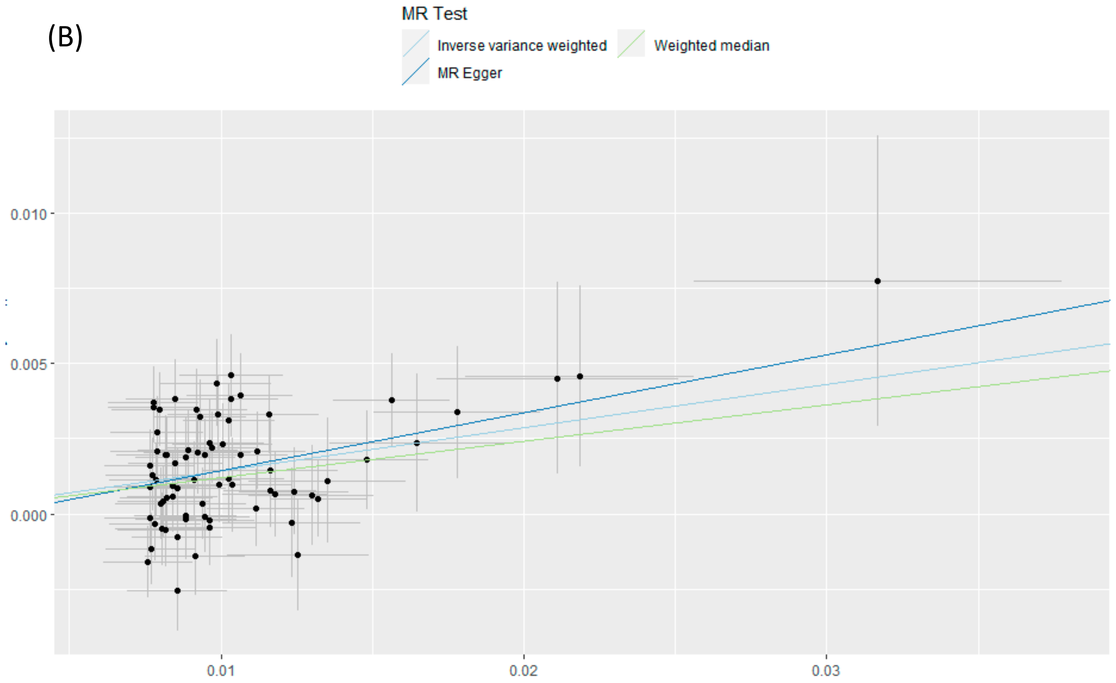

Figure S3. Scatter plots for effect sizes of SNPs for fall without outliers. (A)Scatter plots of hip circumference on fall without outlier rs76895963. (B)Scatter plots of sleeplessness on fall without outlier rs113851554.

(A)

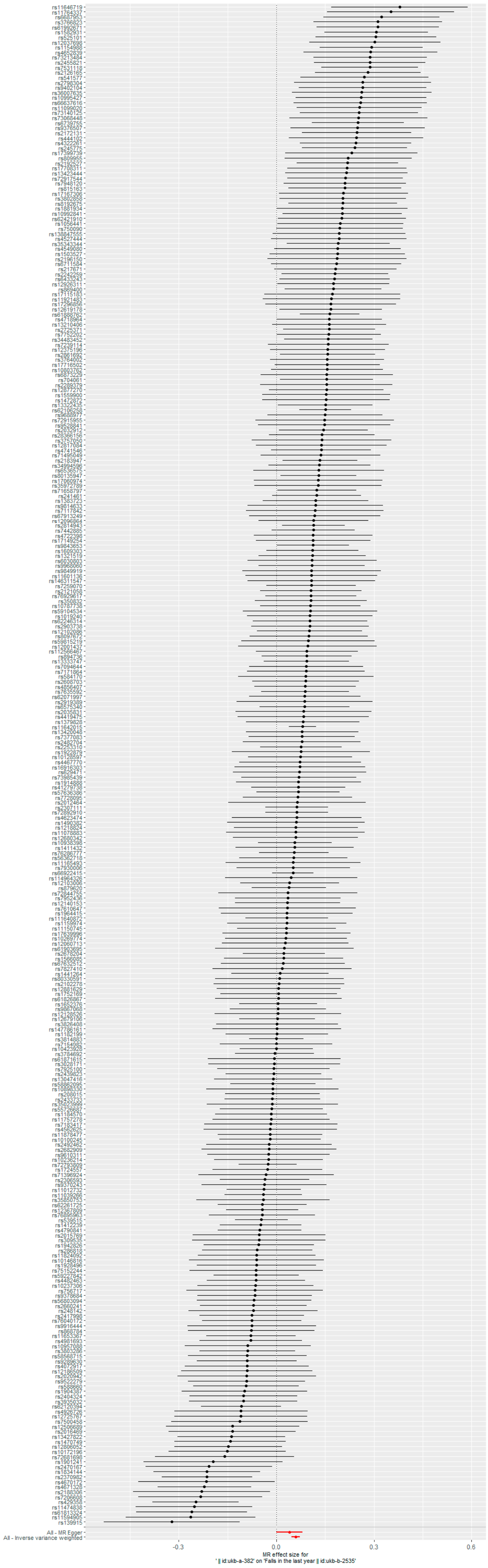
All - MR Egger
All - Inverse variance weighted
-0.3
0.0
0.3
0.6
MR effect size for
' || id:ukb-a-382' on 'Falls in the last year || id:ukb-b-2535'

(B)

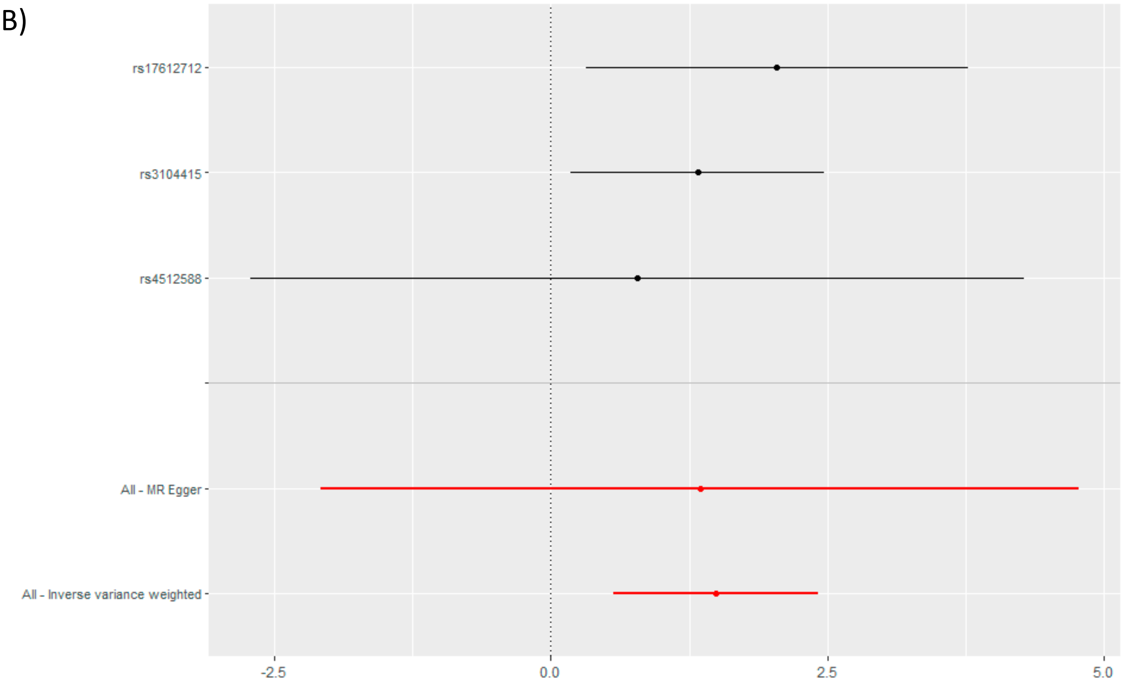
rs17612712
rs3104415
rs4512588
All - MR Egger
All - Inverse variance weighted
-2.5
0.0
2.5
5.0

(C)

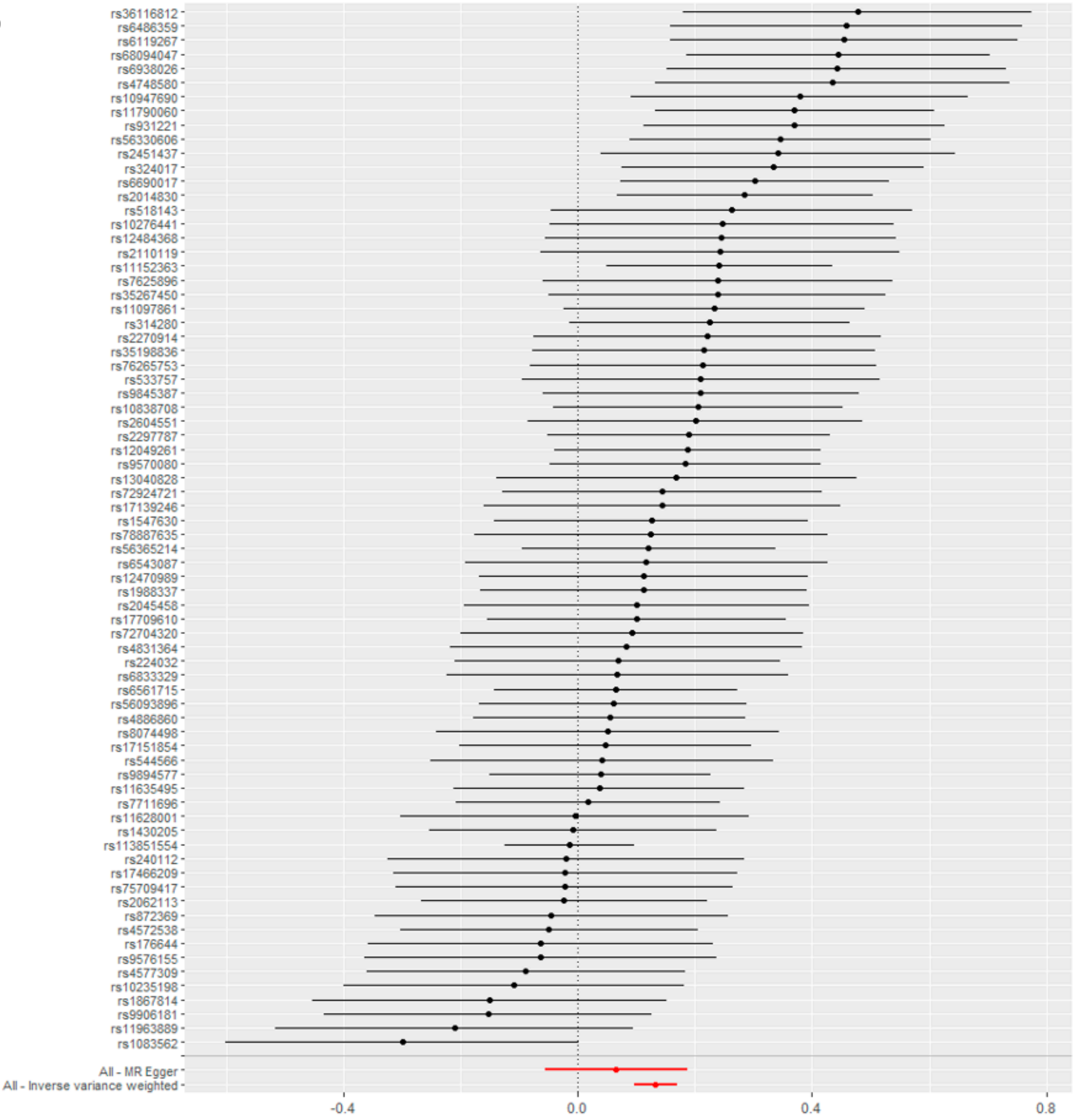
rs36116812
rs6486359
rs6119267
rs68094047
rs6938026
rs4748580
rs10947690
rs11790060
rs931221
rs56330606
rs2451437
rs324017
rs6690017
rs2014830
rs518143
rs10276441
rs12484368
rs2110119
rs11152363
rs7625896
rs35267450
rs11097861
rs314280
rs2270914
rs35198836
rs76265753
rs533757
rs9845387
rs10838708
rs2604551
rs2297787
rs12049261
rs9570080
rs13040828
rs72924721
rs17139246
rs1547630
rs78887635
rs56365214
rs6543087
rs12470989
rs1988337
rs2045458
rs17709610
rs72704320
rs4831364
rs224032
rs6833329
rs6561715
rs56093896
rs4886860
rs8074498
rs17151854
rs544566
rs9894577
rs11635495
rs7711696
rs11628001
rs1430205
rs113851554
rs240112
rs17466209
rs75709417
rs2062113
rs872369
rs4572538
rs176644
rs9576155
rs4577309
rs10235198
rs1867814
rs9906181
rs11963889
rs1083562
All - MR Egger
All - Inverse variance weighted
-0.4
0.0
0.4
0.8

(D)

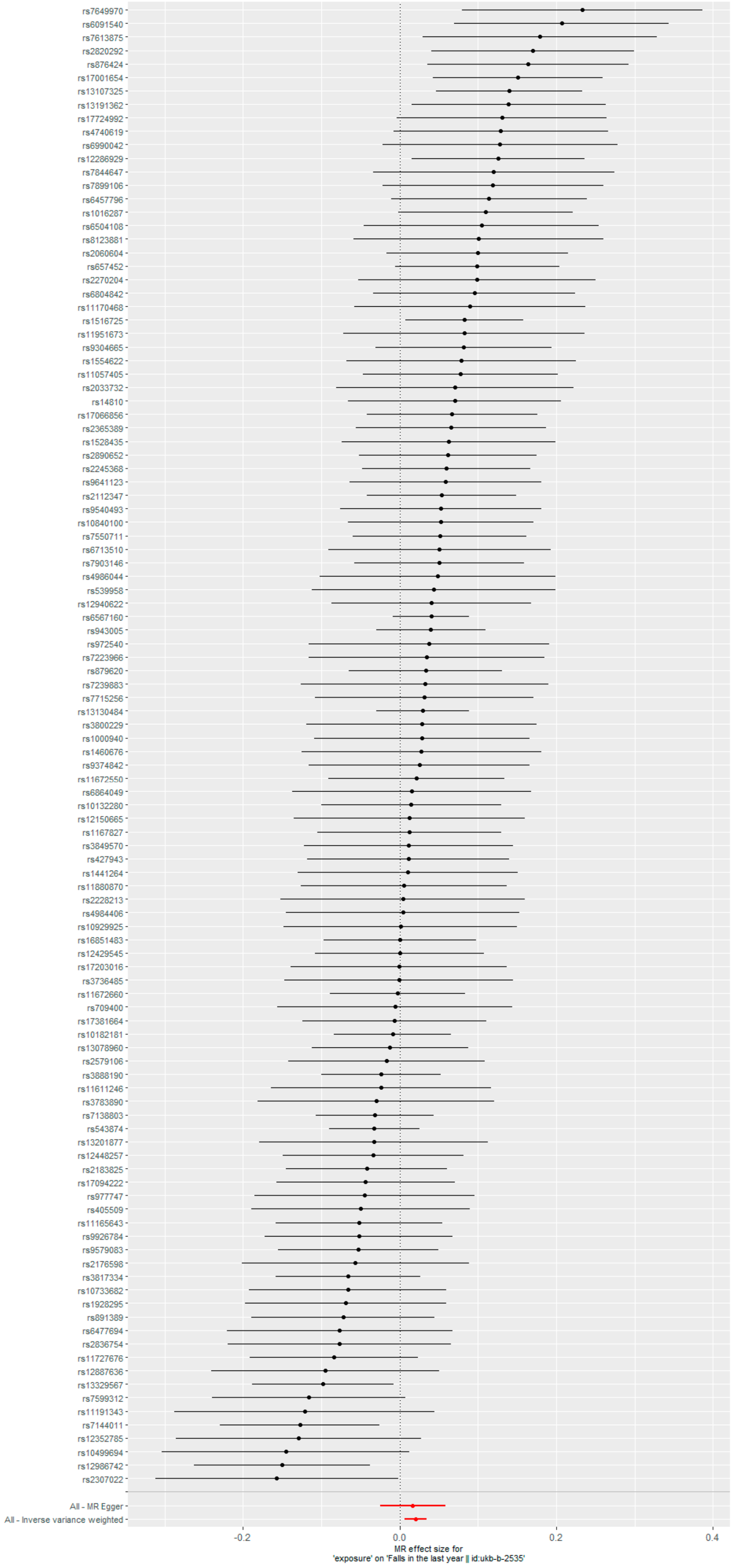
rs7649970
rs6091540
rs7613875
rs2820292
rs876424
rs17001654
rs13107325
rs13191362
rs17724992
rs4740619
rs6990042
rs12286929
rs7844647
rs7899106
rs6457796
rs1016287
rs6504108
rs8123881
rs2060604
rs657452
rs2270204
rs6804842
rs11170468
rs1516725
rs11951673
rs9304665
rs1554622
rs11057405
rs2033732
rs14810
rs17066856
rs2365389
rs1528435
rs2890652
rs2245368
rs9641123
rs2112347
rs9540493
rs10840100
rs7550711
rs6713510
rs7903146
rs4986044
rs539958
rs12940622
rs6567160
rs943005
rs972540
rs7223966
rs879620
rs7239883
rs7715256
rs13130484
rs3800229
rs1000940
rs1460676
rs9374842
rs11672550
rs6864049
rs10132280
rs12150665
rs1167827
rs3849570
rs427943
rs1441264
rs11880870
rs2228213
rs4984406
rs10929925
rs16851483
rs12429545
rs17203016
rs3736485
rs11672660
rs709400
rs17381664
rs10182181
rs13078960
rs2579106
rs3888190
rs11611246
rs3783890
rs7138803
rs543874
rs13201877
rs12448257
rs2183825
rs17094222
rs977747
rs405509
rs11165643
rs9926784
rs9579083
rs2176598
rs3817334
rs10733682
rs1928295
rs891389
rs6477694
rs2836754
rs11727676
rs12887636
rs13329567
rs7599312
rs11191343
rs7144011
rs12352785
rs10499694
rs12986742
rs2307022
All - MR Egger
All - Inverse variance weighted
-0.2
0.0
0.2
0.4
MR effect size for
'exposure' on 'Falls in the last year || id:ukb-b-2535'

(E)

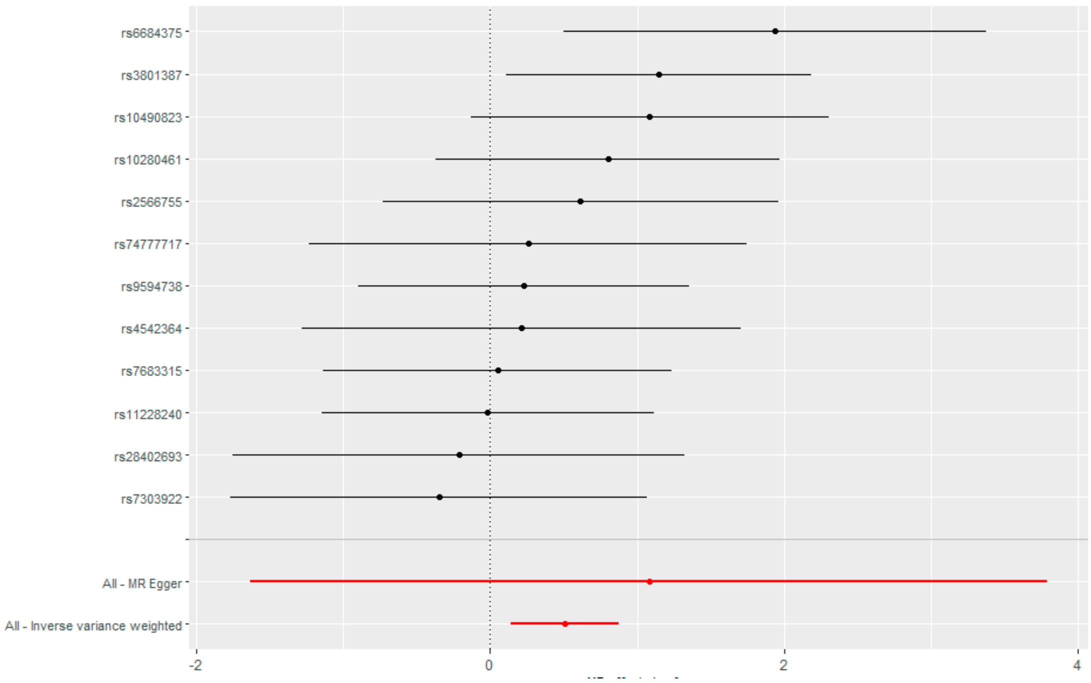
rs6684375
rs3801387
rs10490823
rs10280461
rs2566755
rs74777717
rs9594738
rs4542364
rs7683315
rs11228240
rs28402693
rs7303922
All - MR Egger
All - Inverse variance weighted
-2
0
2
4

(F)

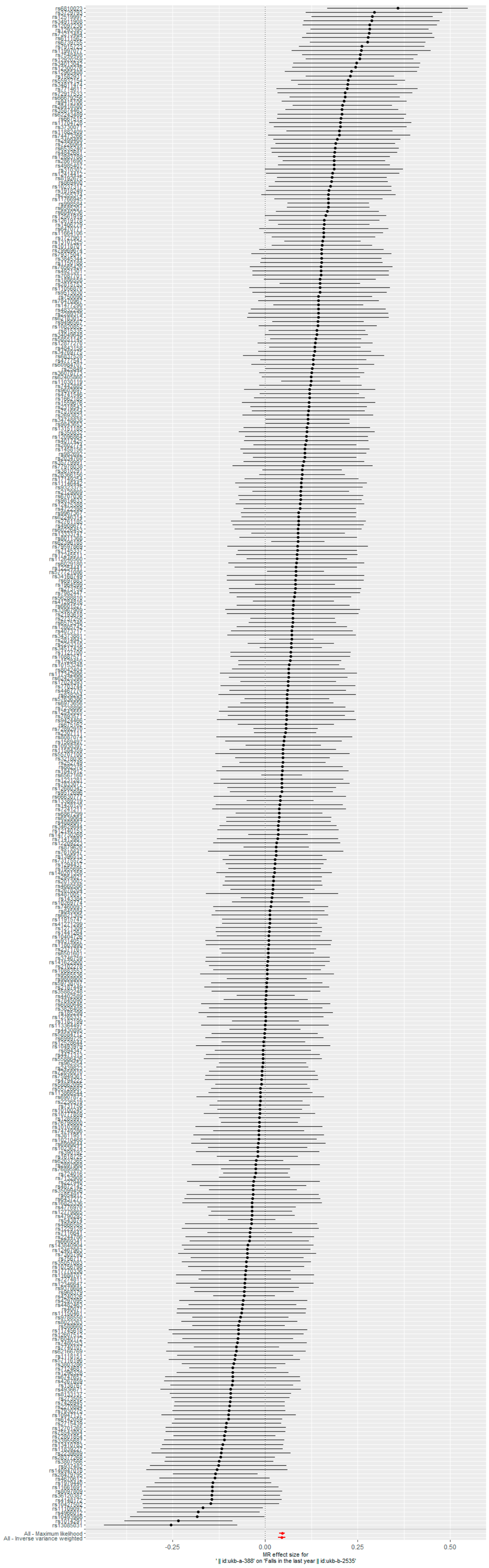
All - Maximum likelihood
All - Inverse variance weighted
-0.25
0.00
0.25
0.50
MR effect size for
' || id:ukb-a-388' on 'Falls in the last year || id:ukb-b-2535'

(G)

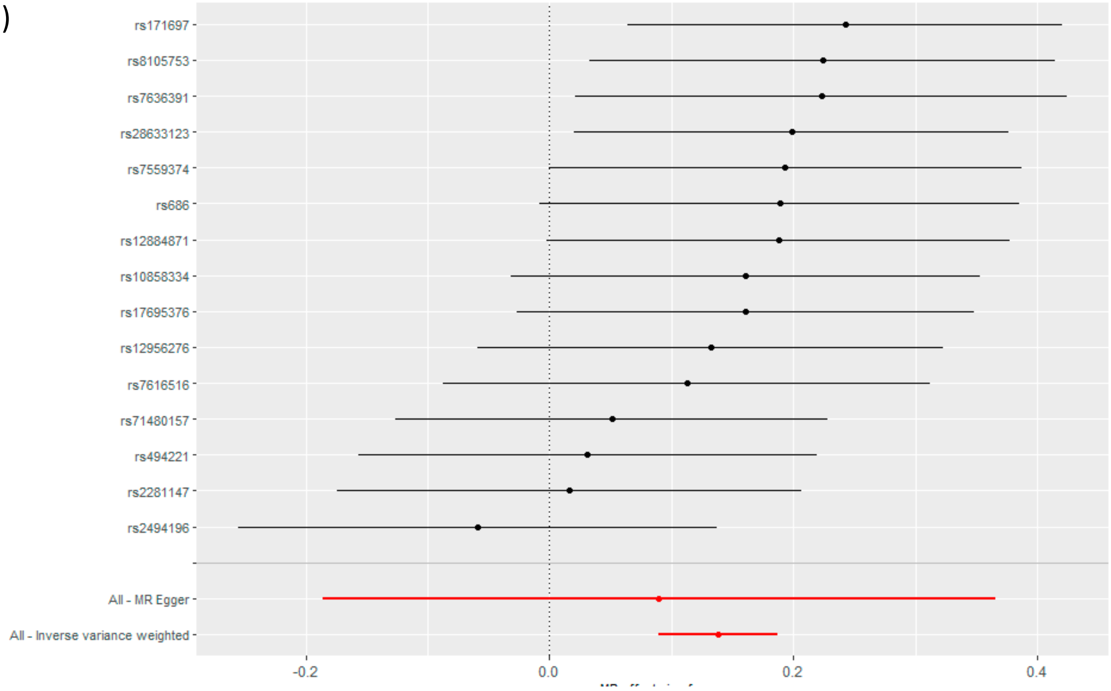
rs171697
rs8105753
rs7636391
rs28633123
rs7559374
rs686
rs12884871
rs10858334
rs17695376
rs12956276
rs7616516
rs71480157
rs494221
rs2281147
rs2494196
All - MR Egger
All - Inverse variance weighted
-0.2
0.0
0.2
0.4

(H)

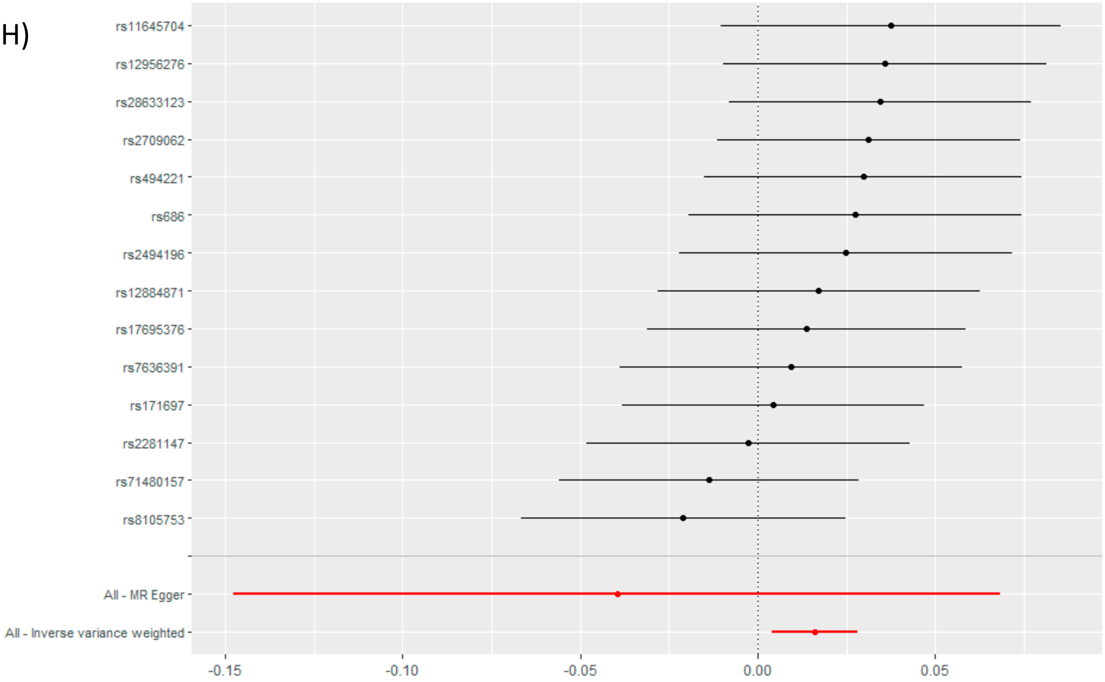
rs11645704
rs12956276
rs28633123
rs2709062
rs494221
rs686
rs2494196
rs12884871
rs17695376
rs7636391
rs171697
rs2281147
rs71480157
rs8105753
All - MR Egger
All - Inverse variance weighted
-0.15
-0.10
-0.05
0.00
0.05

(I)

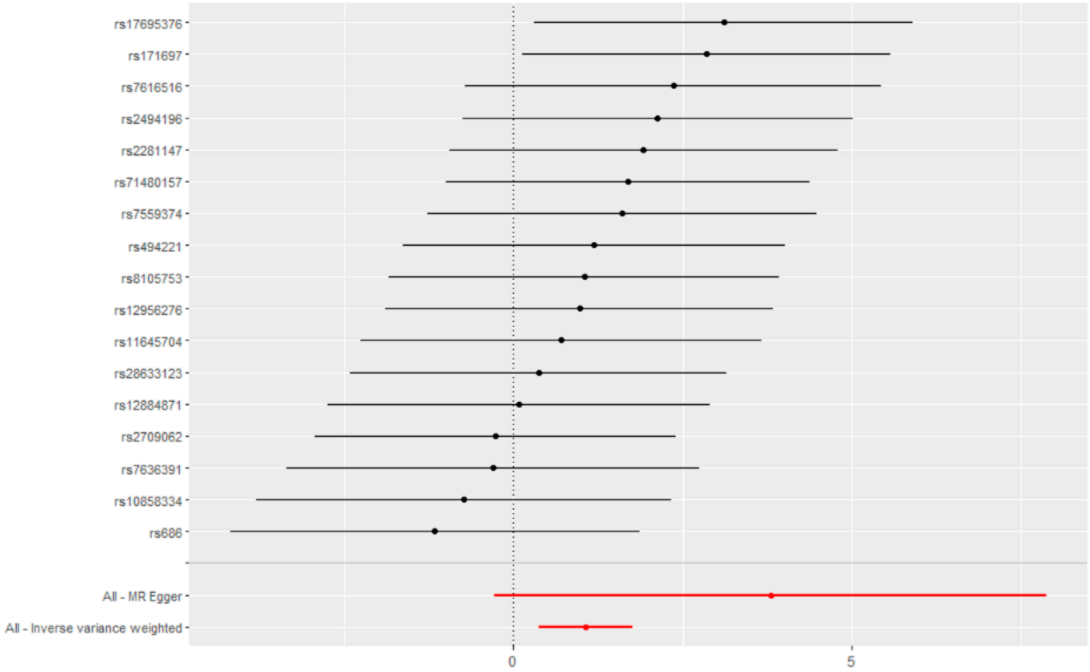

Figure S4 Forest plots between exposures and outcomes.

(A-F) Forest plots presenting the relationships between waist circumference, rheumatoid arthritis, sleeplessness, BMI, osteoporosis, hip circumference, and falls. (G-I) Forest plots presenting the relationships between falls and fracture, epilepsy, and stroke.

(A)

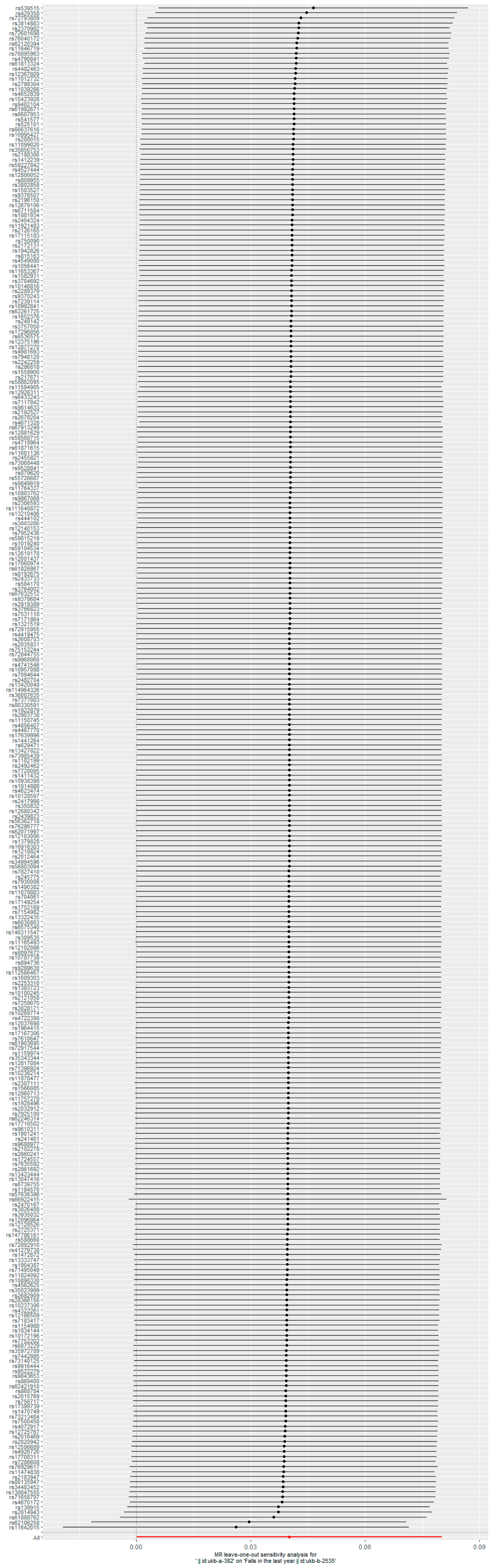
All
0.00
0.03
0.06
0.09
MR leave-one-out sensitivity analysis for
' || id:ukb-a-382' on 'Falls in the last year || id:ukb-b-2535'

(B)

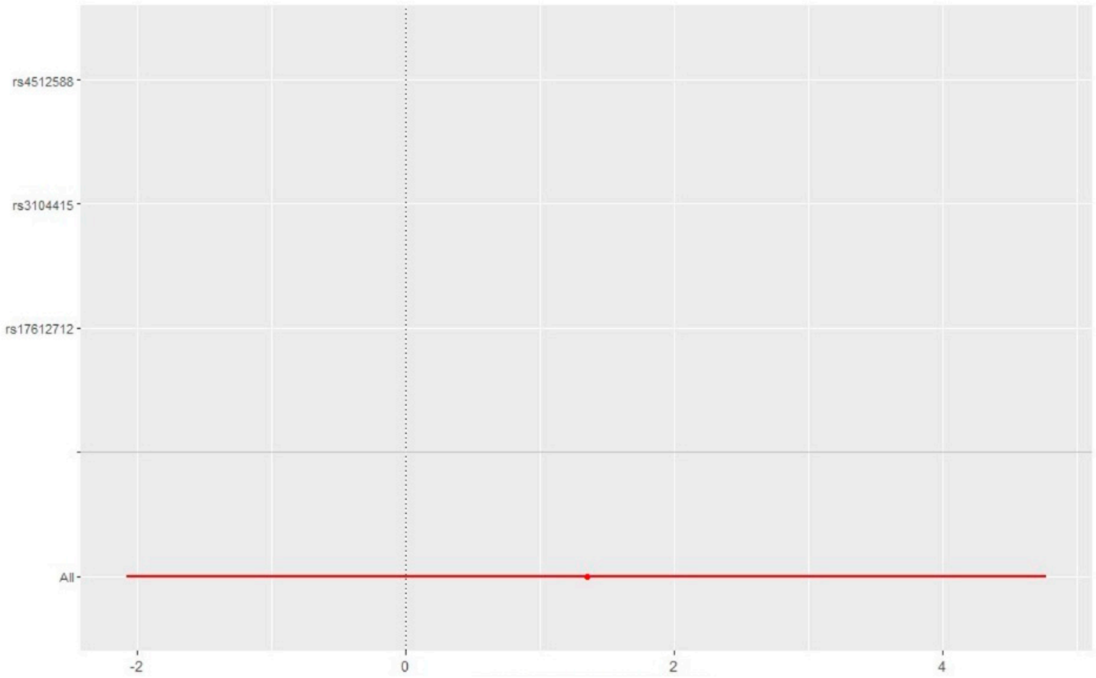

(C)

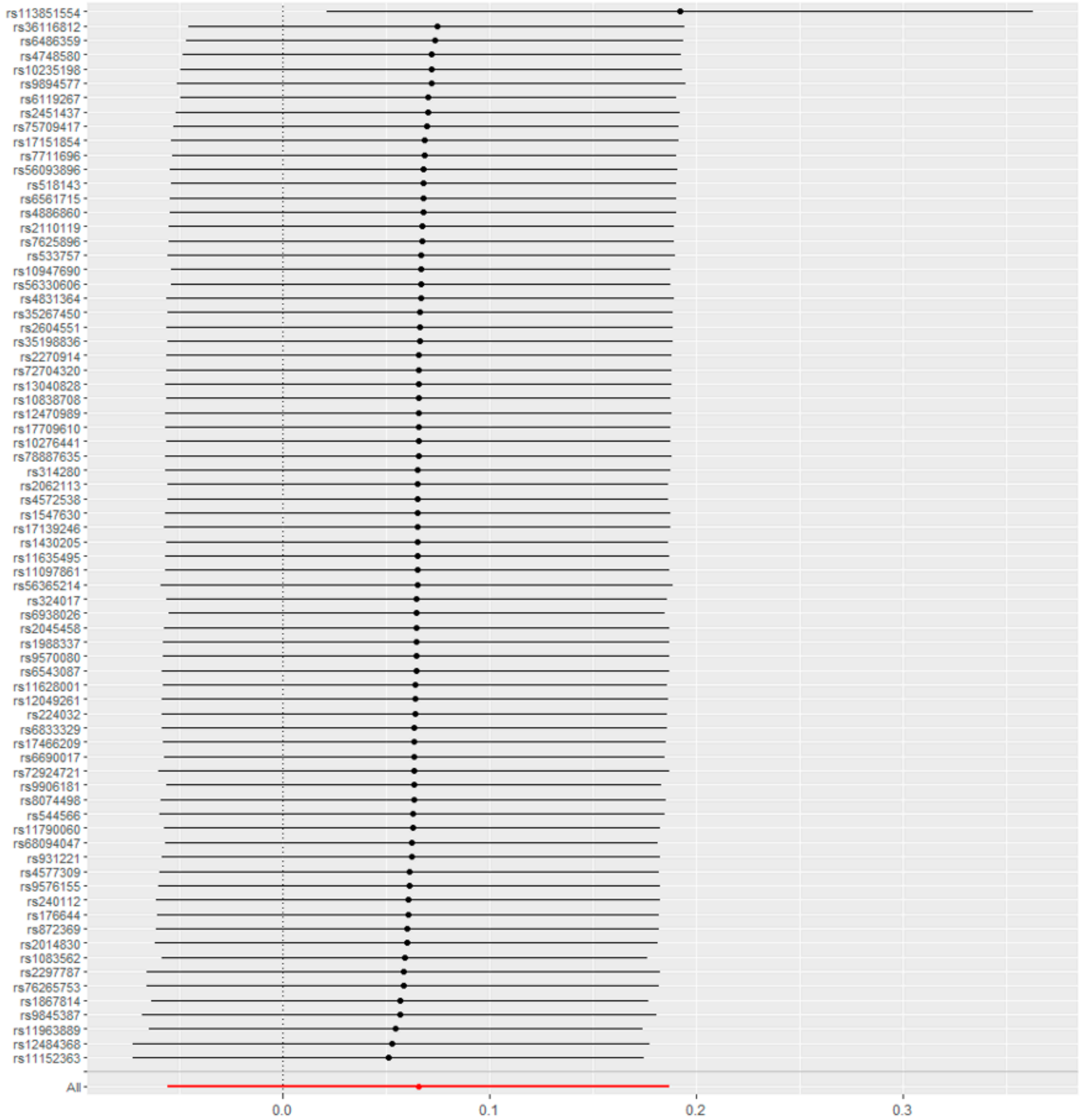
rs113851554
rs36116812
rs6486359
rs4748580
rs10235198
rs9894577
rs6119267
rs2451437
rs75709417
rs17151854
rs7711696
rs56093896
rs518143
rs6561715
rs4886860
rs2110119
rs7625896
rs533757
rs10947690
rs56330606
rs4831364
rs35267450
rs2604551
rs35198836
rs2270914
rs72704320
rs13040828
rs10838708
rs12470989
rs17709610
rs10276441
rs78887635
rs314280
rs2062113
rs4572538
rs1547630
rs17139246
rs1430205
rs11635495
rs11097861
rs56365214
rs324017
rs6938026
rs2045458
rs1988337
rs9570080
rs6543087
rs11628001
rs12049261
rs224032
rs6833329
rs17466209
rs6690017
rs72924721
rs9906181
rs8074498
rs544566
rs11790060
rs68094047
rs931221
rs4577309
rs9576155
rs240112
rs176644
rs872369
rs2014830
rs1083562
rs2297787
rs76265753
rs1867814
rs9845387
rs11963889
rs12484368
rs11152363
All
0.0
0.1
0.2
0.3

(D)

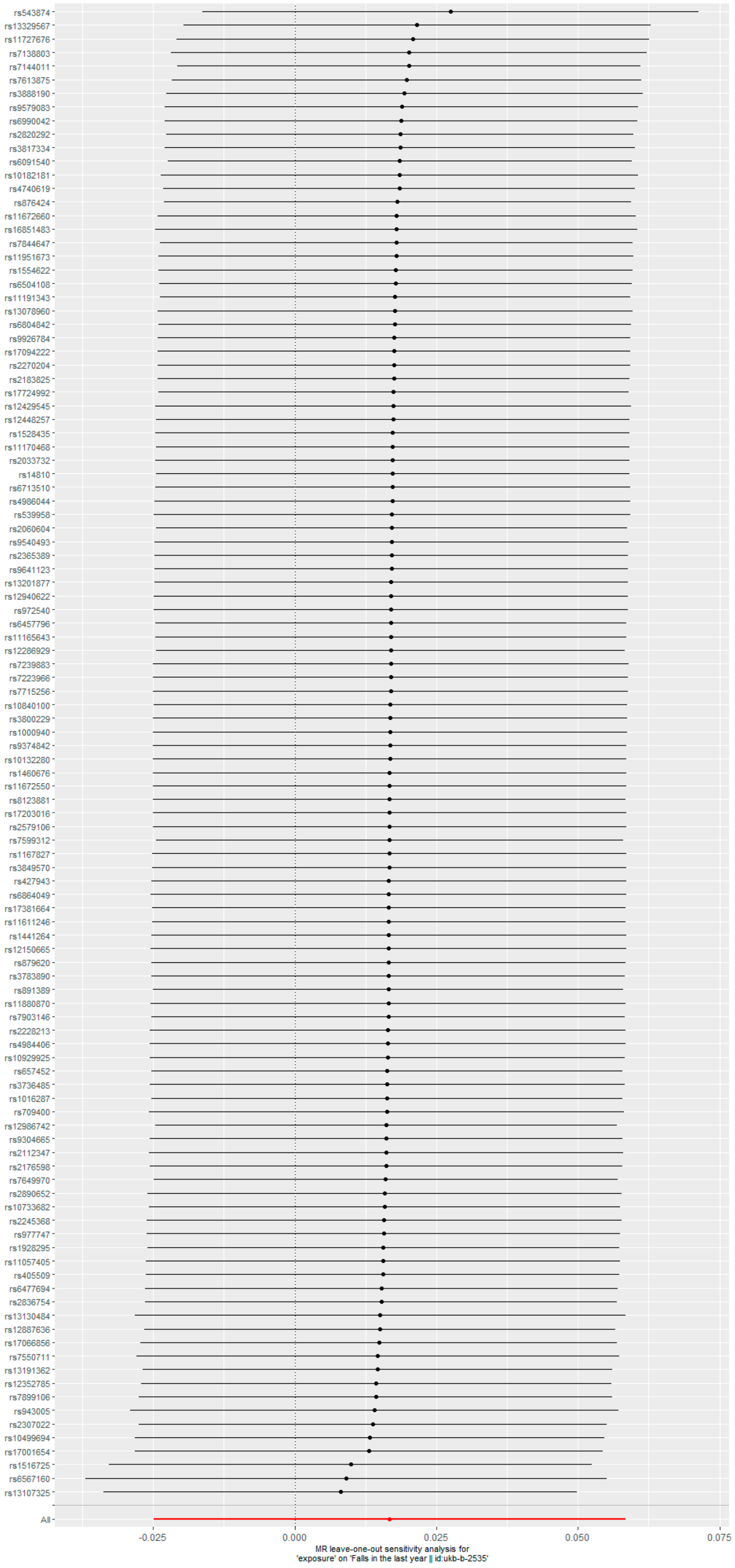
rs543874
rs13329567
rs11727676
rs7138803
rs7144011
rs7613875
rs3888190
rs9579083
rs6990042
rs2820292
rs3817334
rs6091540
rs10182181
rs4740619
rs876424
rs11672660
rs16851483
rs7844647
rs11951673
rs1554622
rs6504108
rs11191343
rs13078960
rs6804842
rs9926784
rs17094222
rs2270204
rs2183825
rs17724992
rs12429545
rs12448257
rs1528435
rs11170468
rs2033732
rs14810
rs6713510
rs4986044
rs539958
rs2060604
rs9540493
rs2365389
rs9641123
rs13201877
rs12940622
rs972540
rs6457796
rs11165643
rs12286929
rs7239883
rs7223966
rs7715256
rs10840100
rs3800229
rs1000940
rs9374842
rs10132280
rs1460676
rs11672550
rs8123881
rs17203016
rs2579106
rs7599312
rs1167827
rs3849570
rs427943
rs6864049
rs17381664
rs11611246
rs1441264
rs12150665
rs879620
rs3783890
rs891389
rs11880870
rs7903146
rs2228213
rs4984406
rs10929925
rs657452
rs3736485
rs1016287
rs709400
rs12986742
rs9304665
rs2112347
rs2176598
rs7649970
rs2890652
rs10733682
rs2245368
rs977747
rs1928295
rs11057405
rs405509
rs6477694
rs2836754
rs13130484
rs12887636
rs17066856
rs7550711
rs13191362
rs12352785
rs7899106
rs943005
rs2307022
rs10499694
rs17001654
rs1516725
rs6567160
rs13107325
All
-0.025
0.000
0.025
0.050
0.075
MR leave-one-out sensitivity analysis for
'exposure' on 'Falls in the last year || id:ukb-b-2535'

(E)

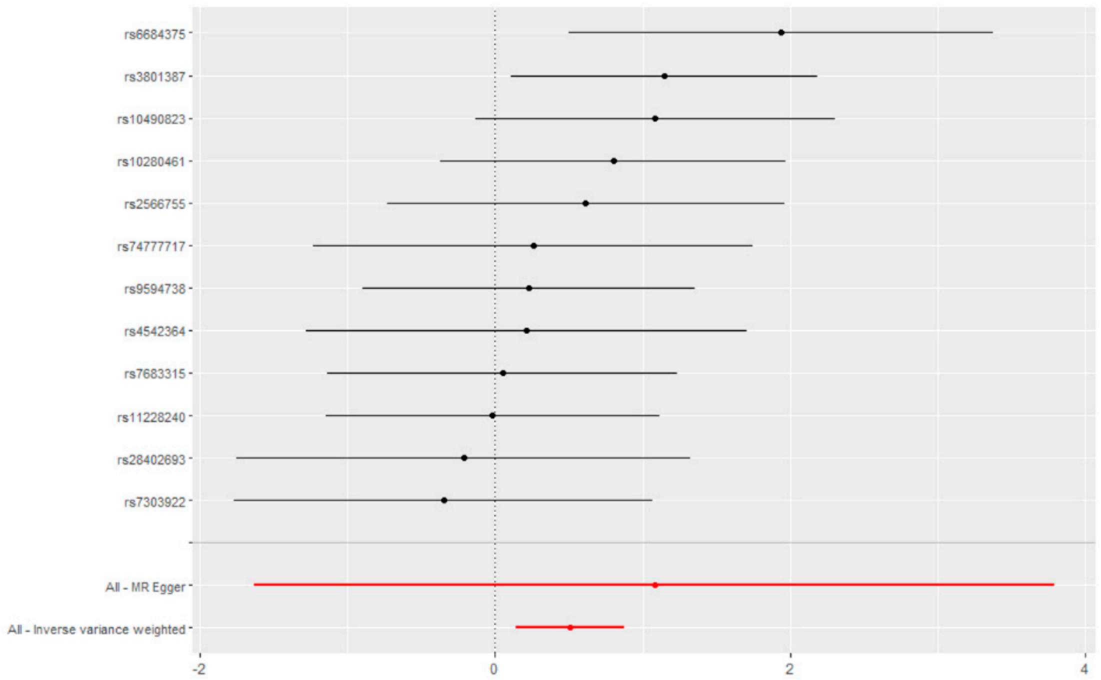
rs6684375
rs3801387
rs10490823
rs10280461
rs2566755
rs74777717
rs9594738
rs4542364
rs7683315
rs11228240
rs28402693
rs7303922
All - MR Egger
All - Inverse variance weighted
-2
0
2
4

(F)

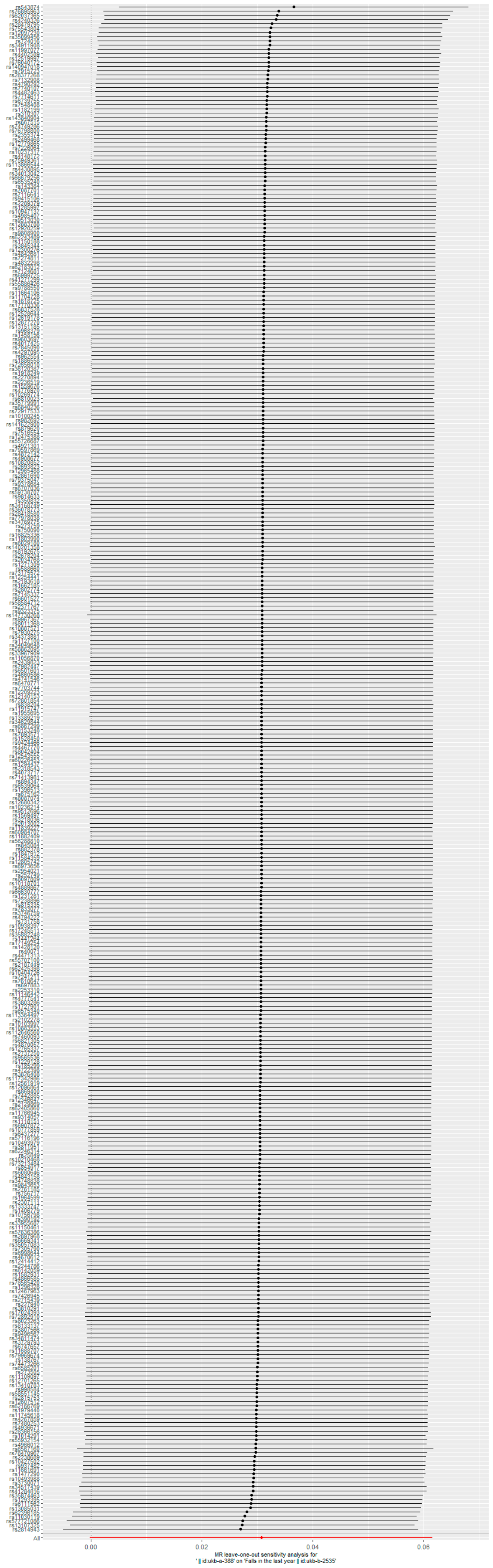

All
0.00
0.02
0.04
0.06
MR leave-one-out sensitivity analysis for
' || id:ukb-a-388' on 'Falls in the last year || id:ukb-b-2535'

(G)

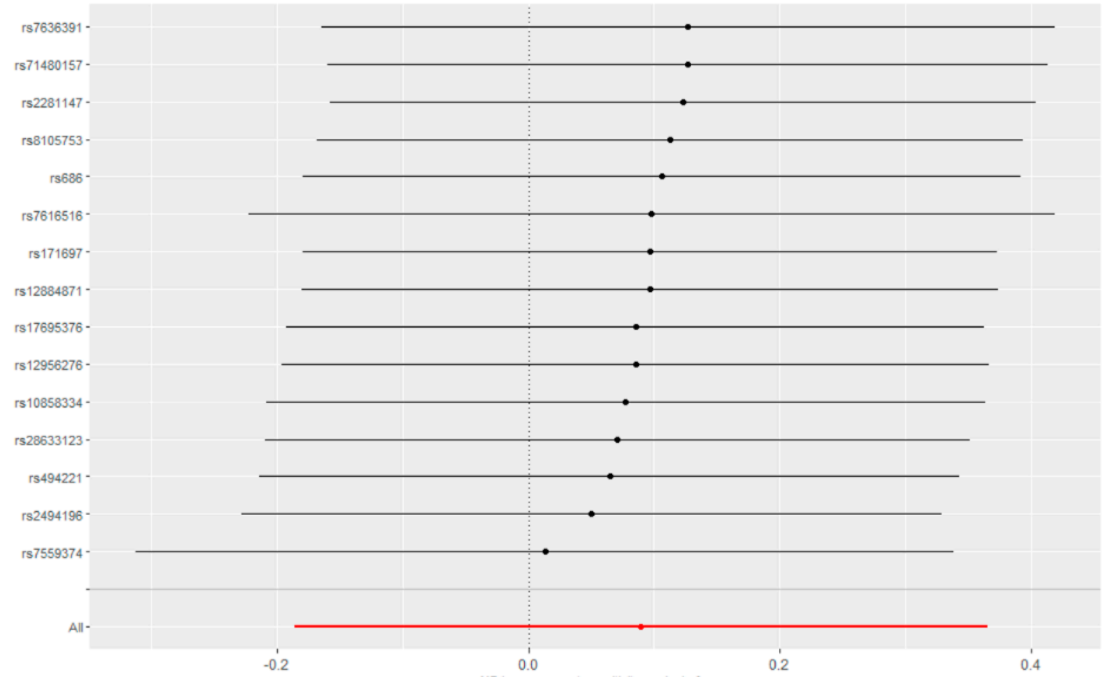
rs7636391
rs71480157
rs2281147
rs8105753
rs686
rs7616516
rs171697
rs12884871
rs17695376
rs12956276
rs10858334
rs28633123
rs494221
rs2494196
rs7559374
All
-0.2
0.0
0.2
0.4

(H)

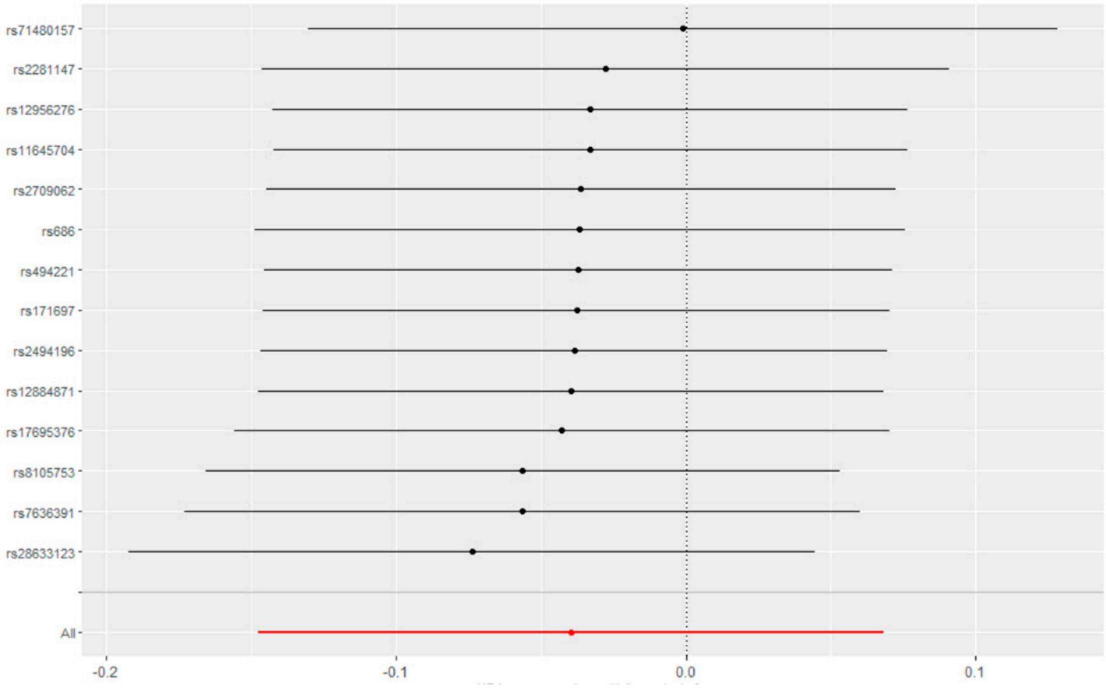
rs71480157
rs2281147
rs12956276
rs11645704
rs2709062
rs686
rs494221
rs171697
rs2494196
rs12884871
rs17695376
rs8105753
rs7636391
rs28633123
All
-0.2
-0.1
0.0
0.1

(I)

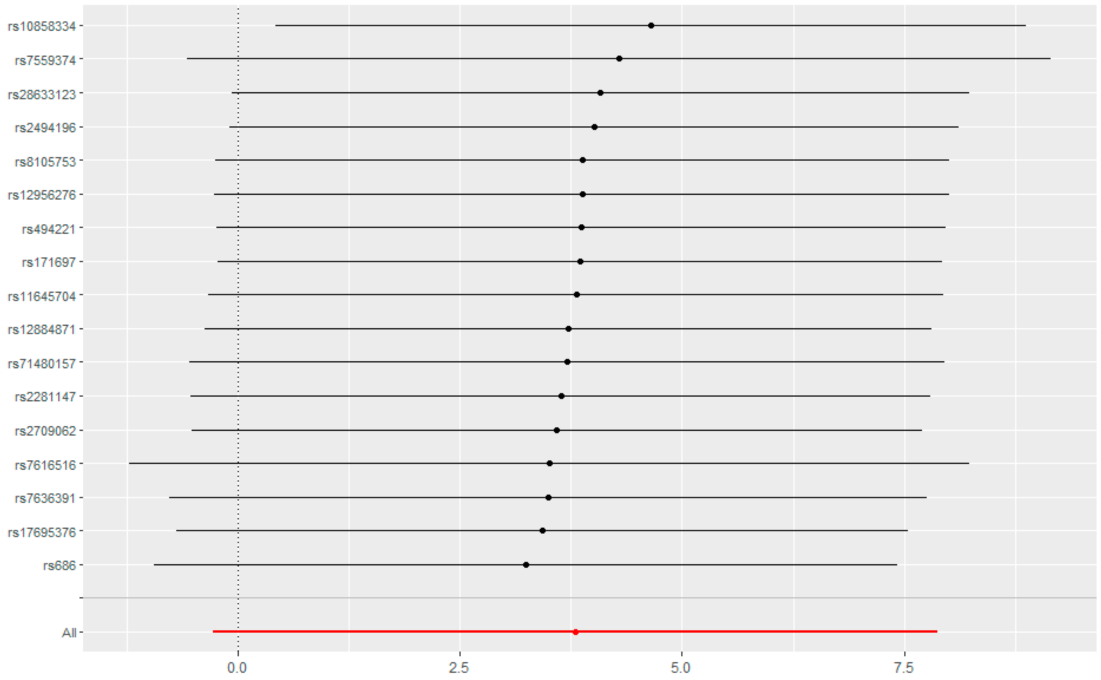

Figure S5 Leave-one-out plots between exposures and outcomes.

(A-F) Leave-one-out plots presenting the relationship between waist circumference, rheumatoid arthritis, sleeplessness, BMI, osteoporosis, hip circumference, and falls. (H-I) Leave-one-out plots present the relationship between falls and fractures, epilepsy, and stroke.

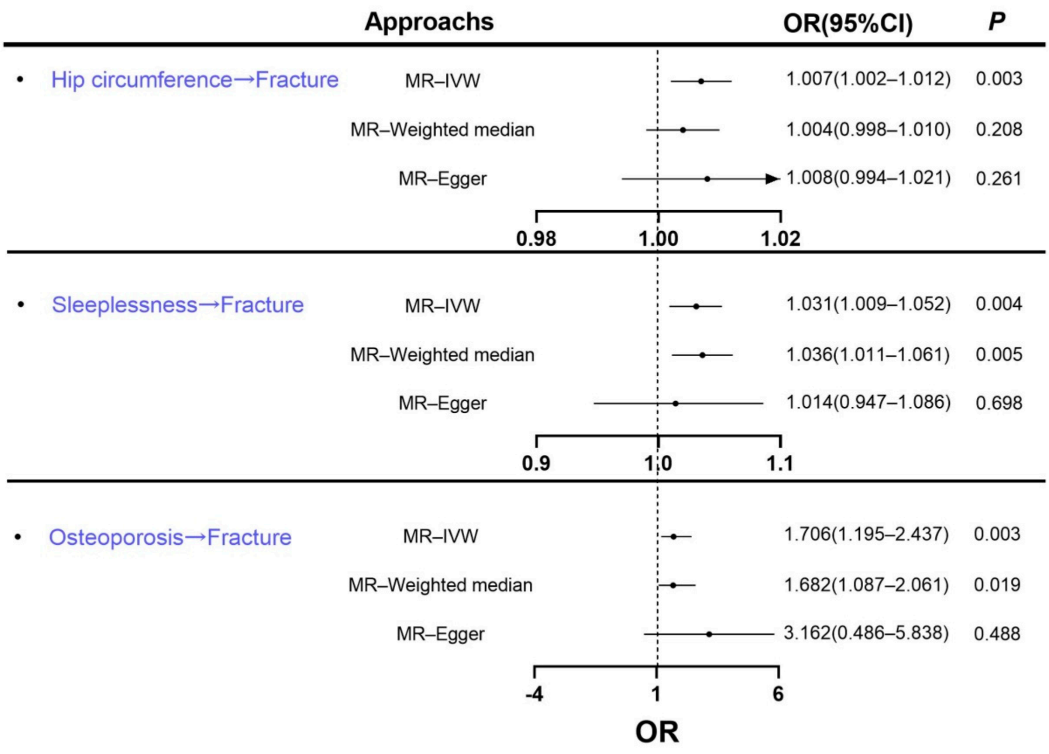

Figure S6 Odds ratios and 95% confidence intervals for the effects of hip circumference, sleeplessness, and osteoporosis on fracture estimated by univariable MR.

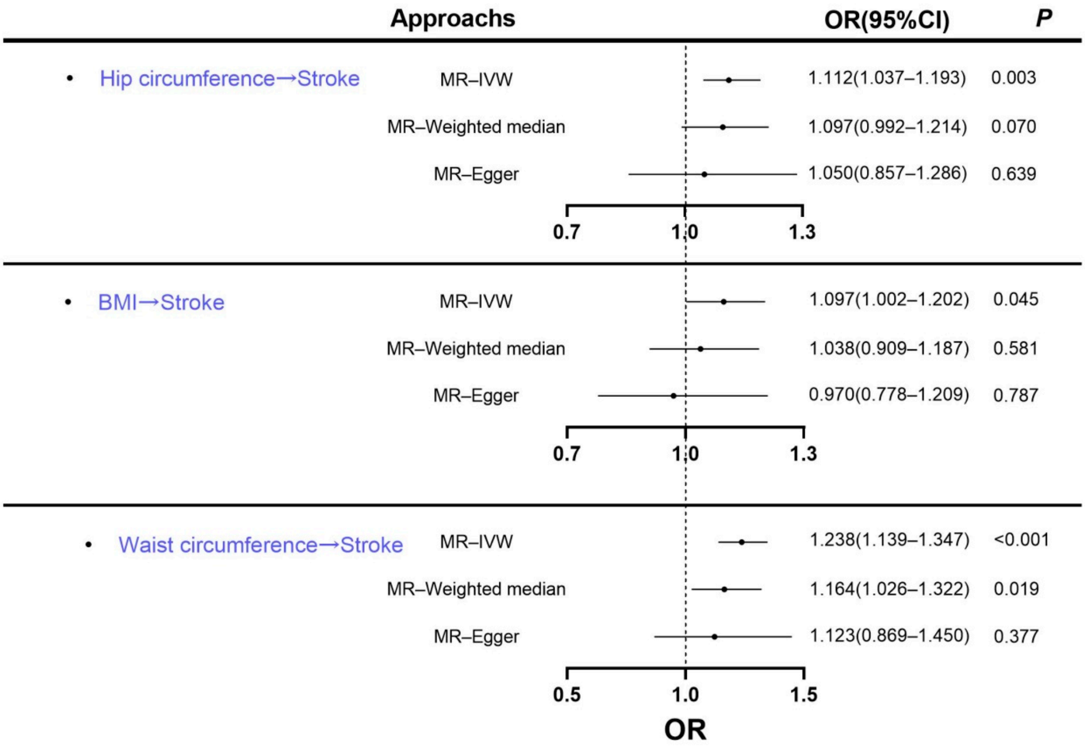

Figure S7 Odds ratios and 95% confidence intervals for the effects of hip circumference, BMI, and waist circumference on stroke estimated by univariable MR.

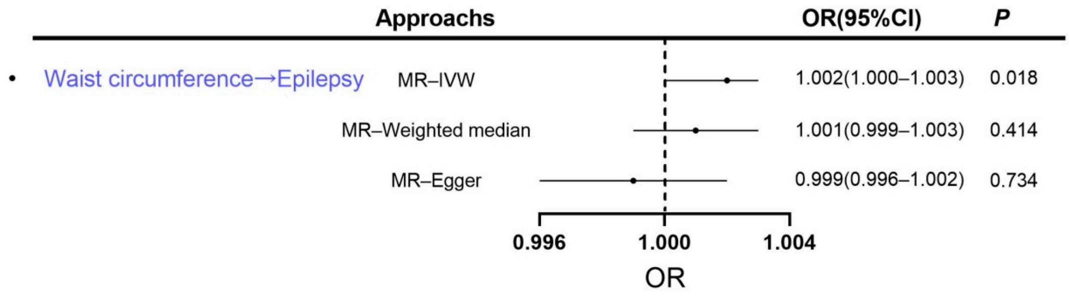

Figure S8 Odds ratios and 95% confidence intervals for the effect of waist circumference on epilepsy estimated by univariable MR.
